# Mirror proteases of Ac-Trypsin and Ac-LysargiNase precisely improve novel event identifications in *Mycolicibacterium smegmatis* MC^2^ 155 by proteogenomic analysis

**DOI:** 10.3389/fmicb.2022.1015140

**Published:** 2022-10-12

**Authors:** Songhao Jiang, Jiahui Shi, Yanchang Li, Zhenpeng Zhang, Lei Chang, Guibin Wang, Wenhui Wu, Liyan Yu, Erhei Dai, Lixia Zhang, Zhitang Lyu, Ping Xu, Yao Zhang

**Affiliations:** ^1^Key Laboratory of Microbial Diversity Research and Application of Hebei, School of Life Sciences, Hebei University, Baoding, China; ^2^Beijing Proteome Research Center, National Center for Protein Sciences Beijing, State Key Laboratory of Proteomics, Research Unit of Proteomics and Research and Development of New Drug of Chinese Academy of Medical Sciences, Institute of Lifeomics, Beijing, China; ^3^Guangzhou University of Chinese Medicine, Second Clinical Medicine College, Guangzhou Higher Education Mega Center, Guangzhou, China; ^4^Research Unit of Proteomics and Research and Development of New Drug, Institute of Medicinal Biotechnology, Chinese Academy of Medical Sciences and Peking Union Medical College, Beijing, China; ^5^The Fifth Hospital of Shijiazhuang, School of Public Health, Shijiazhuang, China; ^6^Key Research Laboratory for Infectious Disease Prevention for State Administration of Traditional Chinese Medicine, Tianjin Institute of Respiratory Diseases, Haihe Hospital, Tianjin University, Tianjin, China

**Keywords:** *Mycolicibacterium smegmatis*, proteogenomics, Ac-Trypsin, Ac-LysargiNase, mirror

## Abstract

Accurate identification of novel peptides remains challenging because of the lack of evaluation criteria in large-scale proteogenomic studies. Mirror proteases of trypsin and lysargiNase can generate complementary *b*/*y* ion series, providing the opportunity to efficiently assess authentic novel peptides in experiments other than filter potential targets by different false discovery rates (FDRs) ranking. In this study, a pair of in-house developed acetylated mirror proteases, Ac-Trypsin and Ac-LysargiNase, were used in *Mycolicibacterium smegmatis* MC^2^ 155 for proteogenomic analysis. The mirror proteases accurately identified 368 novel peptides, exhibiting 75–80% *b* and *y* ion coverages against 65–68% *y* or *b* ion coverages of Ac-Trypsin (38.9% *b* and 68.3% *y*) or Ac-LysargiNase (65.5% *b* and 39.6% *y*) as annotated peptides from *M. smegmatis* MC^2^ 155. The complementary *b* and *y* ion series largely increased the reliability of overlapped sequences derived from novel peptides. Among these novel peptides, 311 peptides were annotated in other public *M. smegmatis* strains, and 57 novel peptides with more continuous *b* and *y* pairs were obtained for further analysis after spectral quality assessment. This enabled mirror proteases to successfully correct six annotated proteins' N-termini and detect 17 new coding open reading frames (ORFs). We believe that mirror proteases will be an effective strategy for novel peptide detection in both prokaryotic and eukaryotic proteogenomics.

## Introduction

Proteogenomics has emerged as the inter-discipline of genomics and proteomics, which was proposed by Jaffe in 2004 (Jaffe et al., 2004). This field has been driven by advances in various sequencing and proteomic strategies (Renuse et al., 2011; Menschaert and Fenyo, 2017; Ang et al., 2019). High throughput sequencing technologies have increased the speed of study and the depth of data coverage (Ruggles et al., 2017). The integration of multi-omic datasets allows not only for the improvement in gene re-annotation (Castellana and Bafna, 2010; de Souza et al., 2011; Herbst et al., 2019; Li et al., 2020) but also for the identification of novel peptides or their variants in precision medicine (Zhang et al., 2016a; Ferrarotto et al., 2021; Dong et al., 2022).

Novel peptides detected by proteogenomics provide unpredictable information and insights into basic and disease research, which are difficult to identify by traditional annotating strategies (Castellana and Bafna, 2010). However, a primary challenge for newly identified peptides is filtering them out from the bloated database and evaluating their authenticity. Erroneous identification might be caused by incomplete fragmentation, noise, and “isometric” peptides (Castellana and Bafna, 2010). To assess novel peptide identification, we used their scoring and ranking of the match of experimental and theoretical spectra in proteogenomic analysis based on different error-rate estimation methods (Zhang et al., 2015; Li et al., 2017; Aggarwal et al., 2022).

There is a rare application for improving continuous and complementary ion fragments in novel peptide evaluation. Huesgen et al. and Yang et al. found that mirror proteases, trypsin, and lysargiNase can generate a deeper *b*/*y* ion coverage because of their C- and N-terminal digestion characteristics, which allowed precision *De Novo* peptide sequencing in the large-scale proteome. In contrast with LysC and LysN (Raijmakers et al., 2010) and other mirror proteases (Fossati et al., 2021), trypsin and lysargiNase have higher digesting specificity and generate more peptides with less complexity, providing a greater opportunity to increase proteomic coverage.

In addition, we found that in-house developed proteases, acelyted Trypsin (Ac-Trypsin; Wu et al., 2016) and LysargiNase (Ac-LysargiNase; Zhang et al., 2019), demonstrate lower self-digestion, superior stability, and higher activity than their recombinational and commercial products, whether in simple protein substrate or quantitative proteomics studies.

In this study, we proposed mirror proteases, Ac-Trypsin and Ac-LysargiNase, which can rapidly and accurately select and assess novel peptides by dramatically improving both *b* and *y* ion coverage. Compared with 65–68% *b* or *y* ion coverage from a single protease, complimentary use of Ac-Trypsin and Ac-LysargiNase increased both *b* and *y* ion coverage to 75–80%, which allowed us to obtain 368 novel peptides with high-quality spectra in *Mycolicibacterium smegmatis* MC^2^ 155. We used these veritable novel peptides to correct six recorded proteins' N-termini and identify 17 novel ORFs. Besides improving gene reannotation on *M. smegmatis* MC^2^ 155, the conserved homology of novel ORFs can also be reannotated in other closely related genetic species in the family *Mycobacteriaceae*.

## Materials and methods

### Strain culture and protein sample preparation

^**1**5^NH_4_Cl was purchased from Cambridge Isotope Laboratories, Andover, MA, United States. *M. smegmatis* MC^2^ 155 was cultured in an M9 minimal medium with ^15^N metabolite labeling described previously (Zhu et al., 2022). Briefly, the ^14^NH_4_Cl- and ^15^NH_4_Cl -labeled cells were equally mixed and disrupted with a Soniprep sonicator (Scientz, Ningbo, China) for 15 min (2 s-on, 4 s-off) at 30% amplitude. Supernatants were collected after centrifugation at 13,000 rpm for 15 min, and protein concentration was measured by a gel-assisted method as described previously (Zhang et al., 2016b).

### In-gel Ac-Trypsin and Ac-LysargiNase digestion

To achieve deep coverage of *M. smegmatis* proteome, we reduced 240 μg of proteins with 5 mM of dithiothreitol (DTT) and alkylated them with 20 mM of iodoacetamide (IAA). The alkylated proteins were split into two samples, separated by a 10% SDS-PAGE for 8 cm, and stained with Coomassie Blue G250. Two gel lanes were excised into 13 fractions based on the molecular weight (MW) and protein abundance and digested with Ac-Trypsin (Wu et al., 2016; 12.5 ng/μL) and Ac-LysargiNase (Zhang et al., 2019; 12.5 ng/μL) at 37°C for 12–24 h. The extracted peptides were desalted with a homemade C_18_ StageTip (Zhai et al., 2013), dried, and dissolved in loading buffer (1% acetonitrile, ACN, and 1% formic acid, FA) for MS analysis.

### LC-MS/MS analysis

The dissolved peptides (500 ng) were analyzed, as described previously. Briefly, the liquid chromatography-tandem mass spectrometry (LC-MS/MS) consisted of an EASY-nLC 1200 system (Thermo Fisher Scientific, San Jose, CA, United States) equipped with a self-packed capillary column (75 μm i.d. × 15 cm, 3 μm C_18_ reversed-phase fused silica) coupled to an Orbitrap Fusion Lumos (Thermo Fisher Scientific). For full MS scans, the automatic gain control (AGC) was set at 5.0 × 10^5^. The scan ranged from 300 to 1,400 *m/z* at a resolution of 1.2 × 10^5^ and a maximum injection time (MIT) of 50 ms. For the MS_2_ scan, only spectra with a charge state of 2–6 were selected for fragmentation by higher energy collision-induced dissociation (HCD) with a normalized collision energy of 32%, an AGC of 1 × 10^5^, and an MIT of 35 ms. The dynamic exclusion was set to 30 s.

### Database construction and searching

The annotated protein database of *M. smegmatis* MC^2^ 155 was downloaded from NCBI (NZ_CP009494, https://www.ncbi.nlm.nih.gov/genome/1026?genome_assembly_id=212020), including 6,385 entries. Using pAnno (Zhang et al., 2015) in pFind software, the six-frame database was constructed based on the complete genome sequence of MC^2^ 155 according to a stop-to-stop translating strategy [183,120 entries with at least seven amino acids (AAs)]. Two MS/MS datasets digested with different proteases were analyzed using pFind (v 3.1.5; Chi et al., 2018) to search against the annotated and six-frame databases. For the two digestion modes, trypsin and lysargiNase, a maximum of two missed cleavages was tolerated; 20 ppm was tolerated in MS and MS/MS search modes. Cysteine carbamidomethyl was set as a fixed modification, whereas acetylation of protein N-termini and methionine oxidation were added as variable modifications. A minimal peptide length of 7 AAs was required. The false discovery rates (FDR) for the peptide-spectrum match (PSM), peptide, and protein were set to < 1%. ^15^N labeling mode was chosen for novel peptide verification.

### Novel peptide selection and verification

The identified peptides were from three types: annotated proteins, novel ORFs, and N-terminal corrections according to the encoding gene position. The potential novel peptides mapping to the N-terminal extension of the annotated proteins and the unannotated regions were obtained and evaluated for their accuracy by “mirror proteases” digesting the evidence, Q-value, raw score, and ^14^N and ^15^N spectrum similarity. A Q-value was calculated based on the target-decoy approach. A raw score is the reliability of the PSM. ^14^N and ^15^N spectrum similarity was calculated by pBuild in pFind software, as described previously (Zhu et al., 2022). In addition, novel peptides with higher, moderate, and lower scores were selected and synthesized for further verification. The spectrum similarity of original and synthesized peptides was checked. A Student's *t*-test was calculated by R (^***^P < 0.001, ^**^P < 0.01, ^*^P < 0.05).

### Reanalysis of public RNA-seq and Ribo-seq datasets

The public RNA-seq (SRR17866681, 2-Feb-2022) and Ribo-seq (E-MTAB-2929, 1-Jan-2015; Shell et al., 2015) datasets were downloaded from NCBI and ArrayExpress, respectively, and aligned to reference DNA sequence by HISAT2 (v 2.2.1; Kim et al., 2019), and SAMtools (v 1.11; Li et al., 2009). The expression of RNA-seq and Ribo-seq was calculated by Cufflinks (v2.2.1; Trapnell et al., 2012) locally and visualized by IGV (v 2.11.1; Thorvaldsdottir et al., 2013) and R (v 4.1.2).

### N-terminal labeling and negative enrichment

A total of 150 μg proteins were reduced by 10 mM dithiothreitol (DTT) at 45°C for 60 min and alkylated by 20 mM IAA for 45 min at room temperature. Then, 40 mM formaldehyde (Sigma-Aldrich, Saint Louis, MO, United States) and 20 mM sodium cyanoborohydride (Sigma-Aldrich) were added to the above protein sample and incubated at 37°C overnight. A solution of 1 M Tris (pH 6.8) with a final concentration of 0.1 M was added to quench the reaction.

The dimethylated proteins were digested with Ac-Trypsin at a protein/enzyme ratio of 50:1 (w/w) at 37°C for 14 h. The high molecular weight dendritic hyperbranched polyglycerol-aldehydes (HPG-ALD, Vancouver, BC, Canada; Kleifeld et al., 2010) polymer was added to the peptide sample. 20 mM sodium cyanoborohydride was immediately added and incubated overnight at 37°C. The naturally blocked and experimentally labeled N-terminal peptides were collected by ultrafiltration with the 30 kDa MWCO Amicon column (Sartorius, Gottingen, Germany).

The N-terminal peptide sample was fractionated by the RP-Tip (Reverse-Phase Tip; Ni et al., 2019) with an increasing acetonitrile step-gradient (6, 9, 12, 15, 18, 21, 25, 30, 35, and 50%, pH = 10). These 10 fractions were combined into six samples (6 + 25%, 9 + 30%, 12 + 35%, 15%, 18%, and 21 + 50%) and dried for LC-MS/MS analysis.

Six raw files were searched against the six-frame database of *M. smegmatis* MC^2^ 155 with pFind. The parameters of database searching were as follows: (1) a maximum of three missed cleavages were tolerated; (2) semi-specific cleavage was set; (3) 20 ppm was tolerated in MS and MS/MS search modes; (4) cysteine carbamidomethyl and dimethyl at lysine were set as fixed modification; (5) acetylation of any N-termini, dimethylation at any N-terminal, pyroglutamate at glutamate and glutamine, and oxidation of methionine were added as variable modifications; (6) A minimal peptide length of 7 AAs was required, and (7) the false discovery rates (FDR) for the peptide-spectrum match (PSM); peptide and protein were set to < 1%.

### Correction of N-termini

The N-terminal-derived sequences were verified by a comparative genomic approach.

Firstly, the newly translated stop-to-stop protein sequences were compared with NCBI publicly annotated sequences using BLASTP. Secondly, the potential expression at the transcriptional and translational levels was checked by the public RNA-seq and Ribo-seq datasets of *M. smegmatis* MC^2^ 155, respectively. Thirdly, the potential start codon was confirmed based on the Ribo-seq and direct evidence of N-terminal labeling with dimethylation.

### Verification of novel ORFs

For novel ORFs, novel peptides were assessed by continuous *b/y* pairs and spectra quality. All potential novel encoding ORFs were rechecked in their annotating state with the publicly available annotated proteins in the NCBI database by BLASTP. The matched orthologous protein sequences were used to construct neighbor-joining (NJ) phylogenetic trees of the novel ORFs encoding proteins by MEGA software (v 10.1.8; Kumar et al., 2018). To observe the conservation of novel ORFs, we used their nucleotide sequences to compare them with 207 reference strains in *Mycobacteriaceae* by local BLASTN analysis. Sequences sharing at least 60% sequence coverage and 70% identity with other genetically distant species belong to conserved genes and can also be used for reannotation for their homologous genes, as described previously (Gallien et al., 2009).

## Results

### Experiment design

**Figure 1:**
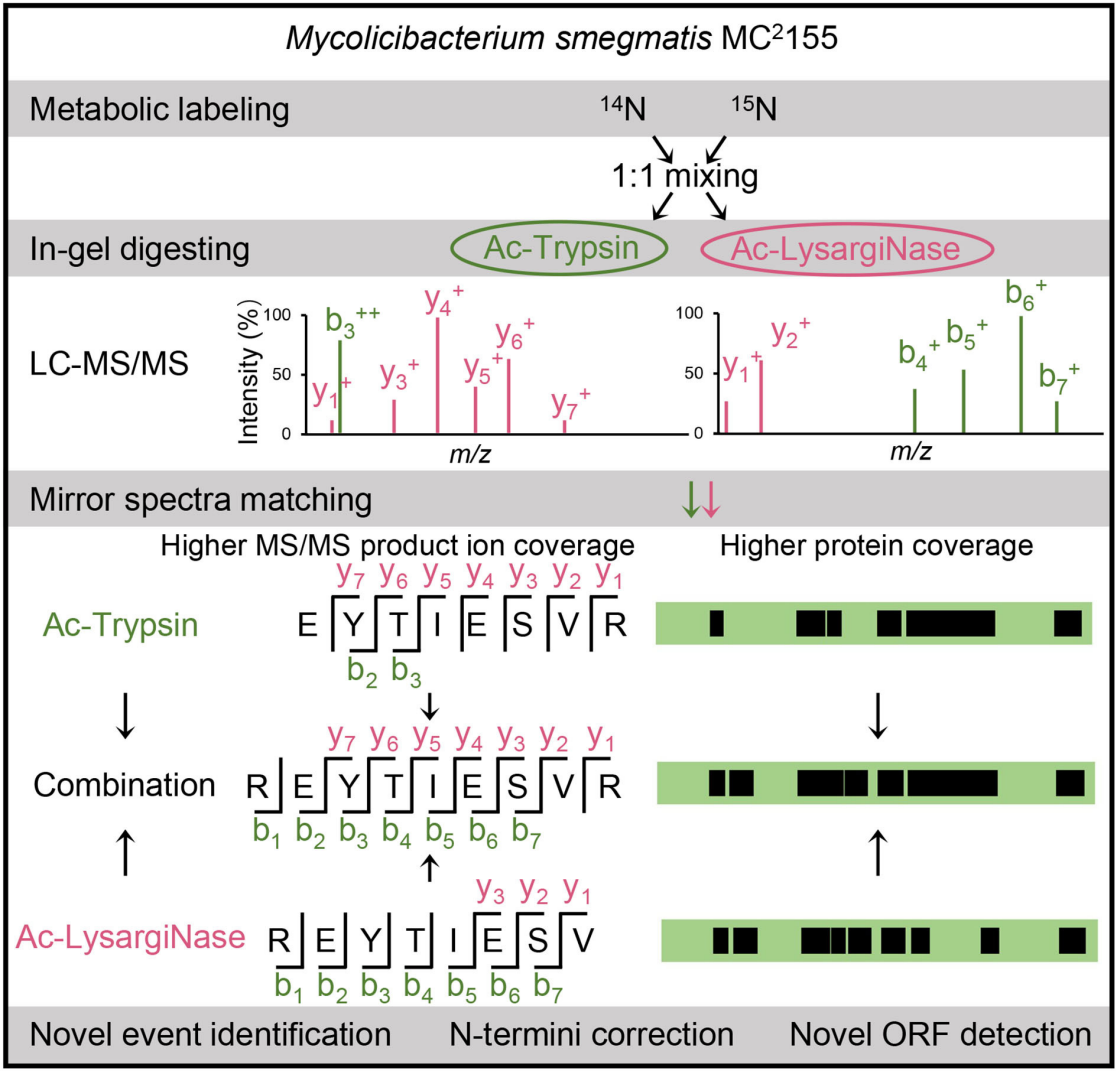
Workflow of this study. Our in-house mirror proteases, Ac-Trypsin and Ac-LysargiNase, were used to assess the authenticity of novel peptides in a large-scale proteogenomic study based on ^14^N- and ^15^N-labeling cells of *M. smegmatis* MC^2^ 155. The verified novel peptides were further used for novel event analysis, including N-termini corrections and novel ORF identifications.

To achieve deep coverage of proteomics for *M. smegmatis* MC^2^ 155, we separated two same samples by a 10% SDS-PAGE gel (8 cm) and digested by Ac-Trypsin (Wu et al., 2016) and Ac-LysargiNase (Yang et al., 2019; Zhang et al., 2019) with high activity and stability (Supplementary Figure 1). The extracted peptides were detected by an Orbitrap Fusion Lumos mass spectrometer under the same conditions. The raw files from different proteases were searched against the annotated database for evaluating protein coverage and the six-frame database for selecting novel peptides. To evaluate the contribution of mirror proteases to the accurate identification of novel peptides, we extracted the merging spectra from mirror peptides and calculated the coverage of the *b*/*y* ion series. Here, mirror peptides were the overlapped sequences digested simultaneously by Ac-Trypsin and Ac-LysargiNase. In addition, two or more unique peptides from the same novel events were also conserved because of different digesting characteristics. These novel peptides were further assessed by spectra score prediction and their ^15^N-labeling spectra matching. All verified novel peptides were used for the genomic reannotation of *M. smegmatis* MC^2^ 155 (Figure 1).

### Large-scale mirror proteome of *M. smegmatis* by Ac-Trypsin and Ac-LysargiNase

**Figure 2:**
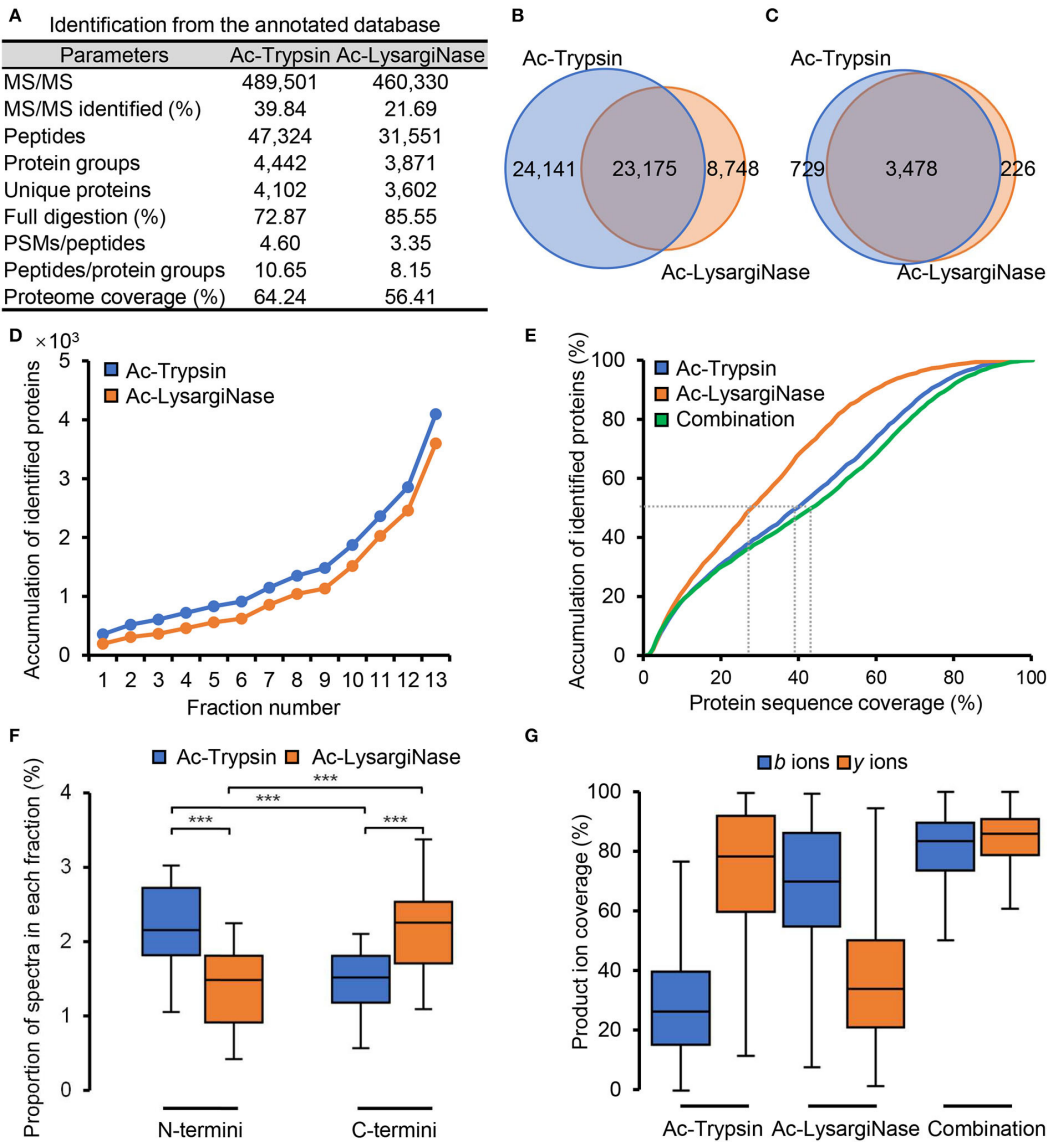
Mirror proteases helpfully improved the proteome coverage of *M. smegmatis* MC^2^ 155 based on the annotated database searching. **(A)** The application of Ac-Trypsin and Ac-LysargiNase in proteomics of *M. smegmatis* MC^2^ 155. Comparison of the identified peptides **(B)** and proteins **(C)** by Ac-Trypsin and Ac-LysargiNase. **(D)** Protein identification saturation using gel-separation methods by Ac-Trypsin and Ac-LysargiNase. **(E)** Comparison of the protein sequence coverage by single protease and combined mirror proteases. **(F)** The proportion of the N-terminal and C-terminal spectra in each fraction from Ac-Trypsin and Ac-LysargiNase digests. **(G)** Comparison of the ion coverages from single protease and combined mirror proteases for annotated peptides. Statistically significant differences by student's *t*-test are indicated for *p* values of ^*^p < 0.05, ^**^p < 0.01, and ^***^p < 0.001.

In Supplementary Figure 2, Ac-Trypsin and Ac-LysargiNase showed higher specificity with more than 95% based on the annotated protein database searching, which also resulted in the identification of more fully digested peptides. In Ac-Trypsin digestion datasets, 47,324 peptides were detected from 4,442 protein groups and 4,102 unique proteins (Supplementary Table 1). In the Ac-LysargiNase datasets, 31,551 peptides were from 3,871 protein groups and 3,602 unique proteins (Supplementary Table 2). The average ratio of the PSM count per peptide and peptides per protein group was more than three and eight (Figure 2A). Among these peptides, 23,175 (40.94%) were mirror peptides, 24,141 unique peptides were from Ac-Trypsin, and 8,748 peptides from Ac-LysargiNase digests (Figure 2B, Supplementary Table 3). At the protein level, 3,478 (78.46%) were shared in two different datasets (Figure 2C), indicating that a larger complement and verification datasets were obtained. The number of non-redundant proteins increased quite steeply and fast with the addition of gel fraction, suggesting the high separation resolution of the gel we used in this study (Figure 2D, Supplementary Figure 1). The total number of unique proteins rose to 4,102 and 3,602 for the Ac-Trypsin and Ac-LysargiNase datasets, which accounted for 64.24 and 56.41% of the total annotated proteins for *M. smegmatis* MC^2^ 155. In addition, the average protein sequence coverage increased from 27.59% of Ac-LysargiNase and 39.15% of Ac-Trypsin to 42.99% when two separation methods were introduced (Figure 2E, Supplementary Figure 3), indicating a high overlap region of the sequenced peptides and high specificity as well as activity of both proteases we applied in this study.

Due to the cleavage characteristics of proteases, Ac-Trypsin and Ac-LysargiNase digested proteins included significantly more N-terminal and C-terminal peptides, respectively (Figure 2F, *P* < 0.001; Huesgen et al., 2015). The spectra from these Ac-Trypsin and Ac-LysargiNase digesting peptides had strong *y* and *b* series ions, which led to different *y* and *b* ion coverages for both Ac-Trypsin and Ac-LysargiNase, respectively. The medium of *b* and *y* ion coverages was 29.70 and 74.69% for the Ac-Trypsin spectra, respectively, and 69.37 and 37.01% for the Ac-LysargiNase spectra. In comparison, both *y* and *b* ion coverages were improved to >80% when we combined two proteases (Figure 2G, Supplementary Figure 3). We further investigated *b* and *y* ion coverages at different positions of all identified peptides. We found that Ac-Trypsin and Ac-LysargiNase spectra could provide complementary ions for whole peptides, including N-terminal, middle, and C-terminal AAs (Supplementary Figure 4). These results support the high quality and deep coverage of *M. smegmatis* proteome generated by mirror proteases, providing verifiable MS evidence and an opportunity to identify novel peptides.

### Ac-Trypsin and Ac-LysargiNase efficiently identified novel peptides

**Figure 3:**
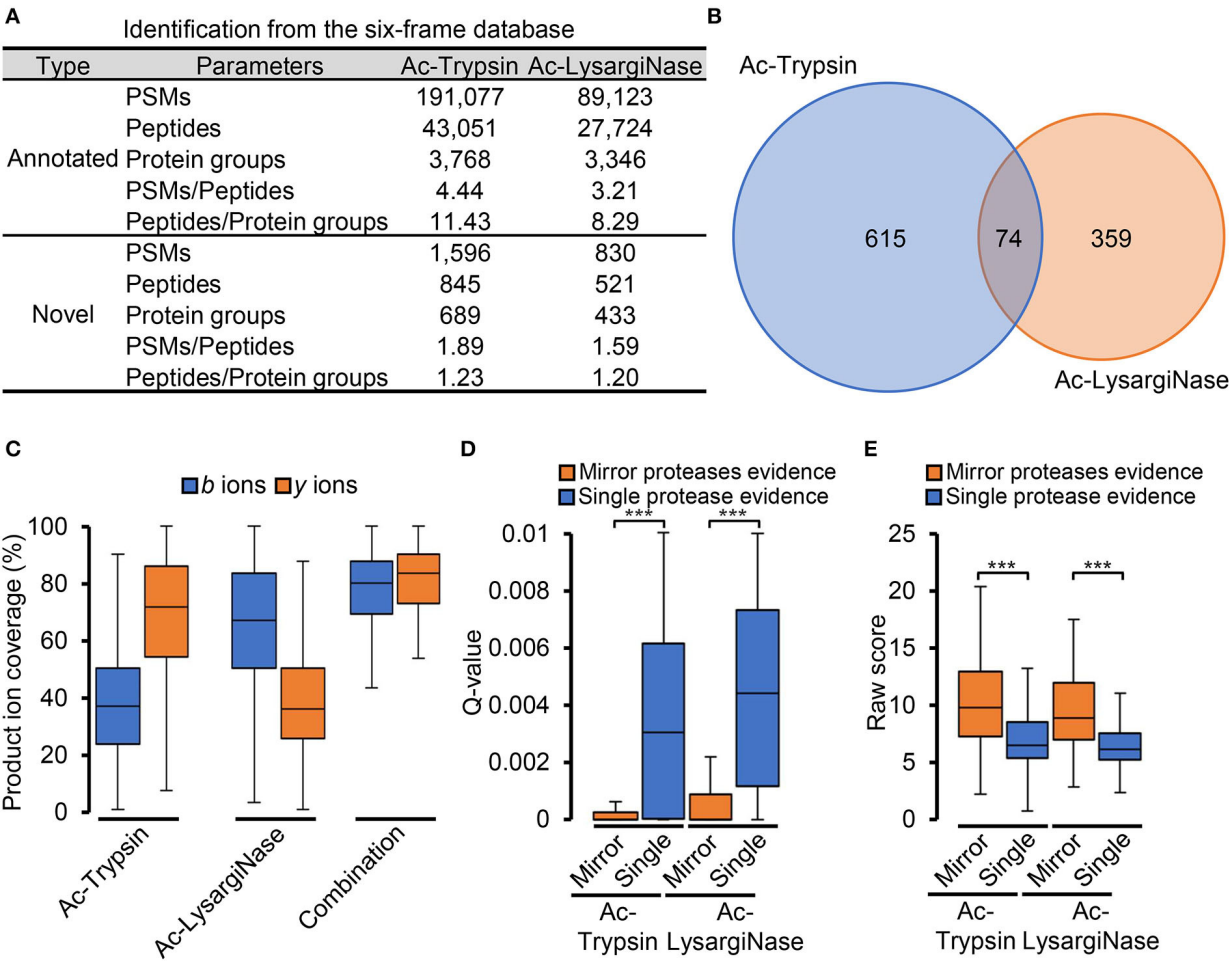
Mirror proteases efficiently identified credible novel peptides. **(A)** The proteogenomic identification is based on the six-frame database of *M. smegmatis* MC^2^ 155. **(B)** Venn diagram of the proteins identified from the Ac-Trypsin and Ac-LysargiNase datasets. **(C)** Comparison of the ion coverages from single protease and combined mirror proteases for novel peptides. Comparison of the *Q*-value **(D)** and raw score **(E)** spectra with single protease and mirror proteases digesting evidence from the Ac-Trypsin and Ac-LysargiNase datasets.

After the six-frame database search, 845 and 521 novel peptides were identified from Ac-Trypsin and Ac-LysargiNase digests (Figure 3A, Supplementary Tables 4–6). The average ratios for the number of PSMs per novel peptide and novel peptides per protein group were 1.78 and 1.22, which were significantly lower than those of annotated gene products for either Ac-Trypsin or Ac-LysargiNase digested proteome samples. In total, 126 and 119 peptides from 845 Ac-Trypsin and 521 Ac-LysargiNase digests were mirror peptides, respectively. Among them, 111 truncated sequences have the same AAs consistently from Ac-Trypsin and Ac-LysargiNase digests, excepting N- or C-terminal AAs (Supplementary Table 6, Supplementary Figure 5), which resulted in the identification of 74 shared proteins (Figure 3B).

As annotated peptides, Ac-Trypsin and Ac-LysargiNase mirror spectra provided complementary *b* and *y* ions with about 80% coverage for novel peptides compared with those of Ac-Trypsin (*b*-ion, 37.88%; *y*-ion, 68.25%) or Ac-LysargiNase (*b*-ion, 65.49%; *y*-ion, 39.60%; Figure 3C). To further evaluate the accuracy of these novel peptides, we compared the *Q*-value and raw score of all spectra with single protease and mirror protease evidence. In Figure 3D, the average *Q*-value of spectra for mirror peptides was 4.91 times lower than that from Ac-Trypsin (single protease, 0.0035; mirror proteases, 0.0007) and 4.22 times lower than that from Ac-LysargiNase (*t*-test *P* < 0.0001; single protease, 0.0044; mirror proteases, 0.0010). However, the average raw score of spectra with two proteases digesting evidence was 1.49 times higher than that from Ac-Trypsin (single protease, 6.79; mirror proteases, 10.12) and 1.41 times higher than that from Ac-LysargiNase (*t*-test *P* < 0.0001; single protease, 6.46; mirror proteases, 9.14, Figure 3E). These results strongly supported that the novel peptides digested by mirror proteases were more confident than those from the single protease digested samples.

A total of 368 novel mirror peptides were observed and used for proteogenomic analysis. According to the genomic position, these 368 novel peptides were identified from two novel events. Among them, 145 peptides were localized to the N-termini extension regions of 40 annotated genes, and the other 223 peptides from 34 ORFs were in the un-annotating regions of *M. smegmatis* MC^2^ 155 (Supplementary Table 6, Supplementary Figure 6A). To evaluate the annotation of these novel peptides, we compared their protein sequences to those in the NCBI nr database of other *M. smegmatis* stains by BLASTP. Results showed that as many as 126 novel peptides from N-termini extension and 185 from novel ORFs were annotated in other annotating versions or the other strains of *M. smegmatis*, implying that more than 80% of novel peptides were from encoding regions of other *M. smegmatis* known strains. The remaining 19 and 38 peptides (Supplementary Figures 6B,C) corresponded to six newly calibrated N-terminus and 17 newly identified ORFs (Supplementary Figures 6D,E).

Among the above mentioned 19 N-terminal novel peptides, 12 sequences were mirror peptides, and the other seven peptides were different products digested by mirror proteases (Supplementary Table 7). Among the other 38 novel peptides, 14 sequences belong to mirror peptides, one lysargiNase digested sequence was overlapped with another non-fully tryptic peptide, and the other 22 peptides were different products from mirror proteases digests (Supplementary Table 8). Complementary use of both proteases also increased *b*/*y* ion coverage and a sequence coverage of novel events (Supplementary Figures 6B,C). These results imply that mirror proteases provide a higher quality of novel peptides in genome reannotation.

### Novel peptides from mirror proteases efficiently corrected N-termini of 6 annotated proteins

**Figure 4:**
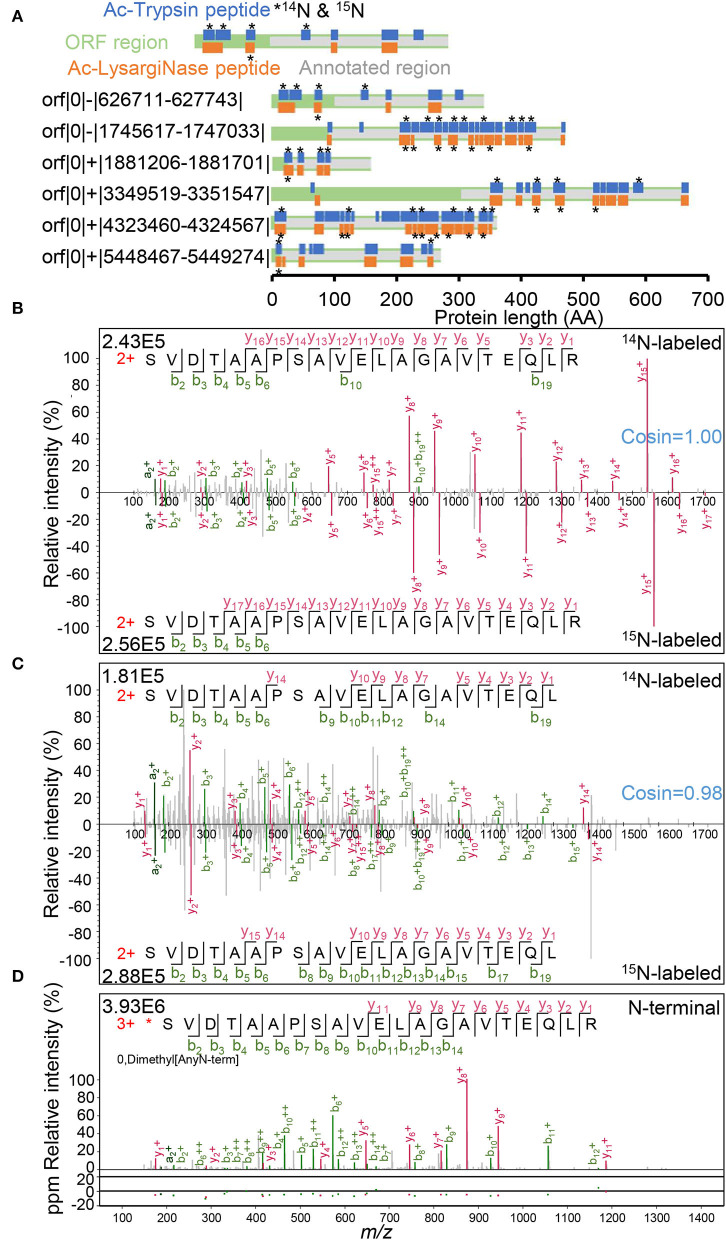
N-termini correction of 6 annotated proteins. **(A)** Novel peptides distribution in N-terminal extension regions. Peptide labeled star stands for ^14^N and ^15^N labeling spectra identification. The confirmed spectra of N-terminal peptides with ^14^N and ^15^N labeling forms were derived from the Ac-Trypsin **(B)** and Ac-LysargiNase **(C)** datasets, respectively. **(D)** The spectra of an N-termini labeled peptide with dimethyl modification from our N-terminomic dataset.

In the 19 novel N-terminal peptides from six annotated proteins identified from the complementary use of both proteases (Figure 4A, Supplementary Table 7, Supplementary Figure 6B), one ORF contained eight novel peptides, 1 ORF contained three peptides, and the other four ORFs each contained two peptides. Among them, 12 novel peptides were detected with essentially identical ^14^N and ^15^N spectra (cosin >0.9; Supplementary Figure 7). For orf|0|-|626711-627743| and orf|0|+|3349519-3351547|, novel peptides were distributed in front of the start codon GTG (V) and ATG (M) of previously annotated genes. For the other four ORFs, novel peptides covered the area of the originally erroneous start codons. Both mirror N-terminal novel sequences and ^14^N/^15^N double labeling spectra confirmed the authenticity of the N-termini correction for six recorded genes by Ac-Trypsin and Ac-LysargiNase (Figure 4A).

For example, both peptides (L)SVDTAAPSAVELAGAVTEQLR and (L)SVDTAAPSAVELAGAVTEQL(R) contained as many as 21 AA residues. These two peptides covered the original start codon GTG (V) of the annotated protein WP_011729667.1 for polyprenyl synthetase family protein (Figures 4B,C). As these two peptides are quite long, none of them were sequenced with either full *y* series or full *b* series product ions. Complementary with both Ac-Trypsin and Ac-LysargiNase, the sequences of all AA residues were confirmed with at least one product ion. These results proved the correctness and uniqueness of the peptide we identified. In addition to ^14^N-labeling spectra, their corresponding ^15^N-labeling forms were also identified as high quality. The ^14^N and ^15^N spectra were essentially the same as the cosin values of similarity (≈1.00) from two peptide pairs generated from Ac-Trypsin and Ac-LysargiNase. Further, we even identified one dimethyl-labeled N-termini peptide started with serine residue with a high-quality spectrum by a dimethylation labeling combined with a negative enrichment strategy (Figure 4D), ^*^SVDTAAPSAVELAGAVTEQLR, which included the previously erroneous codon-terminal valine. These results strongly support that the true start codon was CTG for leucine in front of the dimethyl-labeled serine residue considering the characteristics of aminopeptidase (L^*^S**V**DTAAPSAVELAGAVTEQLR, Figure 4D, Supplementary Figure 7).

In addition to mirror peptides, the novel N-terminal events were efficiently confirmed with different peptides identified from the Ac-Trypsin, and Ac-LysargiNase digested proteome. In the case of orf|0|+|3349519-3351547|, we identified two fully digested peptides, (R)GADHDVLR with three continuous *b*/*y* pairs and (L)RVQPDVGEL(R) with five *b*/*y* pairs, in N-terminal extension region of the recorded protein WP_011728939.1 for ABC transporter ATP-binding protein. Although its N-termini cannot be determined, the true start codon and a potential reason for this mis-annotation are worth elucidating.

In this study, we found erroneous N-termini in six annotated genes. Among them, three were previously annotated at the canonical start codon ATG, whereas the other three were at the non-canonical start codon GTG. Gallien et al. previously found overprediction of ATG and other non-canonical codons as the start codon using a gene prediction program by TMPP labeling strategy in *M. smegmatis* MC^2^ 155 (Gallien et al., 2009). Kelkar et al. and we also observed similar phenomena in *M. tuberculosis* (Kelkar et al., 2011; Shi et al., 2022). Other optimized multi-omic technologies, such as Ribo-RET (Meydan et al., 2019; Supplementary Figure 7) and RNA-seq, also provided corresponding expression evidence (Supplementary Figure 8), which further supported our finding through the complementary use of Ac-Trypsin and Ac-LysargiNase for deep coverage proteomics. On the other hand, as shown in Supplementary Figure 7, deep N-terminal proteomics would be the direct evidence for confirming the starting sites of encoding ORFs.

### Mirror proteases helped in the identification of 17 novel ORFs

**Figure 5:**
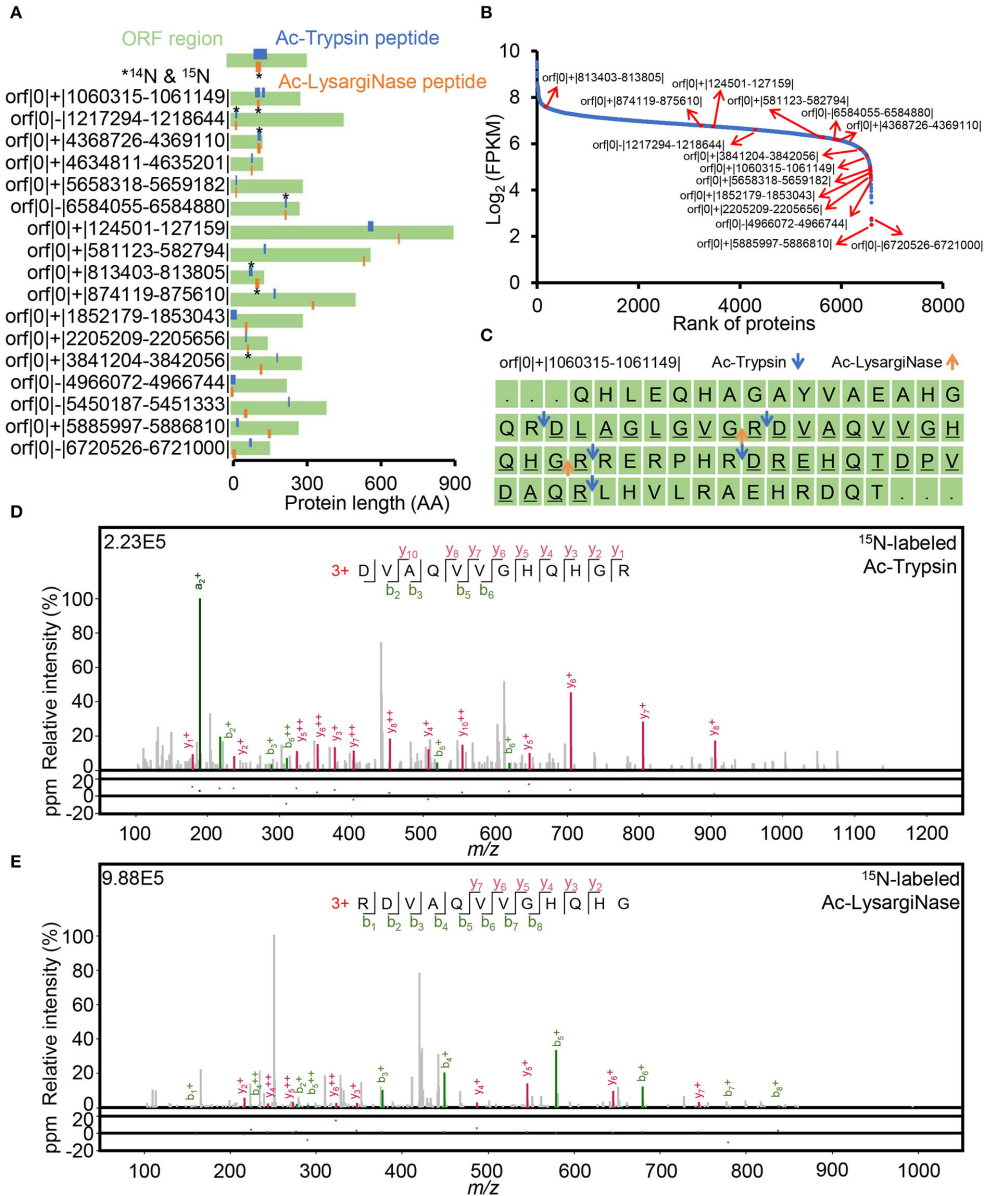
Verification of 17 novel ORFs. **(A)** novel peptide distribution in non-coding regions. Peptide labeled star stands for ^14^N and ^15^N labeling spectra identification. **(B)** FPKM rank of annotated and novel ORFs based on a public RNA-seq dataset. **(C)** Distribution of novel peptides digested by Ac-Trypsin and Ac-LysargiNase in orf|0|+|1060315-1061149|. The underlined sequences were identified as peptides derived from Ac-Trypsin and Ac-LysargiNase digestion in this study. The blue and orange arrows indicate the Ac-Trypsin and Ac-LysargiNase cleavage sites, respectively. The credible spectra of two different peptides, (R)DVAQVVGHQHGR and RDVAQVVGHQHG(R), from the Ac-Trypsin **(D)** and Ac-LysargiNase **(E)** dataset.

Of the identified 17 novel ORFs with 38 new peptides, six ORFs were detected with mirror peptides, which occupied more than 35% of all novel ORFs identified in this study. Among them, one ORF contained four novel peptides, two ORF contained three peptides, and the other 14 ORFs contained two peptides, indicating the high credibility of these new genes (Figure 5A, Supplementary Table 8). All the novel mirror peptides identified from these novel ORFs were specifically digested products. Among them, four tryptic peptides could also be identified from Blackburn (Potgieter et al., 2016) and Blackburn's (Giddey et al., 2017) datasets after the six-frame database research (Supplementary Table 8, Supplementary Figure 10), further confirming the credibility of these identifications in this study. Surprisingly, we even noticed that orf|0|+|813403-813805| showed quite a high abundance, ranking top 163 of all identified 6,596 genes at the mRNA level (Figure 5B, Supplementary Figure 12). While most of the novel ORFs had much low transcription abundance, which may suggest the deep coverage of the mirror proteome datasets. The reason for the missing annotation remains to be studied.

Mirror proteases also helped identify novel unannotated ORFs with high confidence. For example, four fully digested peptides were derived from the novel protein orf|0|+|1060315-1061149|, including a pair of mirror peptides, (R)DVAQVVGHQHGR and RDVAQVVGHQHG(R), and other two tryptic peptides, (R)DLAGLGVGR and (R)DREHQTDPVDAQR—with at least three continuous *b*/*y* ion pairs (Figure 5C).

For mirror peptides, (R)DVAQVVGHQHGR and RDVAQVVGHQHG(R) were identified by 3 and 4 *b*/*y* ion pairs in Ac-Trypsin and Ac-LysargiNase digests (Figures 5D,E). However, their meager theoretical peptide, RDVAQVVGHQHGR, five *b*/*y* ion pairs were efficiently matched with eight continuous *b* and *y* ions by combining Ac-LysargiNase and Ac-Trypsin digestion (Figures 5D,E). In addition, the mirror peptide, RDVAQVVGHQHG(R), was identified with identical ^14^N and ^15^N spectra (cosin ≈ 1.00). The sequenced AAs were 34 and accounted for 12.2% of the total stop-to-stop translating protein sequence. Most importantly, public Ribo-seq observed the confirmed translation initiation site (TIS) reads (Supplementary Figure 9) at the first leucine in orf|0|+|1060315-1061149| protein sequence, implying CTA (L) might be its start codon. The above evidence not only supported the confident identification but also indicated the N-termini of this novel ORF.

In one extreme case, our datasets detected two different peptides with identical ^14^N and ^15^N spectra from the novel gene orf|0|+|813403-813805|. The fully tryptic peptide (R)VATPGDSDASAQIEGLR was also identified from the Blackburn (Giddey et al., 2017) dataset, which is the mirror peptide of RVATPGDSDASAQIEGL(R) from our dataset. In addition, two other fully tryptic peptides, (R)LQQESEAFR and (R)WVTVVADALNSASSSGR, were detected in Blackburn's (Potgieter et al., 2016) and Blackburn (Giddey et al., 2017) work (Supplementary Figures 11A–C). Even more importantly, we successfully identified its N-terminal peptide (V)AAAGLAWAVSR with dimethyl modification at the first alanine in our demethylation-labeling N-terminomic dataset (Supplementary Figure 11D) and observed its expression signal at its TIS regions in a public Ribo-seq dataset, implying that the true start codon was GTC (V) in front of an N-terminal peptide and V might be removed by aminopeptidase (Gonzales and Robert-Baudouy, 1996). Thus, we efficiently identified N- and C-terminal peptides of orf|0|+|813403-813805| and confirmed its encoding sequence was 99 AAs. In total, six unique peptides were accurately identified from this novel small ORF, which covered 71.72% of its protein sequence. Together with the high transcription abundance, these results suggest the high abundance of this small novel ORF in *M. smegmatis* MC^2^ 155 proteome, which might play an important role in the biological process.

Unlike lysargiNase, trypsin-generated N-terminal peptides had C-terminal lysine or arginine residues. We identified 3 N-terminal peptides, (^*^)AVDVQHASVAVGARAALGAHR and (^*^)AVDVQHASVAVGA(R) from orf|0|-|4966072-4966744| with 224 AAs, and (^*^)PLVAPHPGDPAVVVGTLAAVQVEYVR from orf|0|+|1852179-1853043| with 288 AAs. (^*^)AVDVQHASVAVGA(R) was an uncompleted mirror peptide of (^*^)AVDVQHASVAVGARAALGAHR. These two different N-terminal peptides were derived from Ac-Trypsin, and Ac-LysargiNase digests, respectively. For novel ORF orf|0|+|1852179-1853043|, the other fully digested peptide RLGADEGIGLG(R) was also identified in Ac-LysargiNase digests. This spectra evidence supported the highly confident identification of novel ORFs and indicated the application of mirror proteases in genome re-annotation, even for unannotated ORFs.

### Homologous genes of novel ORFs did not annotate in other recorded strains

To investigate the sequence similarity of 17 novel ORFs, we compared their protein sequences with publicly sequenced and annotated strains by BLASTP in the NCBI reference database. Among these ORFs, 6 ORFs matched other annotated proteins with more than 50% coverage and 40% identity, six ORFs only matched less sequence with lower similarity, and five did not show any homology with recorded proteins in NCBI databases (Supplementary Table 8). Among six higher similarity ORFs, orf|0|+|4368726-4369110| might be a 2-oxoacid dehydrogenases acyltransferase family protein, orf|0|+|3841204-3842056| might be an ion transporter based on the function annotation, while other four ORFs were for hypothetical proteins.

Further BLASTN analysis of these novel ORFs found that 13 ORFs showed higher coverage ranging from 95 to 100% and similarity ranging from 85.71 to 93.57% with their ortholog genes in *Mycolicibacterium goodii*. Although 13 novel ORFs showed higher homology with those of genetically closer species, they have not been annotated in these stains by far.

The other 4 ORFs showed lower identity with NCBI sequenced strains. The orf|0|-|1217294-1218644| had 100% sequence coverage and 78.06% identity with *Nocard ioides okcheonensis* MMS20-HV4-12, the orf|0|+|5885997-5886810| had 80% sequence coverage and 67.71% identity with *Mycobacterium fortuitum* subsp. *fortuitum* DSM 46621, while orf|0|+|813403-813805| and orf|0|-|5450187-5451333| showed very low coverage and identity with those from the public strains (Supplementary Tables 8, 9, Supplementary Figures 13A–D).

Among 17 novel ORFs, four genes were highly conserved in almost all type strains of 5 genera from the family *Mycobacteriaceae*, including orf|0|+|3841204-3842056|, orf|0|+|1060315-1061149|, orf|0|+|4368726-4369110|, and orf|0|+|5658318-5659182|, implying that these four novel ORFs may be *M. smegmatis* species-specific genes.

## Discussion

Accurate identification of novel peptides in proteogenomic is hindered by inflated databases, whether in genomic reannotation (Mitchell et al., 2018; Martinez et al., 2020; Yu et al., 2021) or mutated peptides in precision medicine research (Gao et al., 2019; Gillette et al., 2020; Cao et al., 2021; Satpathy et al., 2021). Various FDR filtering strategies have been proposed and applied to evaluate the authority of novel peptides (Li et al., 2016, 2017), while there are still many challenges in identifying and verifying novel peptides in large-scale proteogenomic studies (Aggarwal et al., 2022). In this study, we proposed using mirror proteases of Ac-Trypsin and Ac-LysargiNase to create complementary *b*/*y* ion pairs of identified peptides to identify unannotated gene products confidently. Based on these mirror spectrum characteristics, Ac-Trypsin and Ac-LysargiNase provide strong evidence for novel peptides in proteogenomic analysis, which can be applied to all prokaryotic and eukaryotic samples.

As described previously (Huesgen et al., 2015), the median coverages of the *b* and *y* series of ions were only 47–69% in Ac-LysargiNase or Ac-Trypsin digests, whereas the coverage of both *b* and *y* ions from the combination of Ac-LysargiNase and Ac-Trypsin was increased to 75–84%, which provided extensive evidence for accurately and confidently identifying novel peptides in *M. smegmatis* MC^2^ 155. The same strategy can be applied in other proteogenomic studies to accurately and confidently identify unannotated genes missing from the traditional genomics study.

To confirm the accuracy of novel mirror peptides, we performed ^14^N and ^15^N metabolic labeling in cell culture to provide identical light and heavy spectra in one experiment. Results showed that each sequence of novel mirror peptides provided identical ^14^N and ^15^N-labeling spectra with a similarity score of cosin >0.90. To further assess the authenticity of these novel peptides from novel genes or gene events, we used dimethylation labeling technology, peptide synthesis, and public Ribo-seq datasets to check the N-termini of corrected annotated genes or novel ORFs. We reanalyzed public MS datasets for detecting other peptides derived from novel ORFs and compared the expression abundance at the mRNA level. All of these data supported the reliability of these novel events based on our novel mirror peptides.

In a word, the pair of mirror proteases, Ac-Trypsin and Ac-LysargiNase, helped in novel peptide identification and verification, which could be widely used for rapidly and precisely finding unannotated sequence variants in proteogenomic studies.

## Data availability statement

The raw MS/MS data generated in this study were deposited into iProX (https://www.iprox.org/) (Ma et al., 2019) with the identifier IPX0003644000 (Ac-Trypsin), IPX0003886000 (Ac-LysargiNase), and IPX0004773001 (N-terminal enrichment). The datasets presented in this study can be found in online Supplementary materials.

## Author contributions

PX, YZ, and ZL conceived and designed the experiments. YZ performed ^15^N labeling and proteome sample preparation. JS performed an N-terminal enrichment experiment. YL and GW performed MS data detection. SJ performed data analysis with the assistance of ZZ, LC, WW, LY, ED, and LZ. SJ, YZ, and PX wrote the manuscript with the help of all authors. All authors have read and approved the manuscript.

## Funding

This study was supported by the Chinese National Basic Research Programs (2020YFE0202200, 2017YFA0505002, and 2017YFA0505700), the National Science Foundation (31901037, 32141003, 31870824, 32071431, and 32070668), the Beijing-Tianjin-Hebei Basic Research Cooperation Project (J200001), the Innovation Foundation of Medicine (AWS17J008, 20SWAQX34, and 19SWAQ17), the Foundation of State Key Laboratory of Proteomics (SKLP-KY201901), and the CAMS Innovation Fund for Medical Sciences (2019-I2M-5-017).

## Conflict of interest

The authors declare that the research was conducted in the absence of any commercial or financial relationships that could be construed as a potential conflict of interest.

## Publisher's note

All claims expressed in this article are solely those of the authors and do not necessarily represent those of their affiliated organizations, or those of the publisher, the editors and the reviewers. Any product that may be evaluated in this article, or claim that may be made by its manufacturer, is not guaranteed or endorsed by the publisher.
