# Supplementary material for: Mirror proteases of Ac-Trypsin and Ac-LysargiNase precisely improve novel event identifications in Mycolicibacterium smegmatis MC2 155 by proteogenomic analysis
Source: Front Microbiol. 2022 Oct 12;13:1015140. doi: 10.3389/fmicb.2022.1015140 (PMC9597629; doi:10.3389/fmicb.2022.1015140)
Supplement: Supplementary file 1 [file Data_Sheet_1.PDF]

Mirror Proteases of Ac-Trypsin and Ac-LysargiNase Precisely Improve Novel Event Identifications in *Mycolicibacterium smegmatis* MC^2 155 Proteogenomic Analysis

Songhao Jiang[1,2], Jiahui Shi[2], Yanchang Li[2], Zhenpeng Zhang[2], Lei Chang[2], Guibin Wang[2], Wenhui Wu[2,3], Liyan Yu[4], Erhei Dai[5], Lixia Zhang[6], Zhitang Lyu[1*], Ping Xu[1,2,3,4*] and Yao Zhang[2*]

[1] *Key Laboratory of Microbial Diversity Research and Application of Hebei Province, School of Life Sciences, Hebei University, Baoding 071002, China*
[2] *State Key Laboratory of Proteomics, Beijing Proteome Research Center, National Center for Protein Sciences Beijing, Research Unit of Proteomics & Research and Development of New Drug of Chinese Academy of Medical Sciences, Institute of Lifeomics, Beijing 102206, China*
[3] *Guangzhou University of Chinese Medicine, Second Clinical Medicine Collage, Guangzhou Higher Education Mega Center, Guangzhou 510006, China*
[4] *Institute of Medicinal Biotechnology, Research Unit of Proteomics & Research and Development of New Drug, Chinese Academy of Medical Sciences and Peking Union Medical College, Beijing 100050, China*
[5] *The Fifth Hospital of Shijiazhuang, School of Public Health, Shijiazhuang 050024, China*
[6] *Key Research Laboratory for Infectious Disease Prevention for State Administration of Traditional Chinese Medicine, Tianjin Institute of Respiratory Diseases, Haihe Hospital, Tianjin University, Tianjin 300350, China*

[*] Yao Zhang, Ping Xu, and Zhitang Lyu were corresponding authors.

Supplementary Figure Legends

Supplementary Figure 1 | Separation of total cell proteins of *M. smegmatis* MC^2 155 using 10% SDS-PAGE.

Supplementary Figure 2 | Specificity evaluation of Ac-Trypsin and Ac-LysargiNase.

Supplementary Figure 3 | Advantage of digestion of mirror proteases based on the annotated protein WP_011728341.1. **(A)**, Identification of peptides digested by Ac-Trypsin and Ac-LysargiNase. **(B)**, Spectra of mirror peptides derived from Ac-Trypsin and Ac-LysargiNase.

Supplementary Figure 4 | Comparison of *b* and *y* ion coverages at different position of identified peptide by single protease and combined proteases.

Supplementary Figure 5 | Identification of novel peptides from Ac-Trypsin and Ac-LysargiNase digests. **(A)**, Classification of C- and N-terminal peptides digested by Ac-Trypsin and Ac-LysargiNase. **(B)**, Identification of peptides with single protease and mirror proteases evidence from Ac-Trypsin and Ac-LysargiNase datasets. **(C)**, Detail information of truncated sequences from Ac-Trypsin and Ac-LysargiNase digestion.

Supplementary Figure 6 | The filtering process of novel peptides of *M. smegmatis* MC^2 155. **(A)**, Classification of novel peptides from two events. The filtering process of novel peptides from N-terminal correction **(B, D)** and novel ORFs **(C, E)**.

Supplementary Figure 7 | N-termini correction of 6 recorded proteins. Spectra of novel peptides from Ac-Trypsin **(A)** and Ac-LysargiNase **(B)** digests. **(C)**, The comparative genomics of the N-termini correction based on NCBI public strains. **(D)**, The functional description of the stop-to-stop translational protein sequences. **(E)**, The sequenced reads mapping on the N-terminal extension regions based on public Ribo-seq dataset (Shell et al., 2015). **(F)**, Verification of the selected novel peptides by peptide synthesis.

Supplementary Figure 8 | Transcriptome depth of 6 N-termini corrected genes based on public RNA-seq dataset (SRR17866681).

Supplementary Figure 9 | Verification of 17 novel ORFs. Spectra of novel peptides from Ac-Trypsin **(A)** and Ac-LysargiNase **(B)** digests. BLASTP **(C)** and BLASTN **(D)** result of sequence alignment of novel ORFs against NCBI nr database and the maximum likelihood tree of proteins aligned by BLASTP. **(E),** Transcriptome depth of 17 novel ORFs based on public Ribo-seq dataset (Shell et al., 2015). **(F)**, Verification of the selected novel peptides by peptide synthesis.

Supplementary Figure Legends

Supplementary Figure 10 | Spectra of other tryptic peptides of novel ORFs from public proteome of *M. smegmatis* MC^2 155 (Potgieter et al., 2016; Giddey et al., 2017).

Supplementary Figure 11 | Identification of novel ORF orf|0|+|813403-813805|. **(A)**, The peptides identified from our and public datasets. Spectra of novel peptides from our Ac-Trypsin **(B)** and Ac-LysargiNase **(C)** datasets. **(D)**, The spectra of N-termini labeled peptide with dimethyl modification from our N-terminomic dataset.

Supplementary Figure 12 | Transcriptome depth of 17 novel ORFs based on public RNA-seq dataset (SRR17866681).

Supplementary Figure 13 | The homology and conservation analysis of 17 novel ORFs in *Mycobacteriaceae* **(A)**, *Mycolicibacterium* **(B)**, *Mycobacterium* **(C)**, *Mycolicibacillus*, *Mycobacteroides*, and *Mycolicibacter* **(D)**.

Supplementary Figure 1

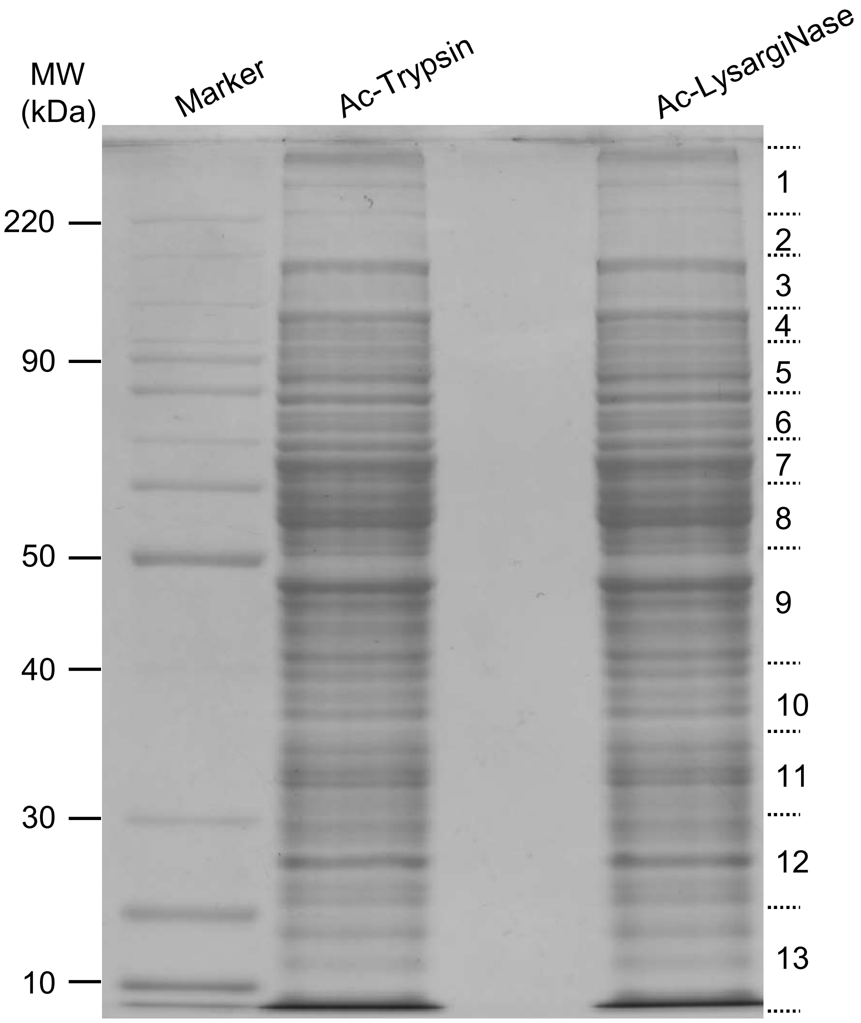

Supplementary Figure 2

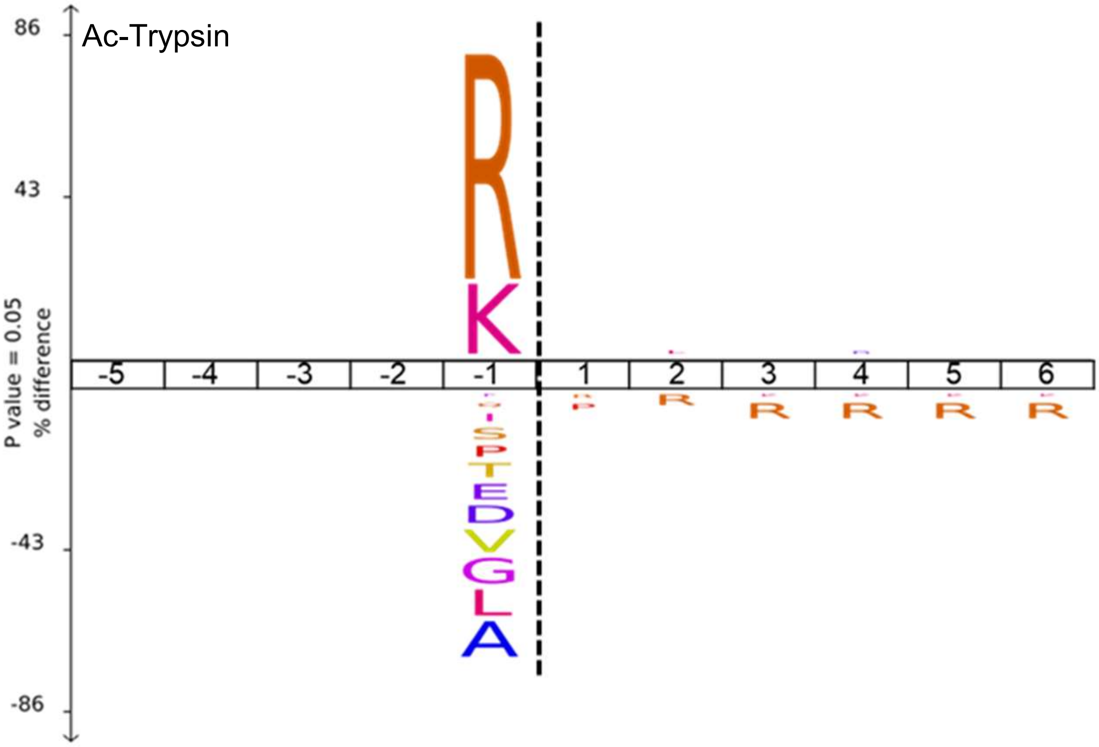

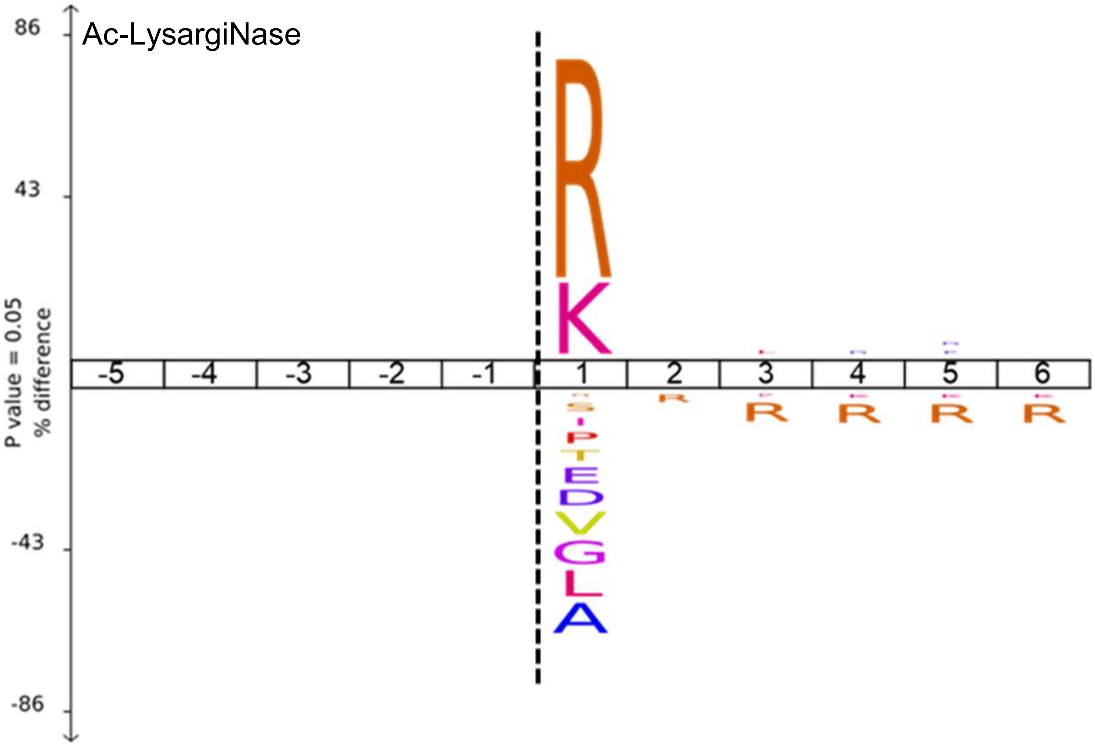

Supplementary Figure 3

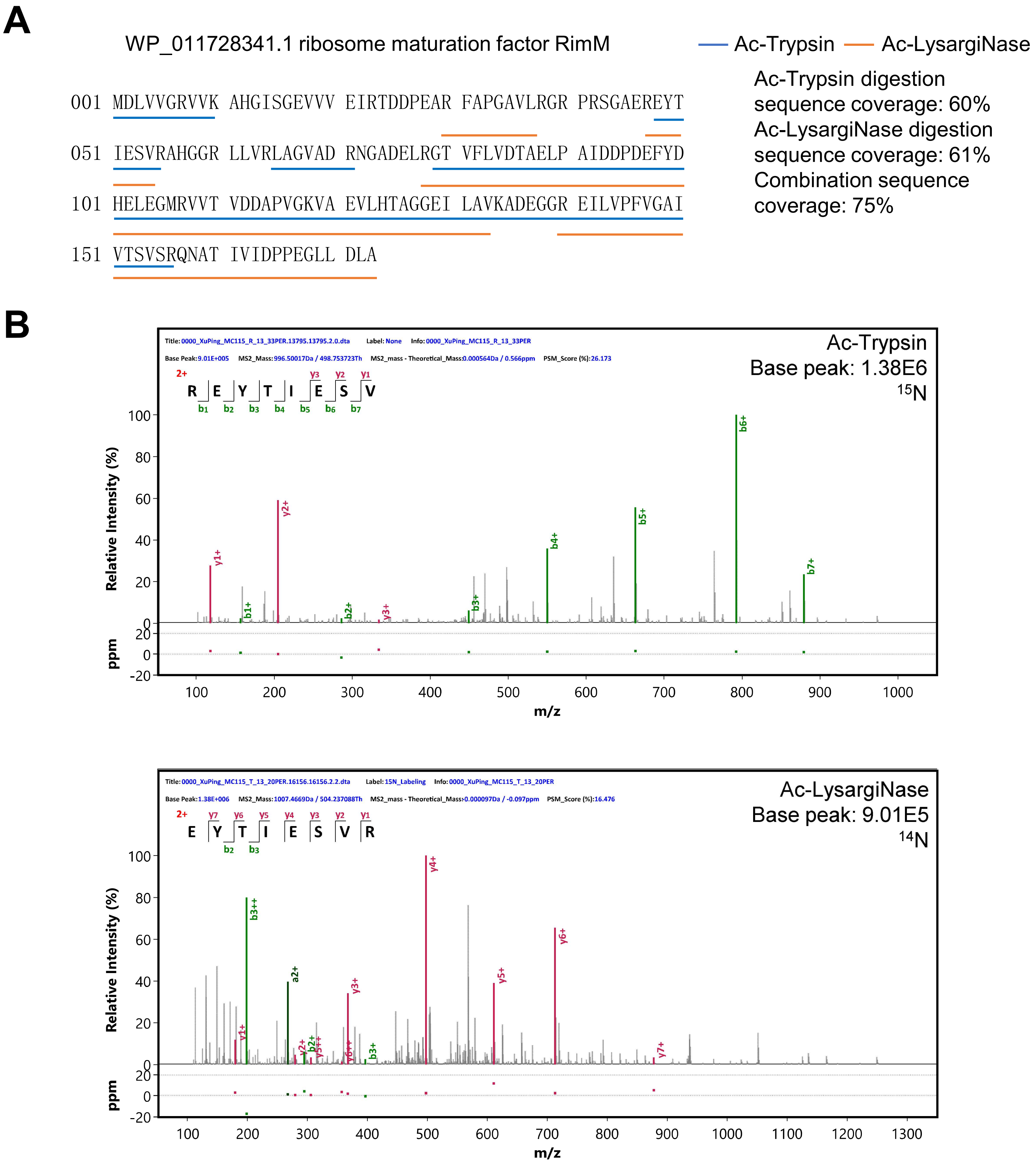

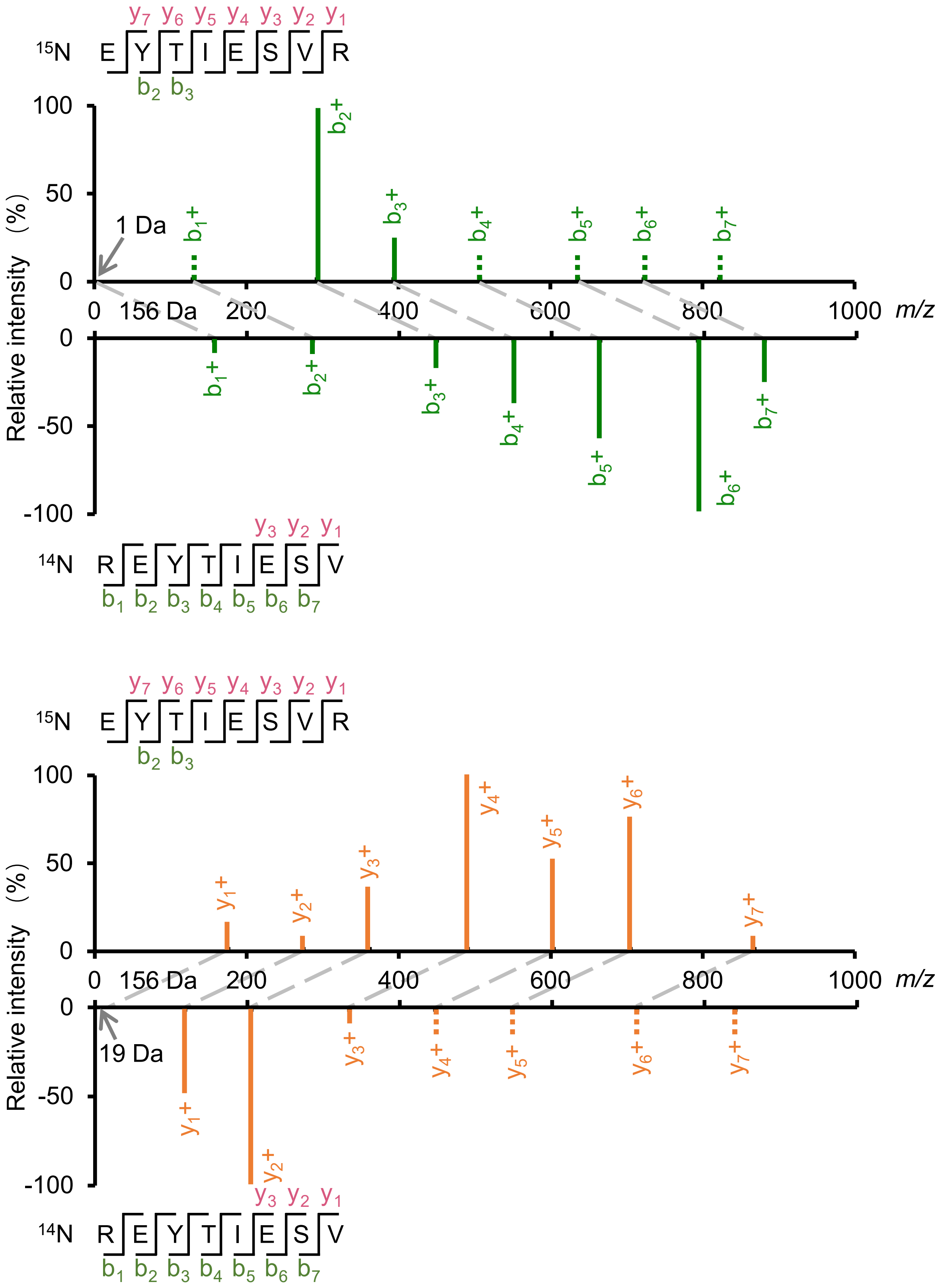

15N
E Y T I E S V R
y7 y6 y5 y4 y3 y2 y1
b2 b3
14N
R E Y T I E S V
y3 y2 y1
b1 b2 b3 b4 b5 b6 b7
Relative intensity (%)
100
50
0
-50
-100
0
200
400
600
800
1000
m/z
1 Da
156 Da
19 Da
b1+
b2+
b3+
b4+
b5+
b6+
b7+
y1+
y2+
y3+
y4+
y5+
y6+
y7+

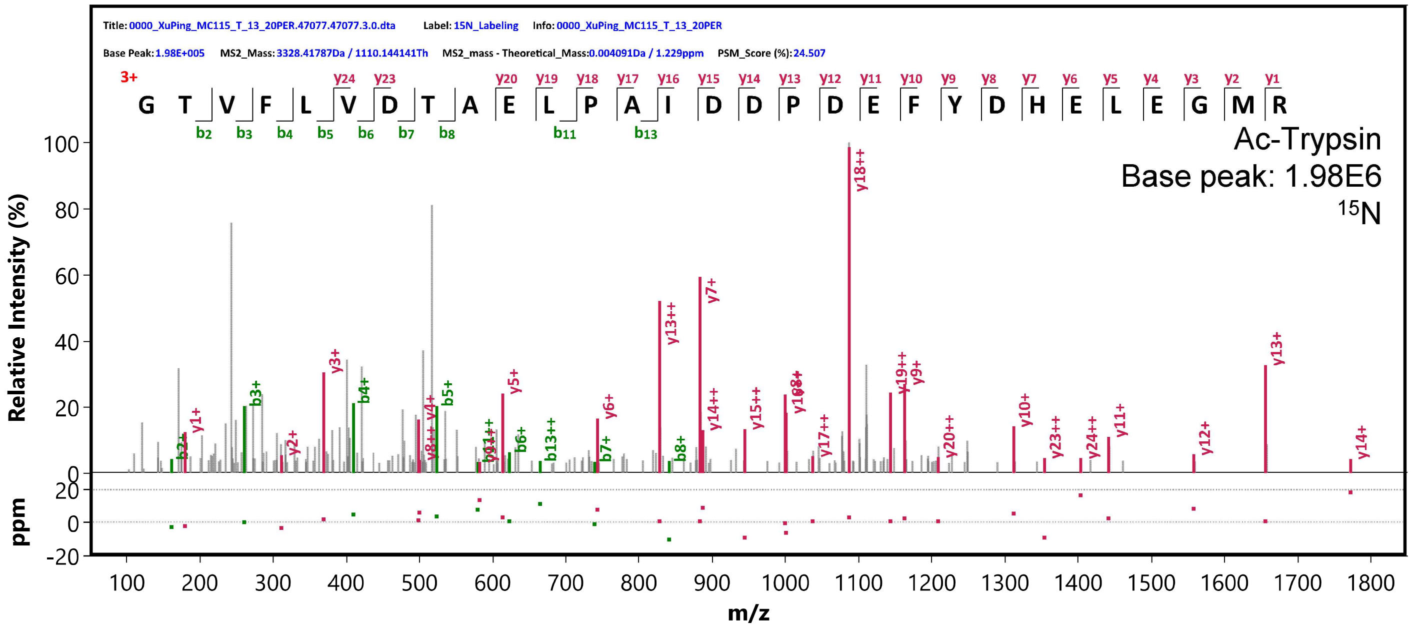
Title: 0000_XuPing_MC115_T_13_20PER.47077.47077.3.0.dta Label: 15N_Labeling Info: 0000_XuPing_MC115_T_13_20PER
Base Peak: 1.98E+005 MS2_Mass: 3328.41787Da / 1110.144141Th MS2_mass - Theoretical_Mass: 0.004091Da / 1.229ppm PSM_Score (%): 24.507
3+
G T V F L V D T A E L P A I D D P D E F Y D H E L E G M R
Ac-Trypsin
Base peak: 1.98E6
15N
Relative Intensity (%)
ppm
m/z

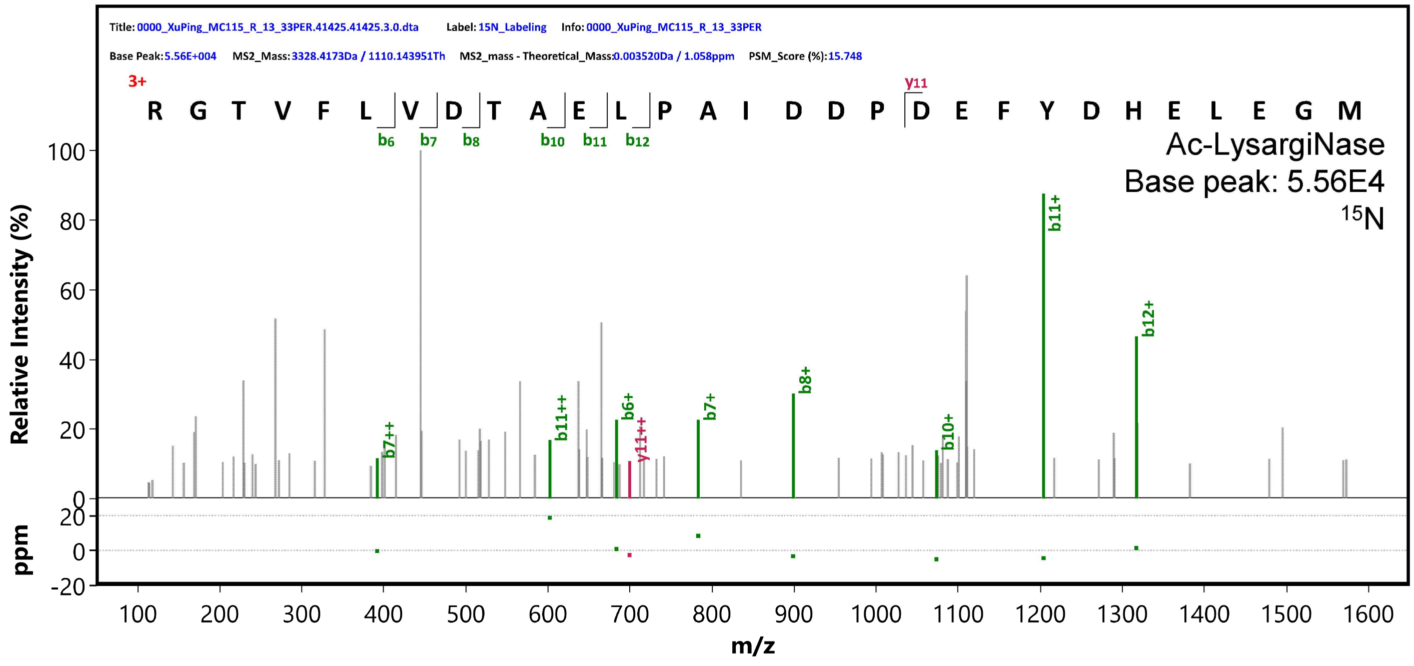
Title: 0000_XuPing_MC115_R_13_33PER.41425.41425.3.0.dta Label: 15N_Labeling Info: 0000_XuPing_MC115_R_13_33PER
Base Peak: 5.56E+004 MS2_Mass: 3328.4173Da / 1110.143951Th MS2_mass - Theoretical_Mass: 0.003520Da / 1.058ppm PSM_Score (%): 15.748
3+
R G T V F L V D T A E L P A I D D P D E F Y D H E L E G M
Ac-LysargiNase
Base peak: 5.56E4
15N
Relative Intensity (%)
ppm
m/z

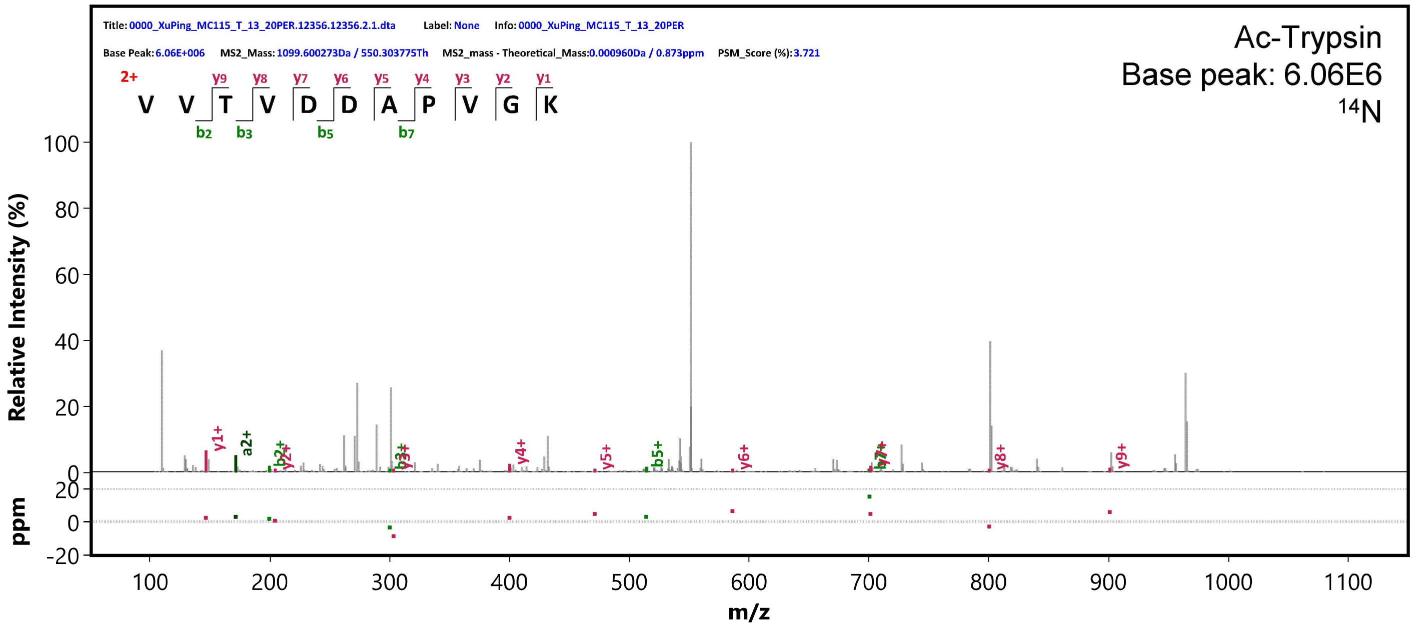
Title: 0000_XuPing_MC115_T_13_20PER.12356.12356.2.1.dta Label: None Info: 0000_XuPing_MC115_T_13_20PER
Base Peak: 6.06E+006 MS2_Mass: 1099.600273Da / 550.303775Th MS2_mass - Theoretical_Mass: 0.000960Da / 0.873ppm PSM_Score (%): 3.721
Ac-Trypsin
Base peak: 6.06E6
14N
2+
V V T V D D A P V G K
Relative Intensity (%)
ppm
m/z

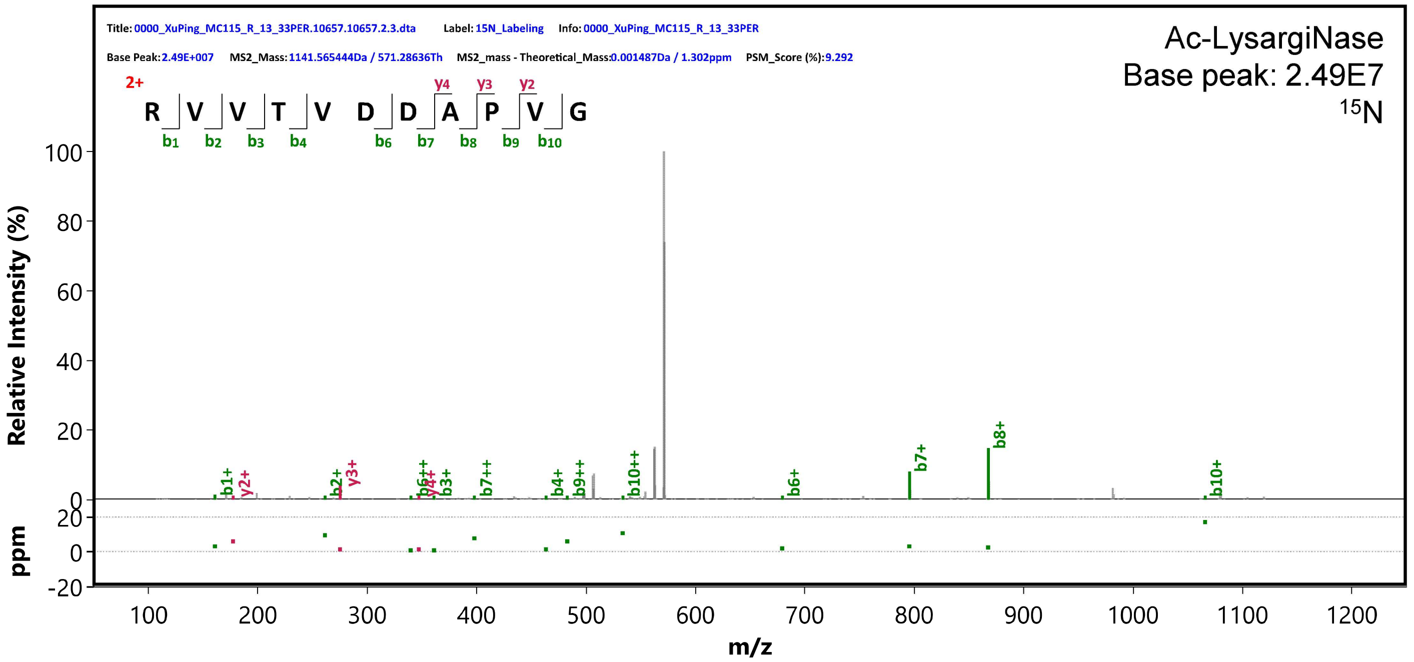
Title: 0000_XuPing_MC115_R_13_33PER.10657.10657.2.3.dta Label: 15N_Labeling Info: 0000_XuPing_MC115_R_13_33PER
Base Peak: 2.49E+007 MS2_Mass: 1141.565444Da / 571.28636Th MS2_mass - Theoretical_Mass: 0.001487Da / 1.302ppm PSM_Score (%): 9.292
Ac-LysargiNase
Base peak: 2.49E7
15N
2+
R V V T V D D A P V G
Relative Intensity (%)
ppm
m/z

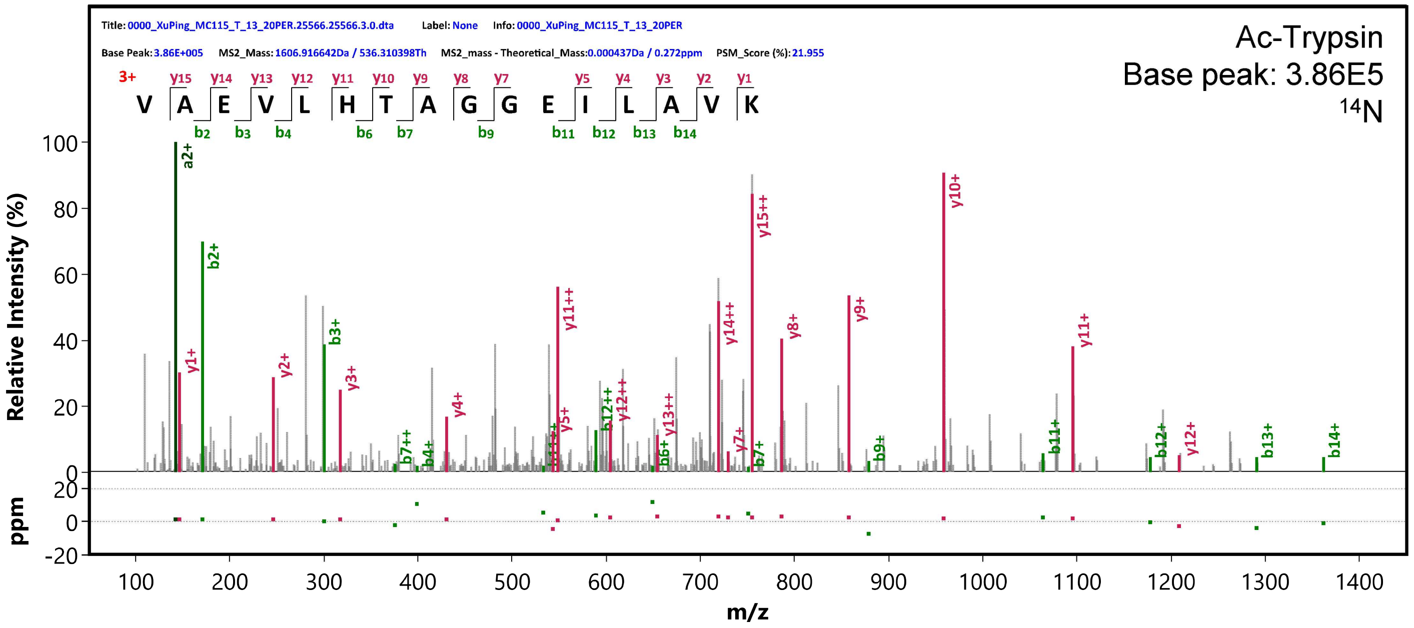
Title: 0000_XuPing_MC115_T_13_20PER.25566.25566.3.0.dta Label: None Info: 0000_XuPing_MC115_T_13_20PER
Base Peak: 3.86E+005 MS2_Mass: 1606.916642Da / 536.310398Th MS2_mass - Theoretical_Mass: 0.000437Da / 0.272ppm PSM_Score (%): 21.955
Ac-Trypsin
Base peak: 3.86E5
14N
3+
V A E V L H T A G G E I L A V K
Relative Intensity (%)
ppm
m/z
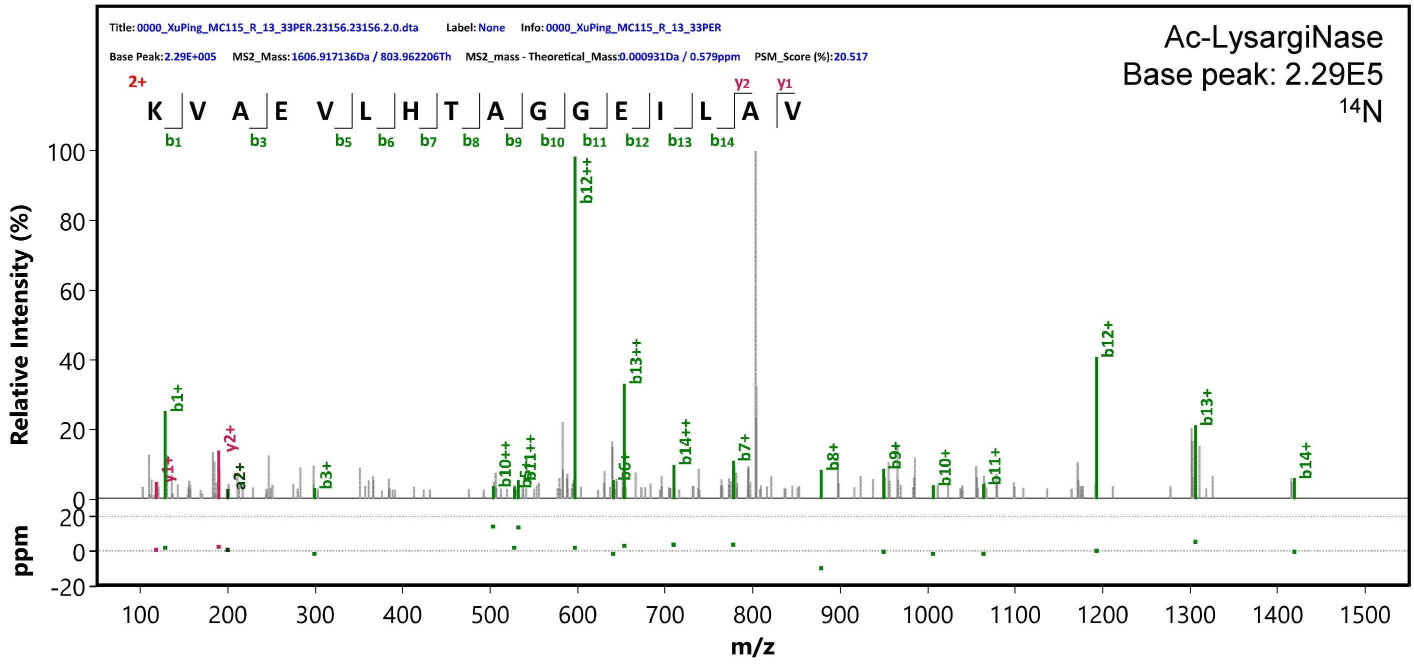
Title: 0000_XuPing_MC115_R_13_33PER.23156.23156.2.0.dta Label: None Info: 0000_XuPing_MC115_R_13_33PER
Base Peak: 2.29E+005 MS2_Mass: 1606.917136Da / 803.962206Th MS2_mass - Theoretical_Mass: 0.000931Da / 0.579ppm PSM_Score (%): 20.517
Ac-LysargiNase
Base peak: 2.29E5
14N
2+
K V A E V L H T A G G E I L A V
Relative Intensity (%)
ppm
m/z

Supplementary Figure 4

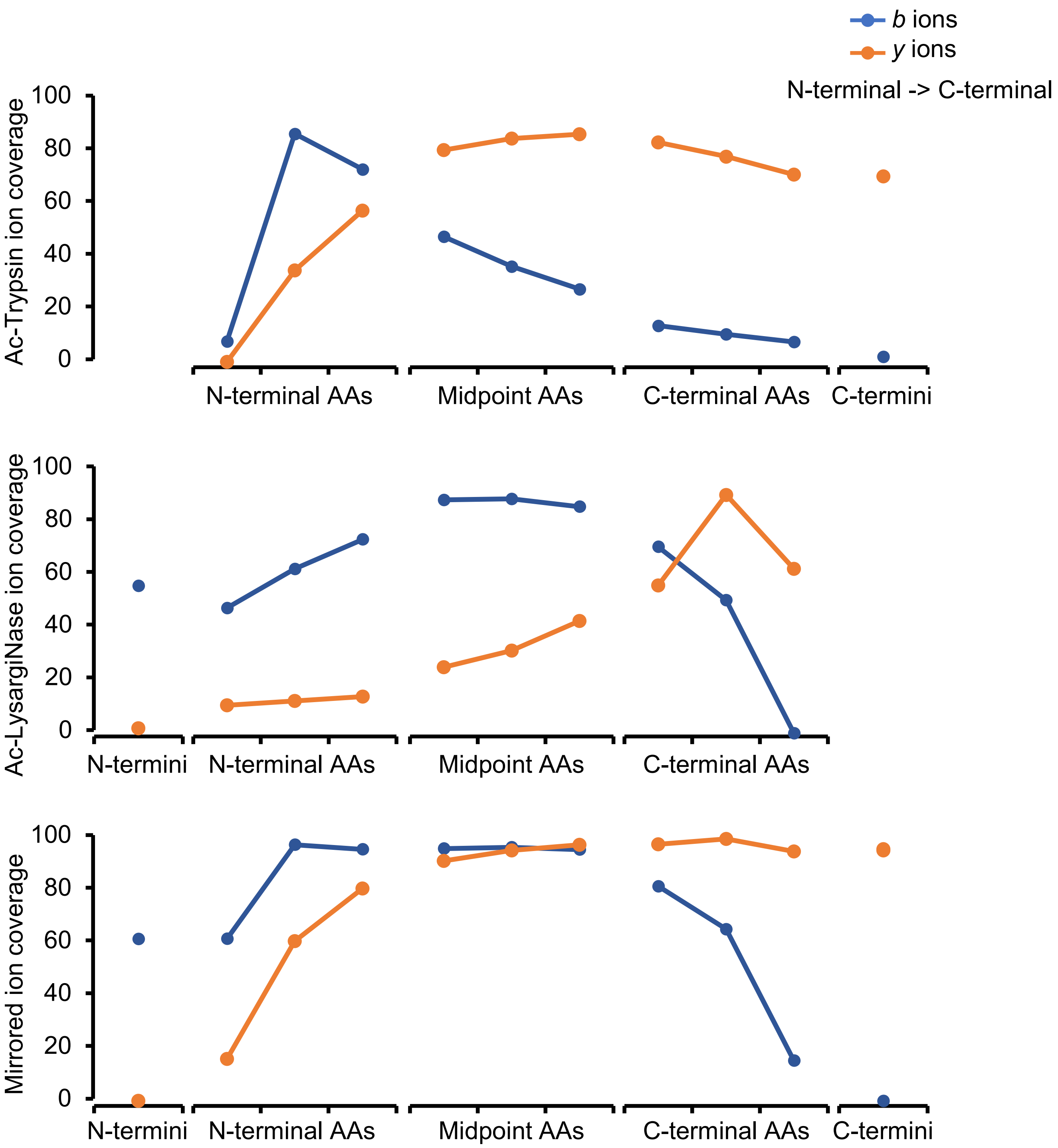

Supplementary Figure 5

A

Classification of C- and N-terminal peptides digested by Ac-Trypsin Ac-LysargiNase.

Classification	Ac-Trypsin		Ac-LysargiNase	
	Type	No.	Type	No.
Cleavage residue on the K/R remain side	...A	6	A...	65
	...AK/R	808	K/RA...	441
	...AKK/RR/KR/RK	31	KK/RR/KR/RKA...	15
Cleavage residue on the K/R rejection side	K/RA...	47	...AK/R	11
	A...	798	...A	510
Sum		845		521

B

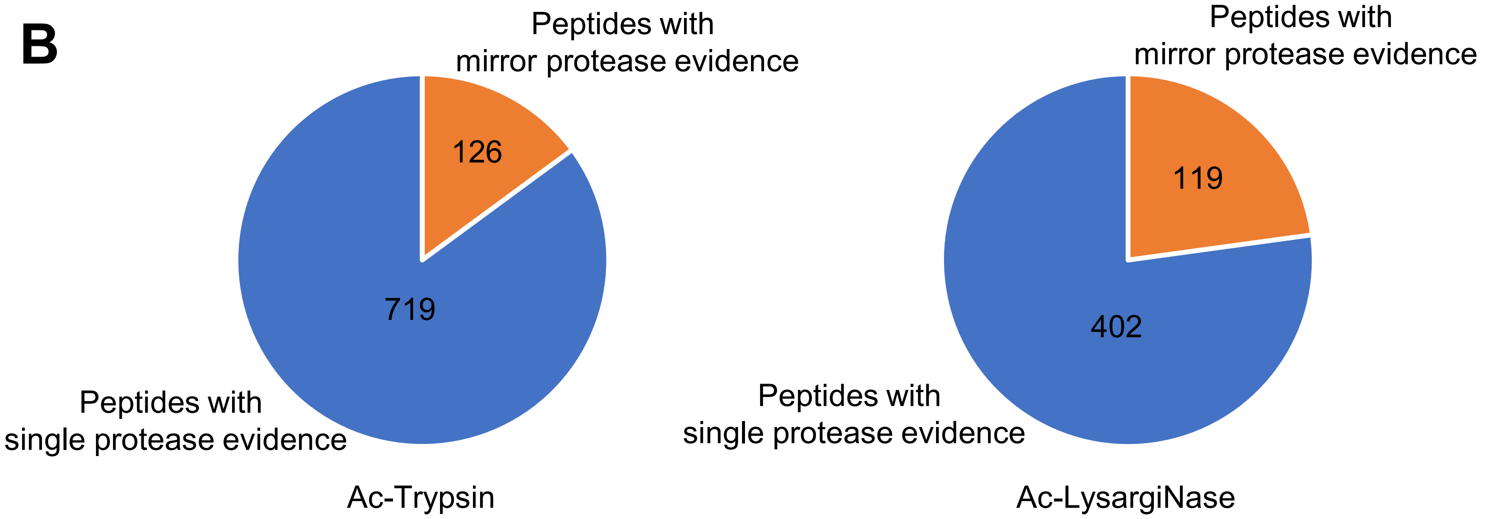

C

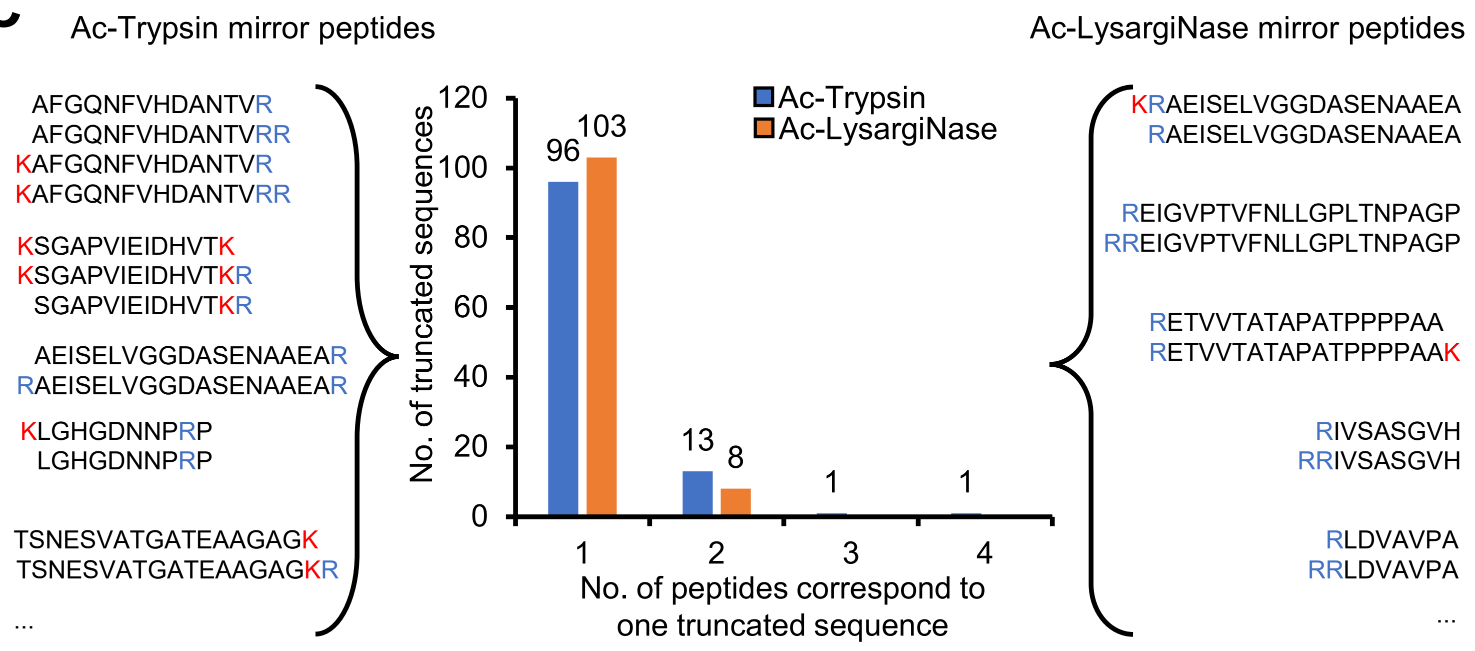

Supplementary Figure 6

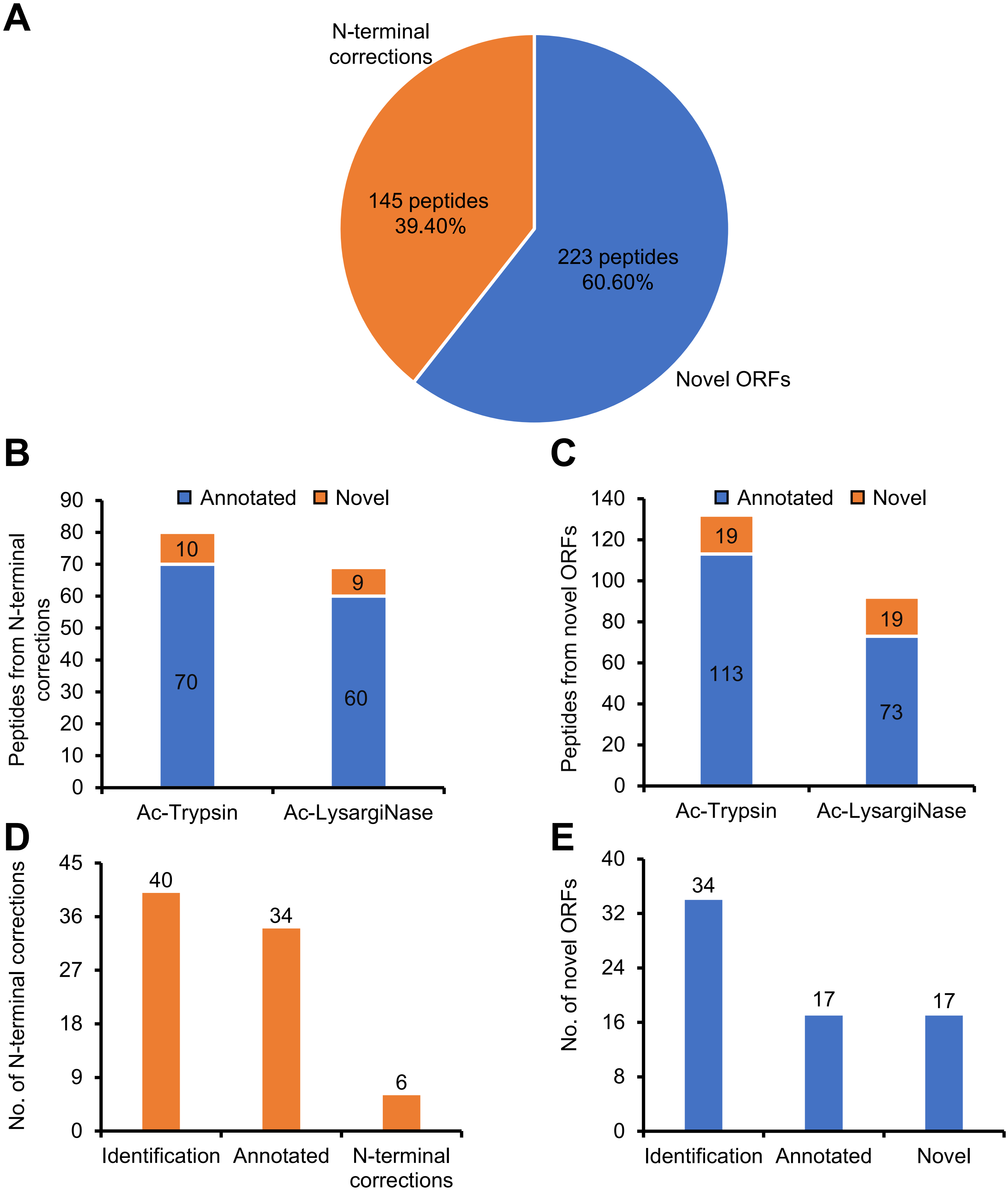

Supplementary Figure 7

No. 1 orf|0|-|626711-627743|

A

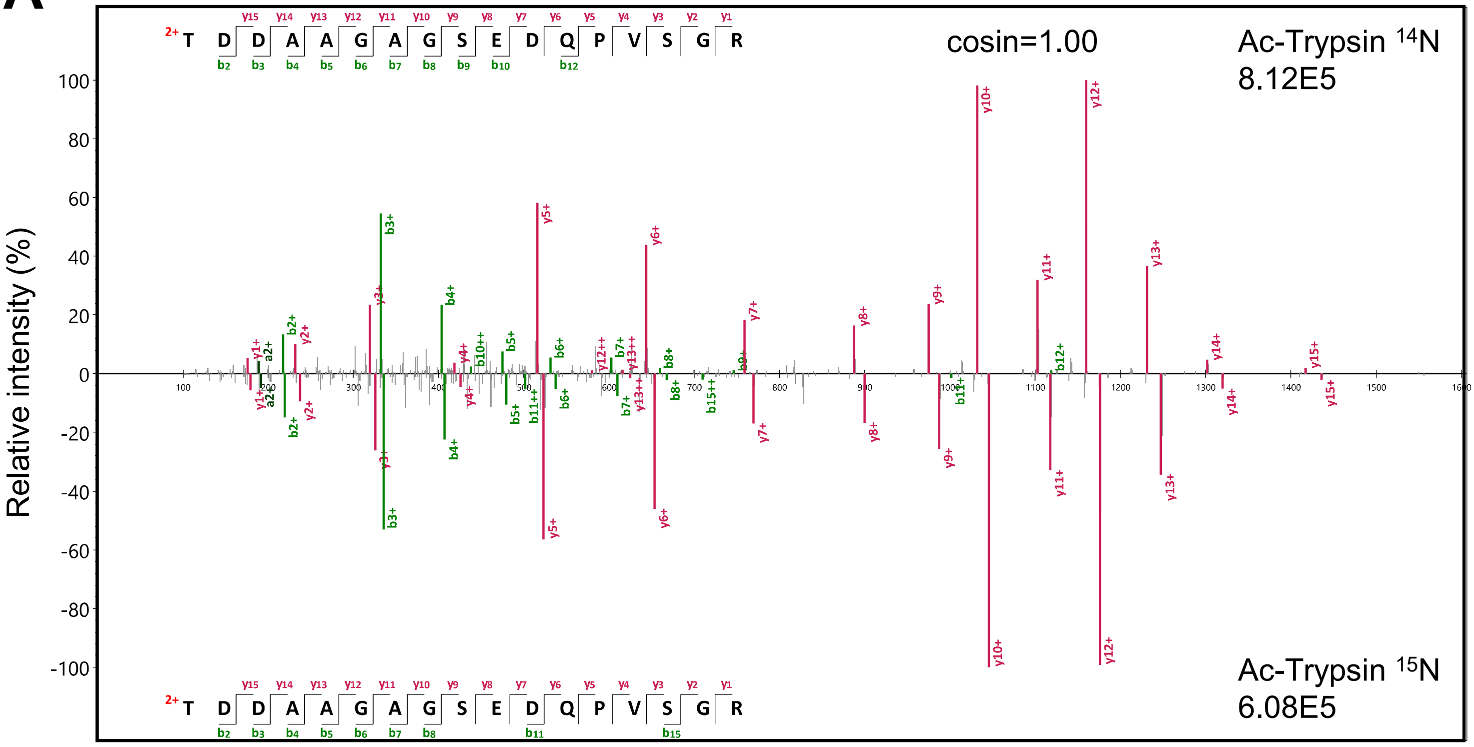

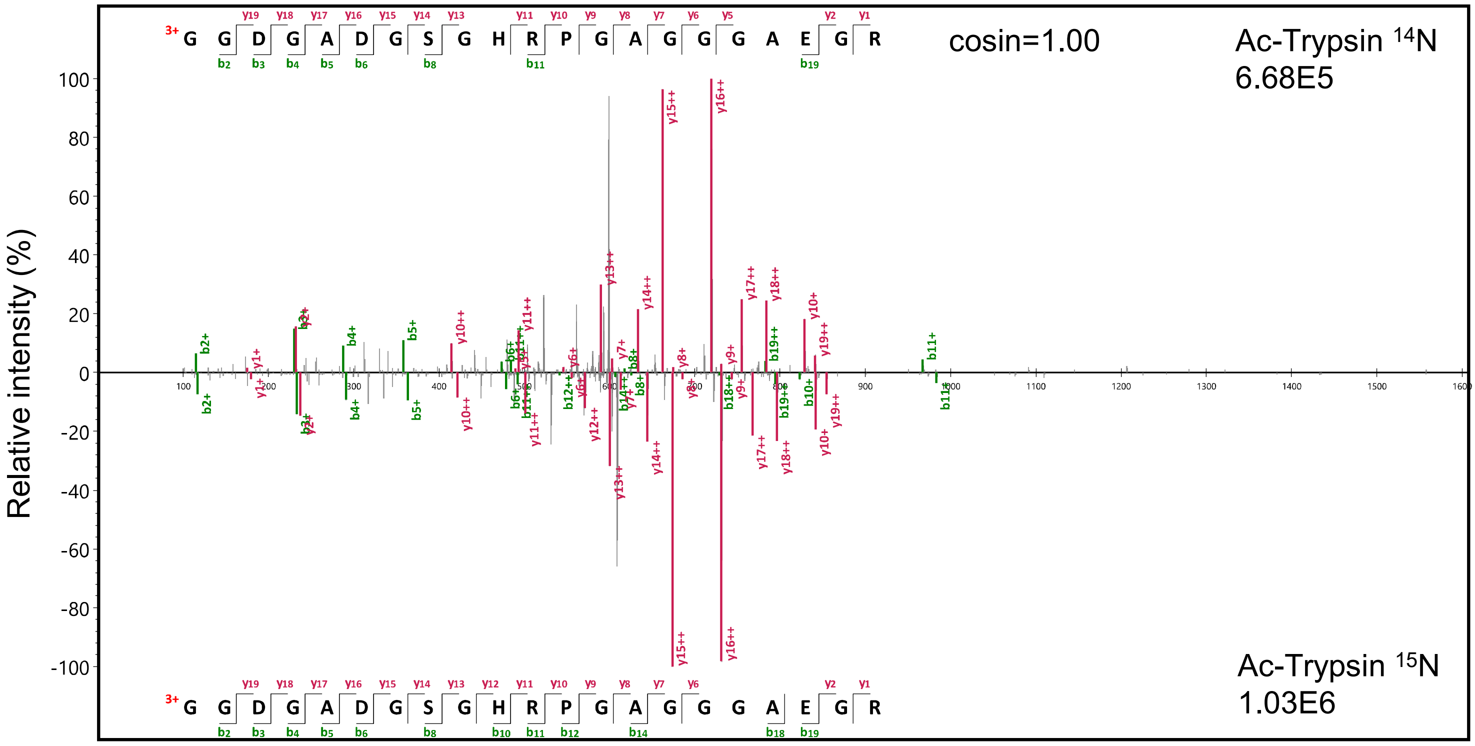

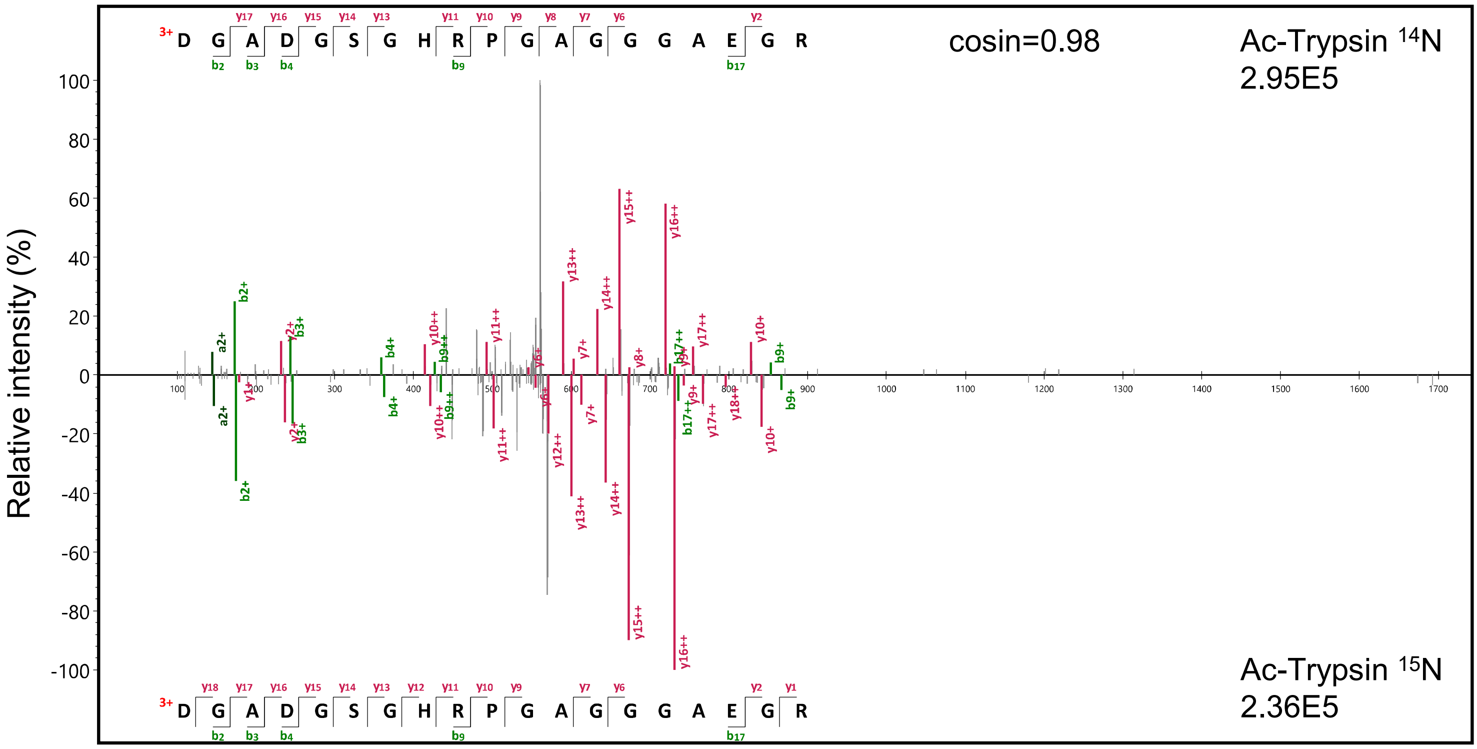
3+ D G A D G S G H R P G A G G G A E G R
cosin=0.98
Ac-Trypsin 14N
2.95E5
Ac-Trypsin 15N
2.36E5
Relative intensity (%)

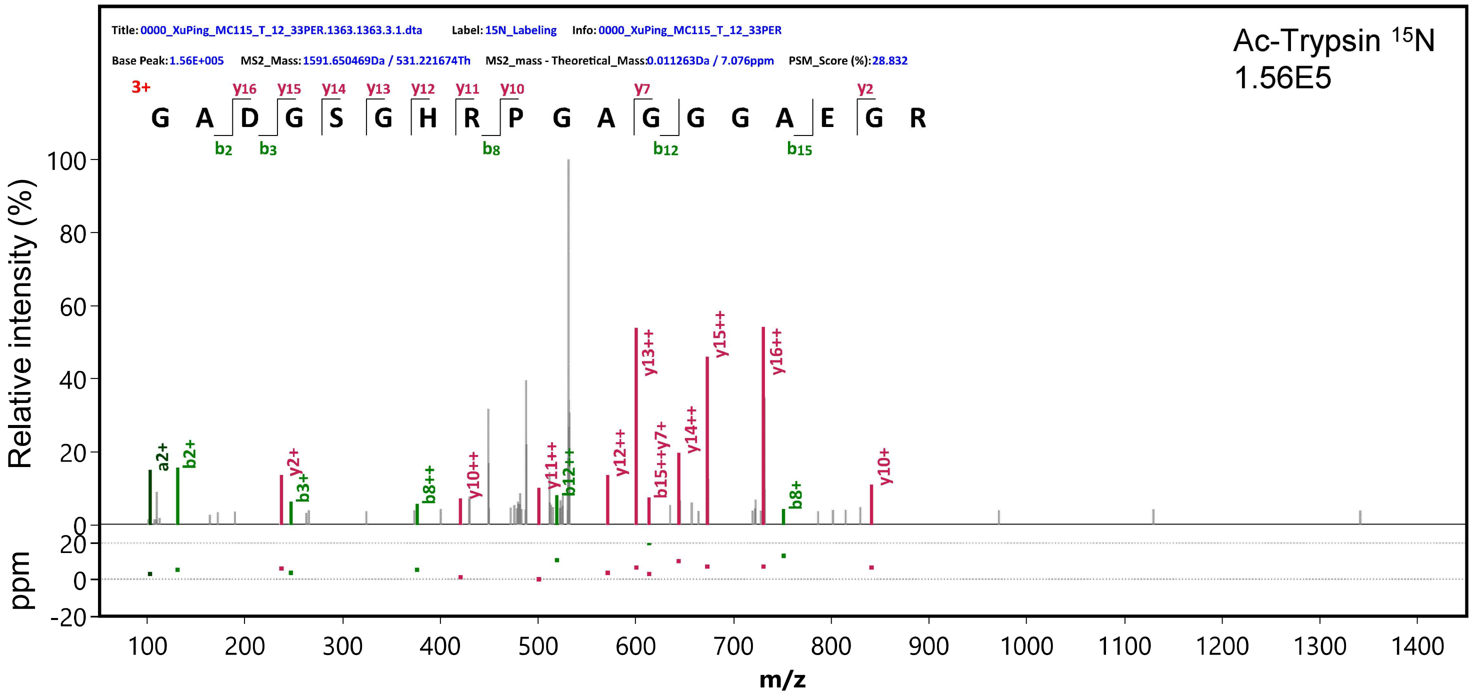
Title: 0000_XuPing_MC115_T_12_33PER.1363.1363.3.1.dta
Label: 15N_Labeling
Info: 0000_XuPing_MC115_T_12_33PER
Base Peak: 1.56E+005
MS2_Mass: 1591.650469Da / 531.221674Th
MS2_mass - Theoretical_Mass: 0.011263Da / 7.076ppm
PSM_Score (%): 28.832
Ac-Trypsin 15N
1.56E5
3+ G A D G S G H R P G A G G G A E G R
Relative intensity (%)
ppm
m/z

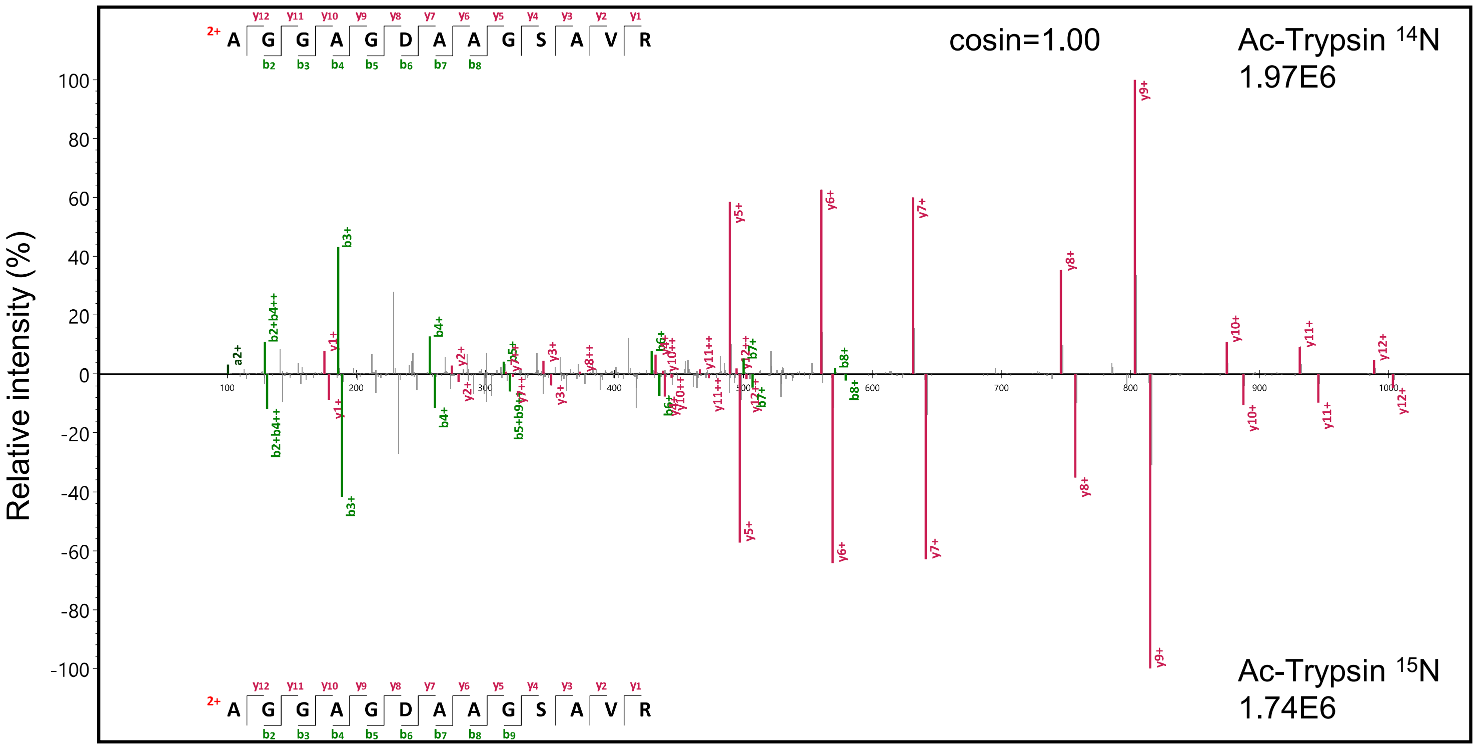

cosin=1.00
Ac-Trypsin ^{14}N
1.97E6
Ac-Trypsin ^{15}N
1.74E6
2+ A G G A G D A A G S A V R
Relative intensity (%)

B

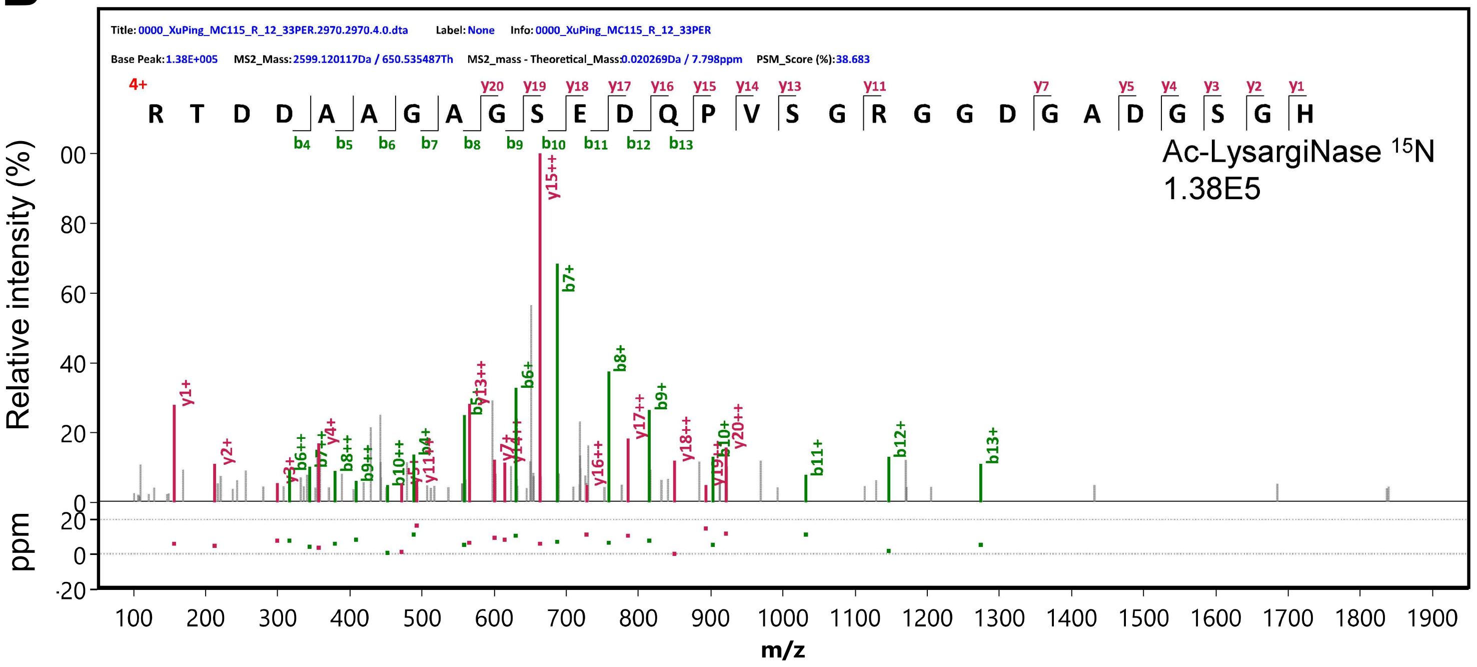

Title: 0000_XuPing_MC115_R_12_33PER.2970.2970.4.0.dta Label: None Info: 0000_XuPing_MC115_R_12_33PER
Base Peak: 1.38E+005 MS2_Mass: 2599.120117Da / 650.535487Th MS2_mass - Theoretical_Mass: 0.020269Da / 7.798ppm PSM_Score (%): 38.683
4+ R T D D A A G A G S E D Q P V S G R G G D G A D G S G H
Ac-LysargiNase ^{15}N
1.38E5
Relative intensity (%)
ppm
m/z

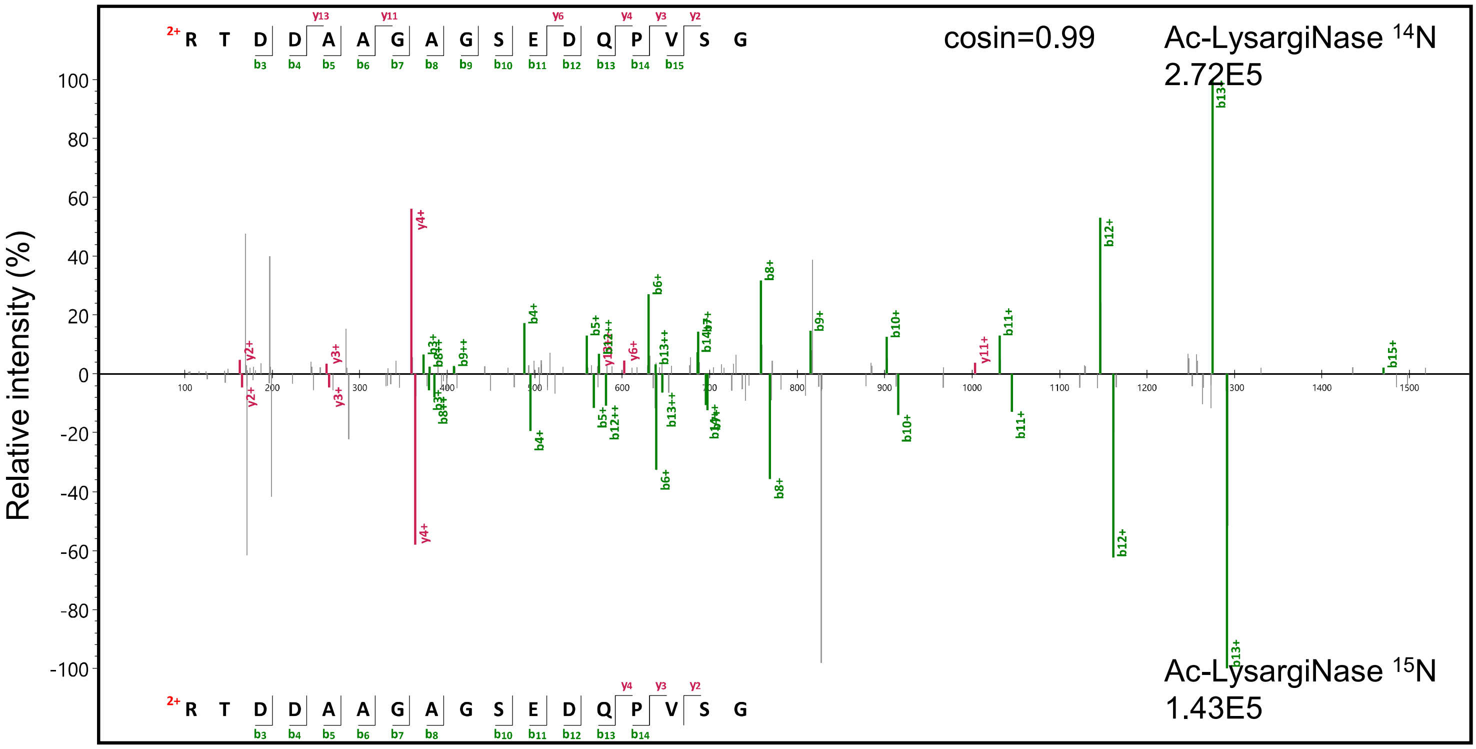

R T D D A A G A G S E D Q P V S G
cosin=0.99
Ac-LysargiNase 14N
2.72E5
Relative intensity (%)
Ac-LysargiNase 15N
1.43E5
R T D D A A G A G S E D Q P V S G

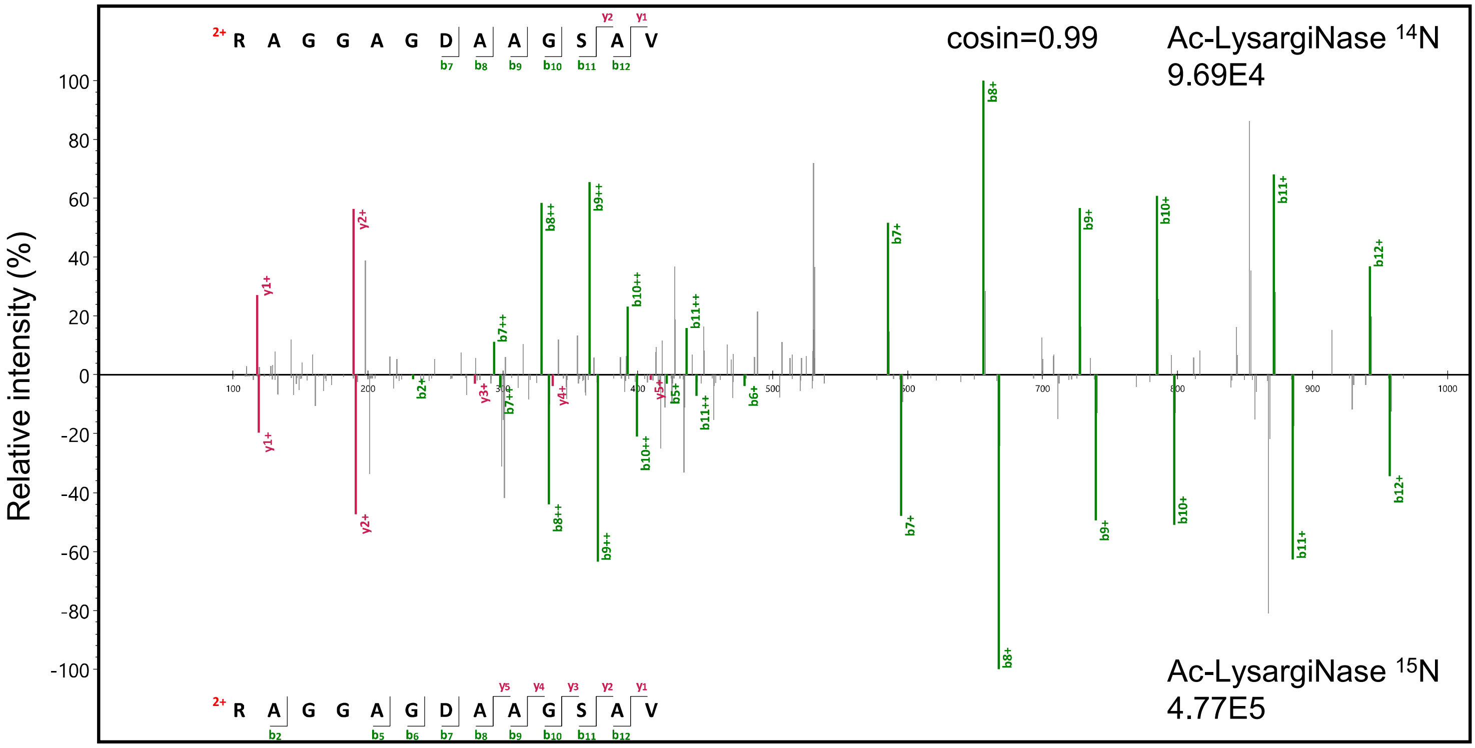

R A G G A G D A A G S A V
cosin=0.99
Ac-LysargiNase 14N
9.69E4
Relative intensity (%)
Ac-LysargiNase 15N
4.77E5
R A G G A G D A A G S A V

C

Ac-Trypsin
Ac-LysargiNase

```
orf|0|-|626711-627743|                    GGPHMGAGAPSRTDDAAGAGSEDQPVSGRG
                     GDGADGSGHRPGAGGGAEGRAGEDRSDRGGRRRYQIRAGRAG
                    GAGDAAGSAVRAERGLKIRAALLNKLSLFVVGLIVPVALLVIWH------
M. smegmatis (WP_011727031.1)                      MGLIVPVALLVIWH------
M. smegmatis (CKG96993.1)                         MVGLIVPVALLVIWH------
M. smegmatis (WP_058127509.1)                     MVGLIVPVALLVIWH------
M. smegmatis (WP_228095910.1)                        MIVPVALLVIWH------
```

D

	Description	Scientific Name	Max Score	Total Score	Query Cover	E value	Per. Ident	Acc. Len	Accession
☑	ABC transporter permease [Mycolicibacterium smegmatis]	Mycolicibacteriu...	392	392	70%	2e-134	99.59%	242	WP_011727031.1
☑	binding-protein-dependent transport system inner membrane protein [Mycolicibacterium smegmatis]	Mycolicibacteriu...	392	392	70%	3e-134	99.18%	243	CKG96993.1
☑	ABC transporter permease [Mycolicibacterium smegmatis]	Mycolicibacteriu...	390	390	70%	1e-133	99.17%	242	WP_058127509.1
☑	ABC transporter permease [Mycolicibacterium smegmatis]	Mycolicibacteriu...	390	390	69%	2e-133	99.58%	240	WP_228095910.1
☑	ABC transporter permease [Mycobacterium goodii]	Mycobacterium ...	377	377	70%	3e-128	95.45%	242	AKS30987.1
☑	ABC transporter permease [Mycobacterium goodii]	Mycobacterium ...	374	374	70%	6e-127	94.21%	242	MBU8808150.1
☑	ABC transporter permease [Mycobacterium goodii]	Mycobacterium ...	373	373	70%	1e-126	94.21%	242	PJK21005.1
☑	ABC transporter permease [Mycobacterium goodii]	Mycobacterium ...	372	372	70%	2e-126	93.80%	242	MBU8818534.1
☑	unnamed protein product [Mycobacterium goodii]	Mycobacterium ...	370	370	69%	1e-125	94.17%	240	WP_240174061.1
☑	ABC transporter permease [Mycobacterium diernhoferi]	Mycobacterium ...	331	331	70%	5e-110	78.60%	247	WP_234800217.1

F

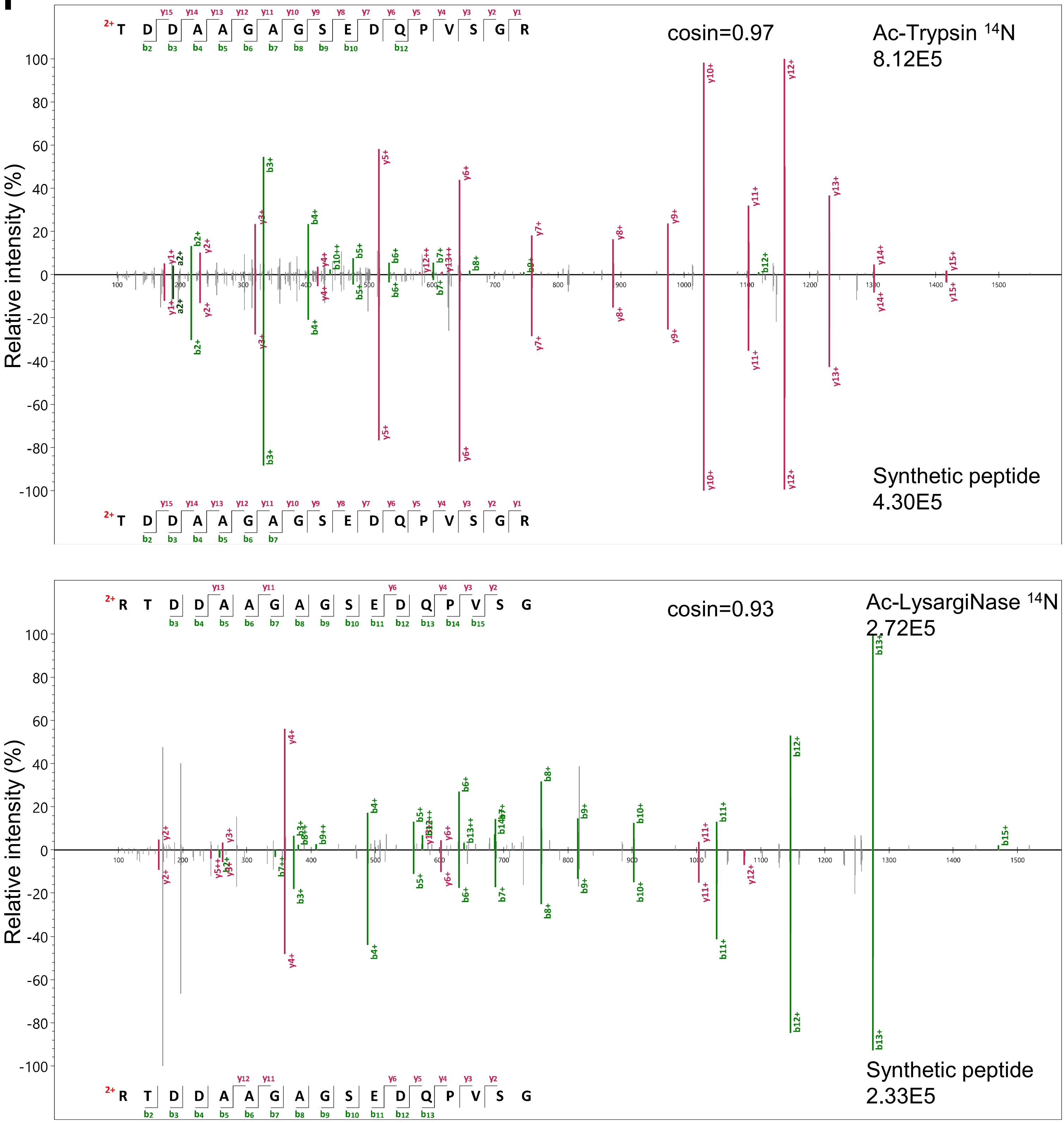

2+ T D D A A G A G S E D Q P V S G R
cosin=0.97
Ac-Trypsin ^{14}N
8.12E5
Synthetic peptide
4.30E5
Relative intensity (%)
2+ R T D D A A G A G S E D Q P V S G
cosin=0.93
Ac-LysargiNase ^{14}N
2.72E5
Synthetic peptide
2.33E5
Relative intensity (%)

No. 2 orf|0|-|1745617-1747033|

A

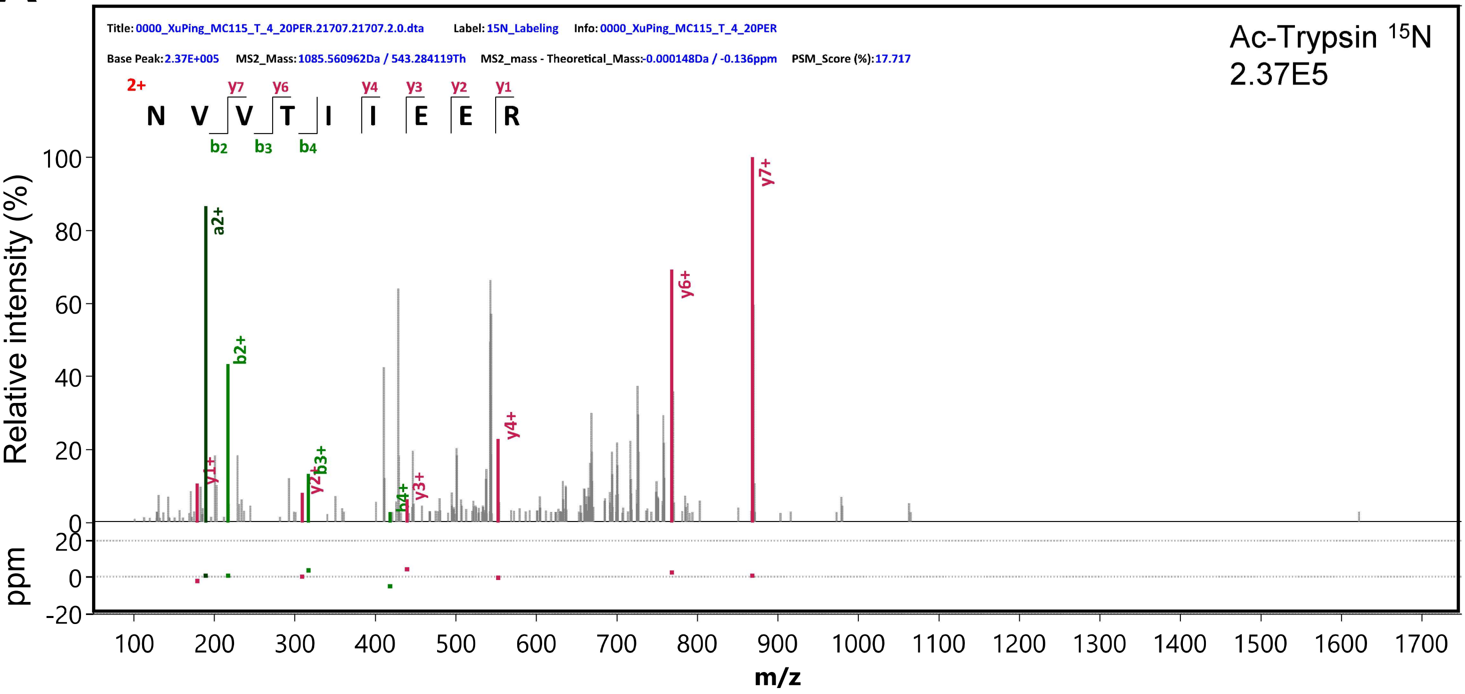

B

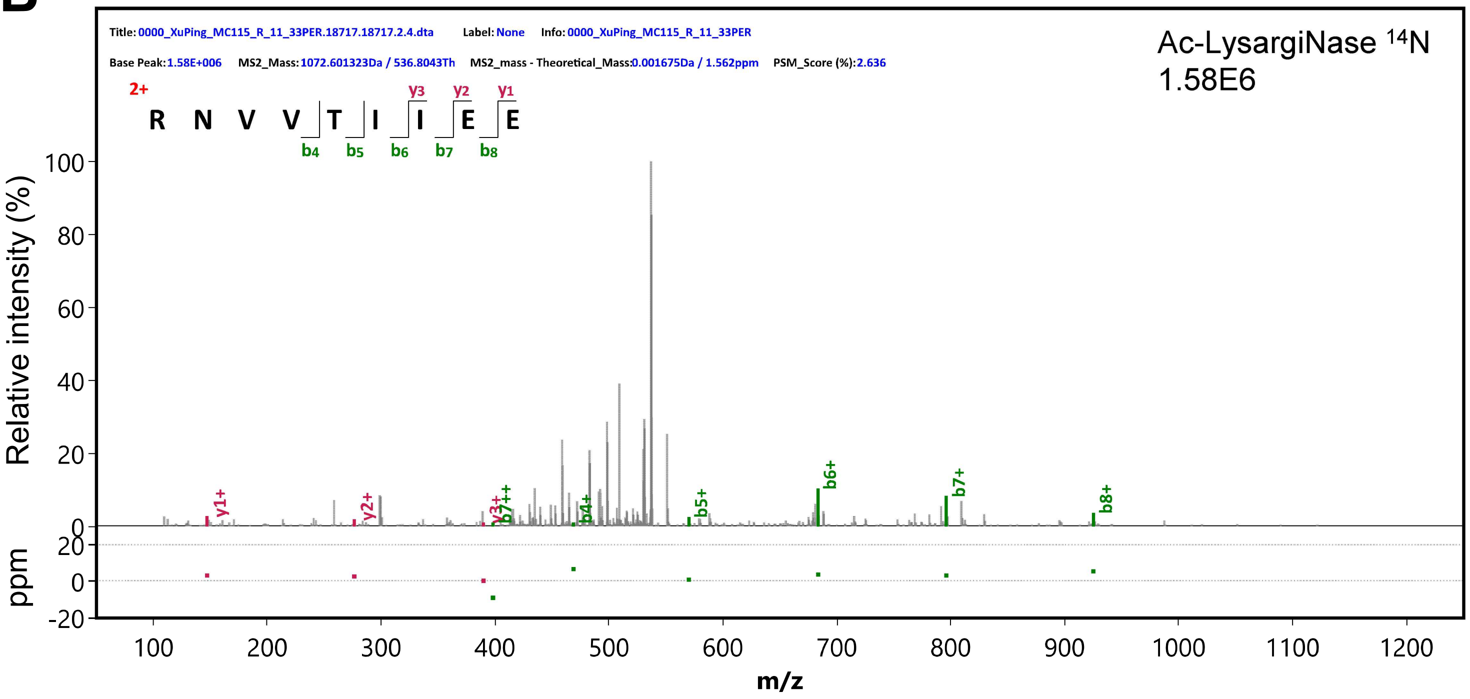

C

Ac-Trypsin
Ac-LysargiNase

orf|0|-|1745617-1747033|	GLRERADAAQSRRQHRRRAVAGDPPGVDD
	APAAEPRGAAVDGCHAGPGAPGGRPRRHRRHHRRPGAGVRRRA
	PVQRRGPDGPDGVSRAGRNVVTIIEERATDTGMATVPLPAEG------
M. smegmatis (WP_011727801.1)	MTIIEERATDTGMATVPLPAEG------
M. smegmatis (WP_158169501.1)	MTIIEERATDTGMATVPLPAEG------
M. smegmatis (WP_036463241.1)	MATVPLPAEG------
M. smegmatis (6IOH_A)	ATVPLPAEG------

D

	Description	Scientific Name	Max Score	Total Score	Query Cover	E value	Per. Ident	Acc. Len	Accession
☑	homoserine O-acetyltransferase [Mycolicibacterium smegmatis]	Mycolicibacterium smegmatis	700	700	80%	0.0	99.74%	380	WP_011727801.1
☑	homoserine O-acetyltransferase [Mycolicibacterium smegmatis]	Mycolicibacterium smegmatis	697	697	80%	0.0	99.47%	380	WP_158169501.1
☑	homoserine O-acetyltransferase [Mycolicibacterium smegmatis]	Mycolicibacterium smegmatis	679	679	77%	0.0	100.00%	368	WP_036463241.1
☑	Chain A, Homoserine O-acetyltransferase [Mycolicibacterium smegmatis]	Mycolicibacterium smegmatis	677	677	77%	0.0	100.00%	375	6IOH_A
☑	Chain A, Homoserine O-acetyltransferase [Mycolicibacterium smegmatis]	Mycolicibacterium smegmatis	675	675	77%	0.0	100.00%	374	6IOG_A
☑	homoserine O-acetyltransferase [Mycobacterium goodii]	Mycobacterium goodii	667	667	80%	0.0	94.47%	380	MBU8809204.1
☑	homoserine O-acetyltransferase [Mycobacterium goodii]	Mycobacterium goodii	667	667	80%	0.0	94.74%	380	PJK22580.1
☑	homoserine acetyltransferase [Mycobacterium sp. SWH-M5]	Mycobacterium sp. SWH-M5	665	665	80%	0.0	94.21%	380	OKH66403.1
☑	unnamed protein product [Mycobacterium goodii]	Mycobacterium goodii	652	652	77%	0.0	95.11%	368	WP_216864840.1
☑	homoserine acetyltransferase [Mycobacterium goodii]	Mycobacterium goodii	642	642	77%	0.0	93.21%	368	AKS36772.1

F

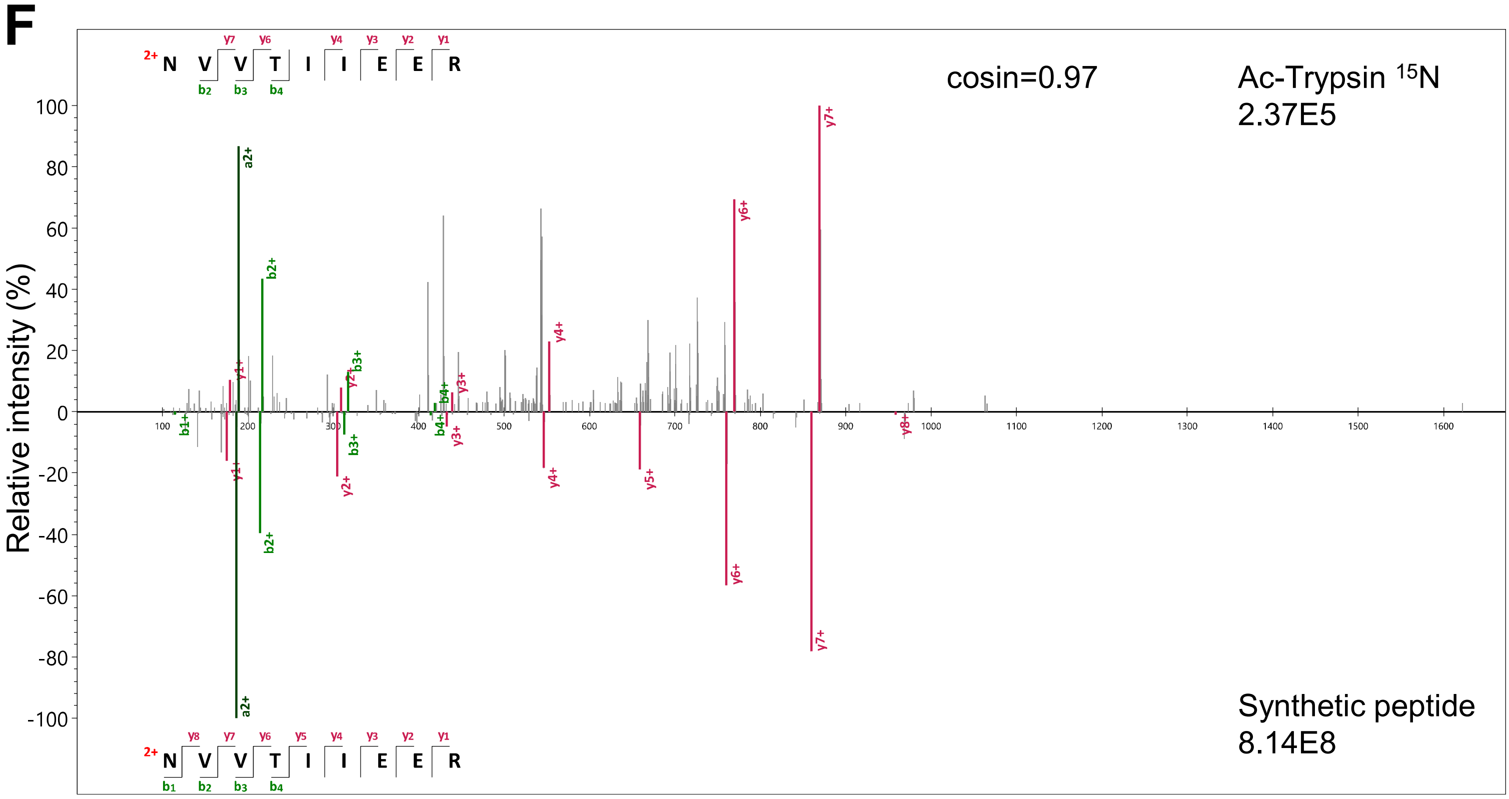

cosin=0.97
Ac-Trypsin 15N
2.37E5
Synthetic peptide
8.14E8
Relative intensity (%)
2+ N V V T I I E E R

No. 3 orf|0|+|1881206-1881701|

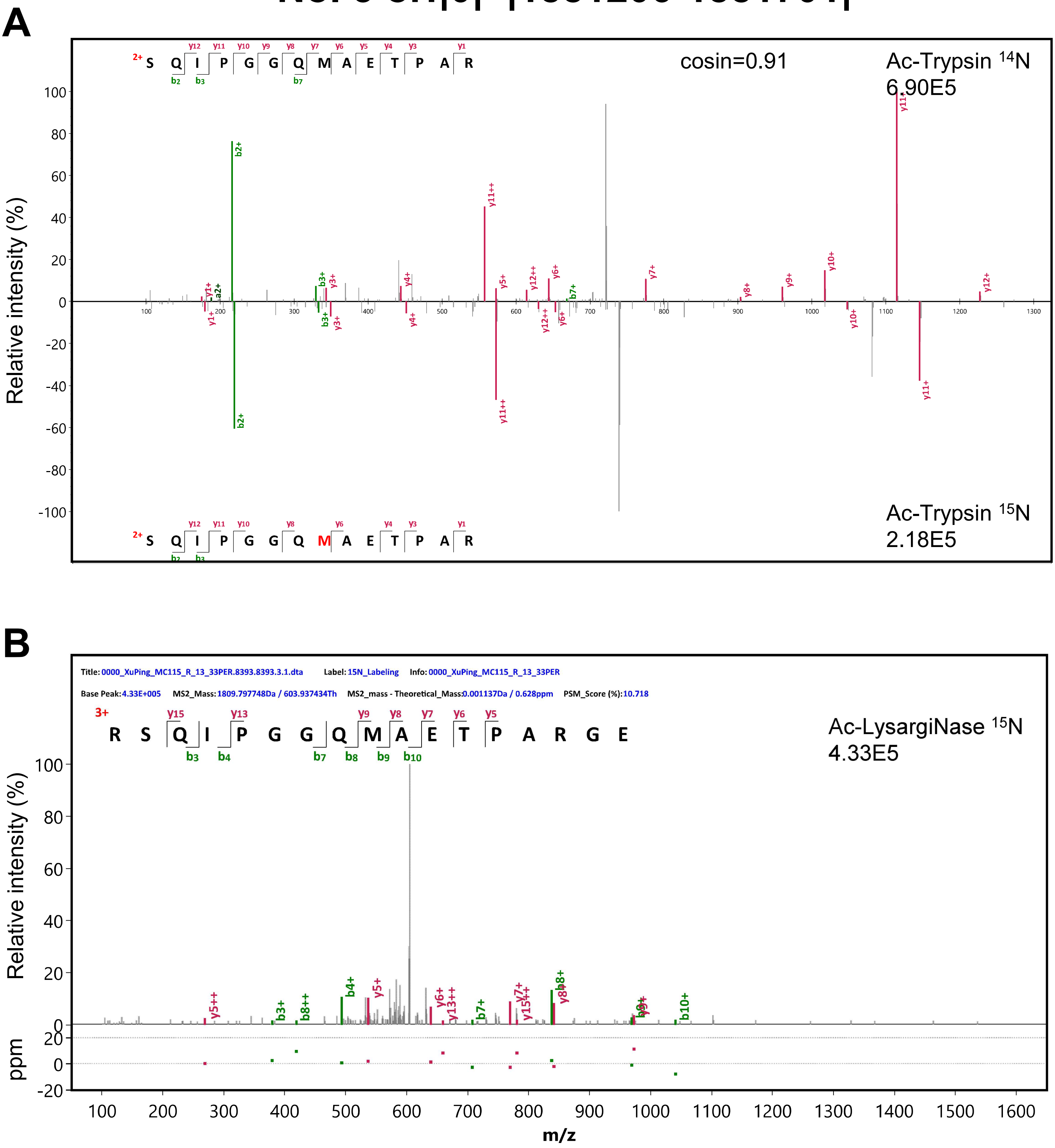

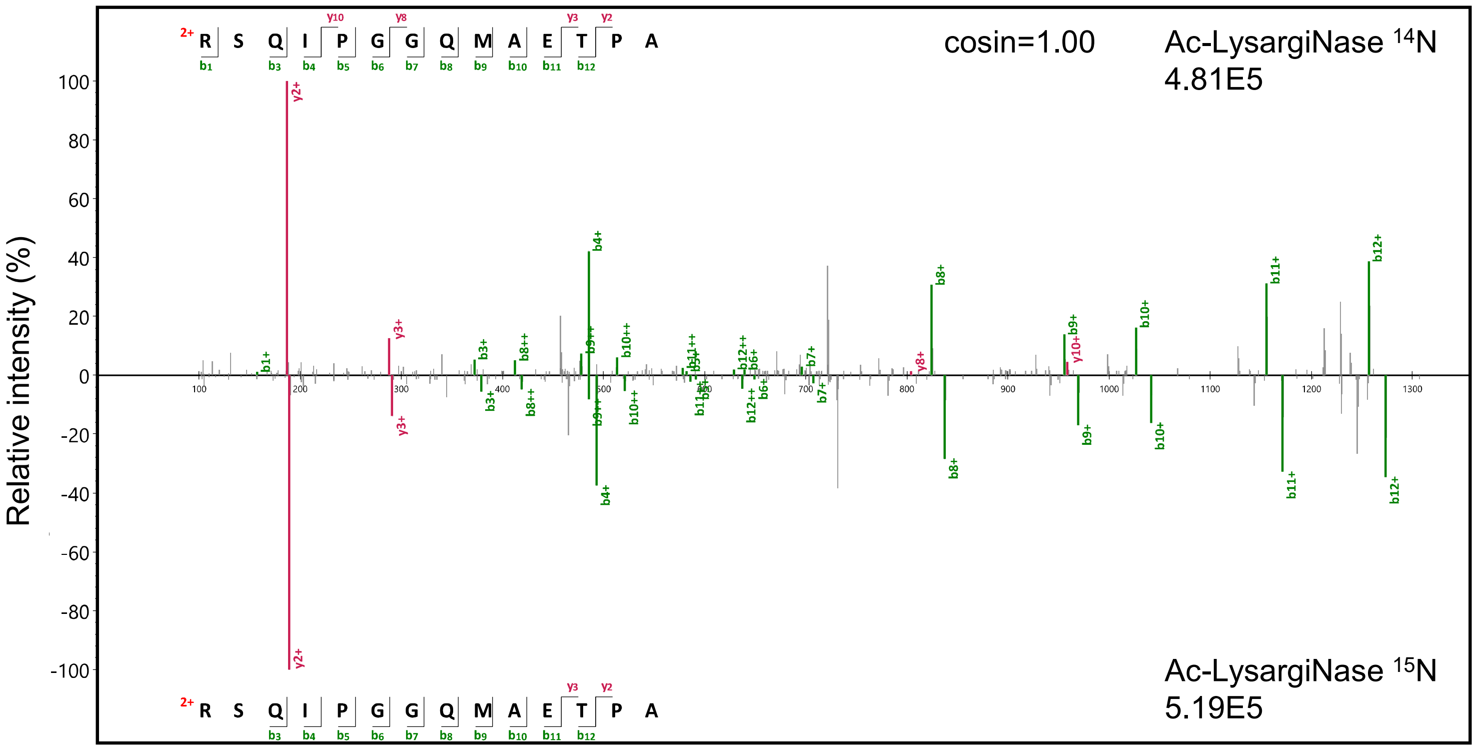

C

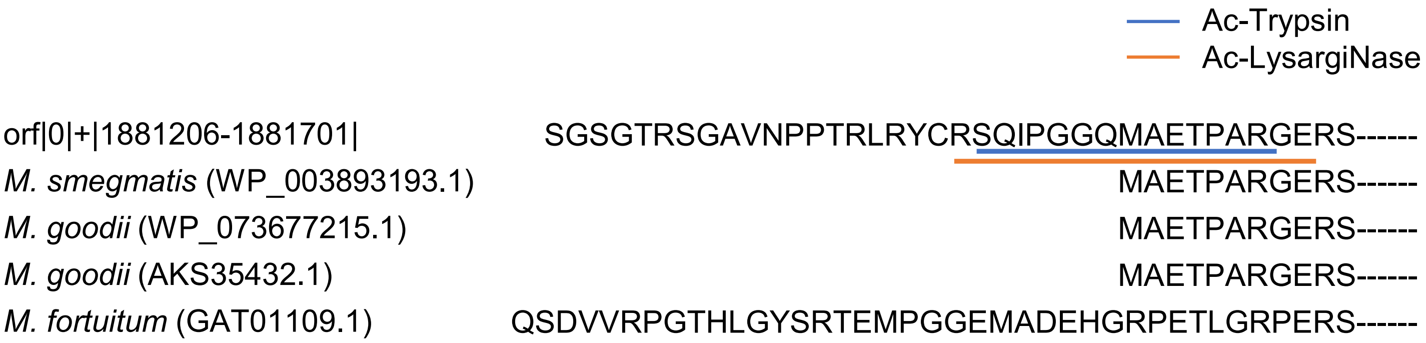

D

	Description	Scientific Name	Max Score	Total Score	Query Cover	E value	Per. Ident	Acc. Len	Accession
☑	anti-sigma B factor RsbW [Mycolicibacterium smegmatis]	Mycolicibacteriu...	248	248	83%	6e-82	100.00%	138	WP_003893193.1
☑	unnamed protein product [Mycobacterium goodii]	Mycobacterium ...	242	242	83%	2e-79	97.10%	138	WP_073677215.1
☑	anti-sigma factor [Mycobacterium goodii]	Mycobacterium ...	236	236	83%	2e-77	95.65%	138	AKS35432.1
☑	anti-sigma factor rsbW [Mycolicibacterium fortuitum subsp. acetamidolyticum]	Mycolicibacteriu...	202	202	96%	2e-63	71.08%	173	GAT01109.1
☑	anti-sigma factor [Mycolicibacterium sp. (ex Dasyatis americana)]	Mycolicibacteriu...	195	195	80%	8e-61	81.95%	144	OFB38220.1
☑	ATP-binding protein [Mycobacterium]	Mycobacterium	194	194	80%	9e-61	81.95%	136	WP_110765068.1
☑	ATP-binding protein [unclassified Mycolicibacterium]	unclassified Myc...	194	194	84%	3e-60	77.86%	144	WP_155916378.1
☑	anti-sigma factor [Mycobacterium sp. DL440]	Mycobacterium ...	193	193	83%	3e-60	78.72%	141	WP_166903195.1
☑	anti-sigma factor [Mycobacteriaceae]	Mycobacteriaceae	193	387	80%	4e-60	83.33%	144	WP_019348831.1
☑	anti-sigma factor [Mycobacteriaceae]	Mycobacteriaceae	193	193	80%	4e-60	83.33%	144	WP_036388772.1

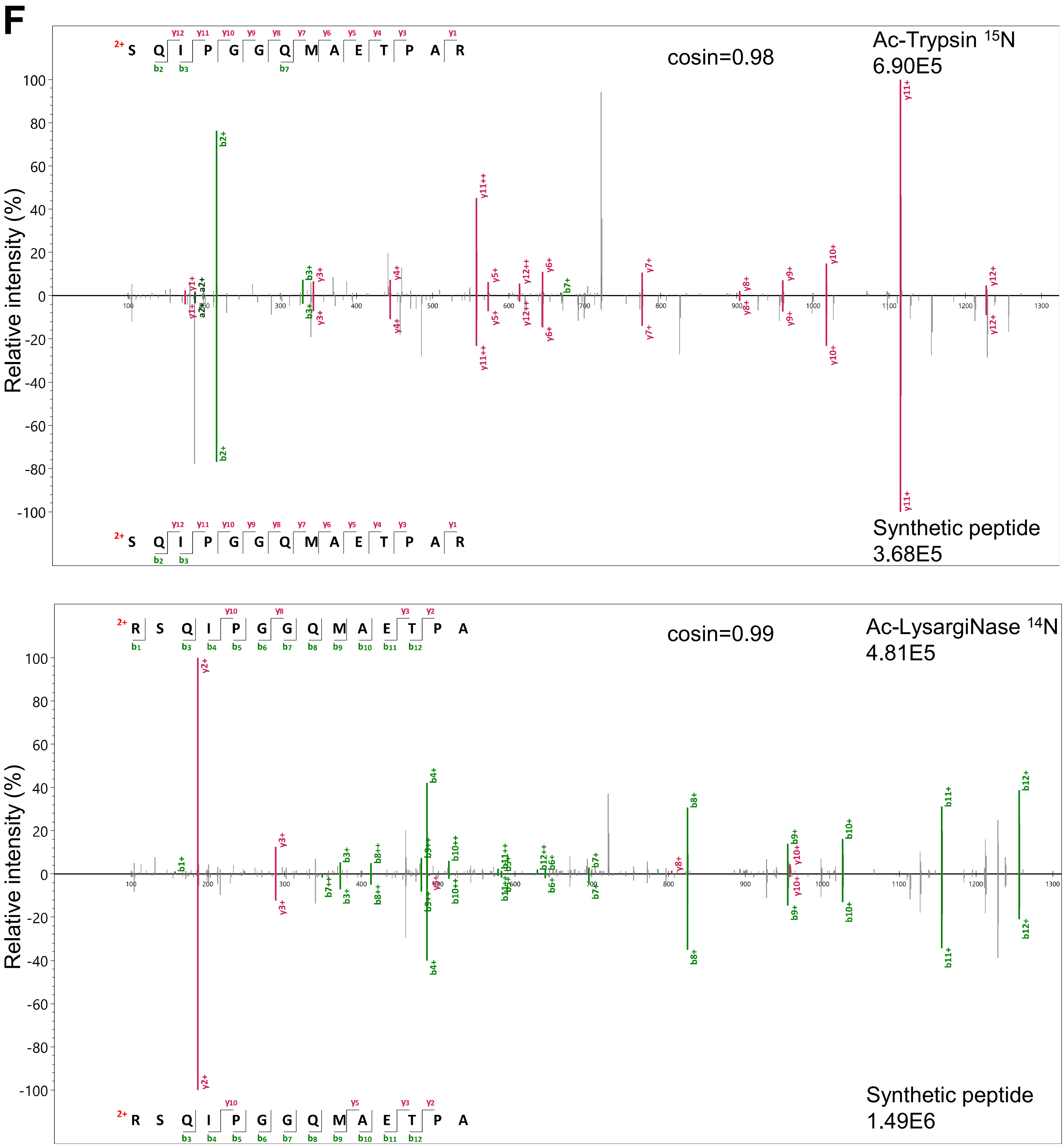
F
Ac-Trypsin 15N
6.90E5
cosin=0.98
Synthetic peptide
3.68E5
2+ S Q I P G G Q M A E T P A R
Relative intensity (%)
Ac-LysargiNase 14N
4.81E5
cosin=0.99
Synthetic peptide
1.49E6
2+ R S Q I P G G Q M A E T P A

No. 4 orf|0|+|3349519-3351547|

A

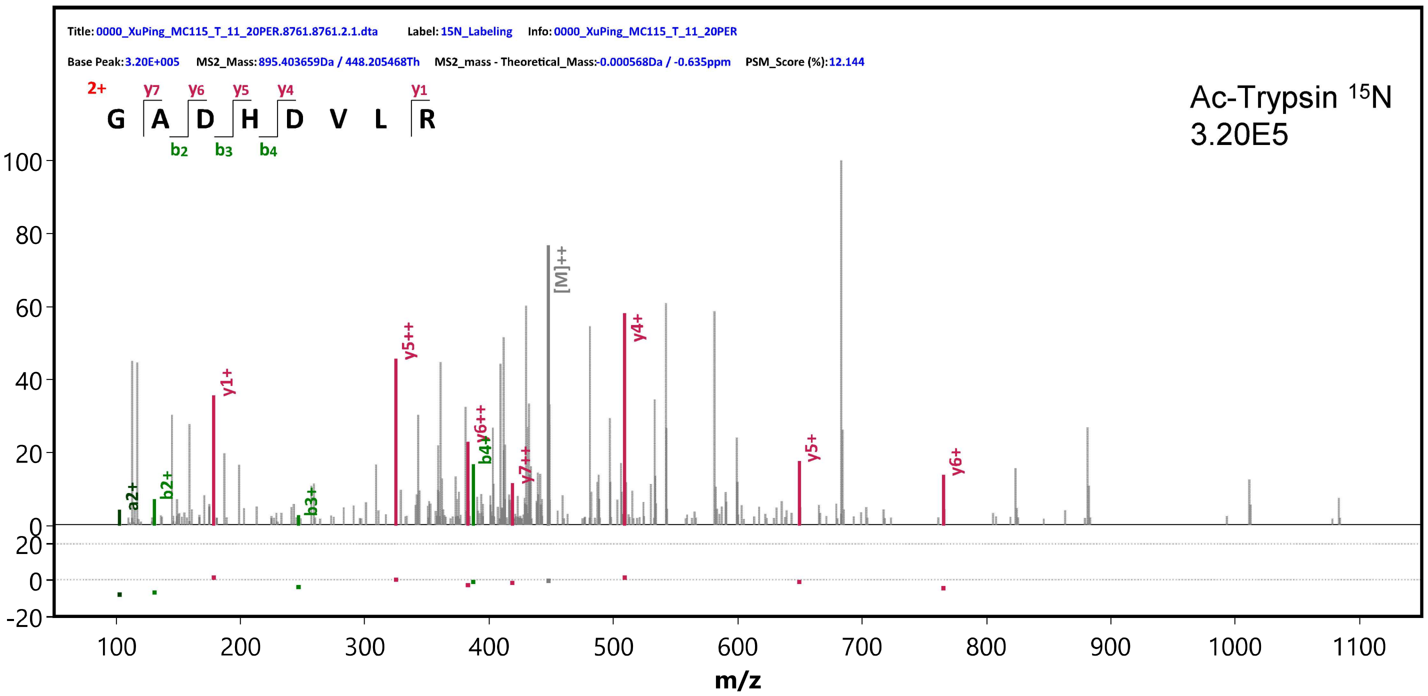

B

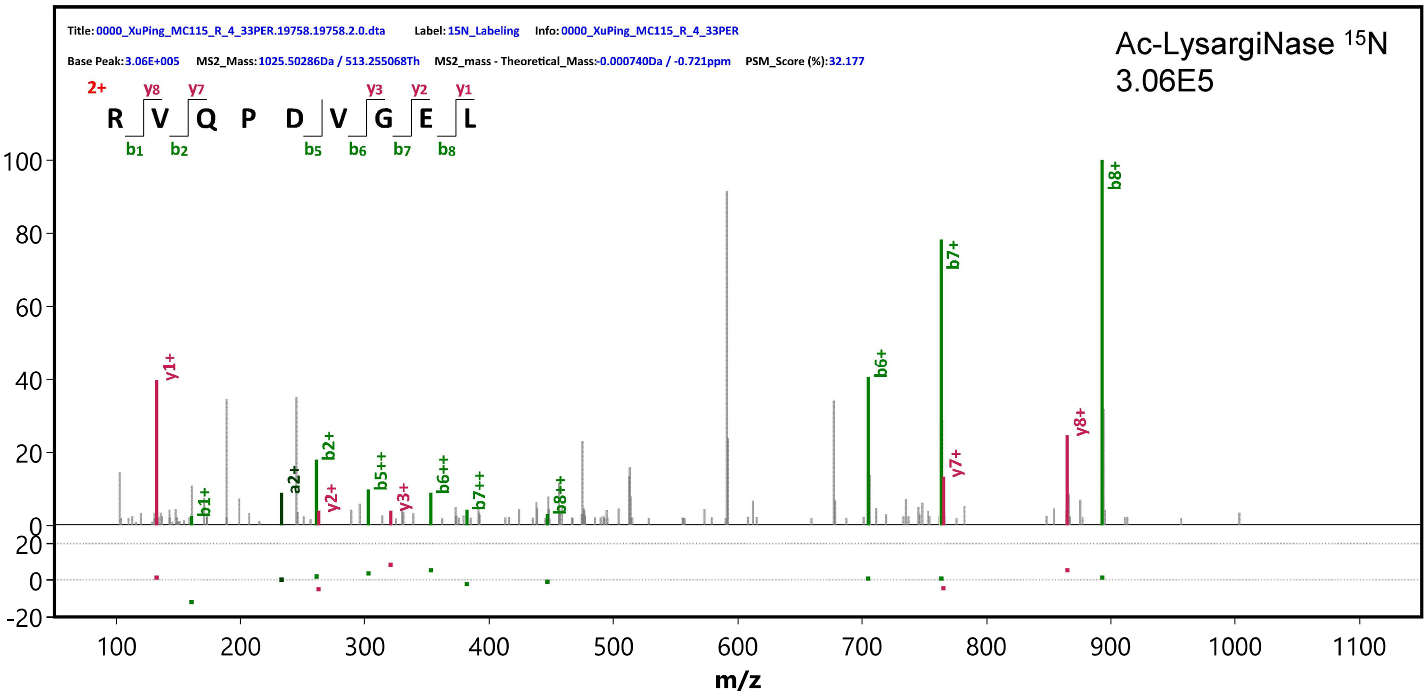

C

Ac-Trypsin
Ac-LysargiNase

orf|0|+|3349519-3351547| ACLARRSAVLPGRSRDHVQ
QNRIAAGPEAVVHRIGGRRCGPGVLVRLLAVPDLLDADAGTEERR
GADHDVLRVQPDVGELRHGADRQGHRDDERRLQGVAADQPAEL
RWRGDRLAAHRHPGGVRGRSLAVQGLQRPDVHHVVVPVRPGAD
GDRAAVRDLQPDRAVRHQGRHDLGAATGHHAARGVDPAVLLPGS
ARGSGTGGTARRLHPAARVPHGRVADRATGYRRGRAARVHLRVE
QLCVPAHPRRQQCRHGDRRDHQIPRWRWPGVLQPDRRGRHHR
RASTADPGSDHPAVPGARPVVRGGEGLMATVSIAGVHKSF------

M. smegmatis (WP_011728939.1) MATVSIAGVHKSF------
M. smegmatis (WP_158166676.1) MATVSIAGVHKSF------
M. smegmatis (WP_003894673.1) MATVSIAGVHKSF------
M. smegmatis (WP_233045489.1) MATVSIAGVHKSF------

D

	Description	Scientific Name	Max Score	Total Score	Query Cover	E value	Per. Ident	Acc. Len	Accession
☑	ABC transporter ATP-binding protein [Mycolicibacterium smegmatis]	Mycolicibacterium smegmatis	734	734	54%	0.0	100.00%	369	WP_011728939.1
☑	ABC transporter ATP-binding protein [Mycolicibacterium smegmatis]	Mycolicibacterium smegmatis	733	733	54%	0.0	99.73%	369	WP_158166676.1
☑	ABC transporter ATP-binding protein [Mycolicibacterium smegmatis]	Mycolicibacterium smegmatis	730	730	54%	0.0	99.19%	369	WP_003894673.1
☑	ABC transporter ATP-binding protein [Mycolicibacterium smegmatis]	Mycolicibacterium smegmatis	724	724	54%	0.0	98.10%	369	WP_233045489.1
☑	ABC transporter ATP-binding protein [Mycolicibacterium smegmatis]	Mycolicibacterium smegmatis	723	723	54%	0.0	97.83%	369	WP_058126210.1
☑	ABC transporter [Mycobacterium goodii]	Mycobacterium goodii	709	709	54%	0.0	96.51%	372	AKS34361.1
☑	ABC transporter ATP-binding protein [Mycobacterium goodii]	Mycobacterium goodii	707	707	54%	0.0	96.21%	369	MBU8815300.1
☑	unnamed protein product [Mycobacterium goodii]	Mycobacterium goodii	707	707	54%	0.0	95.93%	369	WP_214385759.1
☑	ABC transporter [Mycobacterium sp. SWH-M5]	Mycobacterium sp. SWH-M5	706	706	54%	0.0	95.93%	369	OKH67074.1
☑	ABC transporter ATP-binding protein [Mycobacterium goodii]	Mycobacterium goodii	705	705	54%	0.0	95.93%	369	MBU8834727.1

No. 5 orf|0|+|4323460-4324567|

A

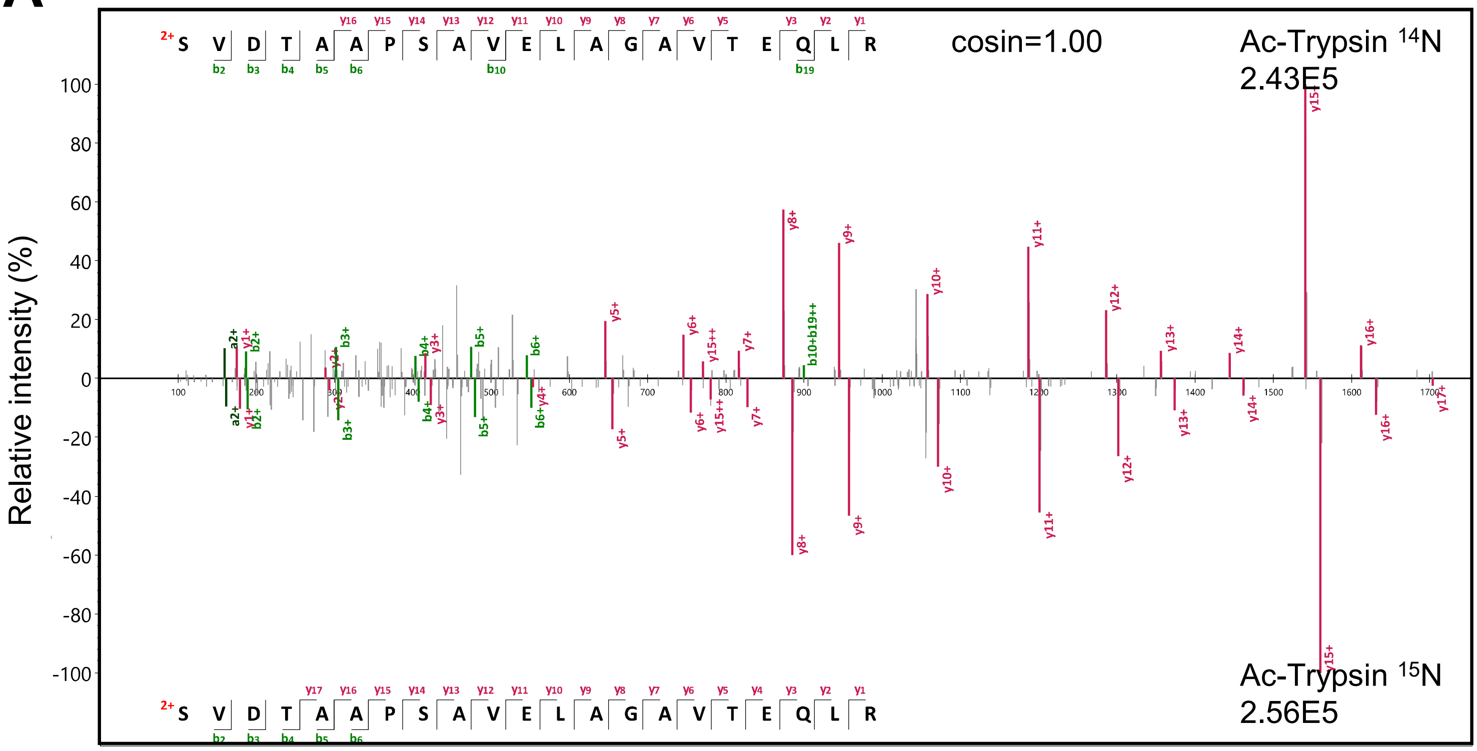

B

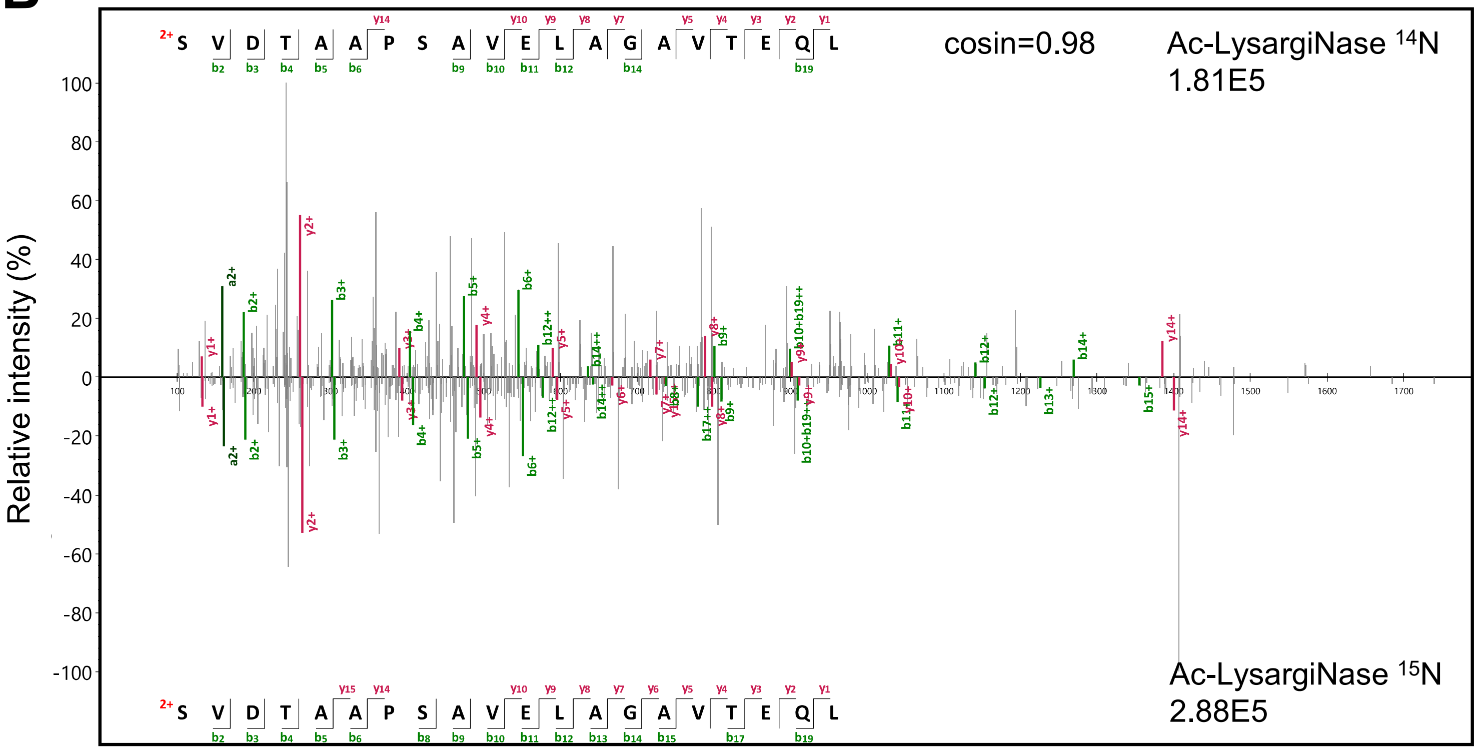

C

Ac-Trypsin
Ac-LysargiNase

orf|0|+|4323460-4324567|	RKGRALSVDTAAPSAVELAGAVTEQLREYLRERRS------
M. smegmatis (WP_011729667.1)	VDTAAPSAVELAGAVTEQLREYLRERRS------
M. smegmatis (WP_036453351.1)	VDTAAPSAVELAGAVTEQLREYLRERRS------
M. smegmatis (WP_233046249.1)	VDTAAPSAVELAGAVTEQLREYLRERRS------
M. smegmatis (WP_014877980.1)	LAGAVTEQLREYLRERRS------

D

Description	Scientific Name	Max Score	Total Score	Query Cover	E value	Per. Ident	Acc. Len	Accession
polyprenyl synthetase family protein [Mycolicibacterium smegmatis]	Mycolicibacterium smegmatis	624	624	98%	0.0	99.17%	362	WP_036453351.1
polyprenyl synthetase family protein [Mycolicibacterium smegmatis]	Mycolicibacterium smegmatis	623	623	98%	0.0	99.72%	362	WP_011729667.1
polyprenyl synthetase family protein [Mycolicibacterium smegmatis]	Mycolicibacterium smegmatis	622	622	98%	0.0	99.45%	362	WP_233046249.1
polyprenyl synthetase family protein [Mycolicibacterium smegmatis]	Mycolicibacterium smegmatis	605	605	95%	0.0	99.72%	352	WP_014877980.1
polyprenyl synthetase family protein [Mycobacterium goodii]	Mycobacterium goodii	587	587	98%	0.0	93.09%	362	MBU8820236.1
unnamed protein product [Mycobacterium goodii]	Mycobacterium goodii	585	585	98%	0.0	93.09%	362	WP_214390316.1
geranylgeranyl pyrophosphate synthase [Mycobacterium goodii]	Mycobacterium goodii	585	585	98%	0.0	93.09%	362	PJK20876.1
geranylgeranyl pyrophosphate synthase [Mycobacterium goodii]	Mycobacterium goodii	575	575	98%	0.0	93.92%	362	AKS33624.1
polyprenyl synthetase family protein [Mycobacterium goodii]	Mycobacterium goodii	566	566	98%	0.0	93.37%	362	MBU8813764.1
polyprenyl synthetase family protein [Mycobacterium goodii]	Mycobacterium goodii	566	566	98%	0.0	93.65%	362	MBU8823943.1

E

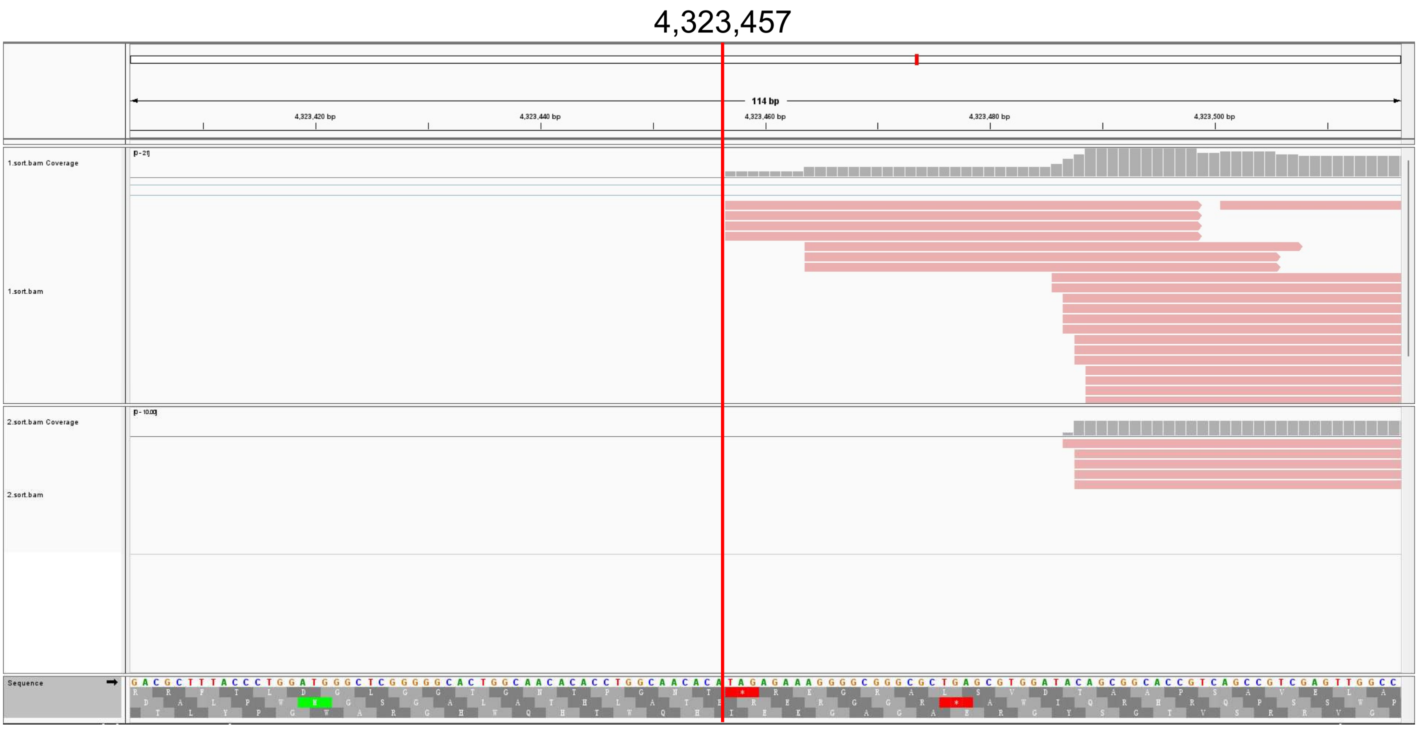

No. 6 orf|0|+|5448467-5449274|

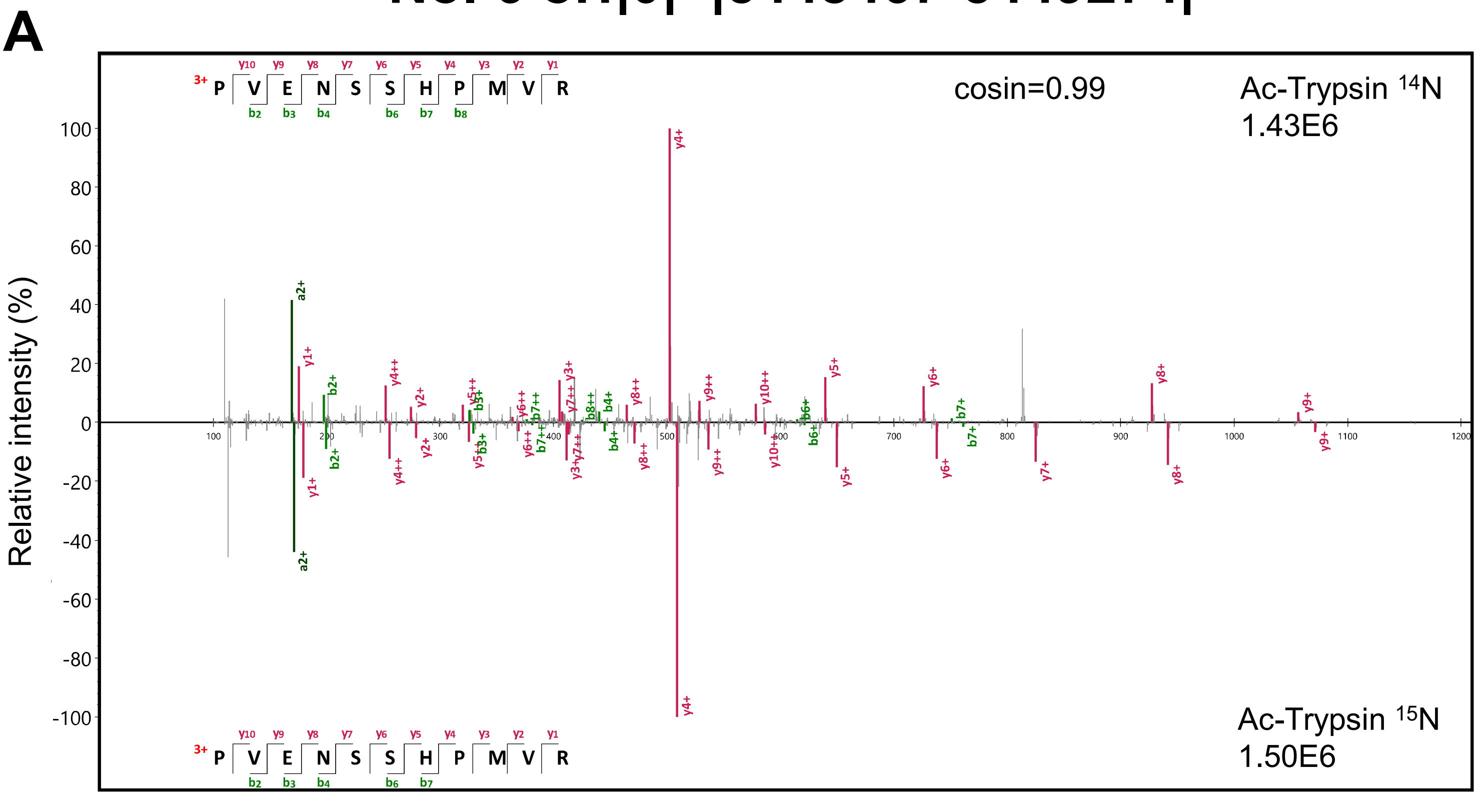

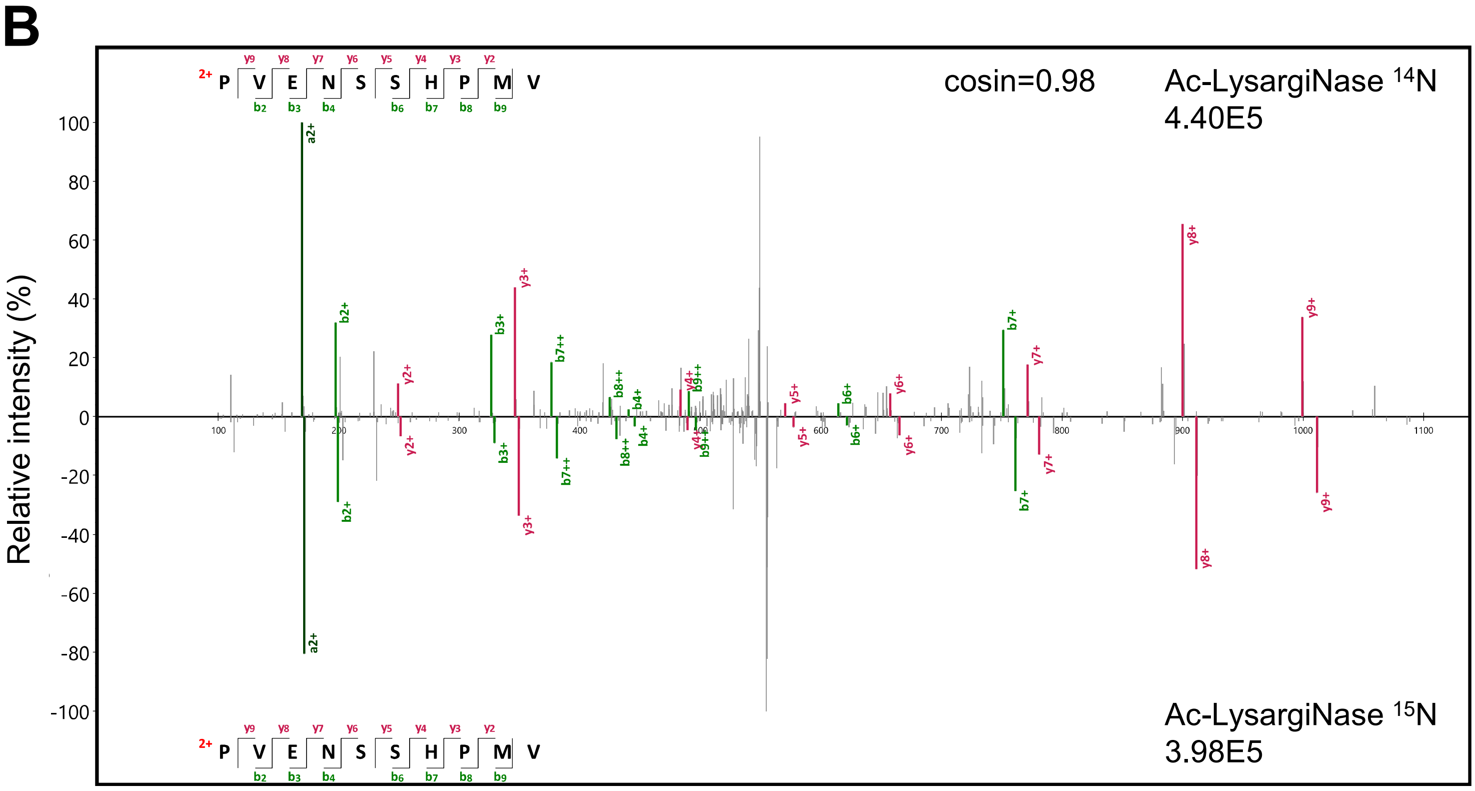

C

Ac-Trypsin
Ac-LysargiNase

orf|0|+|5448467-5449274|	GNWRSALPVENSSHPMVRFENVVKRFG------
M. smegmatis (WP_003896768.1)	MVRFENVVKRFG------
M. smegmatis (WP_058126884.1)	MVRFENVVKRFG------
M. smegmatis (WP_239708967.1)	MVRFENVVKRFG------
M. vanbaalenii (WP_159234487.1)	MVRFENVVKRFG------

D

	Description	Scientific Name	Max Score	Total Score	Query Cover	E value	Per. Ident	Acc. Len	Accession
☑	ectoine/hydroxyectoine ABC transporter ATP-binding protein EhuA [Mycolicibacterium smegmatis]	Mycolicibacteriu...	508	508	94%	0.0	100.00%	254	WP_003896768.1
☑	ectoine/hydroxyectoine ABC transporter ATP-binding protein EhuA [Mycolicibacterium smegmatis]	Mycolicibacteriu...	504	504	94%	2e-179	99.21%	254	WP_058126884.1
☑	unnamed protein product [Mycolicibacterium smegmatis]	Mycolicibacteriu...	502	502	94%	7e-179	98.82%	254	WP_239708967.1
☑	ectoine/hydroxyectoine ABC transporter ATP-binding protein EhuA [Mycolicibacterium vanbaalenii]	Mycolicibacteriu...	474	474	94%	1e-167	92.52%	254	WP_159234487.1
☑	ectoine/hydroxyectoine ABC transporter ATP-binding protein EhuA [Mycolicibacterium hippocampi]	Mycolicibacteriu...	473	473	94%	2e-167	92.91%	254	WP_163894083.1
☑	L-cystine ABC transporter ATP-binding protein YecC [Mycolicibacterium duvalii]	Mycolicibacteriu...	469	469	97%	1e-165	87.83%	263	BBX17245.1
☑	L-cystine ABC transporter ATP-binding protein YecC [Mycolicibacterium chitae]	Mycolicibacteriu...	468	468	97%	4e-165	86.84%	266	BBZ03551.1
☑	ectoine/hydroxyectoine ABC transporter ATP-binding protein EhuA [Mycolicibacterium chitae]	Mycolicibacteriu...	462	462	94%	5e-163	89.37%	254	WP_126336726.1
☑	ectoine/hydroxyectoine ABC transporter ATP-binding protein EhuA [Mycolicibacterium duvalii]	Mycolicibacteriu...	462	462	94%	5e-163	89.76%	254	WP_098001869.1
☑	ectoine/hydroxyectoine ABC transporter ATP-binding protein EhuA [Rhodococcus pyridinivorans]	Rhodococcus py...	395	395	97%	2e-136	75.10%	274	AWZ24582.1

Supplementary Figure 8

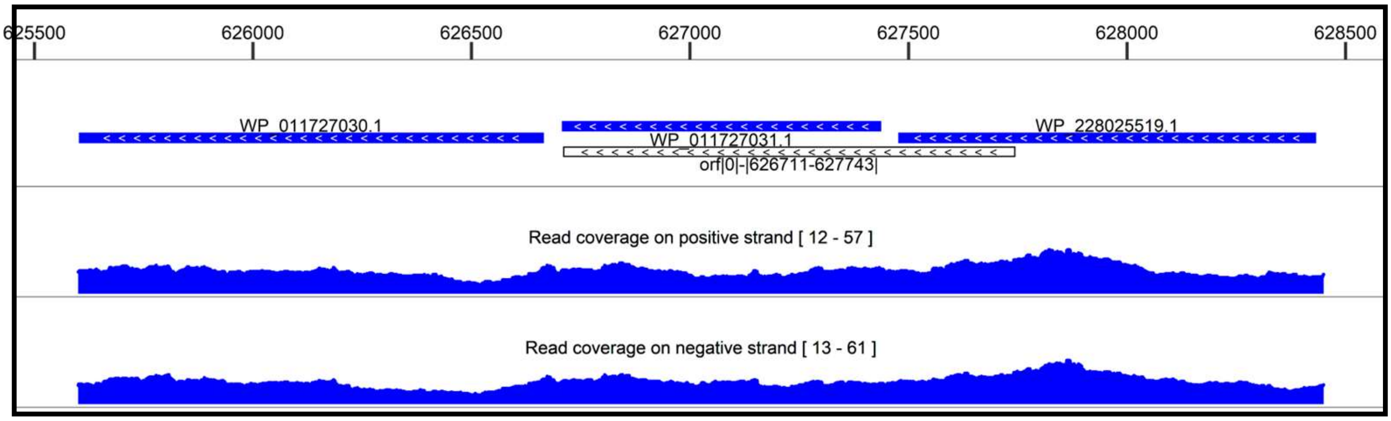

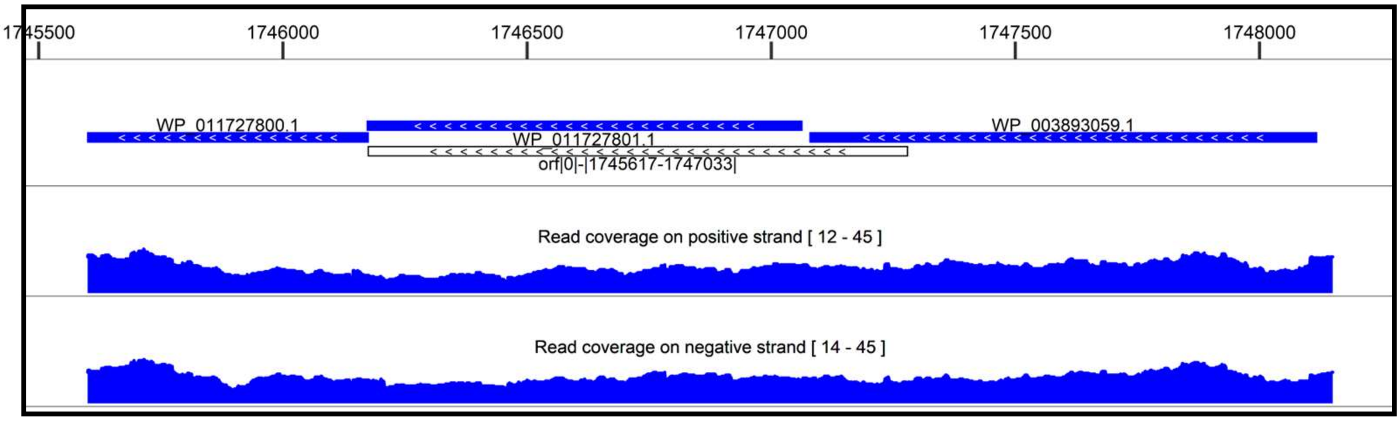

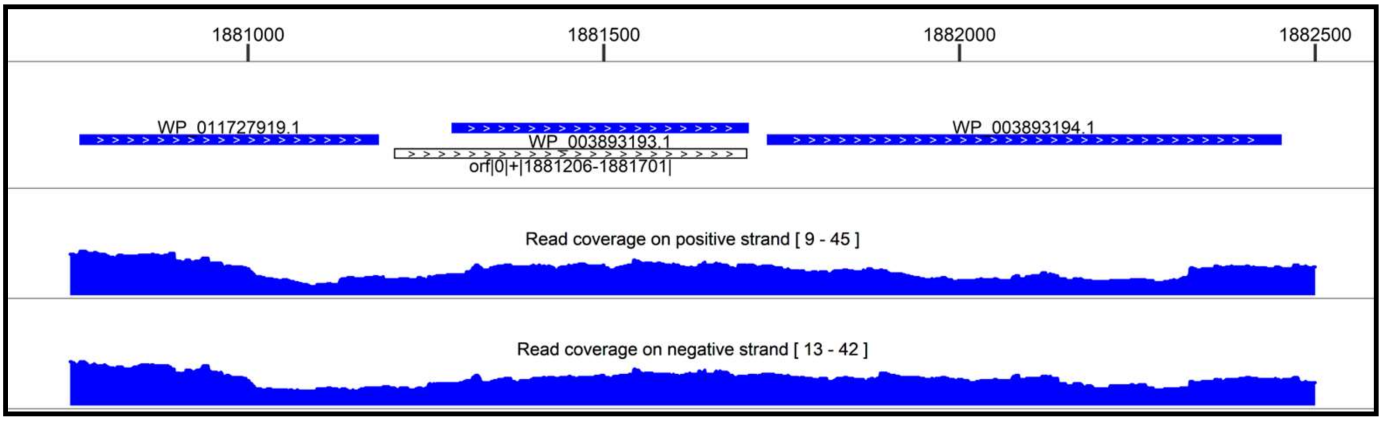

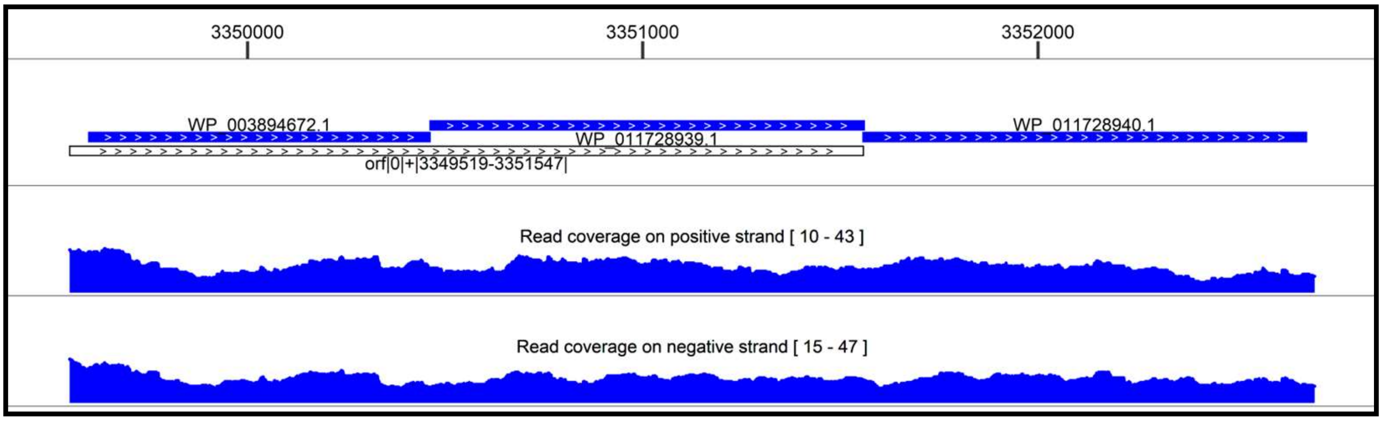
3350000
3351000
3352000
WP_003894672.1
WP_011728939.1
WP_011728940.1
orf|0|+|3349519-3351547|
Read coverage on positive strand [10 - 43]
Read coverage on negative strand [15 - 47]

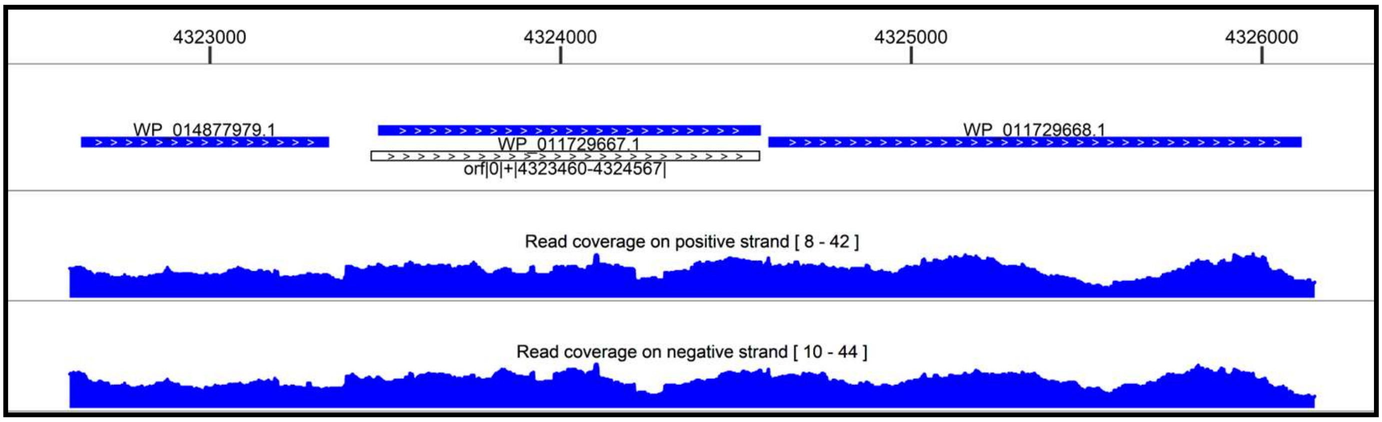
4323000
4324000
4325000
4326000
WP_014877979.1
WP_011729667.1
WP_011729668.1
orf|0|+|4323460-4324567|
Read coverage on positive strand [8 - 42]
Read coverage on negative strand [10 - 44]

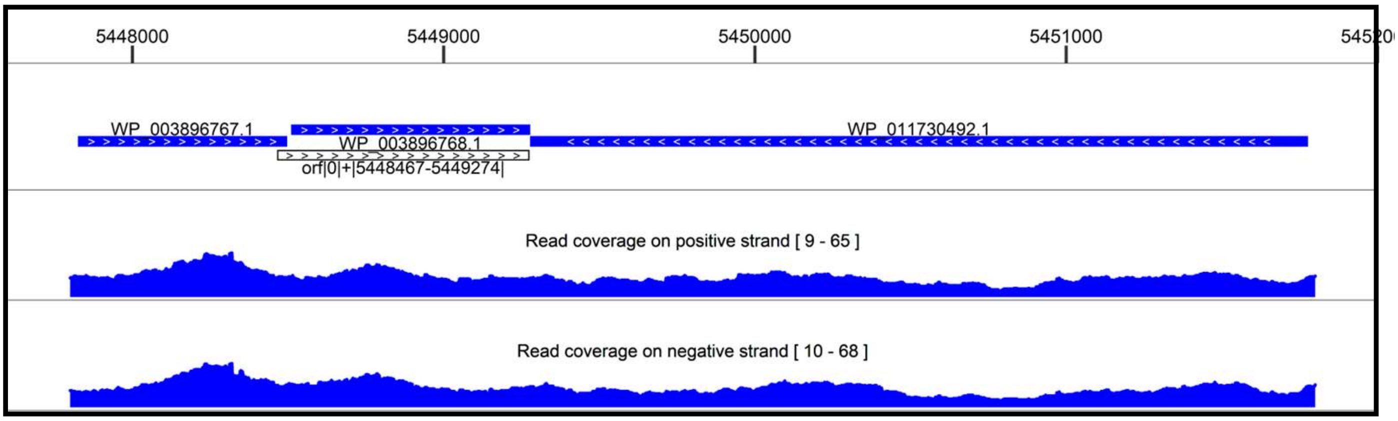
5448000
5449000
5450000
5451000
WP_003896767.1
WP_003896768.1
WP_011730492.1
orf|0|+|5448467-5449274|
Read coverage on positive strand [9 - 65]
Read coverage on negative strand [10 - 68]

Supplementary Figure 9

No. 1 orf|0|+|1060315-1061149|

A

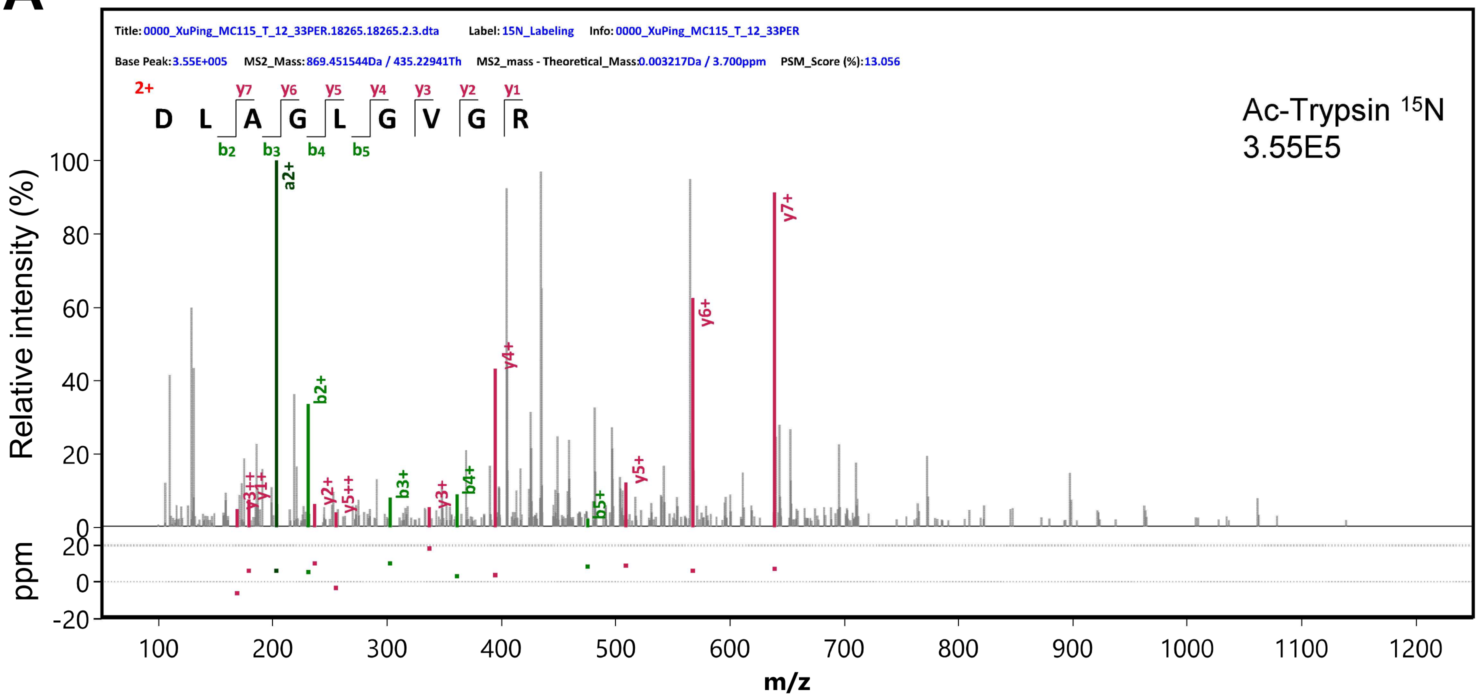

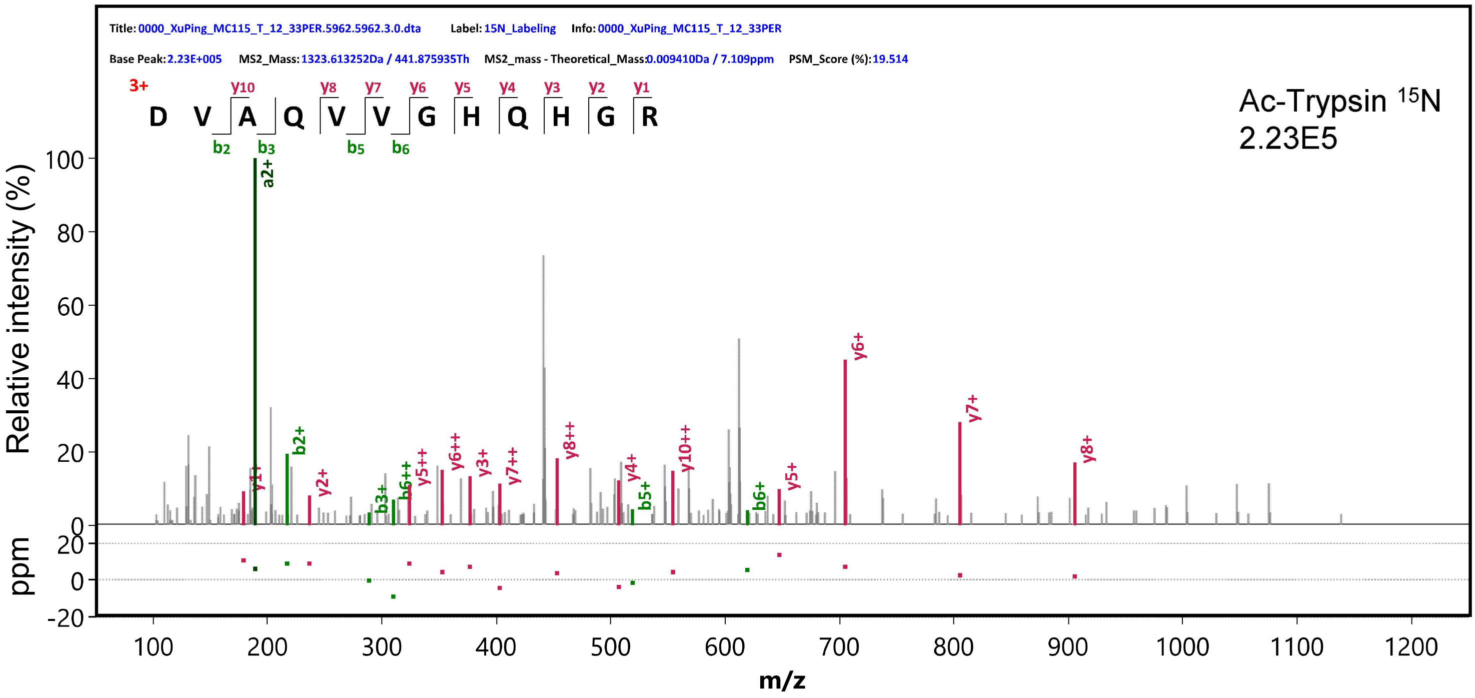

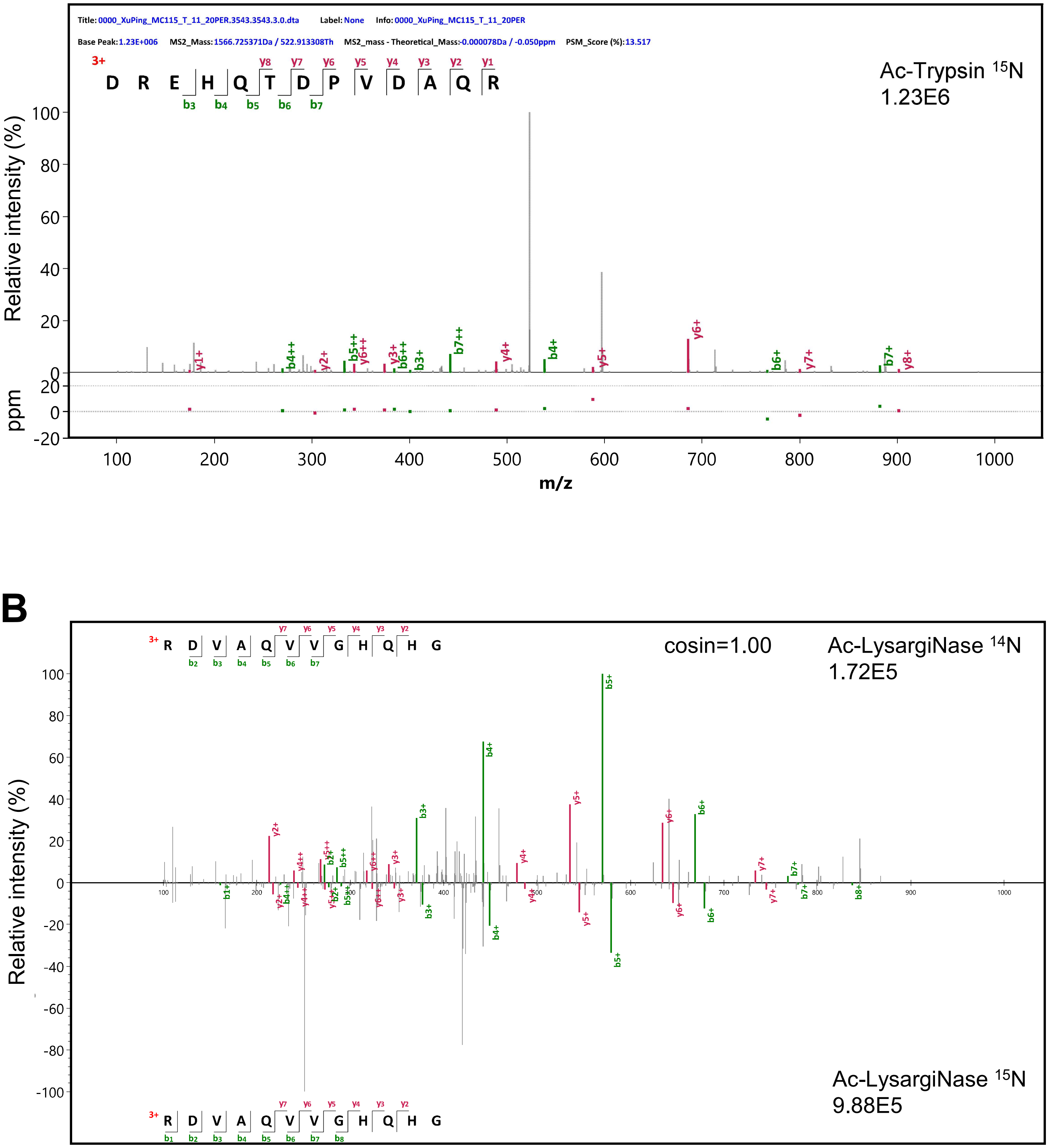

Title: 0000_XuPing_MC115_T_11_20PER.3543.3543.3.0.dta Label: None Info: 0000_XuPing_MC115_T_11_20PER
Base Peak: 1.23E+006 MS2_Mass: 1566.725371Da / 522.913308Th MS2_mass - Theoretical_Mass: -0.000078Da / -0.050ppm PSM_Score (%): 13.517
3+
D R E H Q T D P V D A Q R
Ac-Trypsin ^{15}N
1.23E6
Relative intensity (%)
ppm
m/z
B
3+ R D V A Q V V G H Q H G
cosin=1.00
Ac-LysargiNase ^{14}N
1.72E5
Relative intensity (%)
Ac-LysargiNase ^{15}N
9.88E5
3+ R D V A Q V V G H Q H G

C

‹ Edit Search | Save Search | Search Summary ⌄ | How to read this report? | BLAST Help Videos | Back to Traditional Results Page

Job Title	orf|0|+|1060315-1061149|
RID	61K60340016 Search expires on 04-22 11:17 am Download All ⌄
Program	Citation ⌄
Database	nr See details ⌄
Query ID	lcl|Query_7712
Description	orf|0|+|1060315-1061149|
Molecule type	amino acid
Query Length	278
Other reports	

Filter Results

Percent Identity ___ to ___ | E value ___ to ___ | Query Coverage ___ to ___

Filter | Reset

⚠ No significant similarity found. For reasons why, click here

D

	Description	Scientific Name	Max Score	Total Score	Query Cover	E value	Per. Ident	Acc. Len	Accession
☑	Mycobacterium goodii strain X7B, complete genome	Mycobacteriu...	1064	1064	95%	0.0	90.73%	7105933	CP012150.1
☑	Mycobacterium goodii strain ATCC 700504 chromosome, complete genome	Mycobacteriu...	904	904	95%	0.0	87.03%	6741281	CP092364.1
☑	Mycolicibacterium litorale NIIDNTM18 DNA, complete genome	Mycolicibacter...	749	749	98%	0.0	83.15%	5634149	AP023287.1
☑	Mycolicibacterium septicum strain PDNC012 chromosome	Mycolicibacter...	747	747	94%	0.0	83.98%	6515693	CP070349.1
☑	Mycolicibacterium boenickei JCM 15653 DNA, complete genome	Mycolicibacter...	743	743	94%	0.0	83.94%	6563937	AP022579.1
☑	Mycolicibacterium fortuitum strain W4 chromosome	Mycolicibacter...	741	741	92%	0.0	84.08%	6674224	CP060409.1
☑	Mycolicibacterium boenickei strain PDNC014 chromosome, complete genome	Mycolicibacter...	741	741	94%	0.0	83.88%	7107504	CP070348.1
☑	Mycolicibacterium mageritense JCM 12375 DNA, complete genome	Mycolicibacter...	732	732	93%	0.0	83.65%	8006721	AP022567.1
☑	Mycolicibacterium alvei JCM 12272 DNA, complete genome	Mycolicibacter...	732	732	94%	0.0	83.63%	5712683	AP022565.1
☑	Mycolicibacterium nivoides strain DL90 chromosome	Mycolicibacter...	719	719	93%	0.0	83.44%	6905961	CP034072.1

E

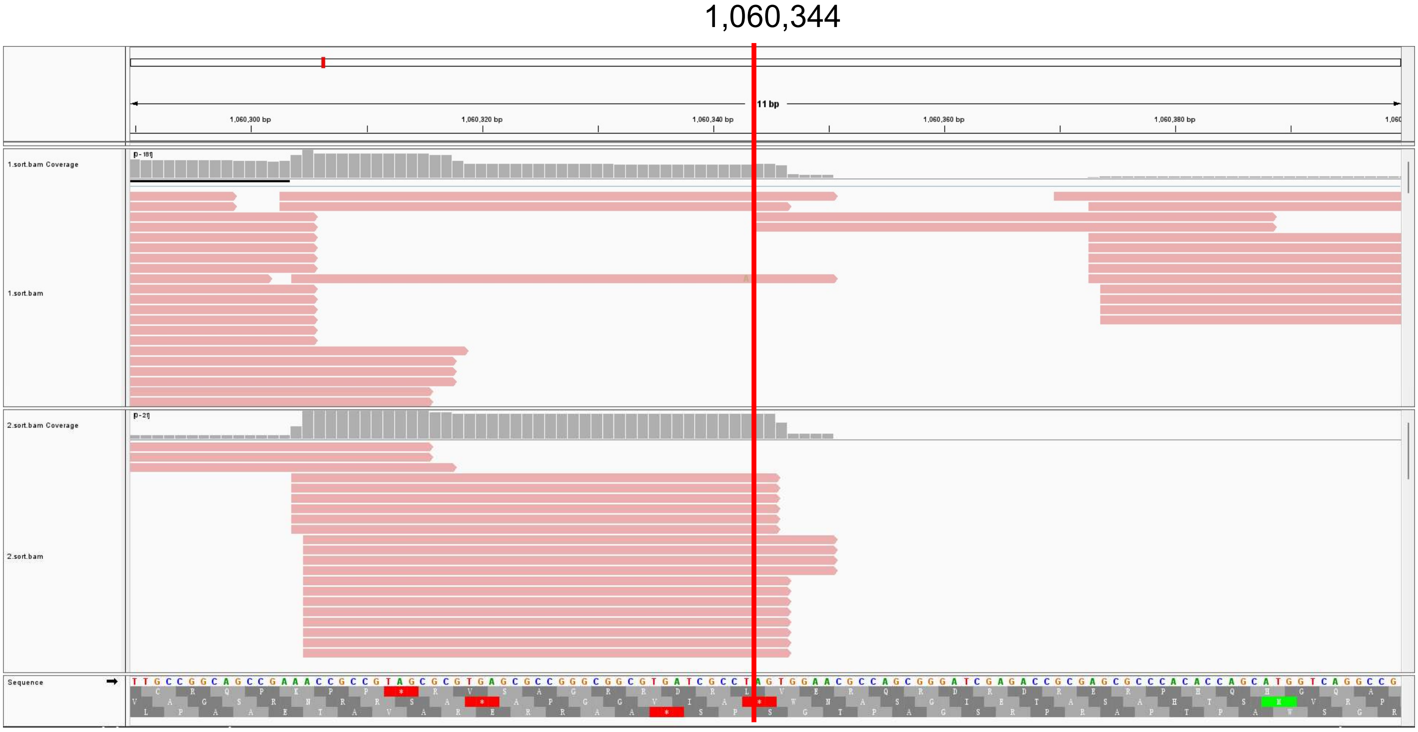

F

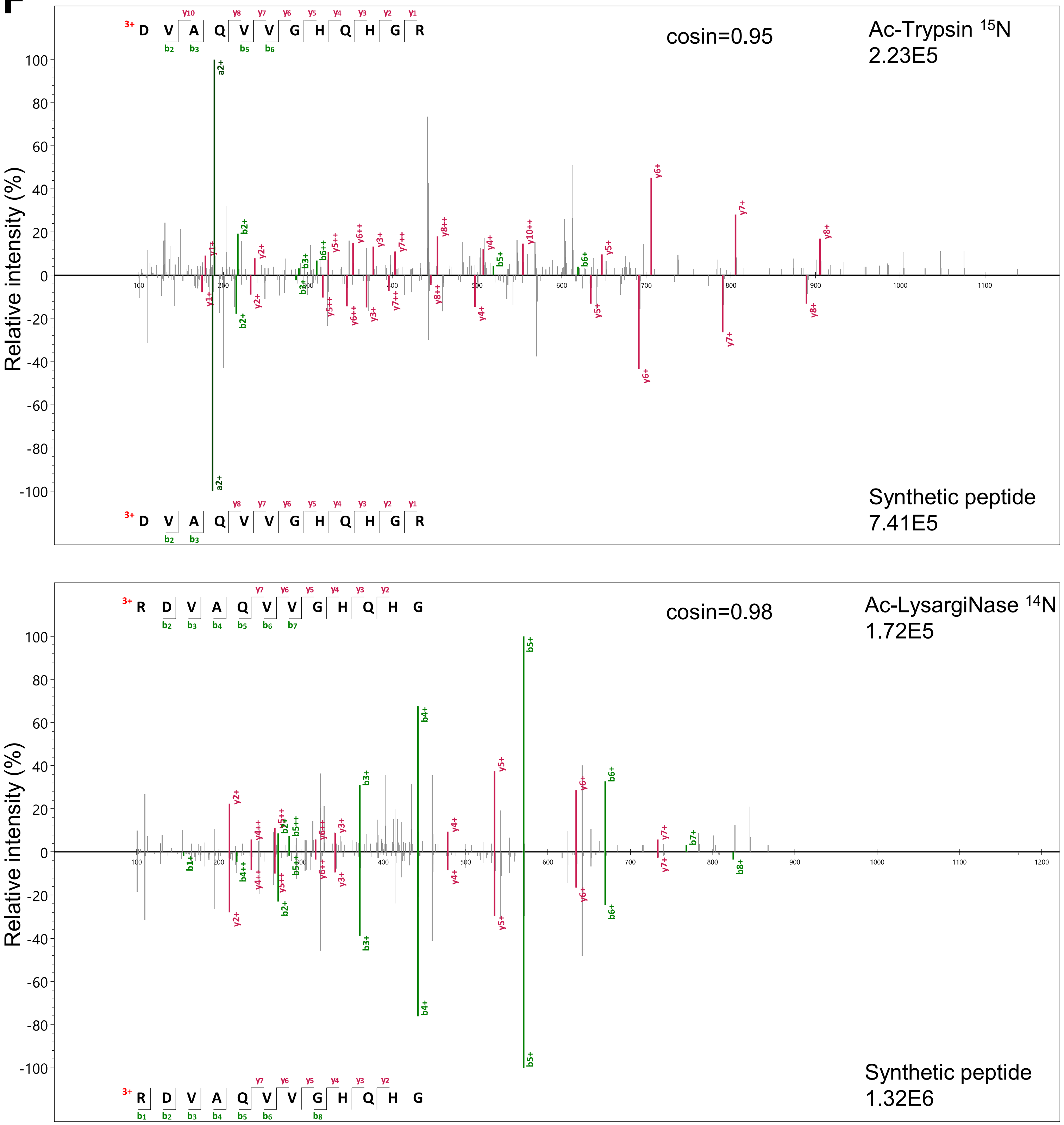

3+ D V A Q V V G H Q H G R
cosin=0.95
Ac-Trypsin 15N
2.23E5
Synthetic peptide
7.41E5
Relative intensity (%)
3+ R D V A Q V V G H Q H G
cosin=0.98
Ac-LysargiNase 14N
1.72E5
Synthetic peptide
1.32E6
Relative intensity (%)

No. 2 orf|0|-|1217294-1218644|

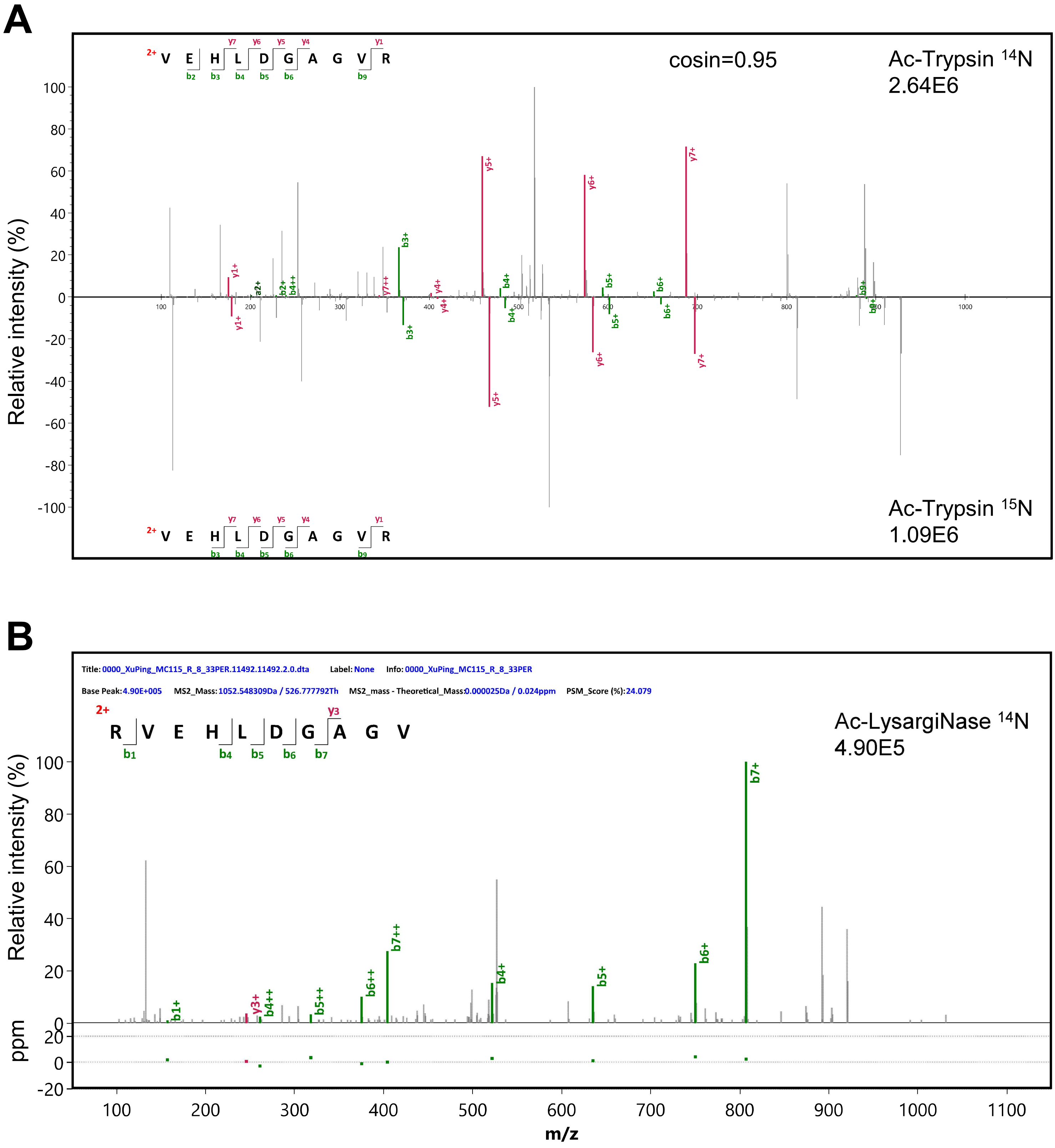

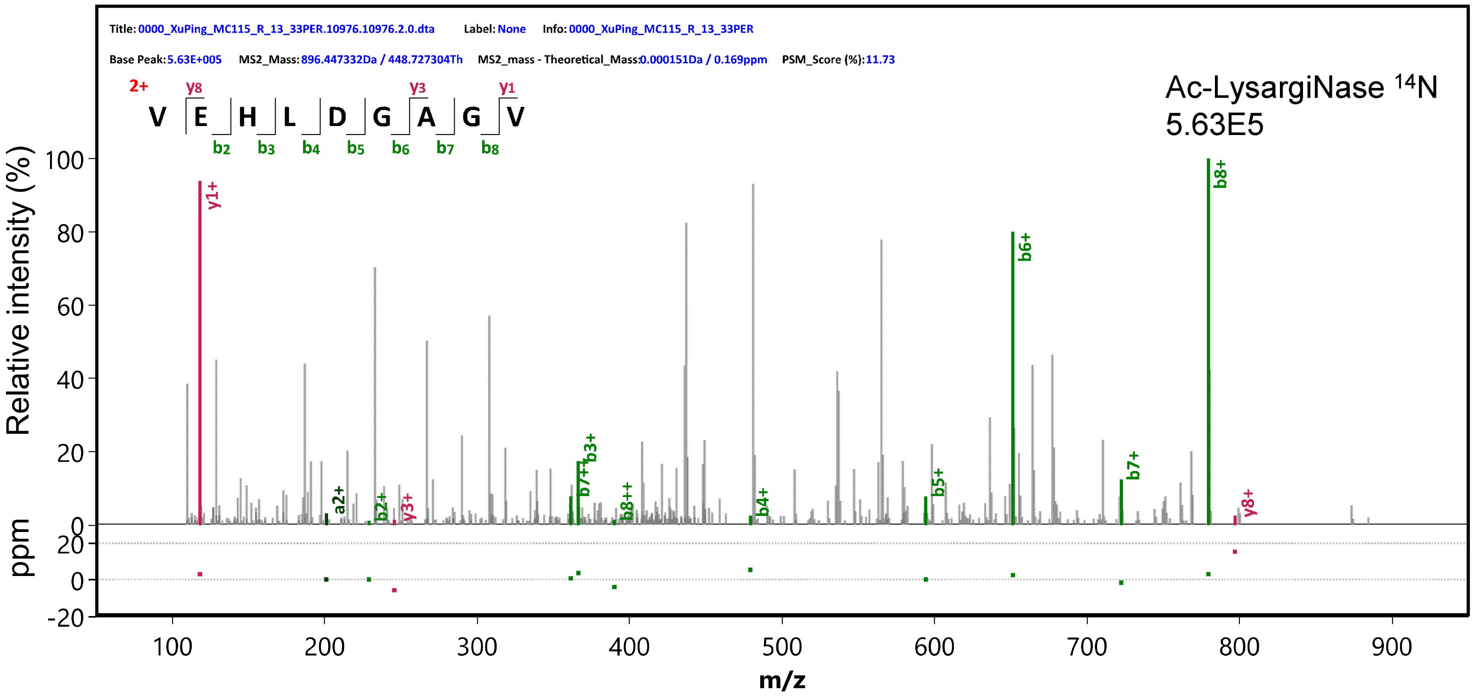

C

‹ Edit Search | Save Search | Search Summary

How to read this report? | BLAST Help Videos | Back to Traditional Results Page

Job Title	orf|0|-|1217294-1218644|
RID	61KBNZA2013 Search expires on 04-22 11:20 am Download All
Program	Citation
Database	nr See details
Query ID	lcl|Query_16919
Description	orf|0|-|1217294-1218644|
Molecule type	amino acid
Query Length	450
Other reports	

Filter Results

Percent Identity ___ to ___ | E value ___ to ___ | Query Coverage ___ to ___

Filter | Reset

No significant similarity found. For reasons why, click here

D

	Description	Scientific Name	Max Score	Total Score	Query Cover	E value	Per. Ident	Acc. Len	Accession
☑	Nocardioides okcheonensis strain MMS20-HV4-12 chromosome, complete genome	Nocardioides okcheo...	911	911	100%	0.0	79.20%	4471533	CP087710.1
☑	Rhodococcus qingshengii strain VT6 plasmid pLP1, complete sequence	Rhodococcus qingsh...	425	425	52%	6e-114	77.66%	501672	CP088909.1
☑	Cnuibacter physcomitrellae strain XA(T), complete genome	Cnuibacter physcomit...	407	407	50%	2e-108	77.73%	4061502	CP020715.1
☑	Microbacterium sp. A18JL241 chromosome, complete genome	Microbacterium sp. A...	337	337	52%	3e-87	75.62%	3267287	CP063815.1
☑	Aquibium microcysteis strain NIBR3 chromosome, complete genome	Aquibium microcysteis	231	231	68%	1e-55	72.01%	6109263	CP061080.1

F

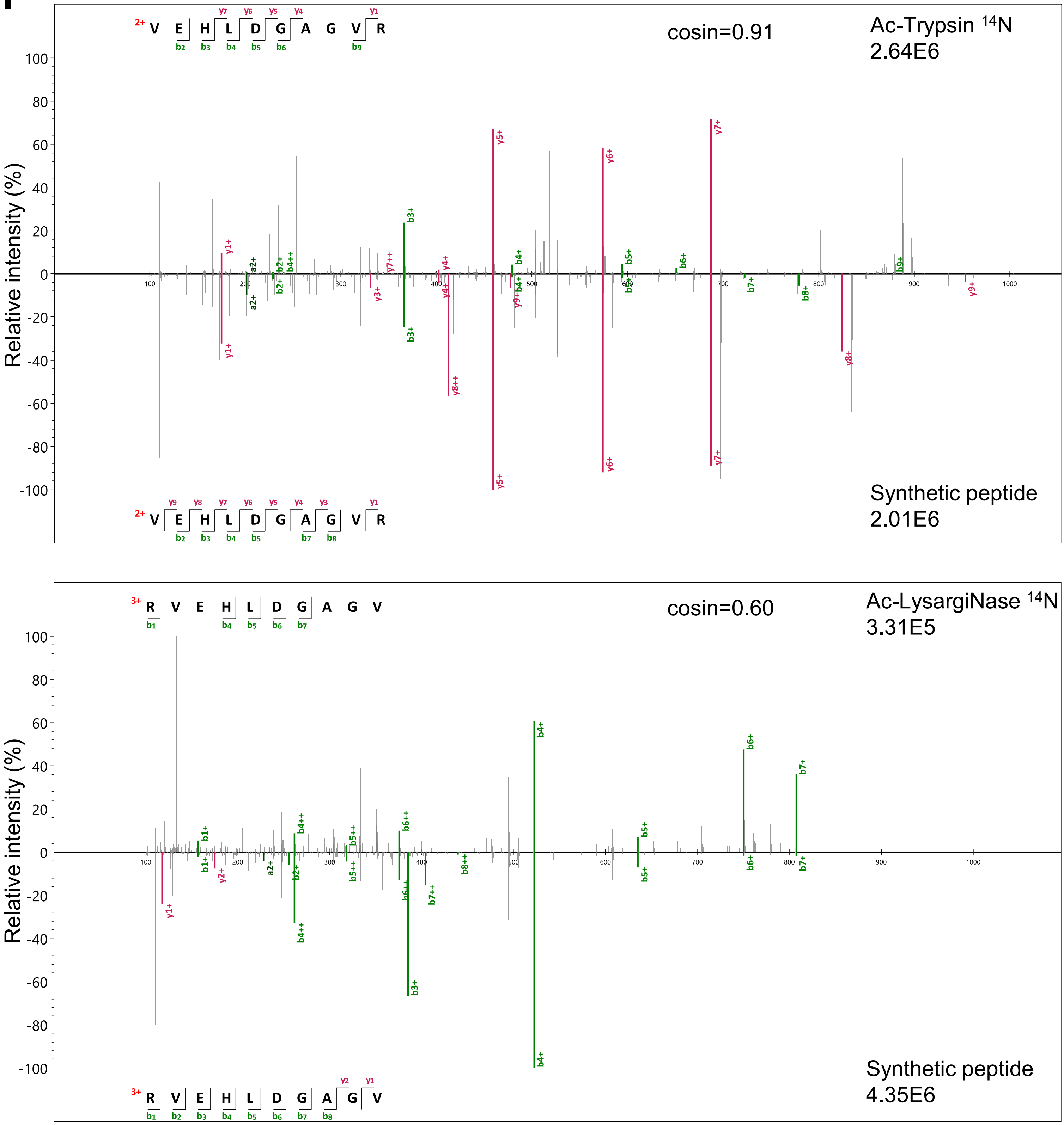

2+ V E H L D G A G V R
cosin=0.91
Ac-Trypsin 14N
2.64E6
Relative intensity (%)
Synthetic peptide
2.01E6
3+ R V E H L D G A G V
cosin=0.60
Ac-LysargiNase 14N
3.31E5
Relative intensity (%)
Synthetic peptide
4.35E6

No. 3 orf|0|+|4368726-4369110|

A

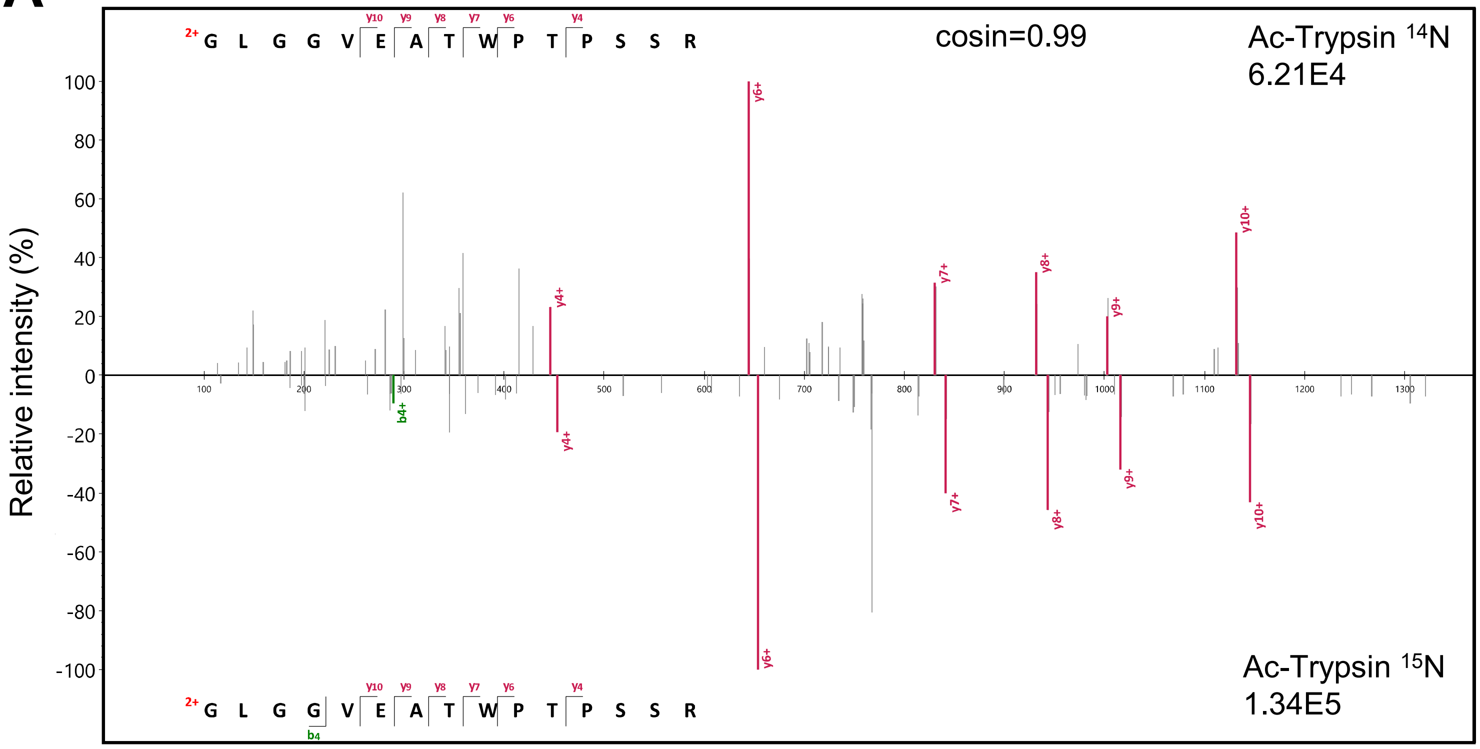

B

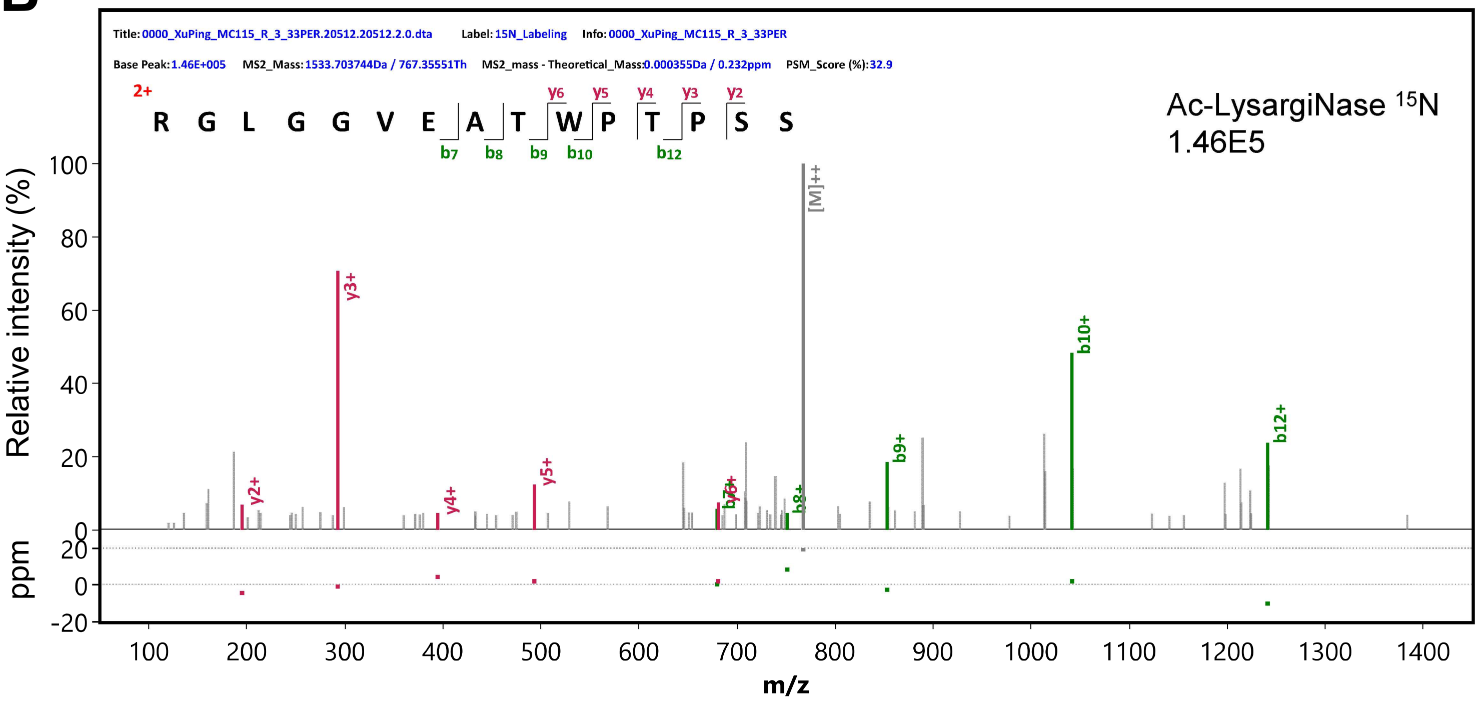

C

< Edit Search | Save Search | Search Summary | How to read this report? | BLAST Help Videos | Back to Traditional Results Page

Job Title	orf|0|+|4368726-4369110|
RID	61KM82SS013 *Search expires on 04-22 11:24 am* Download All
Program	Citation
Database	nr See details
Query ID	lcl|Query_53675
Description	orf|0|+|4368726-4369110|
Molecule type	amino acid
Query Length	128
Other reports	

Filter Results

Percent Identity ___ to ___ E value ___ to ___ Query Coverage ___ to ___

Filter Reset

No significant similarity found. For reasons why, click here

D

	Description	Scientific Name	Max Score	Total Score	Query Cover	E value	Per. Ident	Acc. Len	Accession
☑	Mycobacterium goodii strain X7B, complete genome	Mycobacterium ...	555	555	99%	2e-153	92.95%	7105933	CP012150.1
☑	Mycobacterium goodii strain ATCC 700504 chromosome, complete genome	Mycobacterium ...	547	547	100%	3e-151	92.47%	6741281	CP092364.1
☑	Mycolicibacterium phocaicum JCM 15301 DNA, nearly complete genome	Mycolicibacteriu...	416	416	74%	1e-111	92.98%	5853197	AP022616.1
☑	Mycolicibacterium phocaicum strain RL-HY01 chromosome, complete genome	Mycolicibacteriu...	416	416	74%	1e-111	92.98%	6064759	CP084713.1
☑	Mycolicibacterium aubagnense JCM 15296 DNA, complete genome	Mycolicibacteriu...	412	412	76%	1e-110	91.86%	6055481	AP022577.1
☑	Mycolicibacterium sp. TY81 DNA, complete genome	Mycolicibacteriu...	411	411	74%	5e-110	92.63%	6140486	AP023362.1
☑	Mycolicibacterium sp. TY66 DNA, complete genome	Mycolicibacteriu...	411	411	74%	5e-110	92.63%	6140532	AP023333.1
☑	Mycolicibacterium mucogenicum DSM 44124 chromosome, complete genome	Mycolicibacteriu...	411	411	74%	5e-110	92.63%	6099273	CP062008.1
☑	Mycobacterium rutilum strain DSM 45405 genome assembly, chromosome: I	Mycolicibacteriu...	403	403	75%	8e-108	91.72%	5987931	LT629971.1
☑	Mycolicibacterium neoaurum strain MN2019 chromosome, complete genome	Mycolicibacteriu...	401	401	76%	3e-107	91.22%	5756799	CP074376.1

No. 4 orf|0|+|4634811-4635201|

A

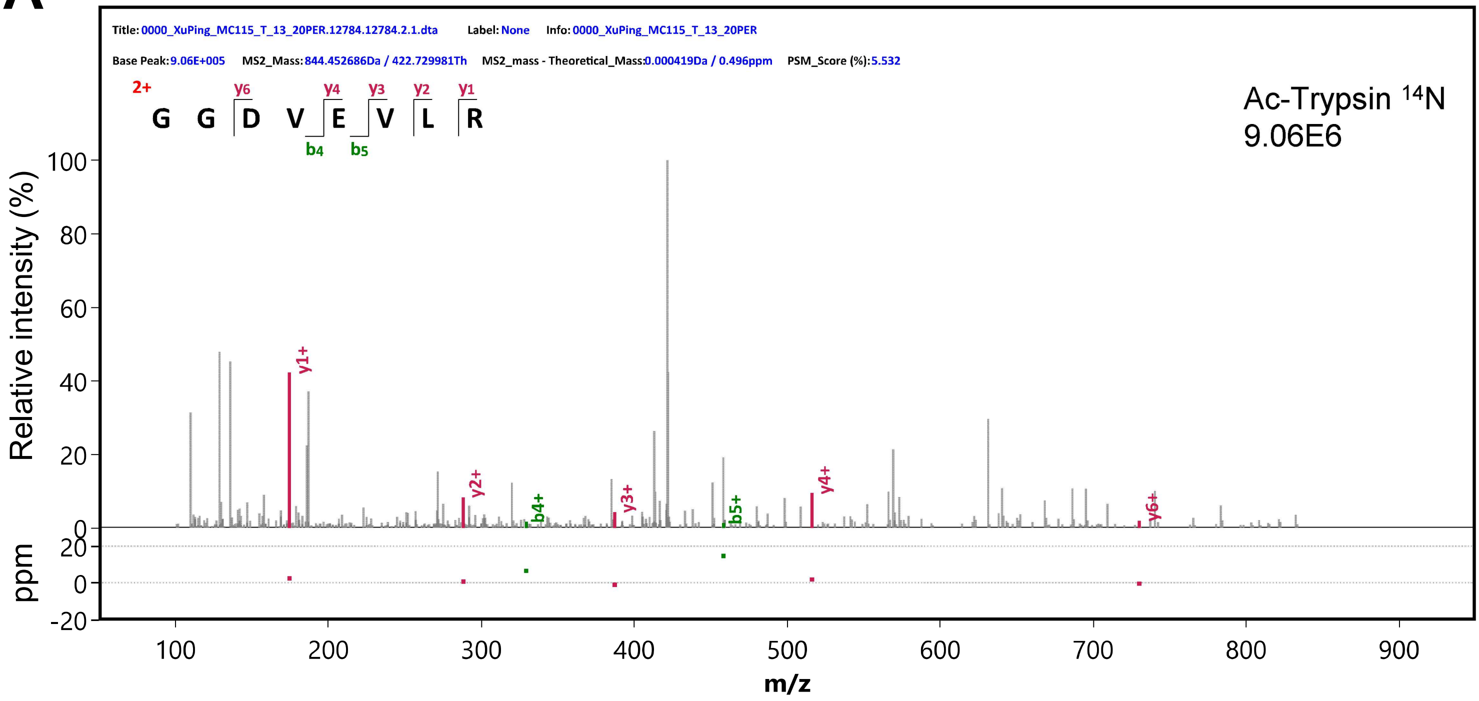

B

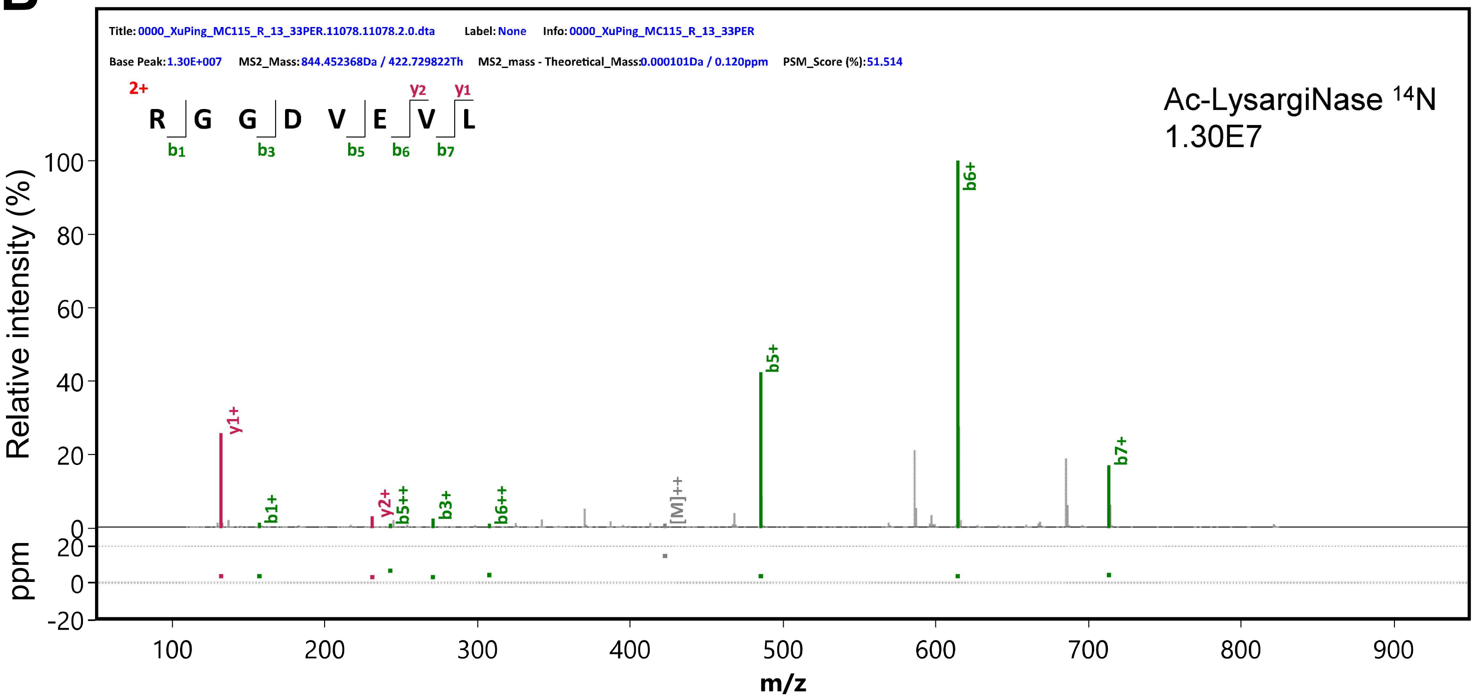

C

< Edit Search | Save Search | Search Summary | How to read this report? | BLAST Help Videos | Back to Traditional Results Page

**Job Title**	**orf|0|+|4634811-4635201|**
**RID**	61KSPTM6016 *Search expires on 04-22 11:27 am* Download All
**Program**	Citation
**Database**	nr See details
**Query ID**	lcl|Query_71180
**Description**	orf|0|+|4634811-4635201|
**Molecule type**	amino acid
**Query Length**	130
**Other reports**	

Filter Results

Percent Identity to | **E value** to | **Query Coverage** to

Filter | Reset

No significant similarity found. For reasons why, click here

D

	Description	Scientific Name	Max Score	Total Score	Query Cover	E value	Per. Ident	Acc. Len	Accession
☑	Mycobacterium goodii strain ATCC 700504 chromosome, complete genome	Mycobacterium goodii	580	580	99%	3e-161	93.57%	6741281	CP092364.1
☑	Mycobacterium goodii strain X7B, complete genome	Mycobacterium goodii	579	579	100%	1e-160	93.35%	7105933	CP012150.1
☑	Mycolicibacterium mageritense JCM 12375 DNA, complete genome	Mycolicibacterium mageritense	403	403	99%	8e-108	85.35%	8006721	AP022567.1
☑	Amycolatopsis mediterranei RB, complete genome	Amycolatopsis mediterranei RB	52.8	52.8	7%	0.032	100.00%	10246864	CP003777.1
☑	Amycolatopsis mediterranei S699, complete genome	Amycolatopsis mediterranei S699	52.8	52.8	7%	0.032	100.00%	10246920	CP003729.1
☑	Amycolatopsis mediterranei S699, complete genome	Amycolatopsis mediterranei S699	52.8	52.8	7%	0.032	100.00%	10236779	CP002896.1
☑	Amycolatopsis mediterranei U32, complete genome	Amycolatopsis mediterranei U32	52.8	52.8	7%	0.032	100.00%	10236715	CP002000.1

F

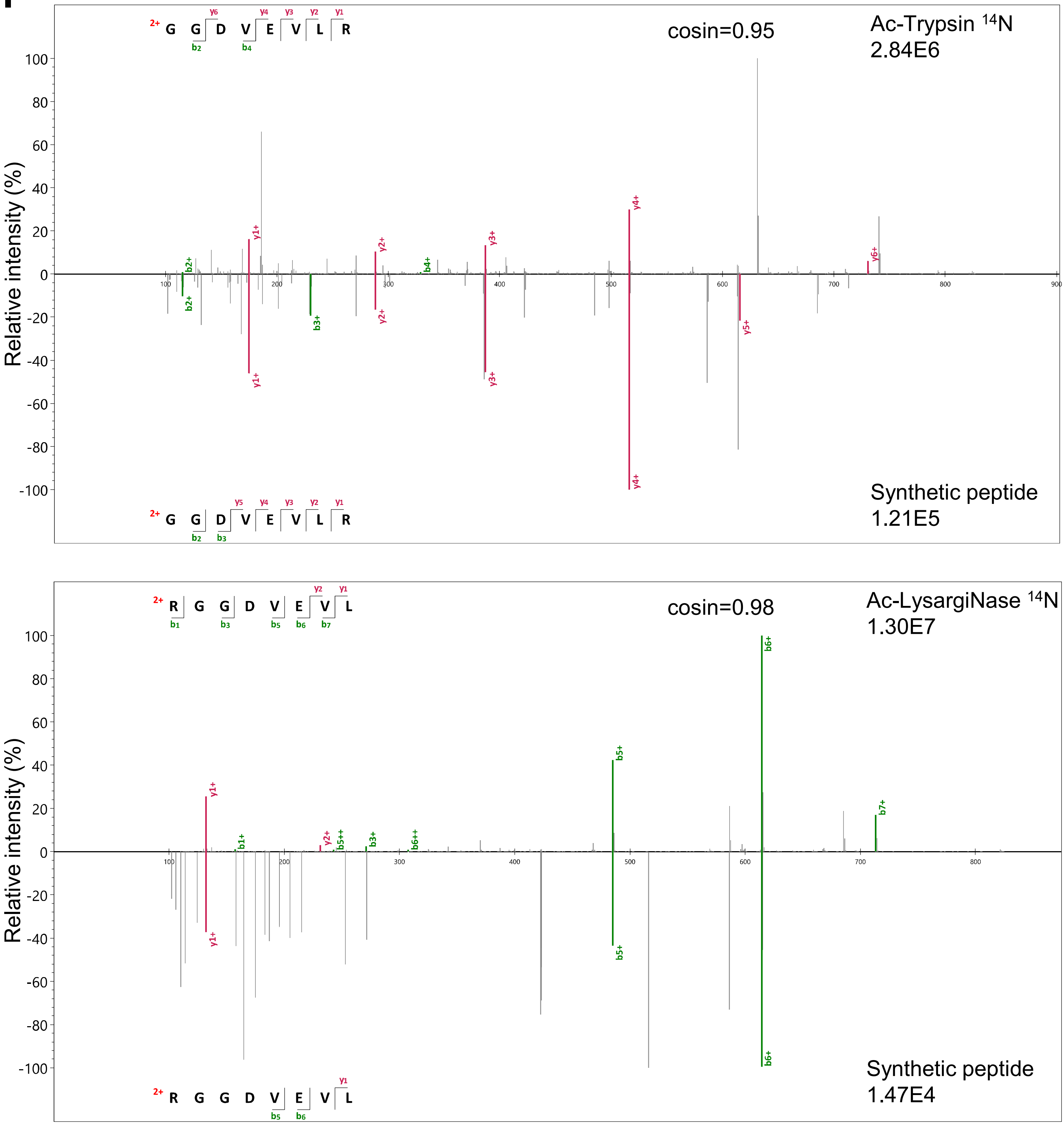
2+ G G D V E V L R
y6 y4 y3 y2 y1
b2 b4
cosin=0.95
Ac-Trypsin 14N
2.84E6
Relative intensity (%)
100
80
60
40
20
0
-20
-40
-60
-80
-100
y1+ y2+ y3+ y4+ y5+ y6+ b2+ b3+ b4+
100 200 300 400 500 600 700 800 900
2+ G G D V E V L R
y5 y4 y3 y2 y1
b2 b3
Synthetic peptide
1.21E5
2+ R G G D V E V L
y2 y1
b1 b3 b5 b6 b7
cosin=0.98
Ac-LysargiNase 14N
1.30E7
Relative intensity (%)
y1+ y2+ b1+ b3+ b5+ b5++ b6+ b6++ b7+
100 200 300 400 500 600 700 800
2+ R G G D V E V L
y1
b5 b6
Synthetic peptide
1.47E4

No. 5 orf|0|+|5658318-5659182|

A

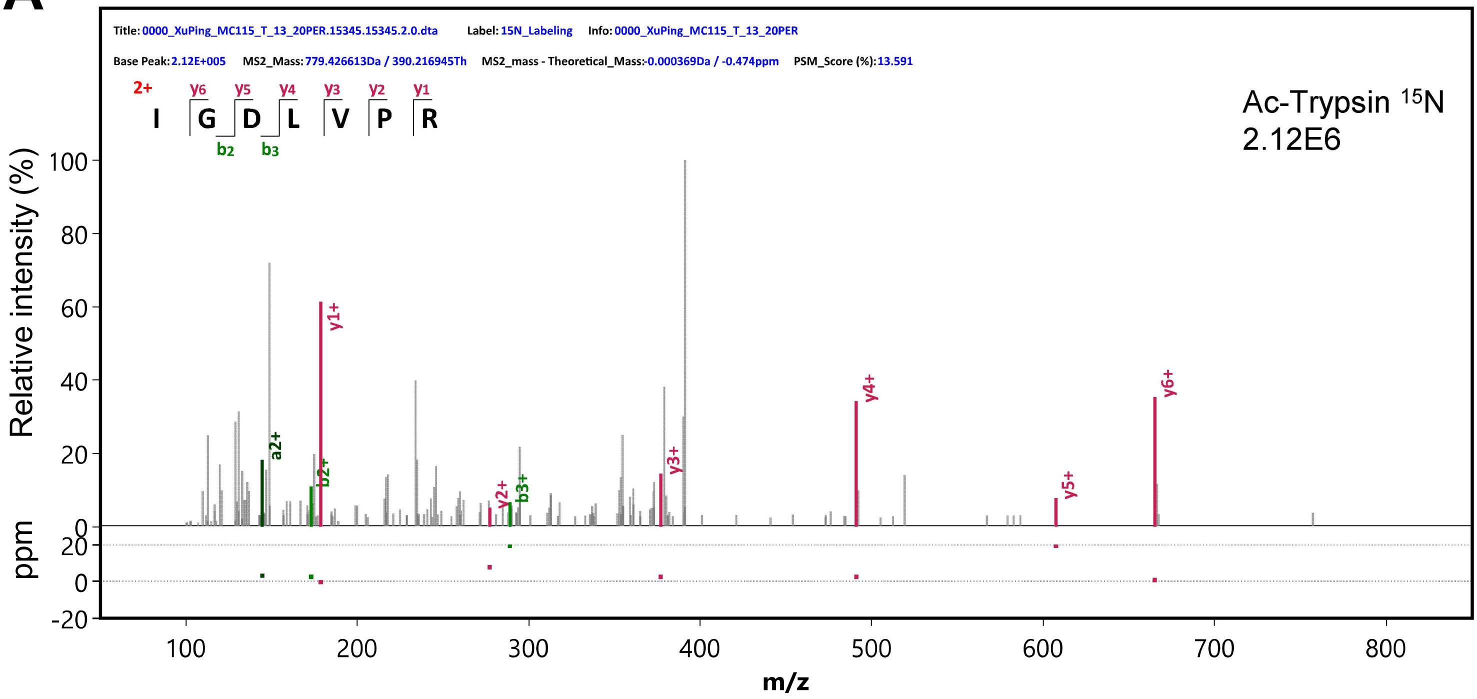

B

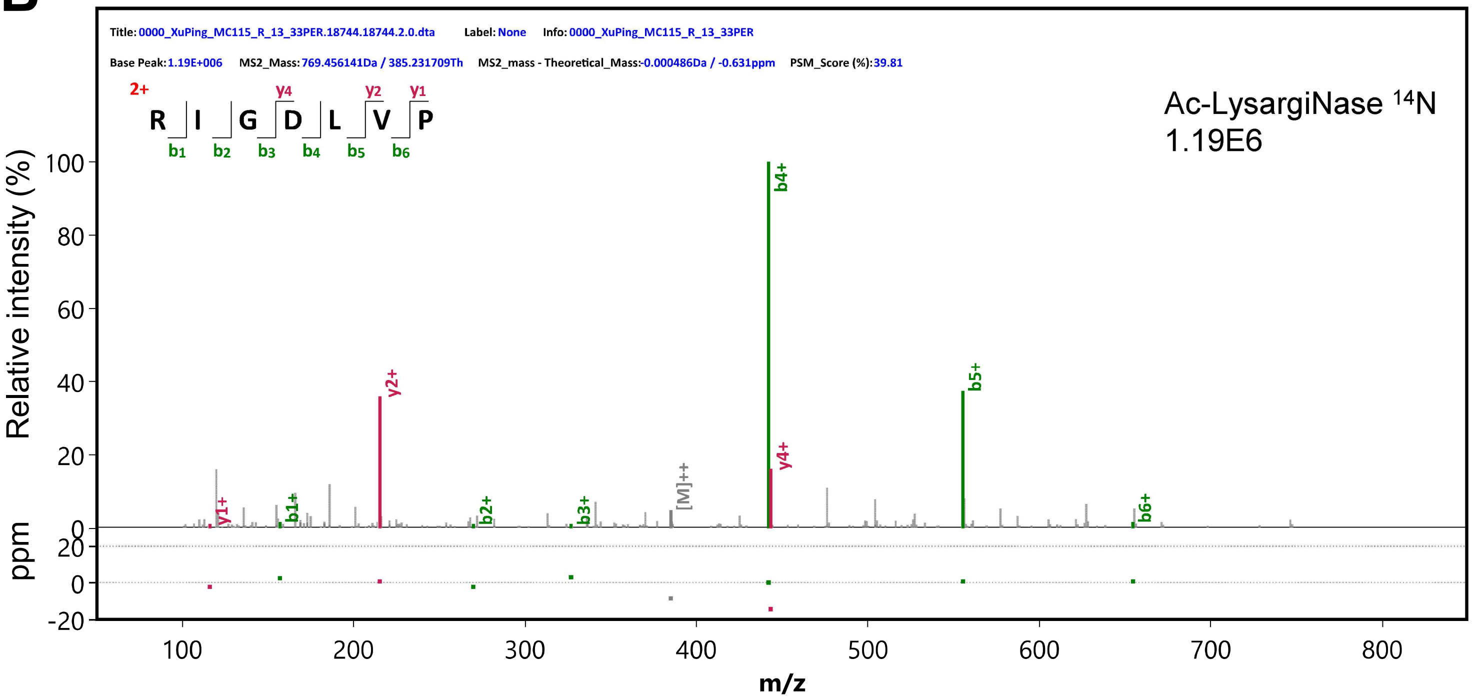

C

	Description	Scientific Name	Max Score	Total Score	Query Cover	E value	Per. Ident	Acc. Len	Accession
☑	hypothetical protein SRABI128_01274 [Microbacterium sp. Bi128]	Microbacterium sp. Bi128	77.8	77.8	57%	8e-12	36.31%	509	CAH0180998.1
☑	hypothetical protein SRABI26_04415 [Arthrobacter sp. Bi26]	Arthrobacter sp. Bi26	50.4	50.4	35%	0.009	40.20%	530	CAH0297134.1

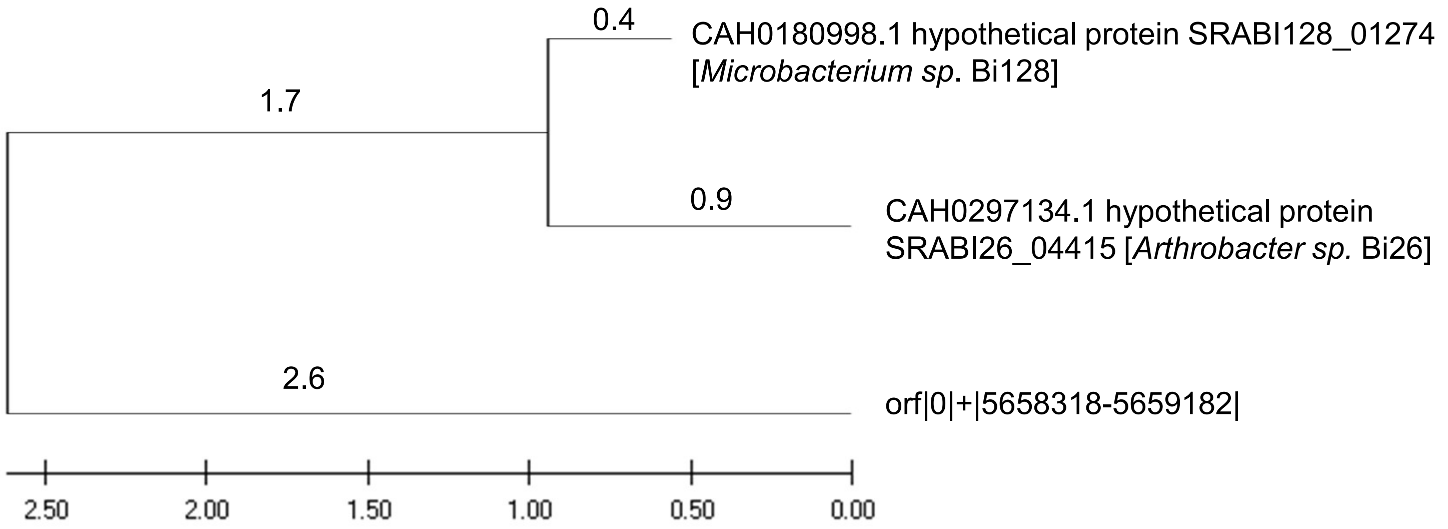

D

	Description	Scientific Name	Max Score	Total Score	Query Cover	E value	Per. Ident	Acc. Len	Accession
☑	Mycobacterium goodii strain X7B, complete genome	Mycobacterium ...	1242	1242	99%	0.0	92.59%	7105933	CP012150.1
☑	Mycobacterium goodii strain ATCC 700504 chromosome, complete genome	Mycobacterium ...	1175	1175	99%	0.0	91.10%	6741281	CP092364.1
☑	Mycolicibacterium fortuitum strain W6 chromosome	Mycolicibacteriu...	769	769	92%	0.0	84.03%	6555851	CP060410.1
☑	Mycolicibacterium fortuitum strain W4 chromosome	Mycolicibacteriu...	763	763	92%	0.0	83.98%	6674224	CP060409.1
☑	Mycolicibacterium mageritense JCM 12375 DNA, complete genome	Mycolicibacteriu...	760	760	93%	0.0	83.66%	8006721	AP022567.1
☑	Mycolicibacterium boenickei JCM 15653 DNA, complete genome	Mycolicibacteriu...	756	756	92%	0.0	83.79%	6563937	AP022579.1
☑	Mycolicibacterium fortuitum strain Kelantanesensis chromosome, complete genome	Mycolicibacteriu...	747	747	92%	0.0	83.52%	6419378	CP089608.1
☑	Mycobacterium sp. DL440 chromosome	Mycobacterium ...	745	745	92%	0.0	83.44%	6381854	CP050191.1
☑	Mycobacterium fortuitum subsp. fortuitum DSM 46621 = ATCC 6841 genome	Mycolicibacteriu...	736	736	92%	0.0	83.33%	6257075	CP014258.1
☑	Mycobacterium fortuitum strain CT6, complete genome	Mycolicibacteriu...	736	736	92%	0.0	83.33%	6254616	CP011269.1

No. 6 orf|0|-|6584055-6584880|

A

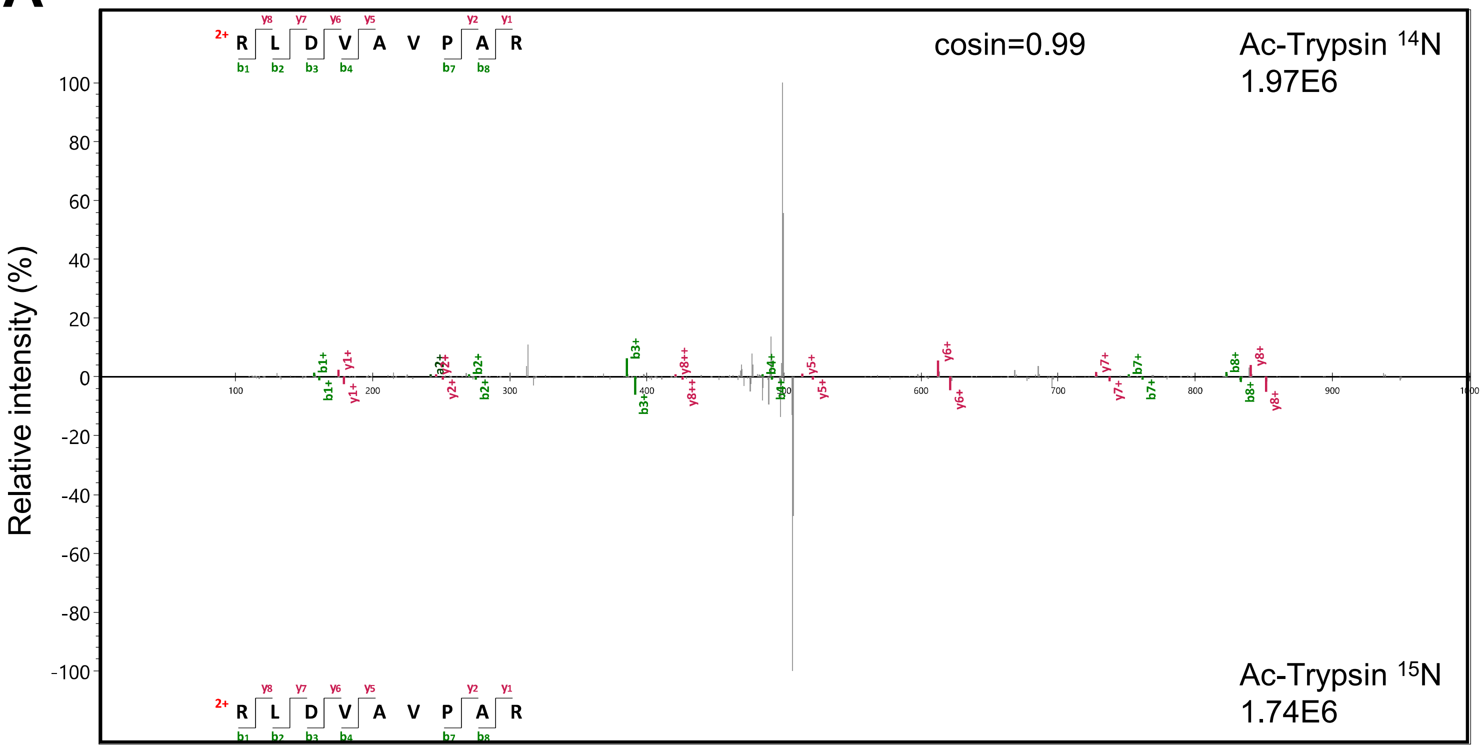

B

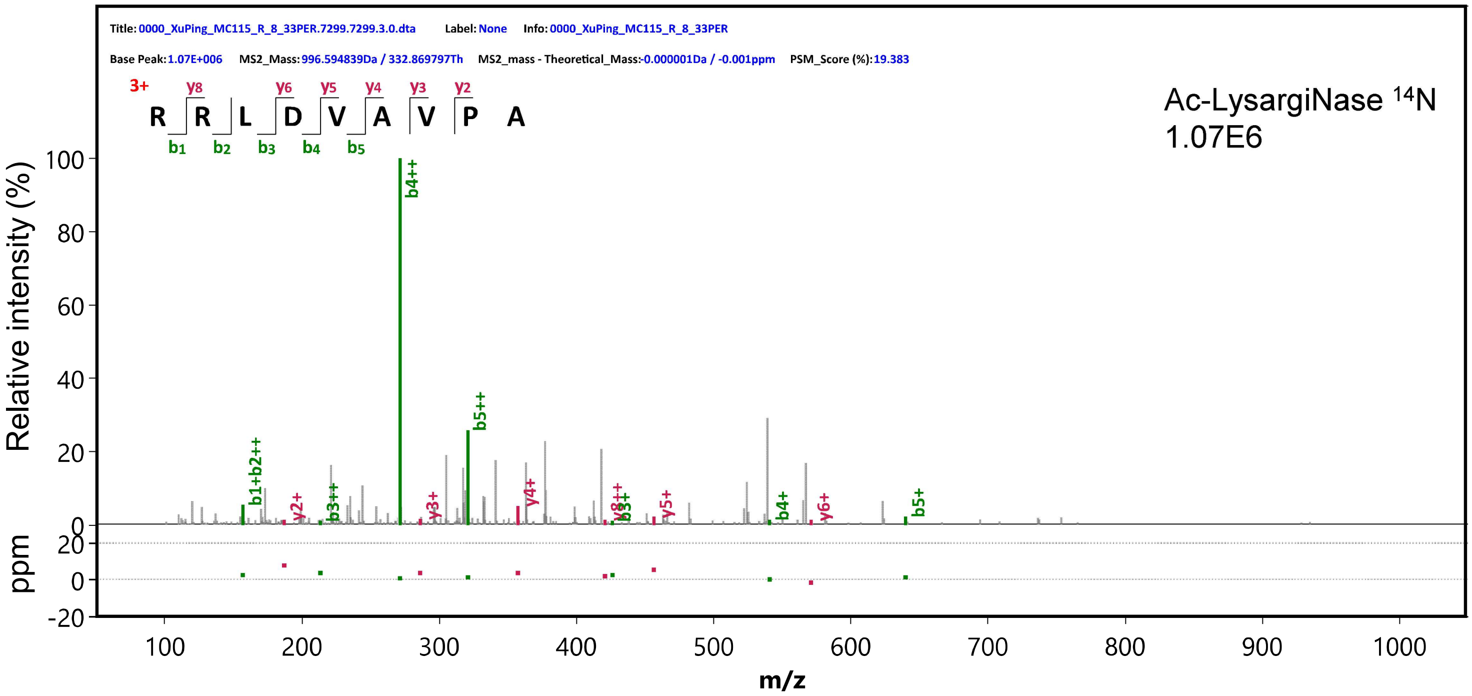

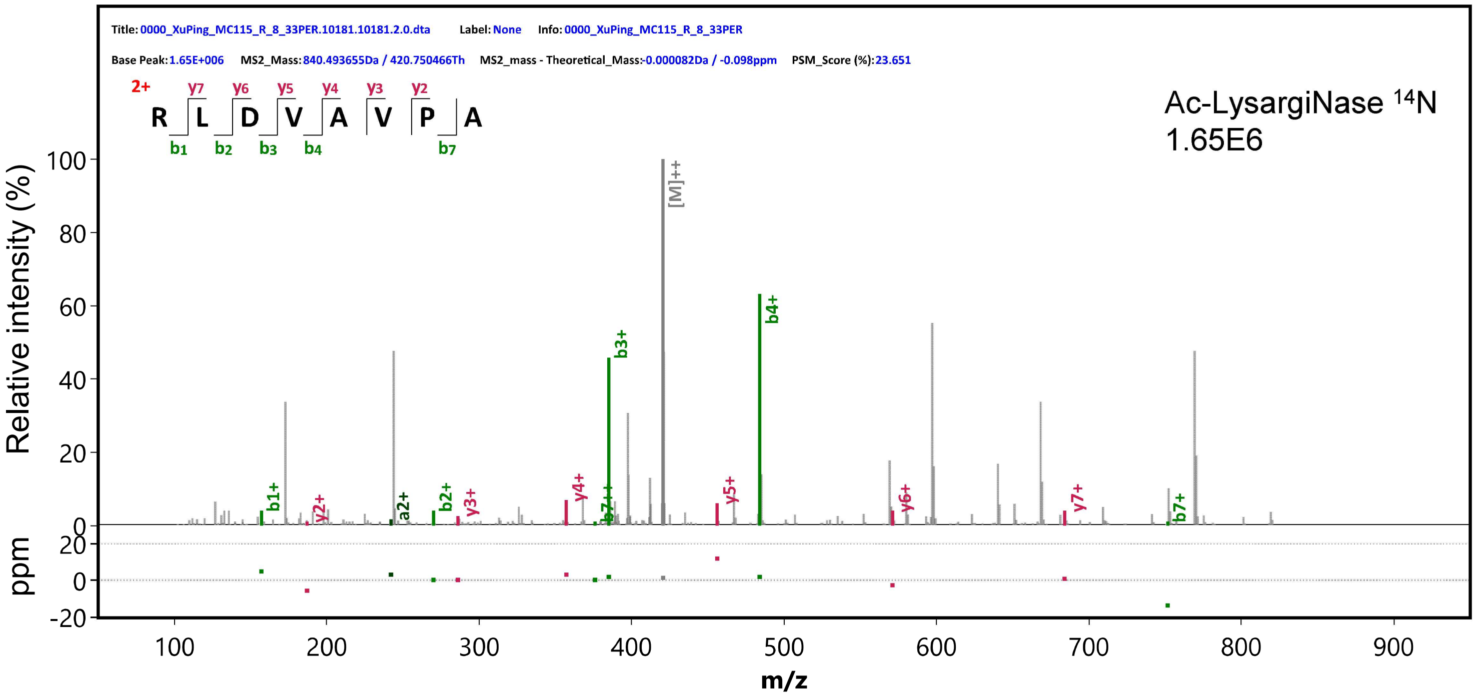

C

Job Title	orf|0|-|6584055-6584880|
RID	61Y17CFH016 *Search expires on 04-22 14:22 pm* Download All
Program	Citation
Database	nr See details
Query ID	lcl|Query_51623
Description	orf|0|-|6584055-6584880|
Molecule type	amino acid
Query Length	275
Other reports	

‹ Edit Search | Save Search | Search Summary | How to read this report? | BLAST Help Videos | Back to Traditional Results Page

Filter Results

Percent Identity ___ to ___ | E value ___ to ___ | Query Coverage ___ to ___

Filter | Reset

⚠ No significant similarity found. For reasons why, click here

D

	Description	Scientific Name	Max Score	Total Score	Query Cover	E value	Per. Ident	Acc. Len	Accession
☑	Mycobacterium goodii strain X7B, complete genome	Mycobacterium goodii	1179	1179	99%	0.0	92.69%	7105933	CP012150.1
☑	Mycobacterium goodii strain ATCC 700504 chromosome, complete genome	Mycobacterium goodii	1062	1062	99%	0.0	90.15%	6741281	CP092364.1
☑	Mycolicibacterium mageritense JCM 12375 DNA, complete genome	Mycolicibacterium mageritense	780	780	98%	0.0	84.17%	8006721	AP022567.1
☑	Mycobacterium sp. DL440 chromosome	Mycobacterium sp. DL440	745	745	95%	0.0	83.87%	6381854	CP050191.1
☑	Mycobacterium fortuitum strain CT6, complete genome	Mycolicibacterium fortuitum	741	741	95%	0.0	83.83%	6254616	CP011269.1
☑	Mycolicibacterium fortuitum strain W6 chromosome	Mycolicibacterium fortuitum	736	736	94%	0.0	83.90%	6555851	CP060410.1
☑	Mycobacterium sp. VKM Ac-1817D, complete genome	Mycobacterium sp. VKM Ac-1817D	730	730	95%	0.0	83.58%	6324222	CP009914.1
☑	Mycolicibacterium fortuitum strain Kelantanesensis chromosome, complete genome	Mycolicibacterium fortuitum	730	730	95%	0.0	83.58%	6419378	CP089608.1
☑	Mycolicibacterium fortuitum subsp. fortuitum JCM6387 DNA, complete genome	Mycolicibacterium fortuitum subsp. fortuitum	725	725	95%	0.0	83.46%	6406072	AP025518.1
☑	Mycolicibacterium fortuitum strain W4 chromosome	Mycolicibacterium fortuitum	725	725	95%	0.0	83.46%	6674224	CP060409.1

No. 7 orf|0|+|124501-127159|

A

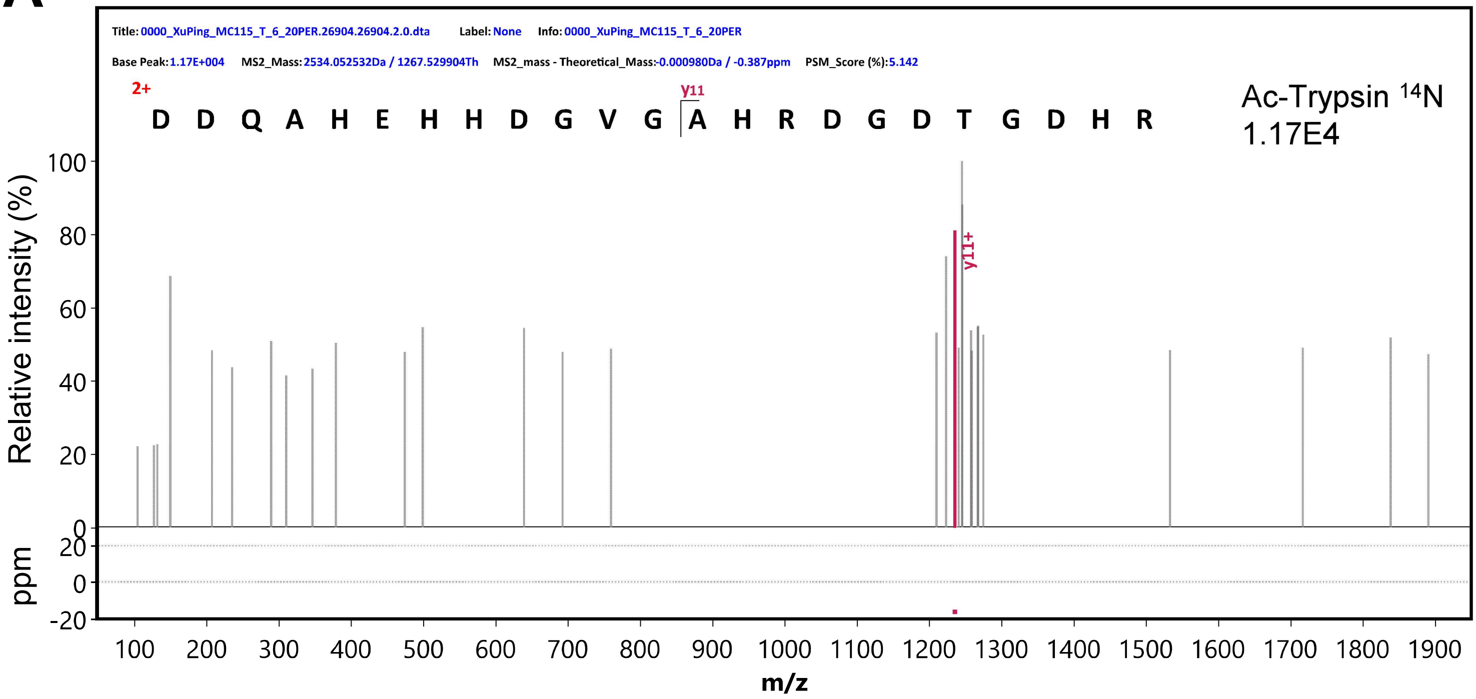

B

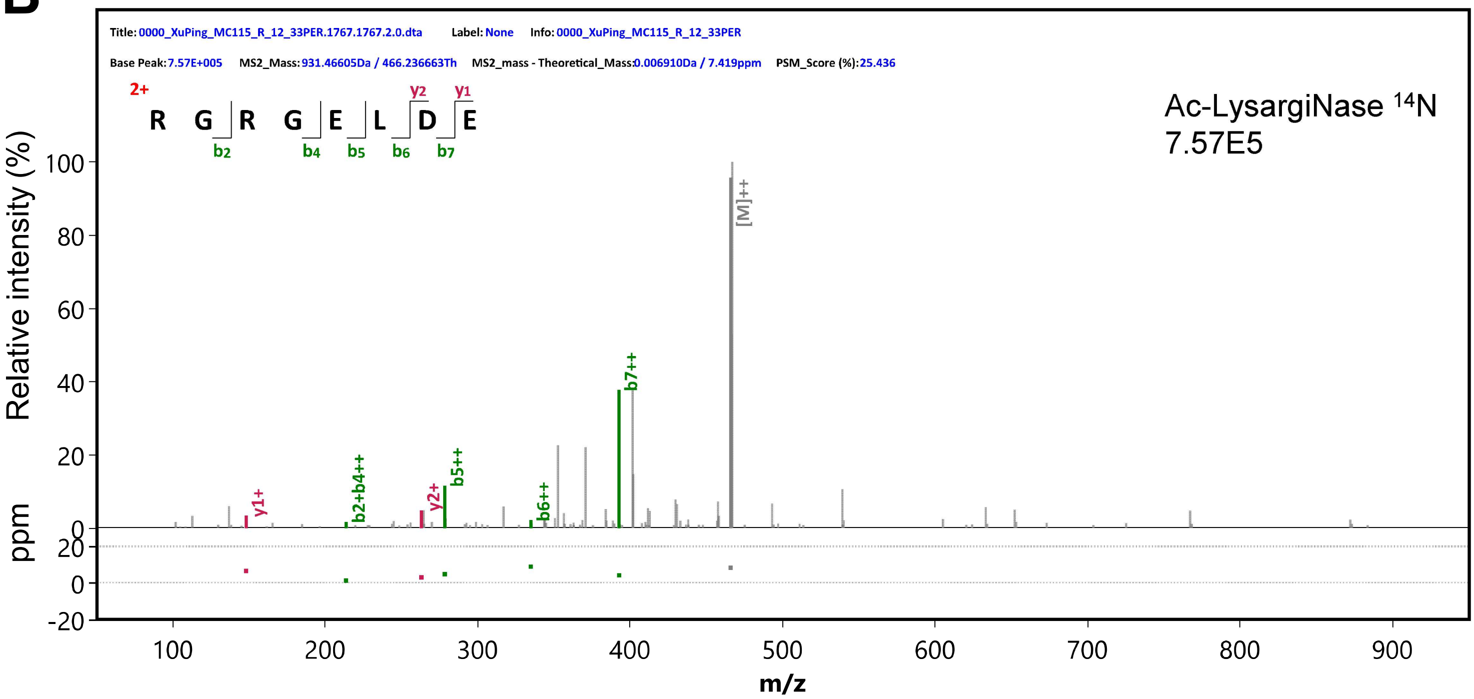

C

	Description	Scientific Name	Max Score	Total Score	Query Cover	E value	Per. Ident	Acc. Len	Accession
☑	hypothetical protein PAERUG_P39_London_29_08_12_01179 [Pseudomonas a...	Pseudomonas aeruginosa	208	208	40%	1e-54	50.14%	465	CRN70776.1
☑	hypothetical protein PSEUDO8BK_30768 [Pseudomonas sp. 8BK]	Pseudomonas sp. 8BK	173	173	38%	7e-43	43.86%	413	VXB60568.1
☑	hypothetical protein PSEUDO9AZ_20004 [Pseudomonas sp. 9AZ]	Pseudomonas sp. 9AZ	172	172	45%	4e-42	38.80%	449	VXC79955.1
☑	regulatory protein, TetR [Phenylobacterium zucineum HLK1]	Phenylobacterium zucineum HLK1	167	167	36%	2e-39	50.46%	597	ACG79582.1
☑	Uncharacterised protein [Acinetobacter baumannii]	Acinetobacter baumannii	140	140	24%	1e-32	54.55%	300	SST13014.1
☑	Uncharacterised protein [Mycobacterium tuberculosis]	Mycobacterium tuberculosis	133	133	18%	8e-32	64.42%	166	COZ55393.1
☑	putative regulatory protein TetR [uncultured bacterium CBNPD1 BAC clone 1664]	uncultured bacterium CBNPD1 BAC clone 1664	135	135	38%	1e-29	43.15%	428	ABM53578.1
☑	regulatory protein TetR [Rhodococcus triatomae BKS 15-14]	Rhodococcus triatomae BKS 15-14	108	108	39%	3e-20	41.19%	611	EME15440.1
☑	hypothetical protein J113_01095 [Mycobacterium tuberculosis CAS/NITR204]	Mycobacterium tuberculosis CAS/NITR204	92.4	92.4	10%	3e-18	58.51%	96	AGL25616.1
☑	hypothetical protein ASE00_18685 [Sphingomonas sp. Root710]	Sphingomonas sp. Root710	89.0	89.0	19%	4e-16	43.43%	177	KRB79740.1
☑	Regulatory protein, TetR [Sulfitobacter mediterraneus KCTC 32188]	Sulfitobacter mediterraneus KCTC 32188	91.7	91.7	38%	1e-15	30.90%	348	KIN78935.1
☑	hypothetical protein PFLmoz3_05245 [Pseudomonas fluorescens]	Pseudomonas fluorescens	88.2	88.2	19%	5e-15	49.44%	262	KWV85097.1
☑	TonB-dependent receptor [Acidovorax sp. CCYZU-2555]	Acidovorax sp. CCYZU-2555	86.7	86.7	30%	3e-13	33.21%	601	WP_213293972.1
☑	hypothetical protein GCM10011610_30650 [Nocardia rhizosphaerihabitans]	Nocardia rhizosphaerihabitans	69.3	69.3	21%	1e-08	32.46%	270	GGN80869.1
☑	hypothetical protein BC938DRAFT_471649 [Jimgerdemannia flammicorona]	Jimgerdemannia flammicorona	67.8	67.8	16%	1e-07	42.28%	344	RUS35366.1
☑	acyl-CoA dehydrogenase domain protein [Burkholderia pseudomallei]	Burkholderia pseudomallei	56.2	56.2	10%	1e-04	50.53%	196	KGD50707.1

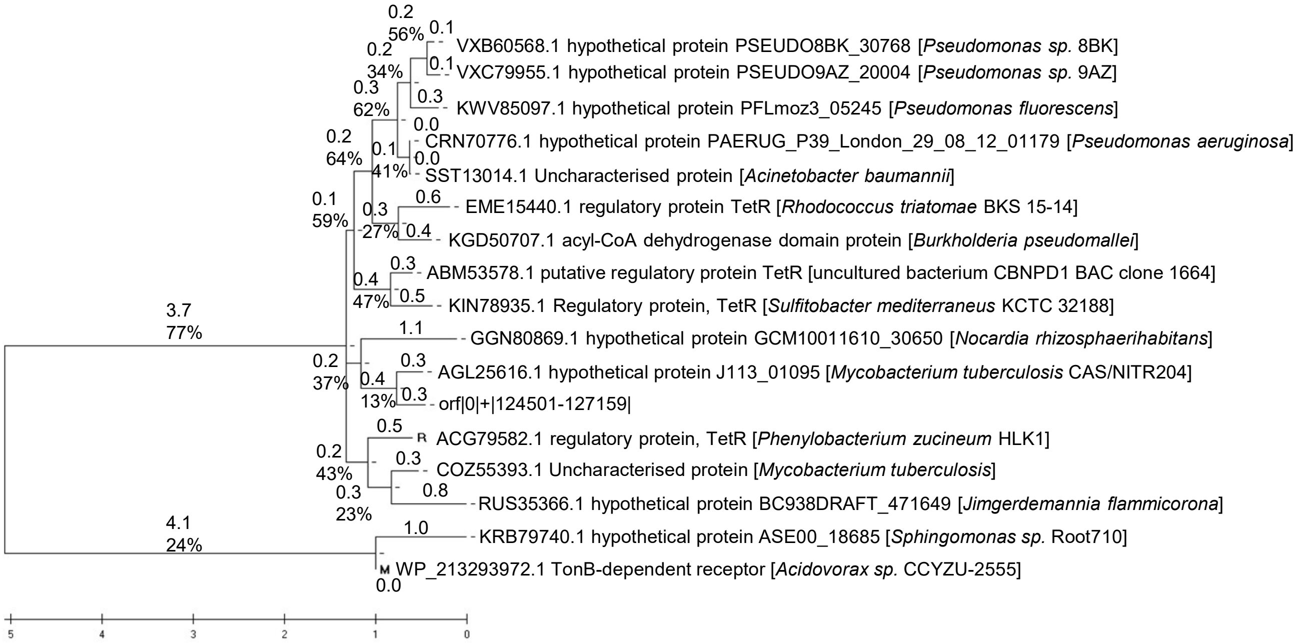

D

	Description	Scientific Name	Max Score	Total Score	Query Cover	E value	Per. Ident	Acc. Len	Accession
☑	Mycobacterium goodii strain ATCC 700504 chromosome, complete genome	Mycobacterium ...	3520	3520	100%	0.0	90.56%	6741281	CP092364.1
☑	Mycobacterium goodii strain X7B, complete genome	Mycobacterium ...	3371	3371	100%	0.0	89.55%	7105933	CP012150.1
☑	Mycobacterium aurum isolate liquid genome assembly, chromosome: I	Mycolicibacteriu...	2004	2004	98%	0.0	80.63%	6032389	LT899394.1
☑	Mycobacterium aurum isolate liquid genome assembly, chromosome: I	Mycolicibacteriu...	2004	2004	99%	0.0	80.61%	6038730	LT549889.1
☑	Mycobacterium diernhoferi strain ATCC 19340 chromosome, complete genome	Mycobacterium ...	1989	1989	90%	0.0	81.81%	5998503	CP080332.1
☑	Mycobacterium sp. YC-RL4, complete genome	Mycobacterium ...	1925	1925	95%	0.0	80.57%	5801417	CP015596.1
☑	Mycolicibacterium septicum strain PDNC012 chromosome	Mycolicibacteriu...	1491	1664	51%	0.0	86.45%	6515693	CP070349.1
☑	Mycolicibacterium farcinogenes strain BKK/CU-MFGFA-001 chromosome, complete genome	Mycolicibacteriu...	1485	2325	91%	0.0	88.77%	6344228	CP081673.1
☑	Mycolicibacterium farcinogenes strain BKK/CU-MFGLA-001 chromosome, complete genome	Mycolicibacteriu...	1480	2336	91%	0.0	88.69%	6276329	CP080510.1
☑	Mycolicibacterium nivoides strain DL90 chromosome	Mycolicibacteriu...	1478	2358	98%	0.0	86.12%	6905961	CP034072.1

E

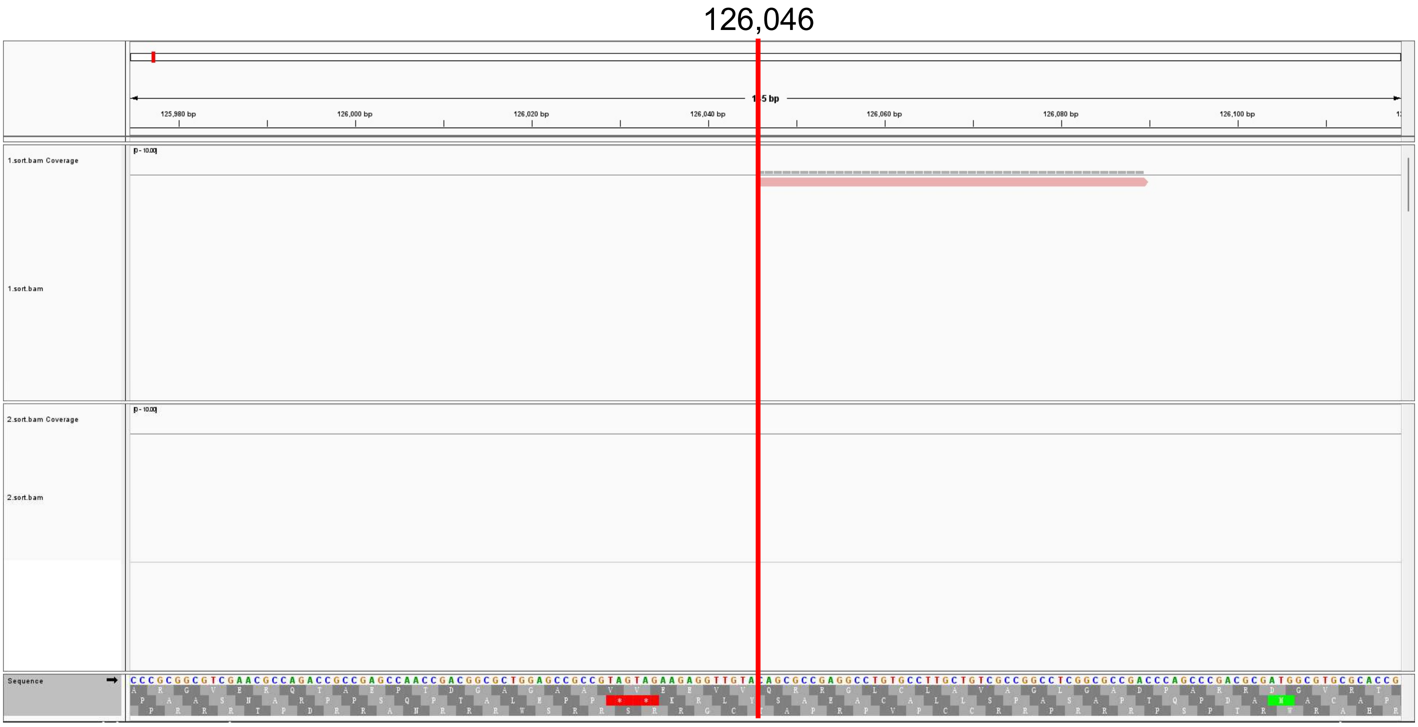
126,046
125,980 bp
126,000 bp
126,020 bp
126,040 bp
126,060 bp
126,080 bp
126,100 bp
1.sort.bam Coverage
1.sort.bam
2.sort.bam Coverage
2.sort.bam
Sequence

F

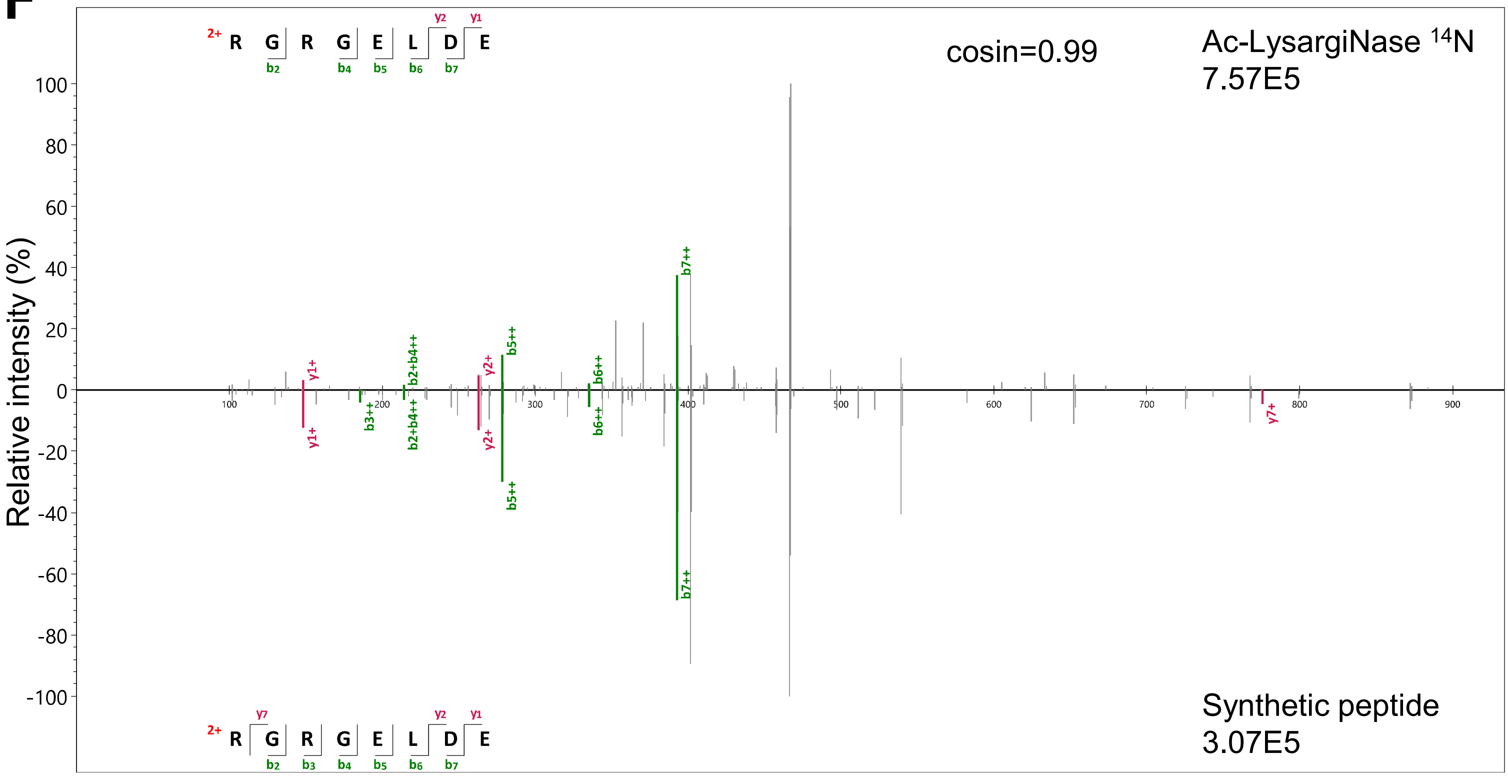

2+ R G R G E L D E
y2 y1
b2 b4 b5 b6 b7
cosin=0.99
Ac-LysargiNase ^{14}N
7.57E5
Relative intensity (%)
100
80
60
40
20
0
-20
-40
-60
-80
-100
y1+
b3++
b2+b4++
y2+
b5++
b6++
b7++
y7+
100
200
300
400
500
600
700
800
900
Synthetic peptide
3.07E5
2+ R G R G E L D E
y7 y2 y1
b2 b3 b4 b5 b6 b7

No. 8 orf|0|+|581123-582794|

A

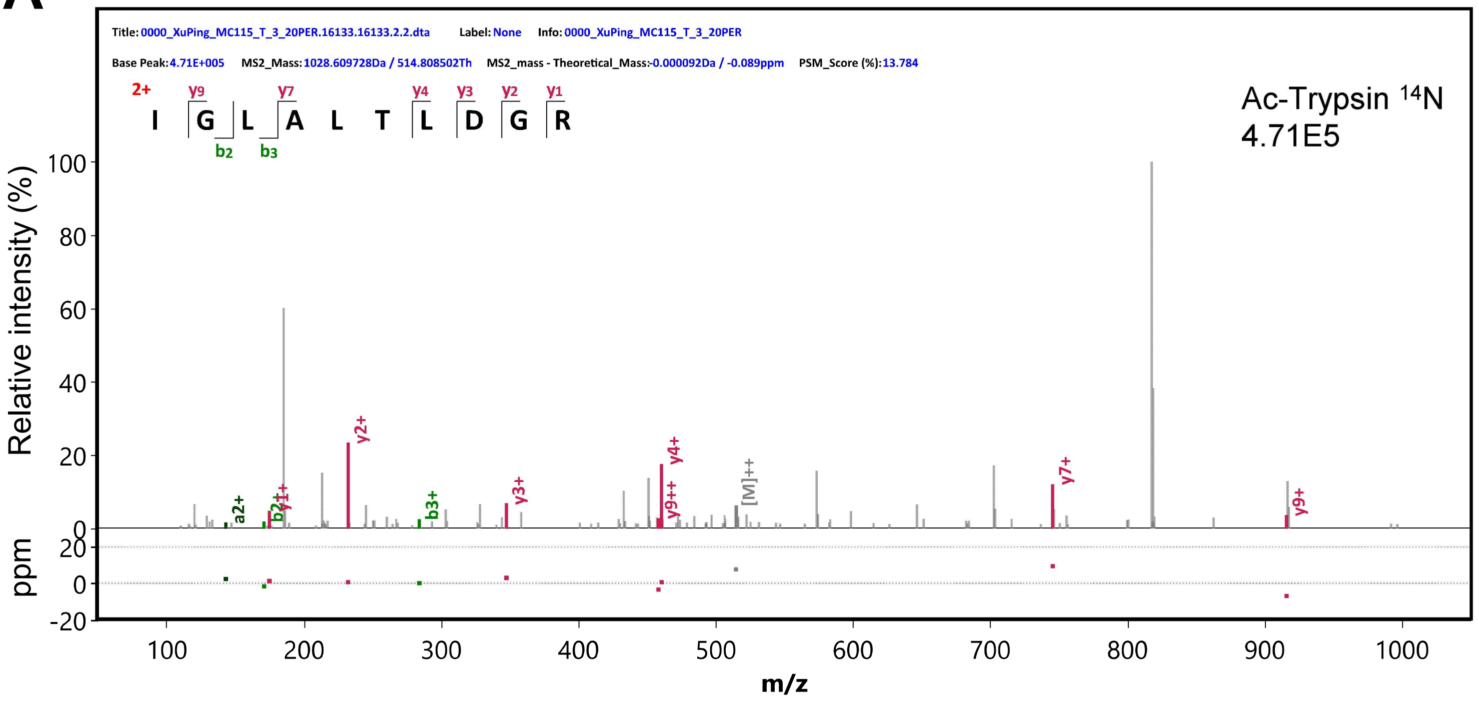

B

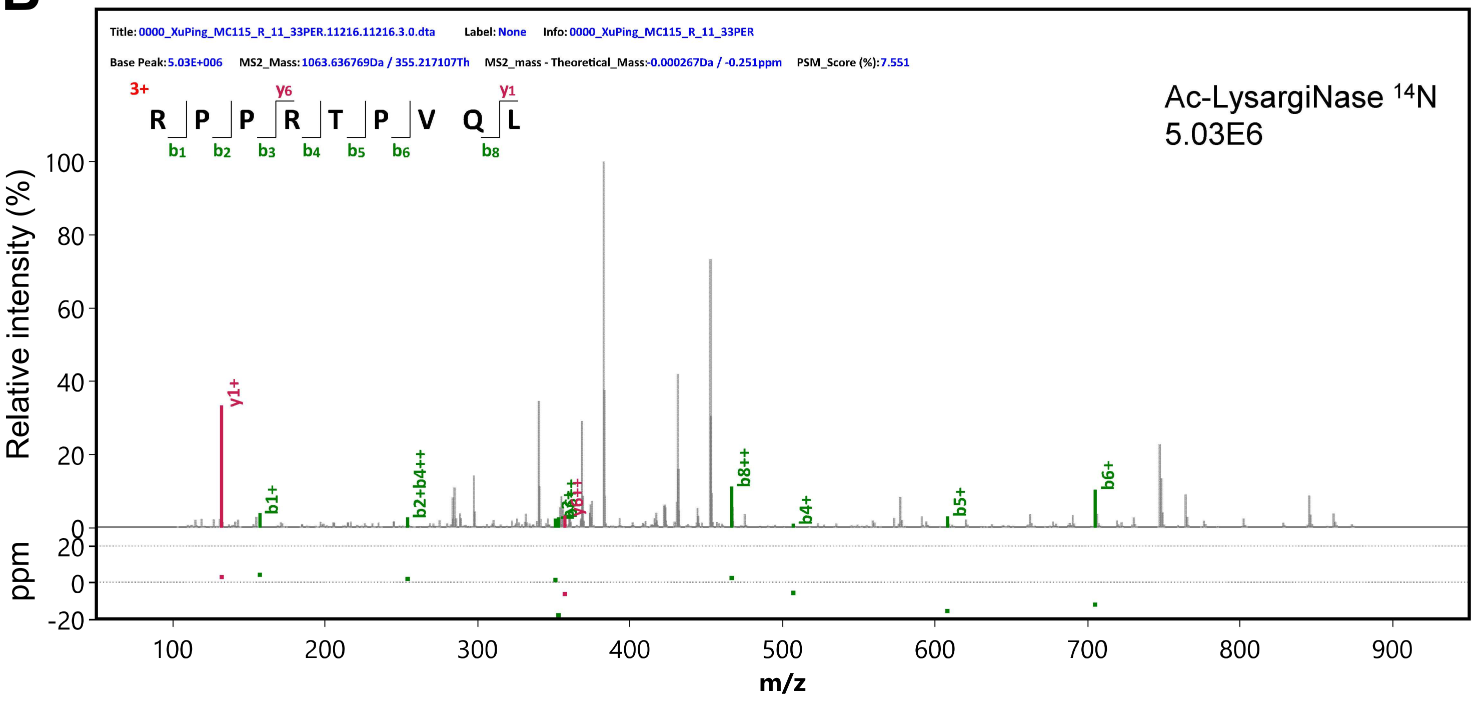

C

	Description	Scientific Name	Max Score	Total Score	Query Cover	E value	Per. Ident	Acc. Len	Accession
☑	hypothetical protein [Devosia sp. DBB001]	Devosia sp. DBB001	60.5	60.5	17%	7e-06	35.00%	281	CDP52652.1
☑	Uncharacterised protein [Acinetobacter baumannii]	Acinetobacter baumannii	58.9	58.9	14%	2e-05	44.30%	262	SVK52358.1

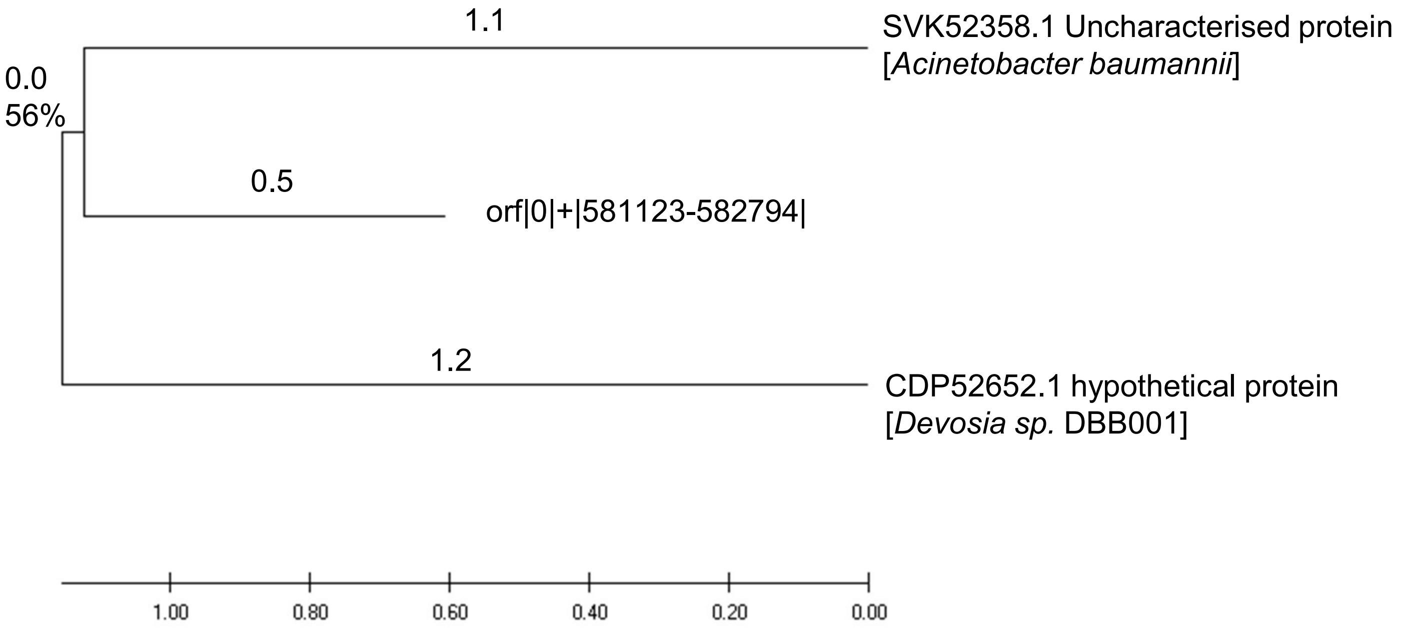

D

	Description	Scientific Name	Max Score	Total Score	Query Cover	E value	Per. Ident	Acc. Len	Accession
☑	Mycobacterium goodii strain X7B, complete genome	Mycobacterium goodii	2207	2207	100%	0.0	90.49%	7105933	CP012150.1
☑	Mycobacterium goodii strain ATCC 700504 chromosome, complete genome	Mycobacterium goodii	2010	2010	99%	0.0	88.39%	6741281	CP092364.1
☑	Mycolicibacterium fluoranthenivorans strain 2A chromosome	Mycolicibacterium fluoranthenivorans	1299	1299	99%	0.0	80.74%	6837082	CP059894.1
☑	Mycolicibacterium litorale strain F4 chromosome, complete genome	Mycolicibacterium litorale	1197	1197	99%	0.0	79.67%	6103712	CP019882.1
☑	Leifsonia sp. PS1209 chromosome, complete genome	Leifsonia sp. PS1209	573	573	92%	3e-158	73.87%	4091164	CP051154.1
☑	Streptomyces iranensis genome assembly Siranensis, scaffold SCAF00001	Streptomyces iranensis	545	545	98%	6e-150	73.34%	11956957	LK022848.1
☑	Leifsonia shinshuensis strain INR9 chromosome, complete genome	Leifsonia shinshuensis	529	529	89%	6e-145	73.64%	4438093	CP043641.1
☑	Streptomyces sp. M56	Streptomyces sp. M56	497	497	98%	2e-135	72.73%	11742376	CP025018.1
☑	Streptomyces malaysiensis strain DSM 4137 chromosome, complete genome	Streptomyces malaysiensis	492	492	98%	7e-134	72.70%	10670746	CP029823.1
☑	Streptomyces malaysiensis DSM4137 chromosome, complete sequence	Streptomyces malaysiensis	492	492	98%	7e-134	72.70%	10694678	CP023992.1

F

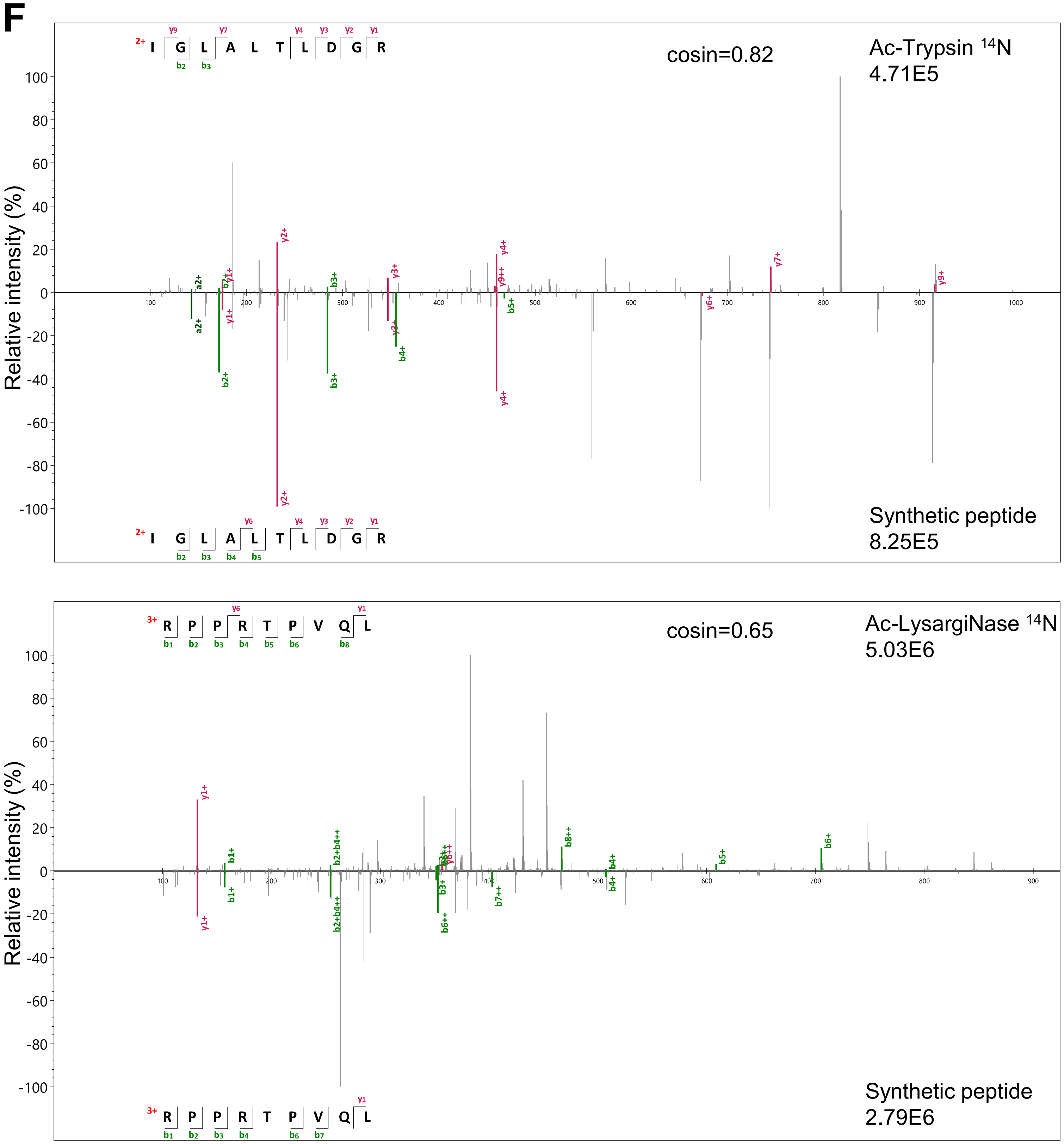
2+ I G L A L T L D G R
cosin=0.82
Ac-Trypsin ^{14}N
4.71E5
Relative intensity (%)
Synthetic peptide
8.25E5
3+ R P P R T P V Q L
cosin=0.65
Ac-LysargiNase ^{14}N
5.03E6
Relative intensity (%)
Synthetic peptide
2.79E6

No. 9 orf|0|+|813403-813805|

A

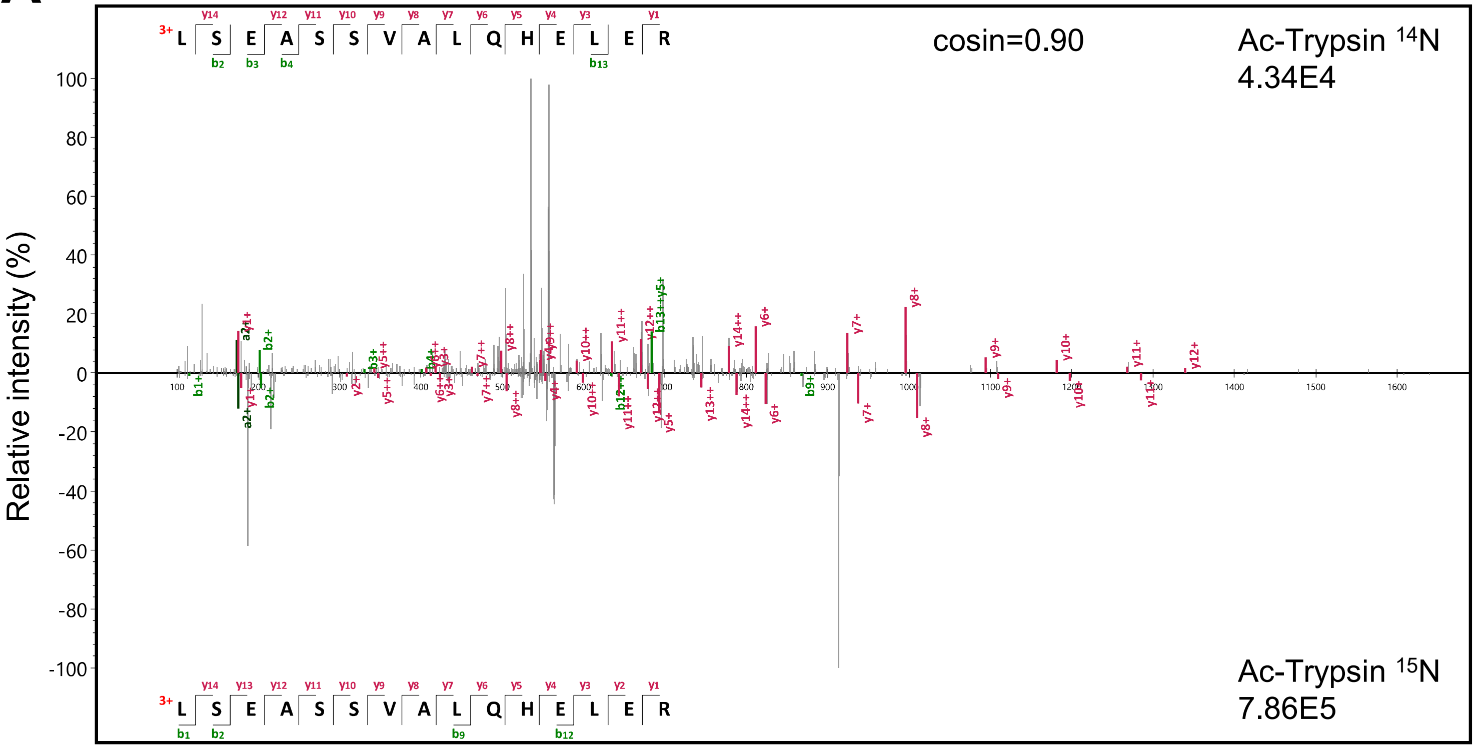

B

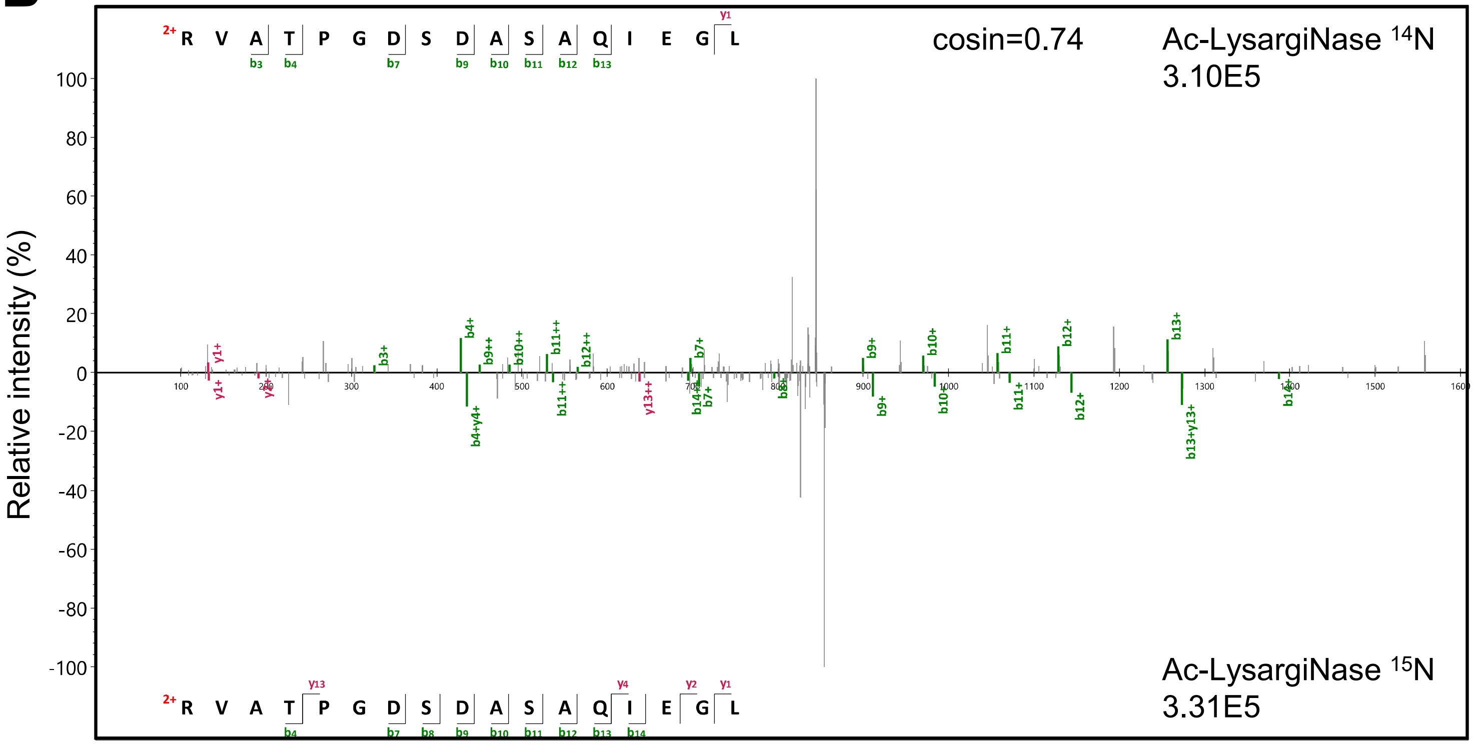

C

	Description	Scientific Name	Max Score	Total Score	Query Cover	E value	Per. Ident	Acc. Len	Accession
☑	hypothetical protein [[Mycobacterium] fortunisiensis]	[Mycobacterium] fortunisiensis	89.7	89.7	97%	8e-20	47.79%	134	WP_217154691.1
☑	hypothetical protein A5734_15385 [Mycolicibacterium fortuitum]	Mycolicibacterium fortuitum	66.6	66.6	60%	4e-11	47.06%	103	OMC01934.1
☑	hypothetical protein [Mycolicibacterium fortuitum]	Mycolicibacterium fortuitum	52.8	52.8	45%	3e-06	44.26%	62	WP_131827694.1
☑	hypothetical protein [Mycolicibacterium houstonense]	Mycolicibacterium houstonense	51.6	51.6	58%	6e-05	34.94%	146	WP_066900079.1
☑	hypothetical protein BVU76_18225 [Mycobacterium porcinum]	Mycobacterium porcinum	47.8	47.8	55%	0.001	38.27%	138	OLP00875.1
☑	hypothetical protein [Mycobacterium sp. DL440]	Mycobacterium sp. DL440	47.8	47.8	55%	0.001	36.71%	146	WP_166907519.1
☑	hypothetical protein [Mycolicibacterium farcinogenes]	Mycolicibacterium farcinogenes	47.0	47.0	58%	0.003	37.80%	149	WP_221852431.1

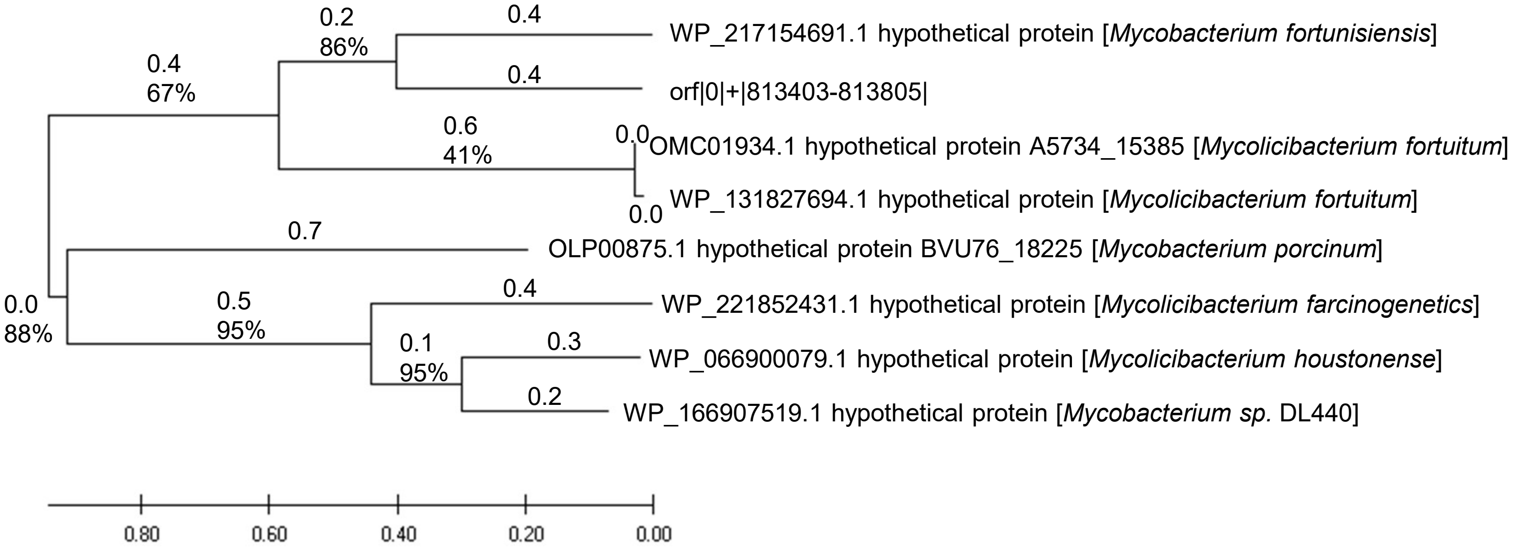

D

	Description	Scientific Name	Max Score	Total Score	Query Cover	E value	Per. Ident	Acc. Len	Accession
☑	Aquila chrysaetos chrysaetos genome assembly, chromosome: Z	Aquila chrysaetos chrysaetos	57.2	57.2	11%	0.002	86.96%	88216475	LR606180.1

E

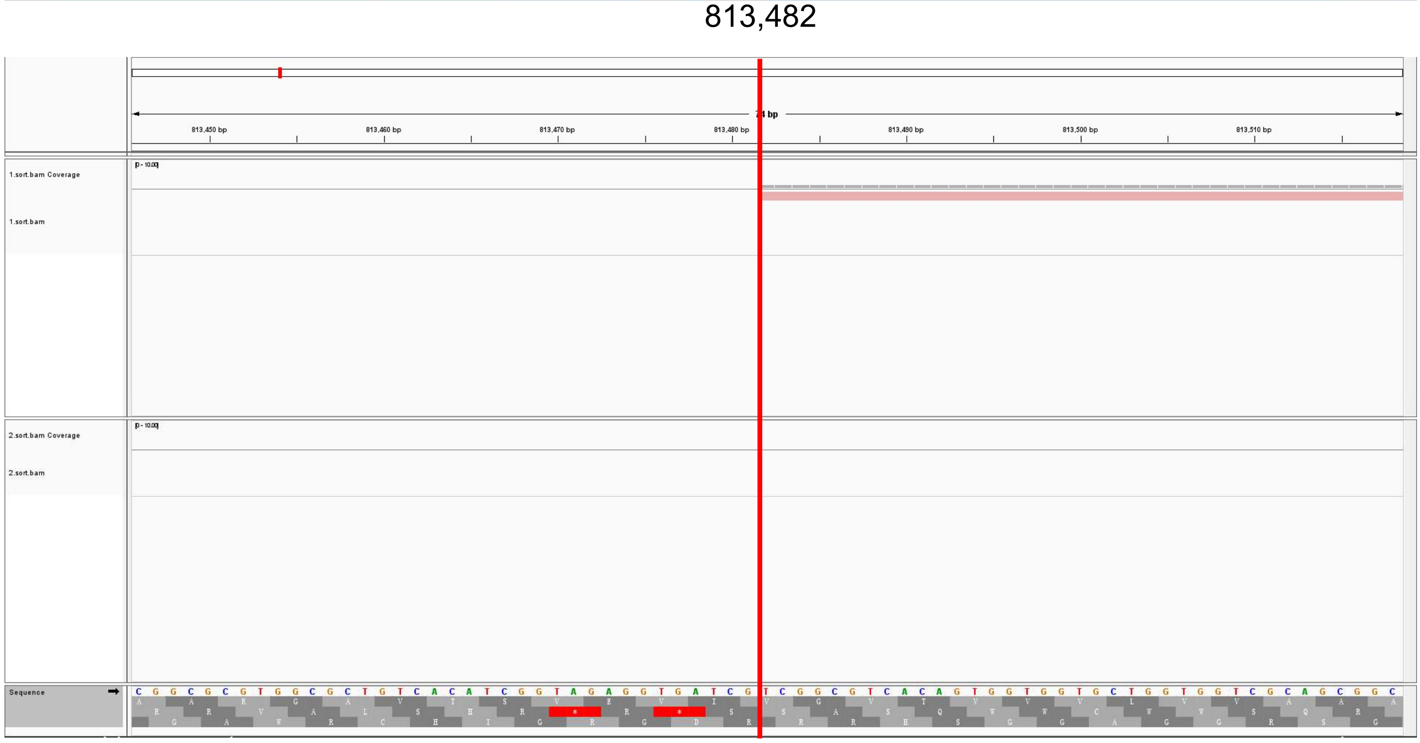

No. 10 orf|0|+|874119-875610|

A

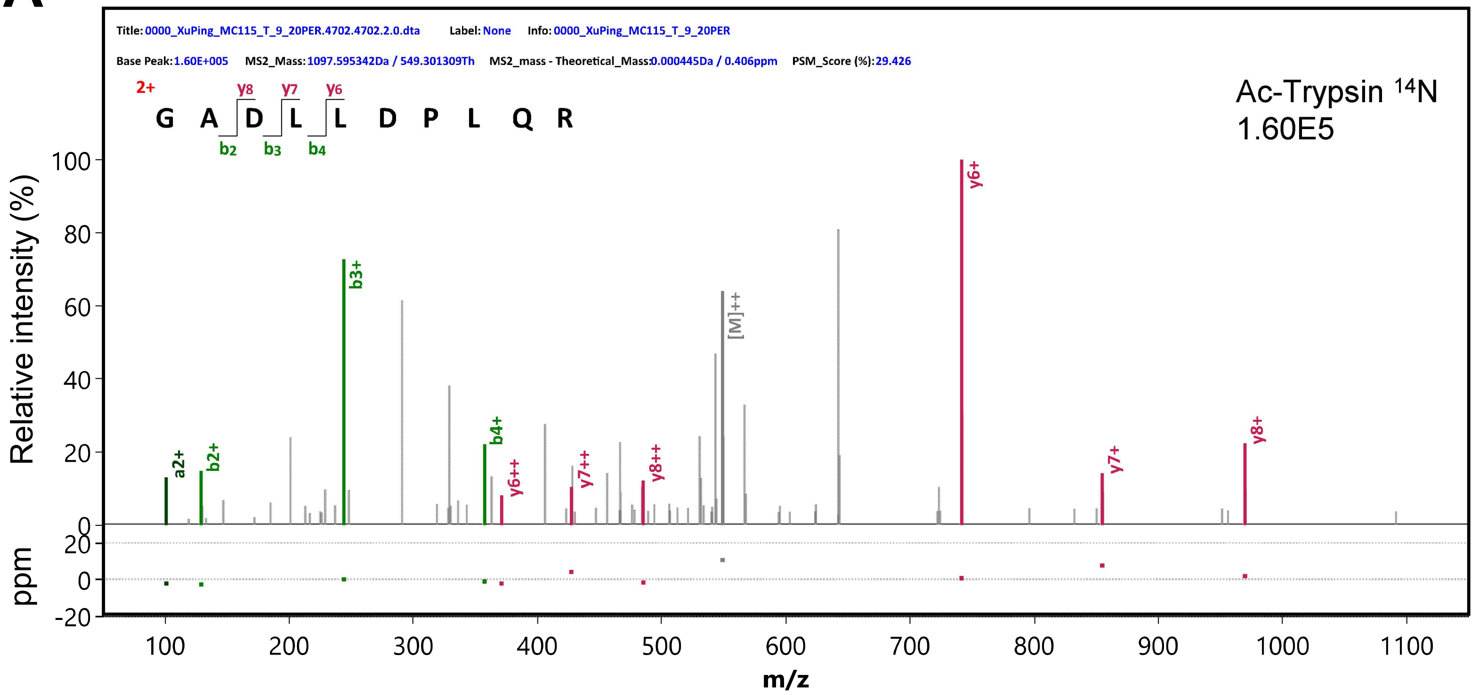

B

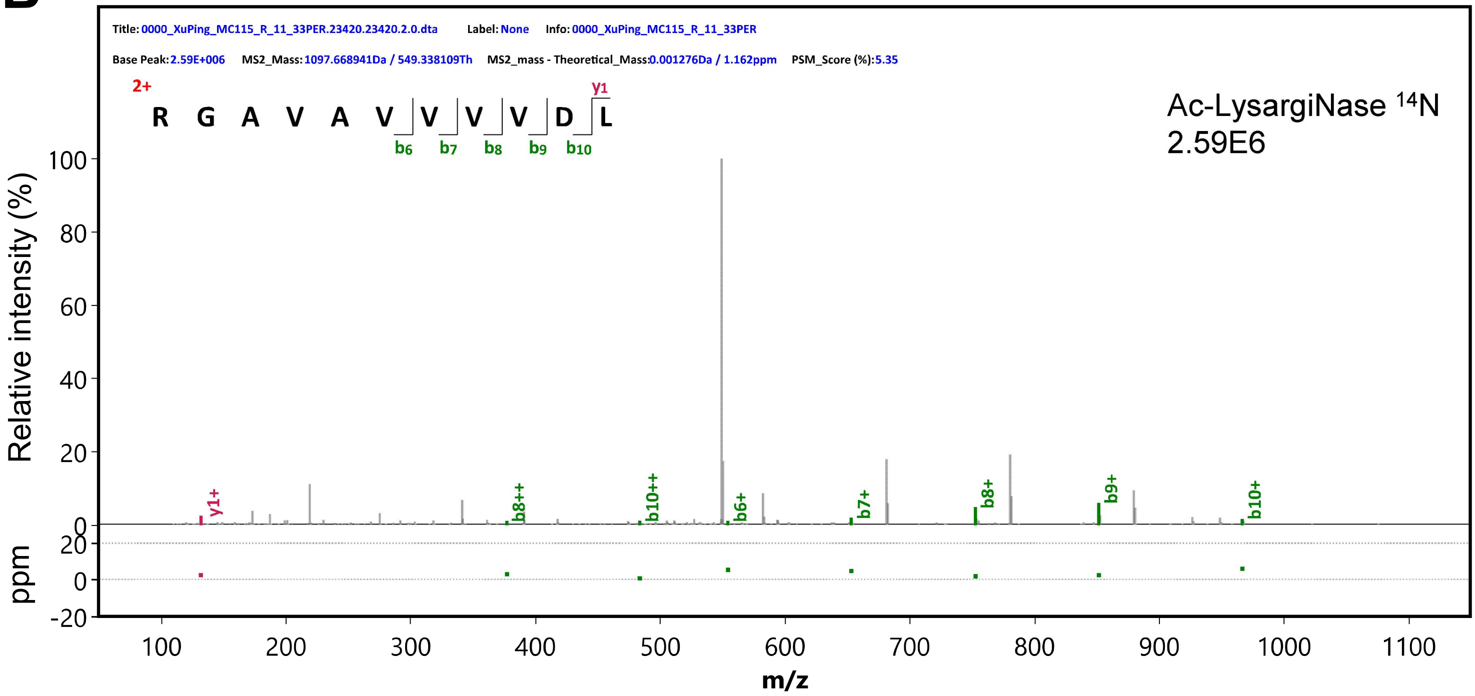

C

Description	Scientific Name	Max Score	Total Score	Query Cover	E value	Per. Ident	Acc. Len	Accession
hypothetical protein R1CP_07920 [Rhodococcus opacus]	Rhodococcus opacus	62.4	62.4	16%	1e-06	47.56%	249	ANS26305.1

D

Description	Scientific Name	Max Score	Total Score	Query Cover	E value	Per. Ident	Acc. Len	Accession
Mycobacterium goodii strain ATCC 700504 chromosome, complete genome	Mycobacterium ...	2082	2353	99%	0.0	91.84%	6741281	CP092364.1
Mycobacterium goodii strain X7B, complete genome	Mycobacterium ...	1687	2289	99%	0.0	92.61%	7105933	CP012150.1
Mycobacterium senegalense strain ATCC 35796 chromosome, complete genome	Mycobacterium ...	1303	1537	98%	0.0	83.05%	6086722	CP081000.1
Mycolicibacterium mageritense JCM 12375 DNA, complete genome	Mycolicibacteriu...	1061	1827	99%	0.0	83.06%	8006721	AP022567.1
Mycolicibacterium farcinogenes strain BKK/CU-MFGLA-001 chromosome, complete genome	Mycolicibacteriu...	1048	1674	94%	0.0	84.17%	6276329	CP080510.1
Mycolicibacterium farcinogenes strain BKK/CU-MFGFA-001 chromosome, complete genome	Mycolicibacteriu...	1037	1037	72%	0.0	83.99%	6344228	CP081673.1
Mycolicibacterium boenickei JCM 15653 DNA, complete genome	Mycolicibacteriu...	1003	1404	93%	0.0	83.52%	6563937	AP022579.1
Mycolicibacterium nivoides strain DL90 chromosome	Mycolicibacteriu...	985	1947	97%	0.0	82.94%	6905961	CP034072.1
Mycolicibacterium boenickei strain PDNC014 chromosome, complete genome	Mycolicibacteriu...	985	1922	97%	0.0	82.96%	7107504	CP070348.1
Mycobacterium fortuitum subsp. fortuitum DSM 46621 = ATCC 6841 genome	Mycolicibacteriu...	977	1510	95%	0.0	83.00%	6257075	CP014258.1

No. 11 orf|0|+|1852179-1853043|

A

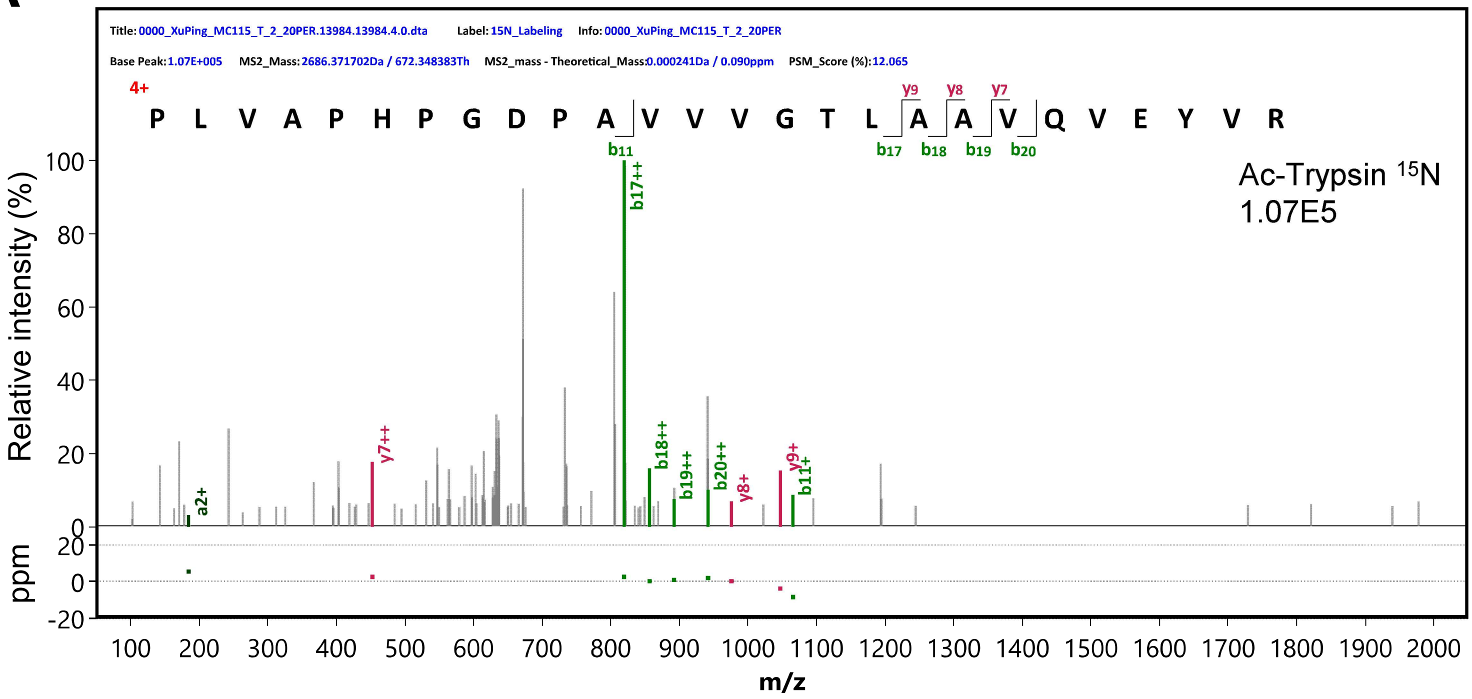

B

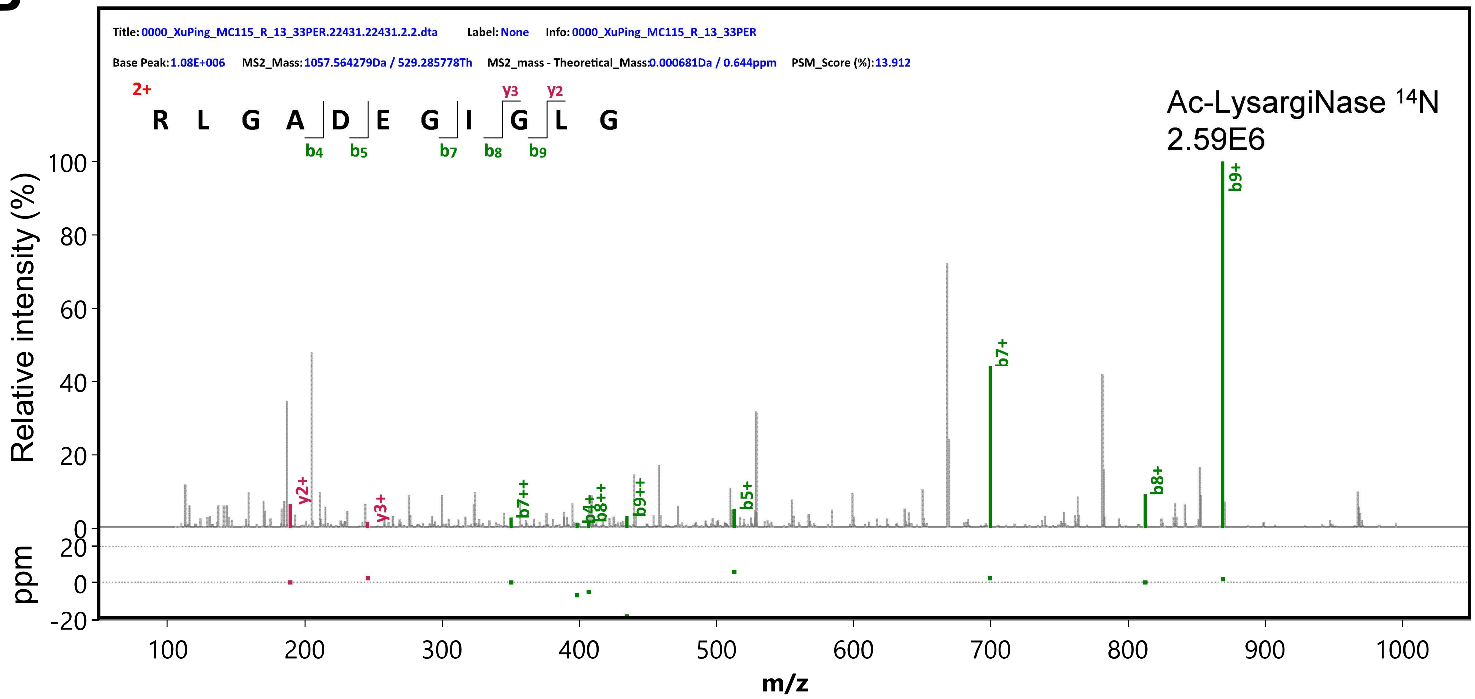

C

	Description	Scientific Name	Max Score	Total Score	Query Cover	E value	Per. Ident	Acc. Len	Accession
☑	hypothetical protein MHPYR_90049 [uncultured Mycobacterium sp.]	uncultured Mycobacterium sp.	110	110	96%	2e-23	40.77%	433	SBS79699.1

D

	Description	Scientific Name	Max Score	Total Score	Query Cover	E value	Per. Ident	Acc. Len	Accession
☑	Mycobacterium goodii strain ATCC 700504 chromosome, complete genome	Mycobacterium goodii	1007	1007	100%	0.0	87.99%	6741281	CP092364.1
☑	Mycobacterium goodii strain X7B, complete genome	Mycobacterium goodii	959	959	100%	0.0	86.95%	7105933	CP012150.1
☑	Mycolicibacterium farcinogenes strain BKK/CU-MFGFA-001 chromosome, complete gen...	Mycolicibacterium farcinogenes	556	556	88%	1e-153	80.08%	6344228	CP081673.1
☑	Mycobacterium senegalense strain ATCC 35796 chromosome, complete genome	Mycobacterium senegalense	551	551	88%	6e-152	79.95%	6086722	CP081000.1
☑	Mycolicibacterium farcinogenes strain BKK/CU-MFGLA-001 chromosome, complete gen...	Mycolicibacterium farcinogenes	540	540	88%	1e-148	79.69%	6276329	CP080510.1
☑	Mycolicibacterium fortuitum strain W4 chromosome	Mycolicibacterium fortuitum	531	531	87%	8e-146	79.55%	6674224	CP060409.1
☑	Mycolicibacterium alvei JCM 12272 DNA, complete genome	Mycolicibacterium alvei	514	514	87%	8e-141	79.11%	5712683	AP022565.1
☑	Mycolicibacterium mageritense JCM 12375 DNA, complete genome	Mycolicibacterium mageritense	484	484	64%	6e-132	82.42%	8006721	AP022567.1
☑	Mycolicibacterium psychrotolerans JCM 13323 DNA, complete genome	Mycolicibacterium psychrotolerans	385	385	90%	7e-102	75.84%	5732362	AP022574.1
☑	Mycobacterium rufum strain JCM 16372 chromosome, complete genome	Mycobacterium rufum	385	385	90%	7e-102	76.05%	5750471	CP092427.1

No. 12 orf|0|+|2205209-2205656|

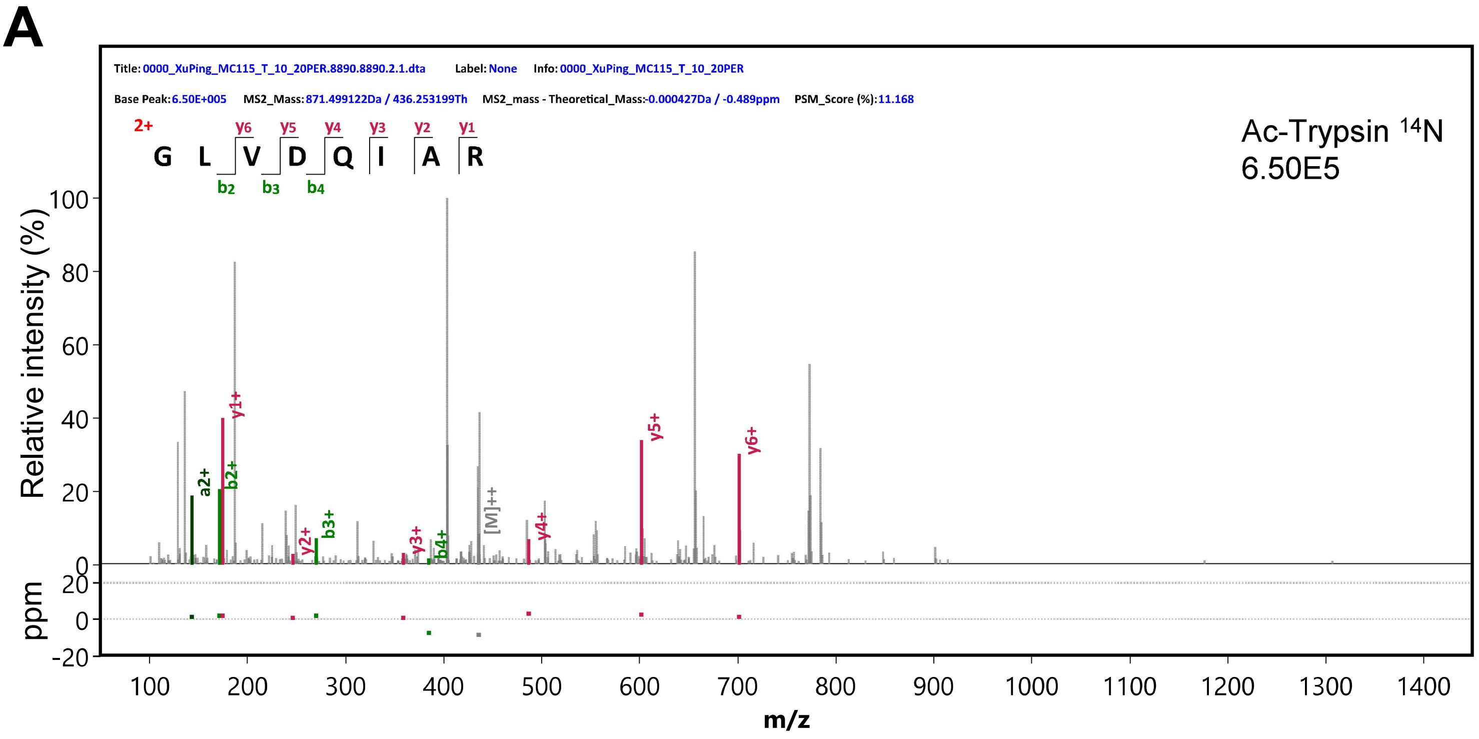

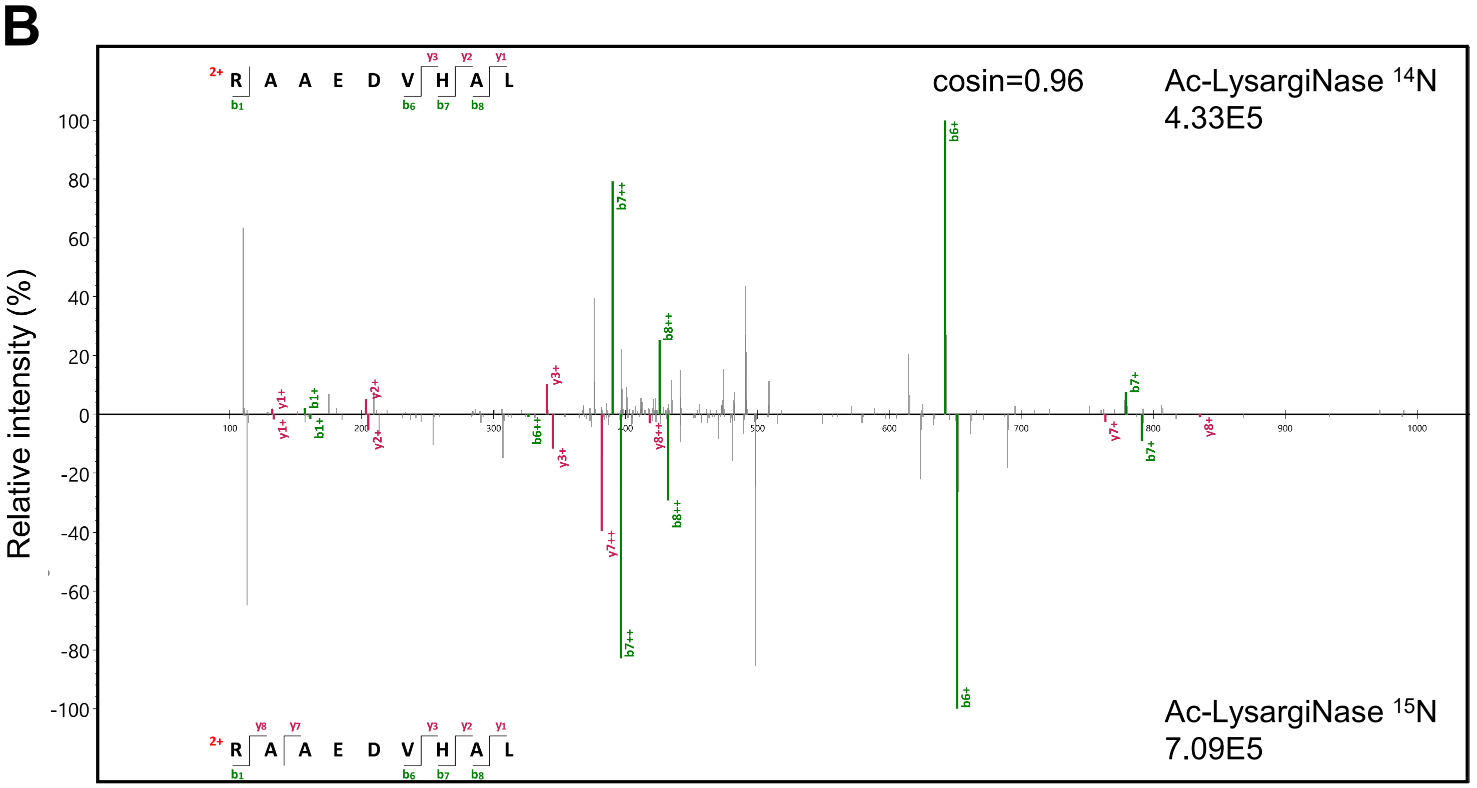

C

‹ Edit Search | Save Search | Search Summary ⌄ | How to read this report? | BLAST Help Videos | Back to Traditional Results Page

Job Title	orf|0|+|2205209-2205656|
RID	621SDG49013 *Search expires on 04-22 15:26 pm* Download All ⌄
Program	Citation ⌄
Database	nr See details ⌄
Query ID	lcl|Query_63242
Description	orf|0|+|2205209-2205656|
Molecule type	amino acid
Query Length	149
Other reports	

Filter Results

Percent Identity ___ to ___ | E value ___ to ___ | Query Coverage ___ to ___

Filter | Reset

No significant similarity found. For reasons why,click here

D

	Description	Scientific Name	Max Score	Total Score	Query Cover	E value	Per. Ident	Acc. Len	Accession
☑	Mycobacterium goodii strain X7B, complete genome	Mycobacterium ...	608	608	89%	2e-169	94.01%	7105933	CP012150.1
☑	Mycobacterium goodii strain ATCC 700504 chromosome, complete genome	Mycobacterium ...	542	542	96%	2e-149	89.35%	6741281	CP092364.1
☑	Mycolicibacterium litorale NIIDNTM18 DNA, complete genome	Mycolicibacteriu...	481	481	86%	4e-131	88.95%	5634149	AP023287.1
☑	Mycolicibacterium litorale JCM 17423 DNA, complete genome	Mycolicibacteriu...	475	475	86%	2e-129	88.69%	5579510	AP022586.1
☑	Mycolicibacterium septicum strain PDNC012 chromosome	Mycolicibacteriu...	455	455	90%	2e-123	87.04%	6515693	CP070349.1
☑	Mycolicibacterium boenickei JCM 15653 DNA, complete genome	Mycolicibacteriu...	453	453	90%	9e-123	86.98%	6563937	AP022579.1
☑	Mycobacterium fortuitum subsp. fortuitum DSM 46621 = ATCC 6841 genome	Mycolicibacteriu...	448	448	88%	4e-121	87.00%	6257075	CP014258.1
☑	Mycobacterium fortuitum strain CT6, complete genome	Mycolicibacteriu...	448	448	88%	4e-121	87.00%	6254616	CP011269.1
☑	Mycobacterium sp. VKM Ac-1817D, complete genome	Mycobacterium ...	448	448	88%	4e-121	87.00%	6324222	CP009914.1
☑	Mycolicibacterium fortuitum subsp. fortuitum JCM6387 DNA, complete genome	Mycolicibacteriu...	448	448	88%	4e-121	87.00%	6406072	AP025518.1

F

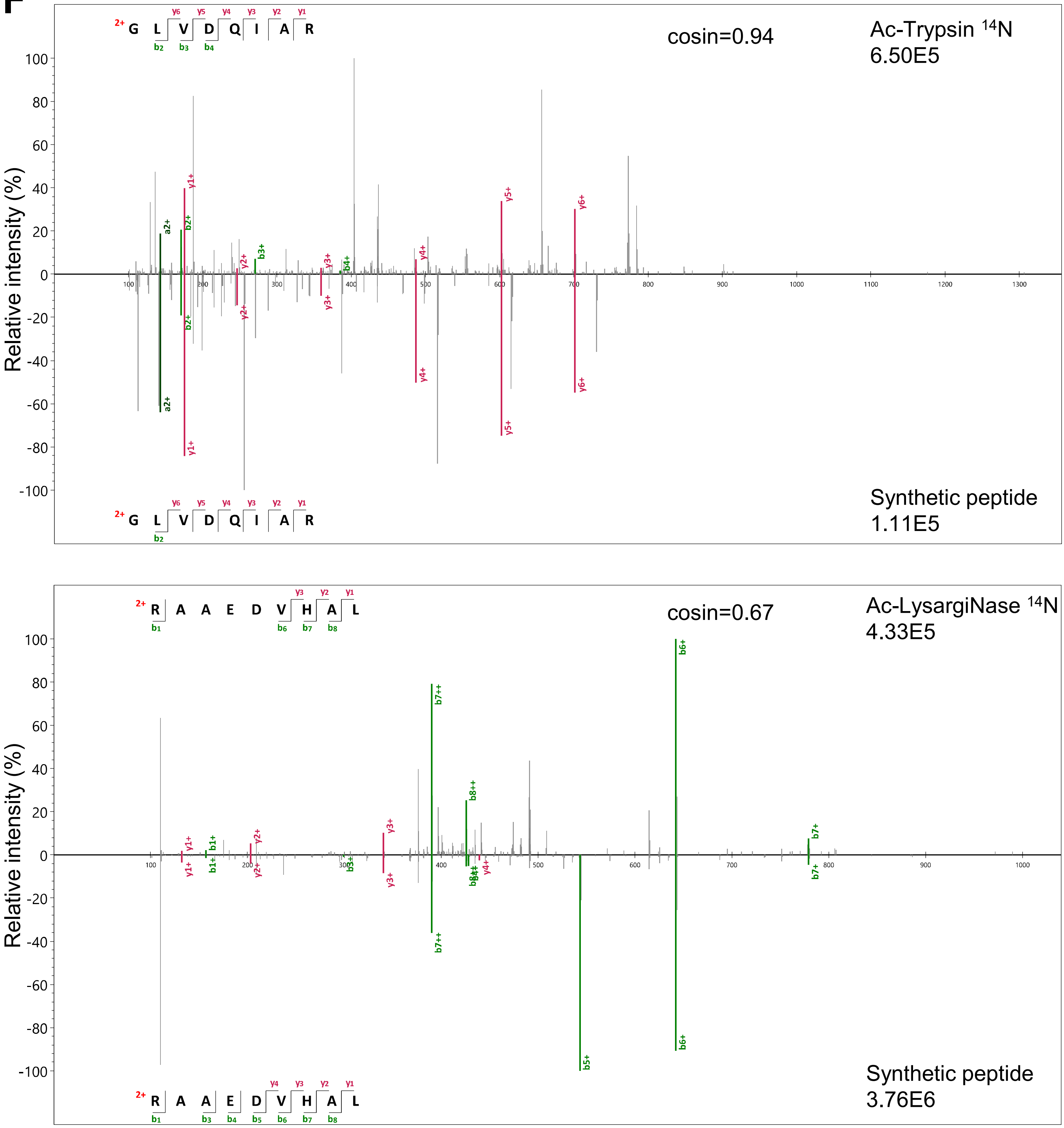
2+ G L V D Q I A R
y6 y5 y4 y3 y2 y1
b2 b3 b4
cosin=0.94
Ac-Trypsin 14N
6.50E5
Relative intensity (%)
100
80
60
40
20
0
-20
-40
-60
-80
-100
a2+ b2+ y1+ y2+ b3+ y3+ b4+ y4+ y5+ y6+
100 200 300 400 500 600 700 800 900 1000 1100 1200 1300
Synthetic peptide
1.11E5
2+ G L V D Q I A R
y6 y5 y4 y3 y2 y1
b2
2+ R A A E D V H A L
y3 y2 y1
b1 b6 b7 b8
cosin=0.67
Ac-LysargiNase 14N
4.33E5
Relative intensity (%)
y1+ b1+ y2+ b3+ y3+ b7++ b8++ y4+ b5+ b6+ b7+
100 200 300 400 500 600 700 800 900 1000
Synthetic peptide
3.76E6
2+ R A A E D V H A L
y4 y3 y2 y1
b1 b3 b4 b5 b6 b7 b8

No. 13 orf|0|+|3841204-3842056|

A

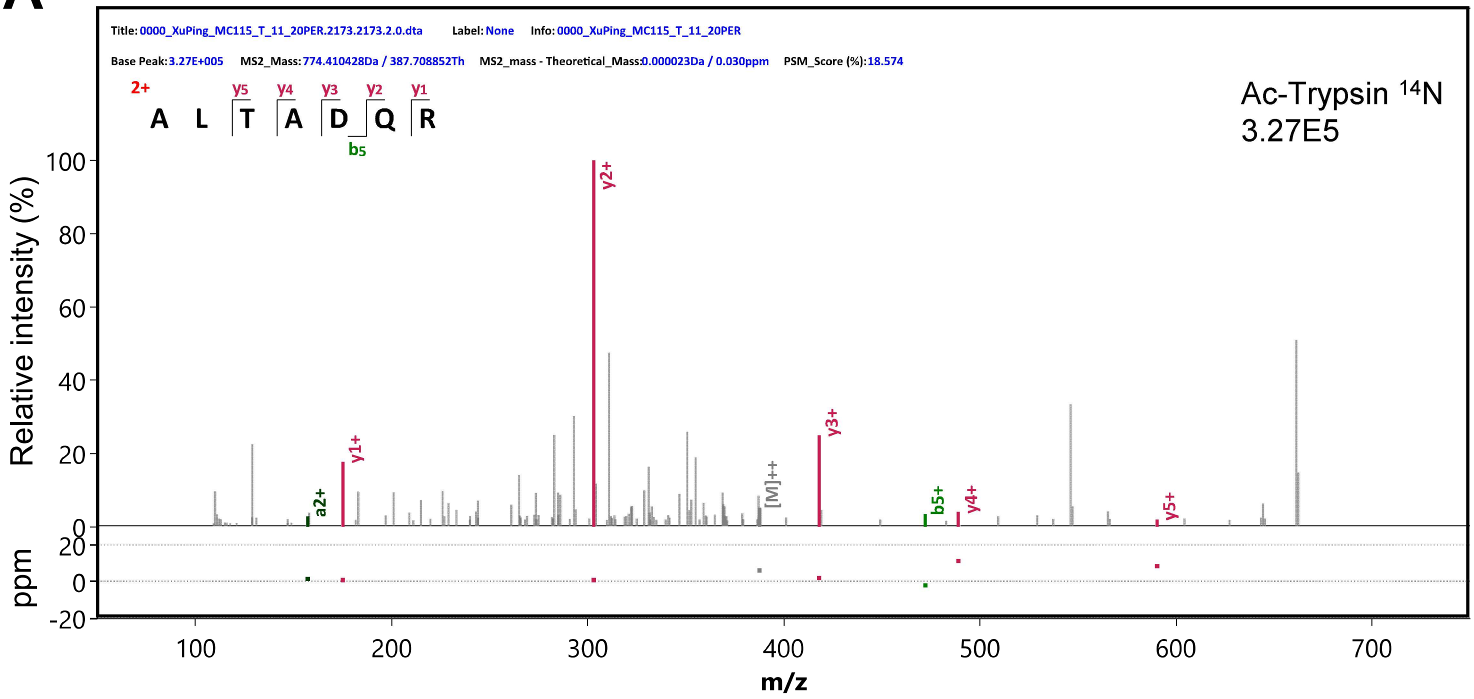

B

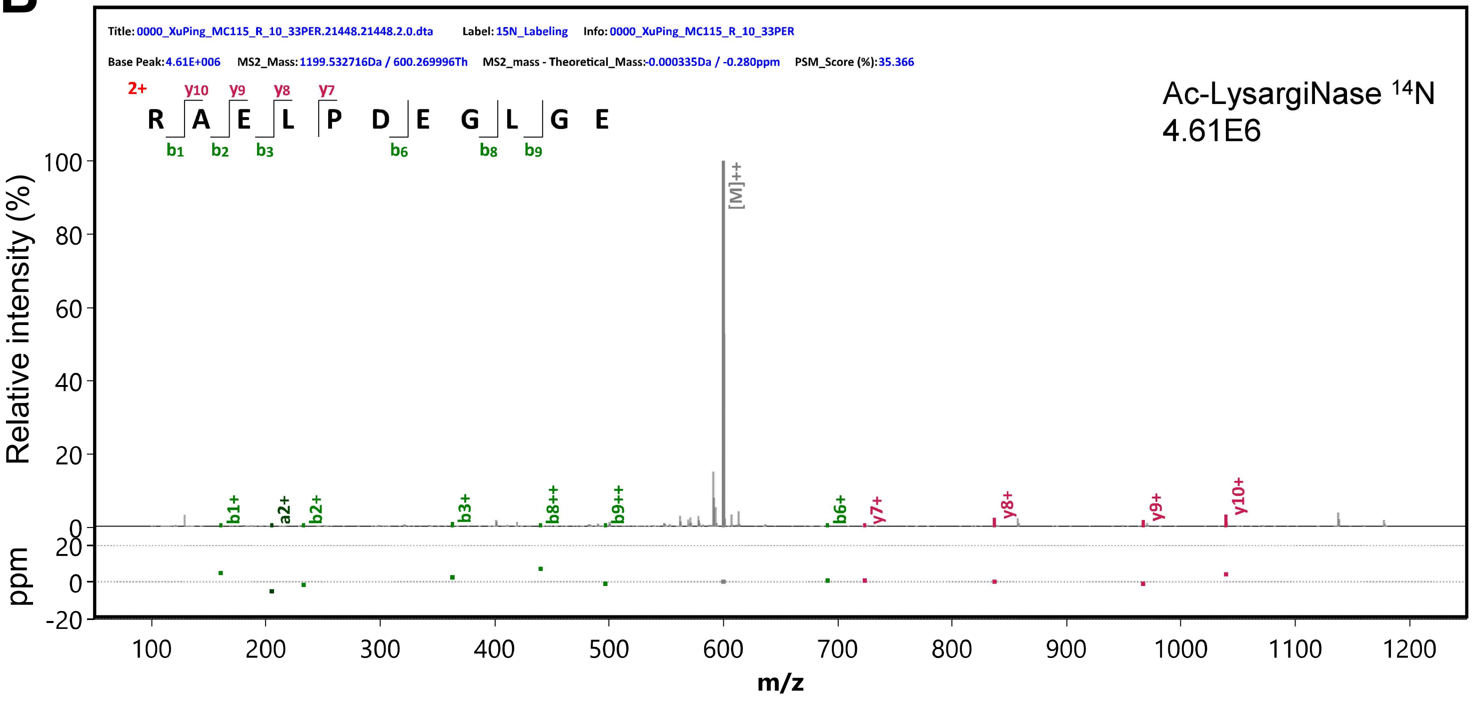

C

	Description	Scientific Name	Max Score	Total Score	Query Cover	E value	Per. Ident	Acc. Len	Accession
☑	ion transporter [Nocardia brasiliensis ATCC 700358]	Nocardia brasiliensis ATCC 700358	179	179	100%	5e-48	47.56%	566	AFU00665.1
☑	Uncharacterised protein [Mycobacteroides abscessus subsp. abscessus]	Mycobacteroides abscessus subsp. abscessus	100	100	52%	6e-21	49.33%	274	SKY78804.1
☑	Uncharacterised protein [Mycobacteroides abscessus subsp. abscessus]	Mycobacteroides abscessus subsp. abscessus	72.8	72.8	66%	4e-11	38.97%	222	SIJ87249.1
☑	Uncharacterised protein [Mycobacterium tuberculosis]	Mycobacterium tuberculosis	66.2	66.2	14%	3e-09	82.93%	152	CNV64836.1
☑	Uncharacterised protein [Mycobacteroides abscessus subsp. massiliense]	Mycobacteroides abscessus subsp. massiliense	58.2	58.2	36%	4e-06	36.36%	178	SKN51276.1
☑	hypothetical protein GY15_25185 [Delftia sp. 670]	Delftia sp. 670	57.8	57.8	35%	2e-05	41.67%	438	KEH11871.1

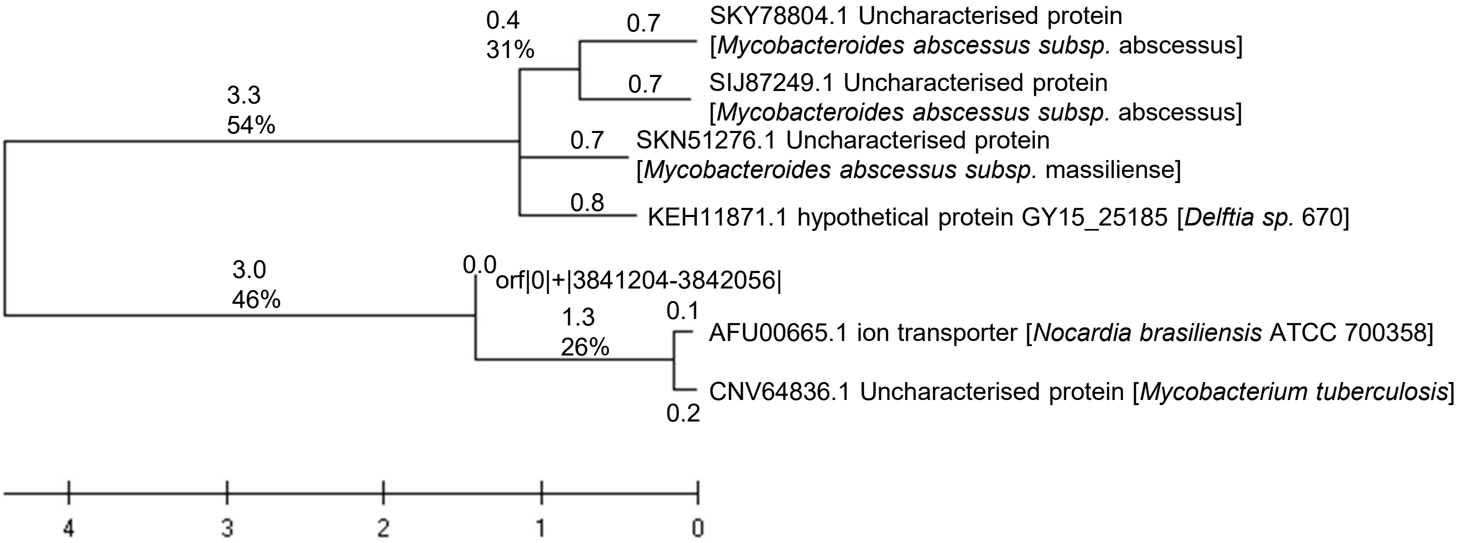

D

	Description	Scientific Name	Max Score	Total Score	Query Cover	E value	Per. Ident	Acc. Len	Accession
☑	Mycobacterium goodii strain ATCC 700504 chromosome, complete genome	Mycobacterium ...	1088	1088	100%	0.0	89.68%	6741281	CP092364.1
☑	Mycobacterium dioxanotrophicus strain PH-06 chromosome, complete genome	Mycobacterium ...	900	900	100%	0.0	85.78%	7595921	CP020809.1
☑	Mycolicibacterium septicum strain PDNC012 chromosome	Mycolicibacteriu...	889	889	100%	0.0	85.51%	6515693	CP070349.1
☑	Mycolicibacterium boenickei JCM 15653 DNA, complete genome	Mycolicibacteriu...	885	885	100%	0.0	85.41%	6563937	AP022579.1
☑	Mycolicibacterium mageritense JCM 12375 DNA, complete genome	Mycolicibacteriu...	878	878	100%	0.0	85.25%	8006721	AP022567.1
☑	Mycolicibacterium alvei JCM 12272 DNA, complete genome	Mycolicibacteriu...	870	870	99%	0.0	85.21%	5712683	AP022565.1
☑	Mycobacteroides chelonae strain NCTC946 genome assembly, chromosome: 1	Mycobacteroide...	861	861	100%	0.0	84.93%	5348362	LR134345.1
☑	Mycolicibacterium phlei strain CCUG 21000 chromosome, complete genome	Mycolicibacteriu...	861	861	100%	0.0	84.93%	5349645	CP014475.1
☑	Mycolicibacterium boenickei strain PDNC014 chromosome, complete genome	Mycolicibacteriu...	861	861	100%	0.0	84.93%	7107504	CP070348.1
☑	Mycolicibacterium nivoides strain DL90 chromosome	Mycolicibacteriu...	850	850	100%	0.0	84.70%	6905961	CP034072.1

No. 14 orf|0|-|4966072-4966744|

A

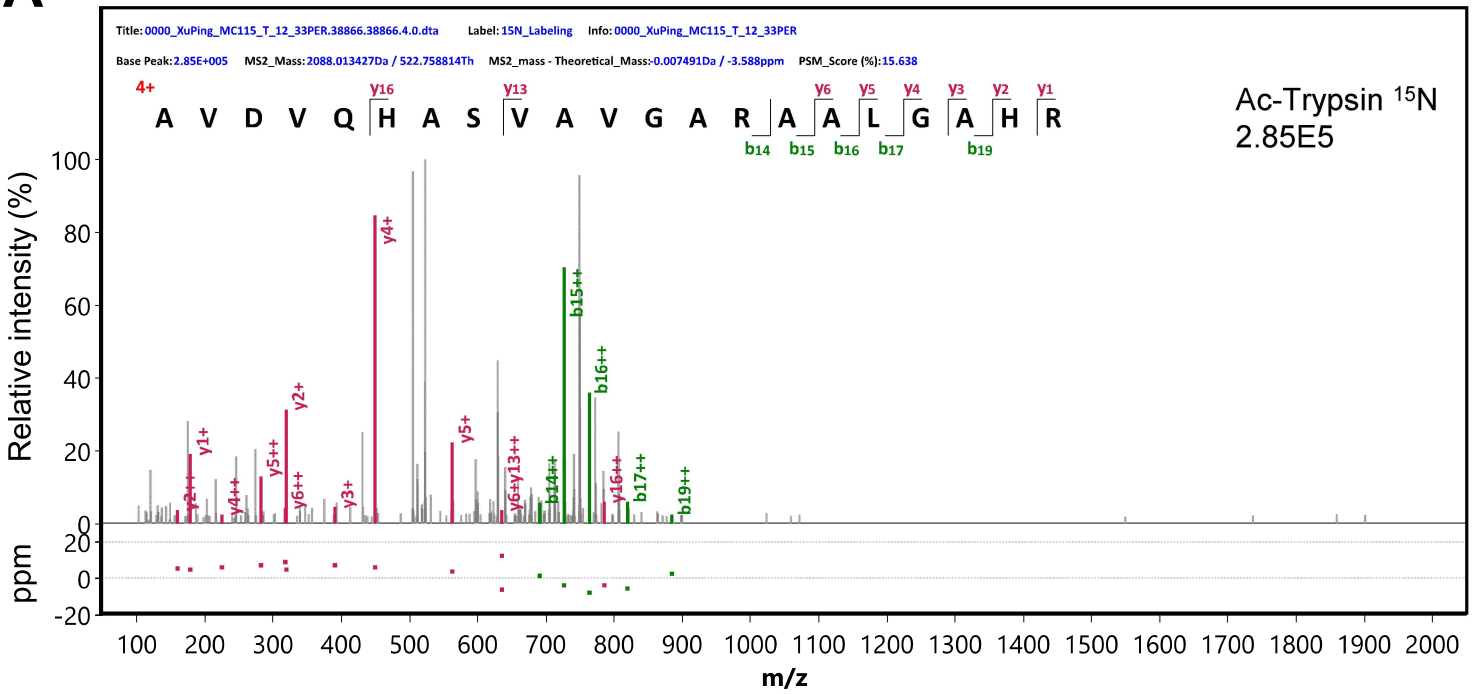

B

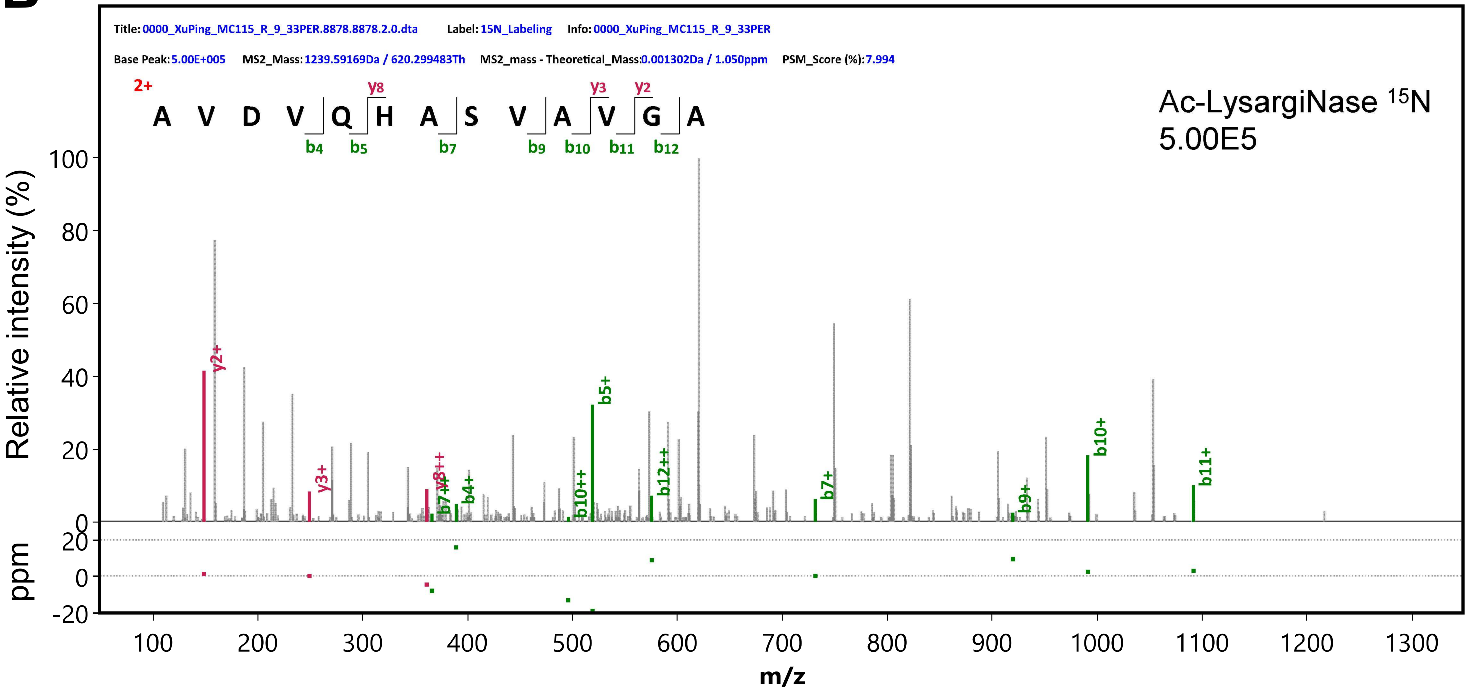

C

	Description	Scientific Name	Max Score	Total Score	Query Cover	E value	Per. Ident	Acc. Len	Accession
☑	hypothetical protein C1Y40_00650 [Mycobacterium talmoniae]	Mycobacterium talmoniae	129	129	60%	8e-31	58.52%	536	PQM49125.1

D

	Description	Scientific Name	Max Score	Total Score	Query Cover	E value	Per. Ident	Acc. Len	Accession
☑	Mycobacterium goodii strain X7B, complete genome	Mycobacterium ...	863	863	99%	0.0	89.90%	7105933	CP012150.1
☑	Mycobacterium goodii strain ATCC 700504 chromosome, complete genome	Mycobacterium ...	863	863	99%	0.0	89.88%	6741281	CP092364.1
☑	Mycobacterium sp. DL440 chromosome	Mycobacterium ...	645	645	99%	2e-180	84.25%	6381854	CP050191.1
☑	Mycolicibacterium mageritense JCM 12375 DNA, complete genome	Mycolicibacteriu...	645	645	99%	2e-180	84.32%	8006721	AP022567.1
☑	Mycolicibacterium boenickei JCM 15653 DNA, complete genome	Mycolicibacteriu...	614	614	99%	6e-171	83.36%	6563937	AP022579.1
☑	Mycobacterium senegalense strain ATCC 35796 chromosome, complete genome	Mycobacterium ...	604	604	99%	4e-168	83.28%	6086722	CP081000.1
☑	Mycolicibacterium alvei JCM 12272 DNA, complete genome	Mycolicibacteriu...	603	603	99%	1e-167	83.16%	5712683	AP022565.1
☑	Mycolicibacterium farcinogenes strain BKK/CU-MFGFA-001 chromosome, complete genome	Mycolicibacteriu...	599	599	99%	2e-166	83.08%	6344228	CP081673.1
☑	Mycolicibacterium farcinogenes strain BKK/CU-MFGLA-001 chromosome, complete genome	Mycolicibacteriu...	599	599	99%	2e-166	83.01%	6276329	CP080510.1
☑	Mycobacterium diernhoferi strain ATCC 19340 chromosome, complete genome	Mycobacterium ...	590	590	99%	1e-163	82.92%	5998503	CP080332.1

No. 15 orf|0|-|5450187-5451333|

A

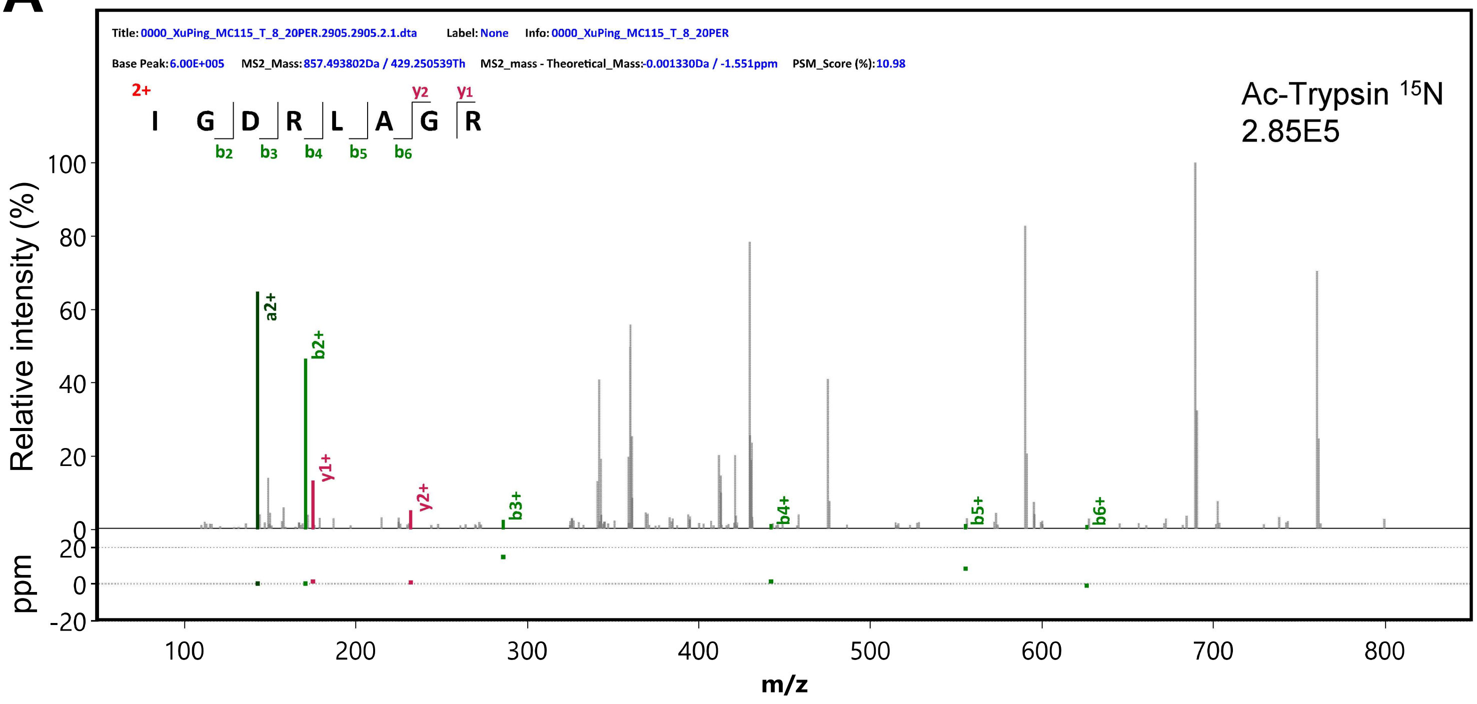

B

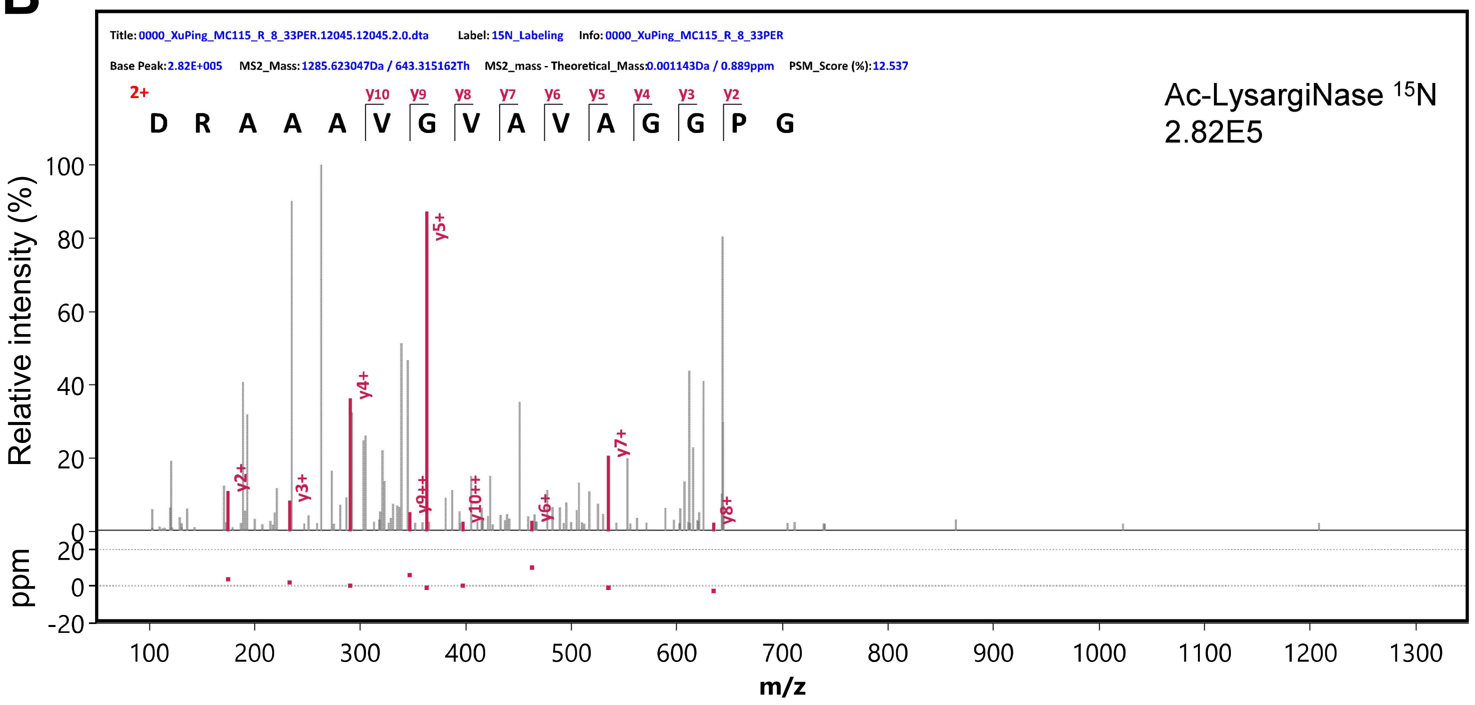

C

‹ Edit Search | Save Search | Search Summary ⌄ | ❓ How to read this report? | BLAST Help Videos | Back to Traditional Results Page

Job Title	**orf|0|-|5450187-5451333|**
RID	622C4F3U016 *Search expires on 04-22 15:36 pm* Download All ⌄
Program	❓ Citation ⌄
Database	nr See details ⌄
Query ID	lcl|Query_69785
Description	orf|0|-|5450187-5451333|
Molecule type	amino acid
Query Length	382
Other reports	❓

Filter Results

Percent Identity [ ] to [ ] E value [ ] to [ ] Query Coverage [ ] to [ ]

Filter | Reset

⚠ No significant similarity found. For reasons why, click here

D

	Description	Scientific Name	Max Score	Total Score	Query Cover	E value	Per. Ident	Acc. Len	Accession
☑	Mycobacteroides chelonae strain NCTC946 genome assembly, chromosome: 1	Mycobacteroides chelonae	468	468	55%	8e-127	79.91%	5348362	LR134345.1
☑	Mycolicibacterium phlei strain CCUG 21000 chromosome, complete genome	Mycolicibacterium phlei	468	468	55%	8e-127	79.91%	5349645	CP014475.1
☑	Mycolicibacterium hassiacum DSM 44199 isolate Mhassiacum genome assembly, chrom...	Mycolicibacterium hassiacum DSM 44...	457	457	55%	2e-123	79.63%	5269097	LR026975.1
☑	Mycolicibacterium parafortuitum JCM 6367 DNA, nearly complete genome	Mycolicibacterium parafortuitum	422	422	97%	7e-113	73.86%	6044196	AP022598.1
☑	Mycolicibacterium insubricum JCM 16366 DNA, nearly complete genome	Mycolicibacterium insubricum	401	401	98%	9e-107	73.52%	4577045	AP022618.1
☑	Mycolicibacterium austroafricanum strain MYC038 chromosome	Mycolicibacterium austroafricanum	289	289	55%	7e-73	75.04%	6260785	CP082189.1
☑	Mycolicibacterium vanbaalenii strain JOB5 chromosome	Mycolicibacterium vanbaalenii	283	283	55%	3e-71	74.85%	6338794	CP060055.1
☑	Mycolicibacterium austroafricanum strain MYC211 chromosome	Mycolicibacterium austroafricanum	283	283	55%	3e-71	74.85%	6189551	CP082191.1
☑	Mycolicibacterium austroafricanum strain MYC221 chromosome	Mycolicibacterium austroafricanum	283	283	55%	3e-71	74.85%	6287627	CP070380.1
☑	Mycobacterium vanbaalenii PYR-1, complete genome	Mycolicibacterium vanbaalenii PYR-1	283	283	55%	3e-71	74.85%	6491865	CP000511.1

No. 16 orf|0|+|5885997-5886810|

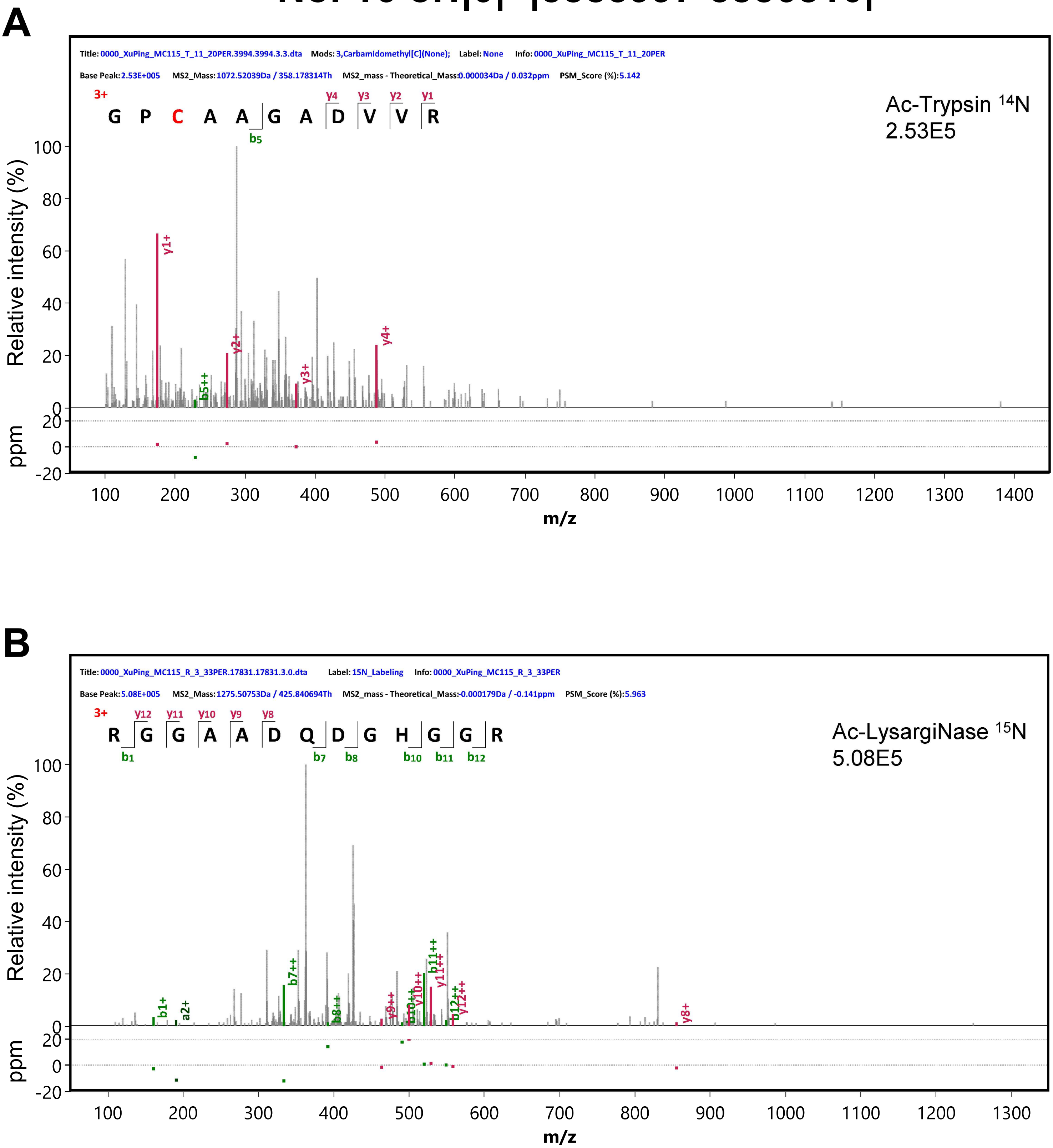

C

‹ Edit Search | Save Search | Search Summary ⌄ | How to read this report? | BLAST Help Videos | Back to Traditional Results Page

Job Title	orf|0|+|5885997-5886810|
RID	622G6YEJ016 *Search expires on 04-22 15:38 pm* Download All ⌄
Program	Citation ⌄
Database	nr See details ⌄
Query ID	lcl|Query_72257
Description	orf|0|+|5885997-5886810|
Molecule type	amino acid
Query Length	271
Other reports	

Filter Results

Percent Identity ___ to ___ | E value ___ to ___ | Query Coverage ___ to ___

Filter | Reset

⚠ No significant similarity found. For reasons why, click here

D

‹ Edit Search | Save Search | Search Summary ⌄ | How to read this report? | BLAST Help Videos | Back to Traditional Results Page

Your search is limited to records that exclude: Mycolicibacterium smegmatis (taxid:1772)

Job Title	orf|0|+|5885997-5886810|
RID	622GFPN6013 *Search expires on 04-22 15:38 pm* Download All ⌄
Program	Citation ⌄
Database	nt See details ⌄
Query ID	lcl|Query_56565
Description	orf|0|+|5885997-5886810|
Molecule type	dna
Query Length	814
Other reports	

Filter Results

Percent Identity ___ to ___ | E value ___ to ___ | Query Coverage ___ to ___

Filter | Reset

⚠ No significant similarity found. For reasons why, click here

Somewhat blastn

Description	Scientific Name	Max Score	Total Score	Query Cover	E value	Per. Ident	Acc. Len	Accession
Mycobacterium fortuitum subsp. fortuitum DSM 46621 = ATCC 6841 genome	Mycolicibacterium fortuitum subsp. fortuitu...	204	204	80%	4e-47	67.71%	6257075	CP014258.1
Mycobacterium sp. VKM Ac-1817D, complete genome	Mycobacterium sp. VKM Ac-1817D	204	204	80%	4e-47	67.71%	6324222	CP009914.1
Mycolicibacterium fortuitum subsp. fortuitum JCM6387 DNA, complete genome	Mycolicibacterium fortuitum subsp. fortuitum	204	204	80%	4e-47	67.71%	6406072	AP025518.1
Mycobacterium fortuitum strain CT6, complete genome	Mycolicibacterium fortuitum	200	200	80%	4e-46	67.21%	6254616	CP011269.1
Mycolicibacterium alvei JCM 12272 DNA, complete genome	Mycolicibacterium alvei	197	197	86%	2e-45	67.13%	5712683	AP022565.1
Mycolicibacterium fortuitum strain Kelantanesensis chromosome, complete genome	Mycolicibacterium fortuitum	196	196	80%	5e-45	67.21%	6419378	CP089608.1
Mycolicibacterium fortuitum strain W6 chromosome	Mycolicibacterium fortuitum	196	196	80%	5e-45	67.21%	6555851	CP060410.1
Mycolicibacterium fortuitum strain W4 chromosome	Mycolicibacterium fortuitum	186	186	70%	1e-41	67.75%	6674224	CP060409.1
Mycobacterium sp. DL440 chromosome	Mycobacterium sp. DL440	175	175	81%	2e-38	66.37%	6381854	CP050191.1
Mycolicibacterium nivoides strain DL90 chromosome	Mycolicibacterium nivoides	159	159	88%	4e-34	65.38%	6905961	CP034072.1

No. 17 orf|0|-|6720526-6721000|

A

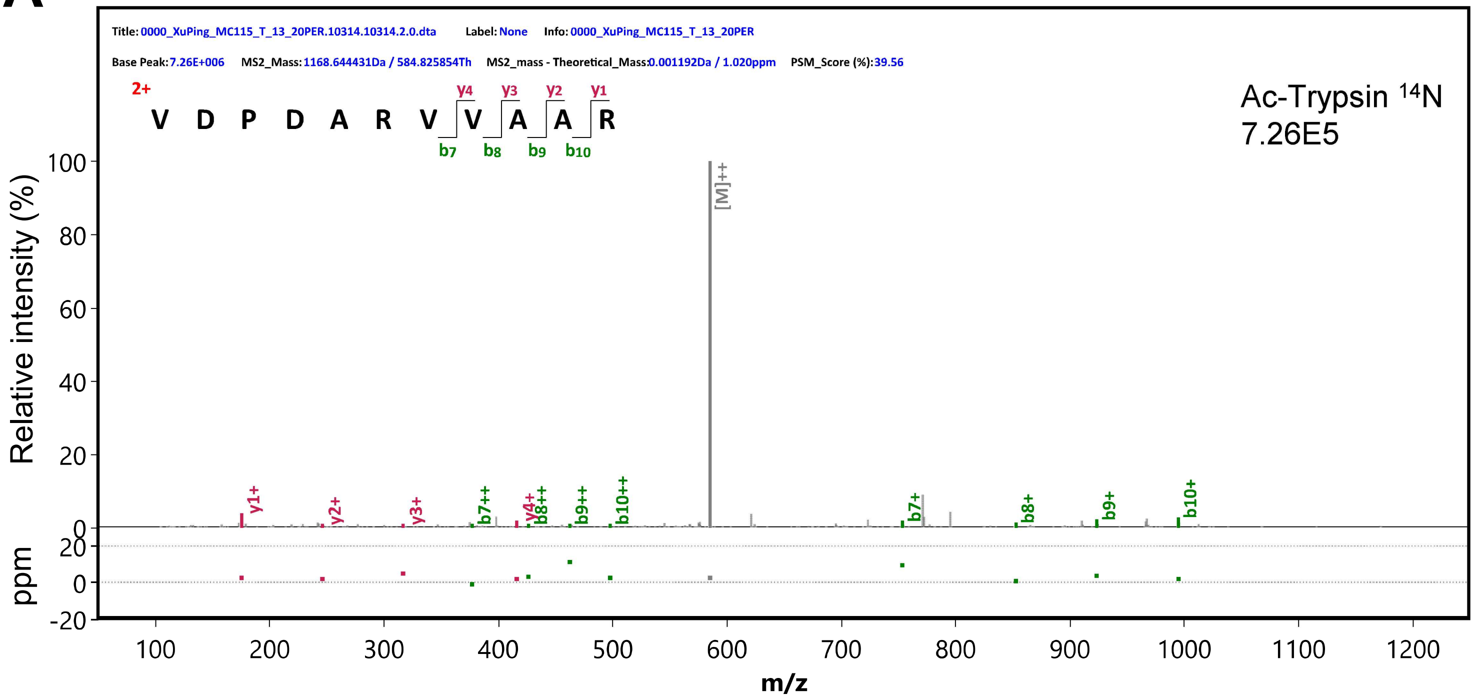

B

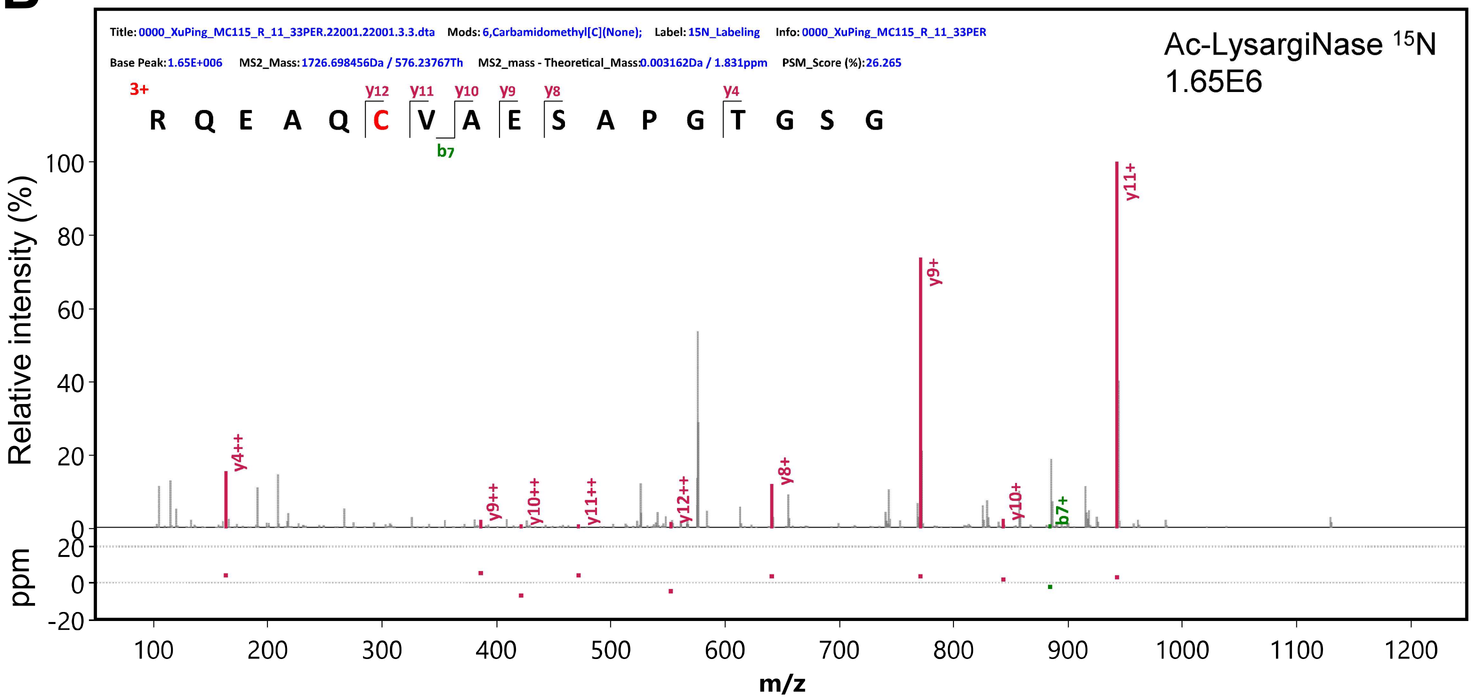

C

‹ Edit Search | Save Search | Search Summary ⌄ | How to read this report? | BLAST Help Videos | Back to Traditional Results Page

Job Title	orf|0|-|6720526-6721000|
RID	622NN73G016 *Search expires on 04-22 15:41 pm* Download All ⌄
Program	Citation ⌄
Database	nr See details ⌄
Query ID	lcl|Query_29864
Description	orf|0|-|6720526-6721000|
Molecule type	amino acid
Query Length	158
Other reports	

Filter Results

Percent Identity ___ to ___ | E value ___ to ___ | Query Coverage ___ to ___

Filter | Reset

No significant similarity found. For reasons why, click here

D

	Description	Scientific Name	Max Score	Total Score	Query Cover	E value	Per. Ident	Acc. Len	Accession
☑	Mycobacterium goodii strain ATCC 700504 chromosome, complete genome	Mycobacterium goodii	486	486	95%	9e-133	85.96%	6741281	CP092364.1
☑	Mycolicibacterium baixiangningiae strain LJ126 chromosome, complete genome	Mycolicibacterium baixiangningiae	392	392	86%	2e-104	83.82%	6181092	CP066218.1
☑	Mycolicibacterium litorale JCM 17423 DNA, complete genome	Mycolicibacterium litorale	385	385	89%	3e-102	83.06%	5579510	AP022586.1
☑	Mycobacterium sp. djl-10, complete genome	Mycobacterium sp. djl-10	320	320	87%	1e-82	80.58%	6395946	CP016640.1
☑	Mycolicibacterium arabiense JCM 18538 DNA, complete genome	Mycolicibacterium arabiense	302	302	88%	4e-77	79.72%	6017160	AP022593.1
☑	Nocardioides houyundeii strain 78 chromosome, complete genome	Nocardioides houyundeii	259	259	74%	2e-64	80.00%	3840802	CP025581.1
☑	Nocardioides sp. ZJ1313 chromosome, complete genome	Nocardioides sp. ZJ1313	246	246	75%	2e-60	79.40%	4264922	CP061769.1
☑	Nocardioides dongkuii strain S-713 chromosome, complete genome	Nocardioides dongkuii	231	231	72%	5e-56	79.08%	4207844	CP059903.1
☑	Methylobacterium brachiatum strain TX0642 chromosome	Methylobacterium brachiatum	97.1	97.1	16%	2e-15	88.10%	5982025	CP033231.1
☑	Thermobispora bispora strain ZC4 chromosome, complete genome	Thermobispora bispora	97.1	97.1	14%	2e-15	91.43%	4241484	CP039124.1

Supplementary Figure 10

No. 2 orf|0|-|1217294-1218644|

Blackburn[2]

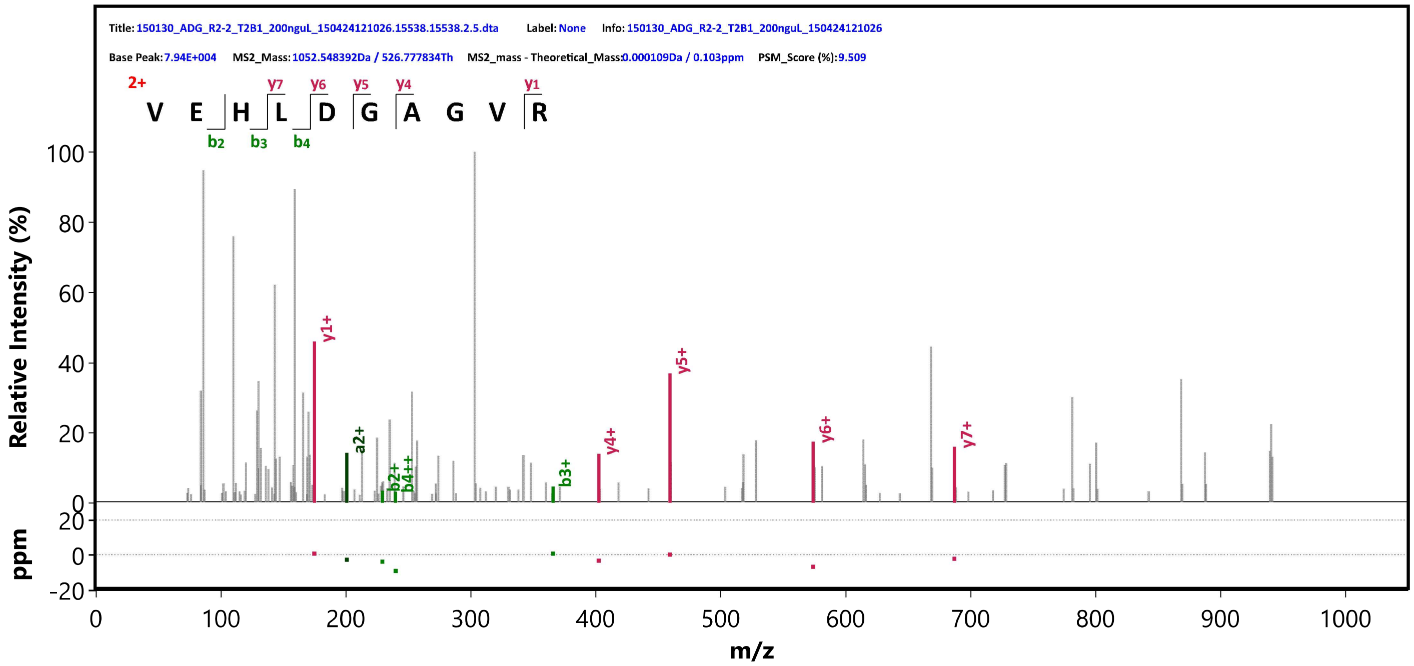

No. 6 orf|0|-|6584055-6584880|

Blackburn[1]

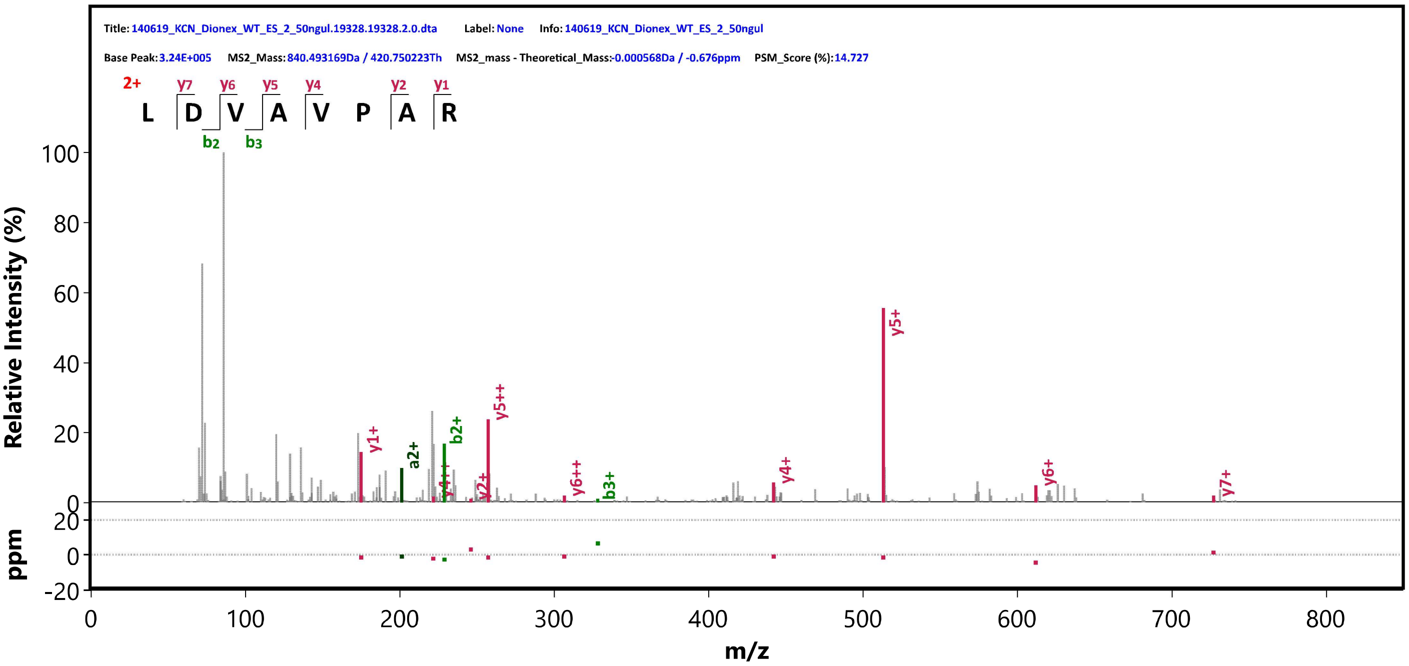

No. 9 orf|0|+|813403-813805|

Blackburn[1]

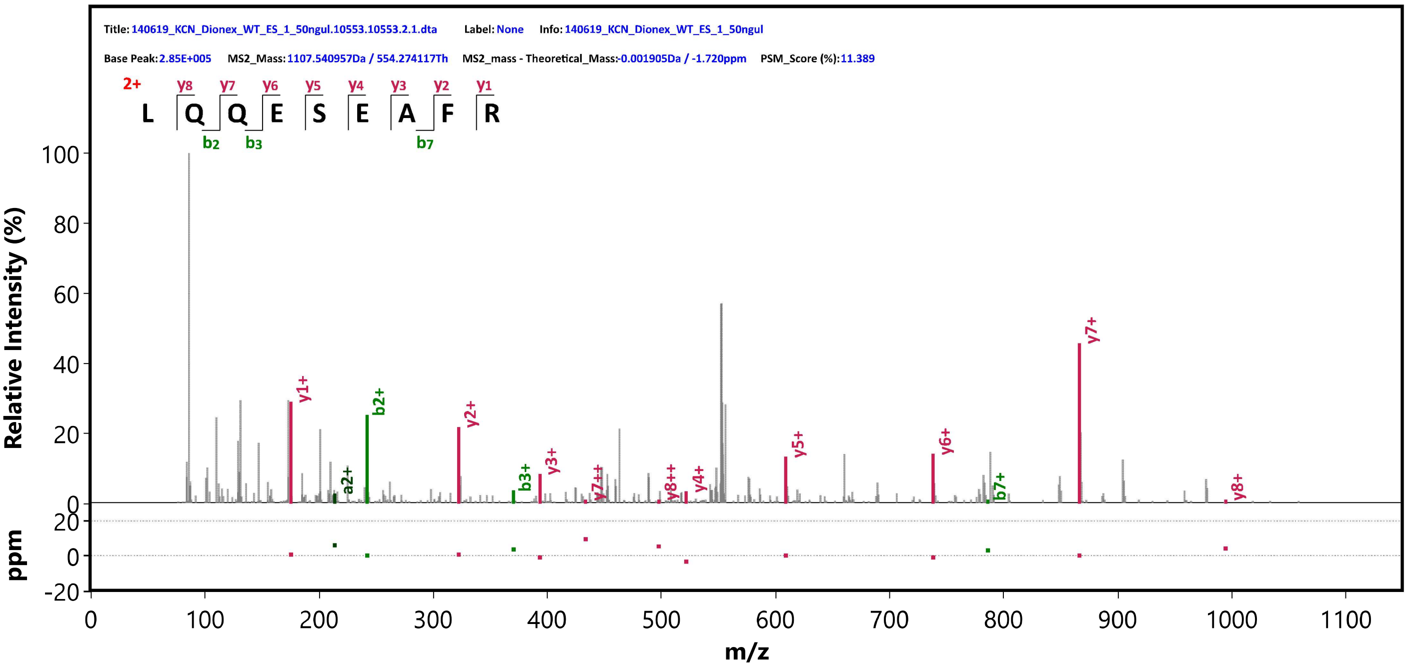

Blackburn[2]

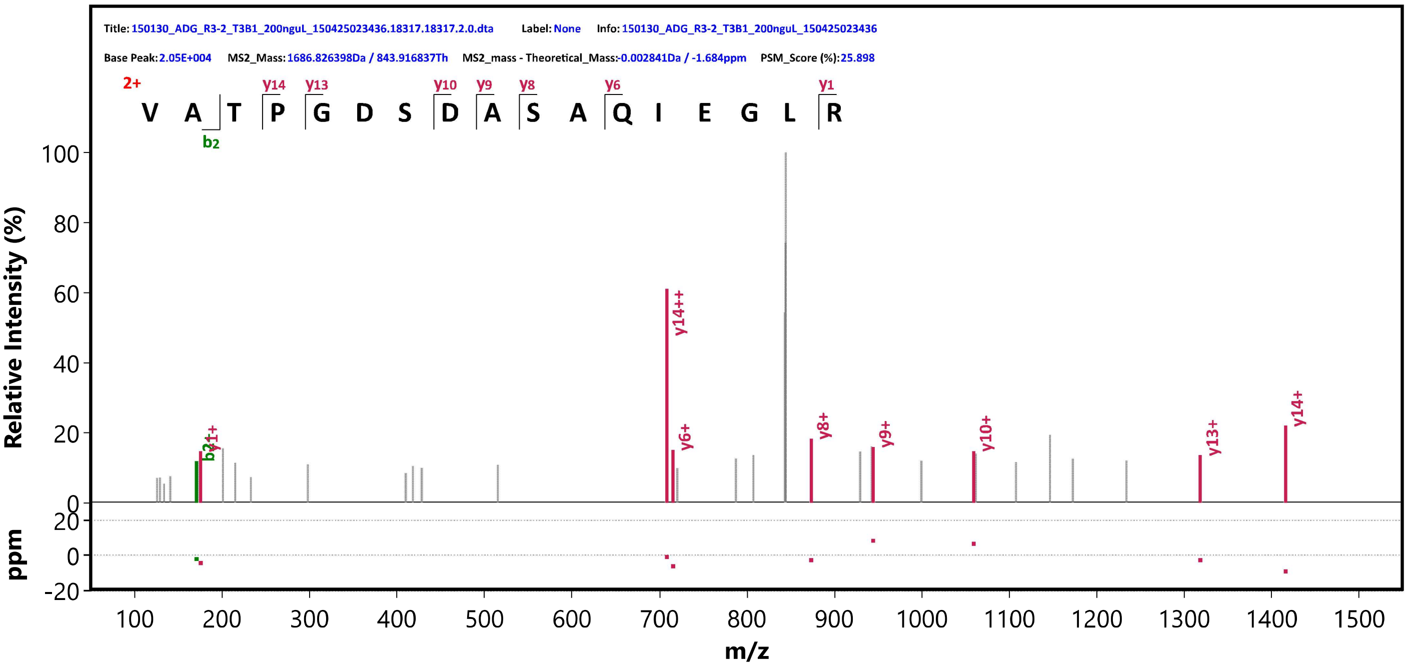

No. 9 orf|0|+|813403-813805|

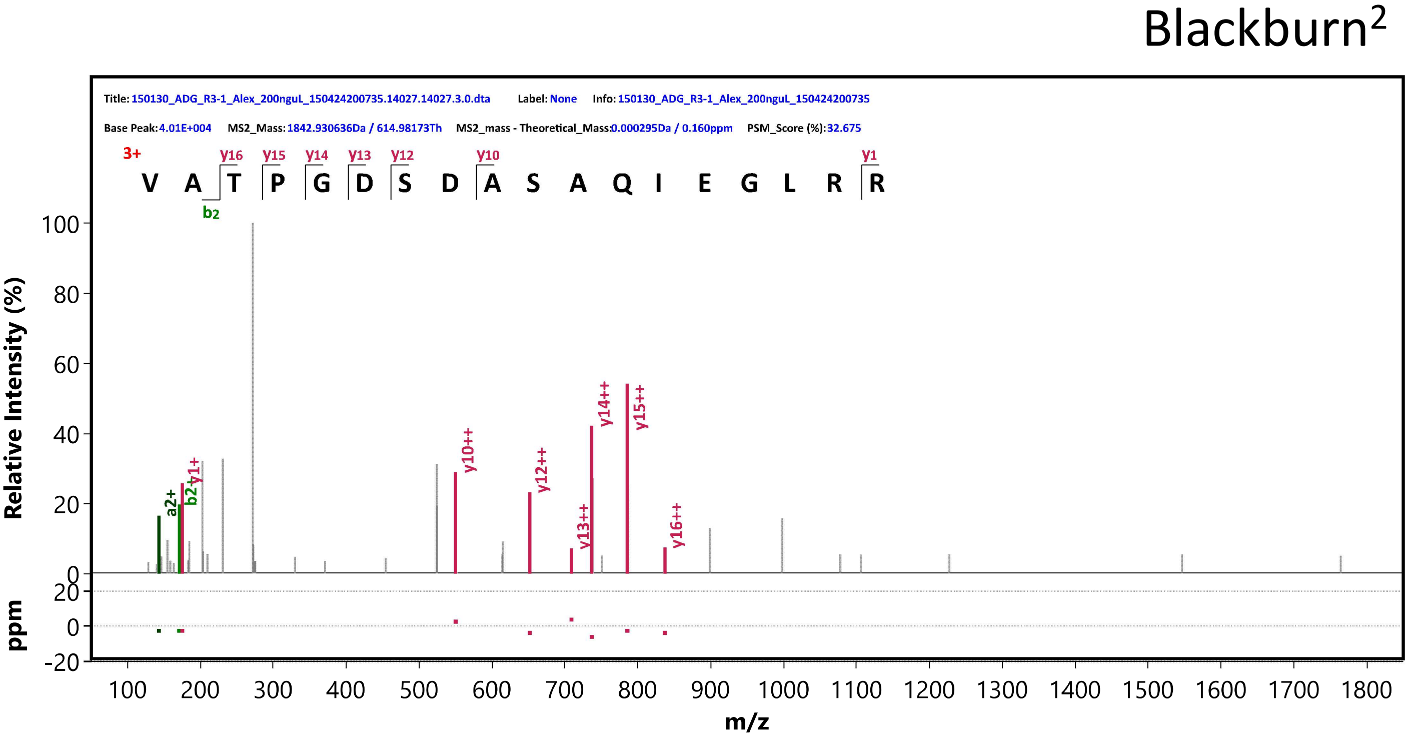

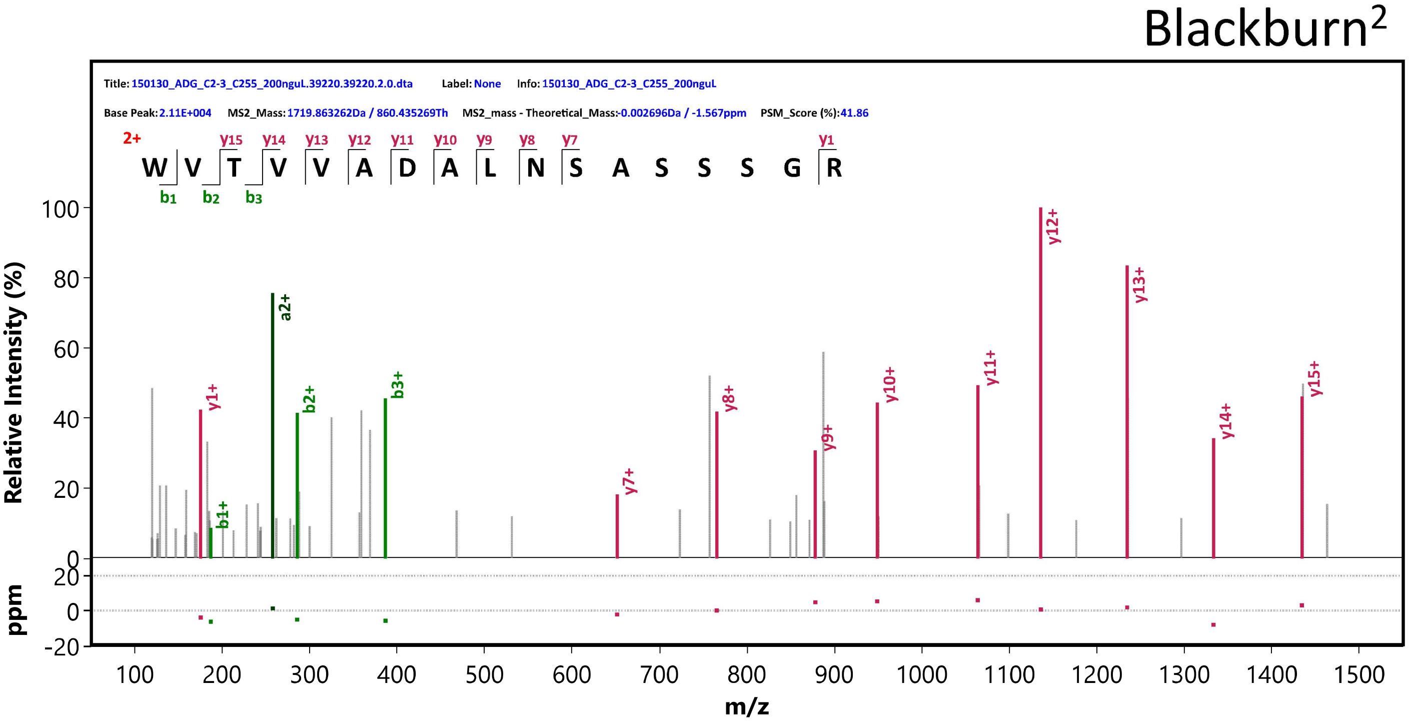

No. 15 orf|0|-|5450187-5451333|

Blackburn[1]

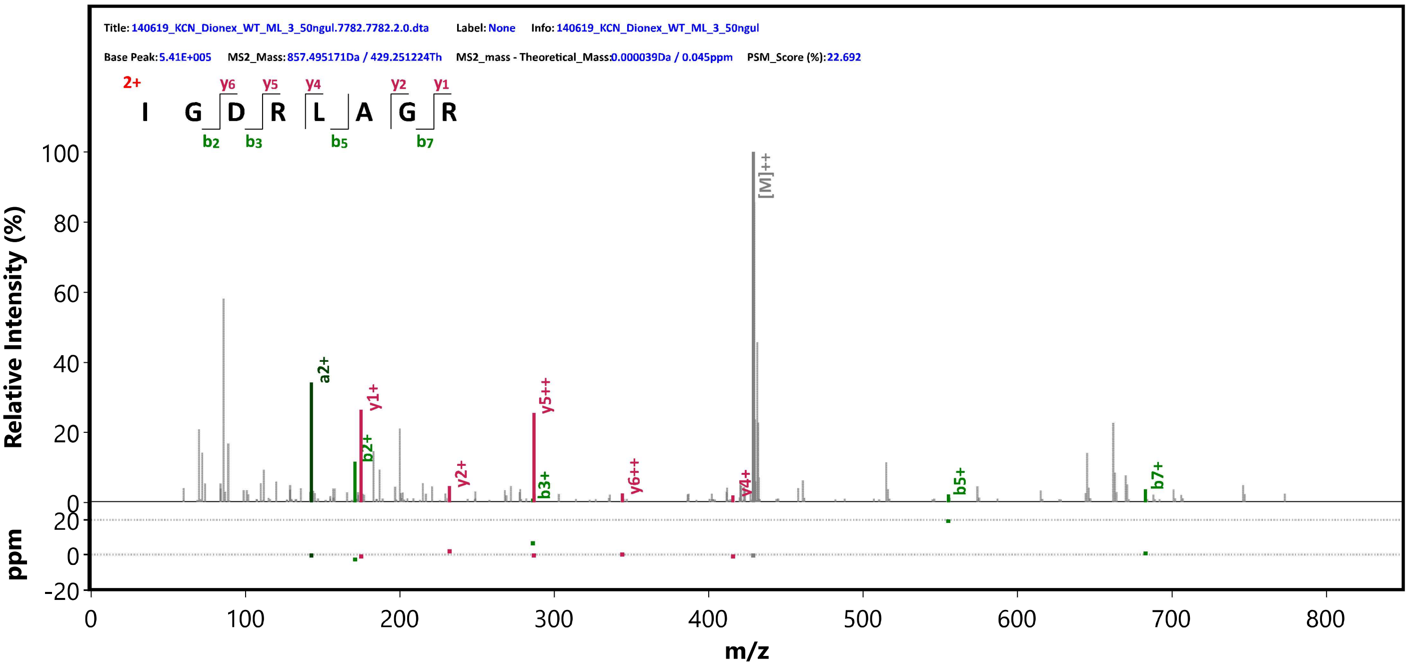

Supplementary Figure 11

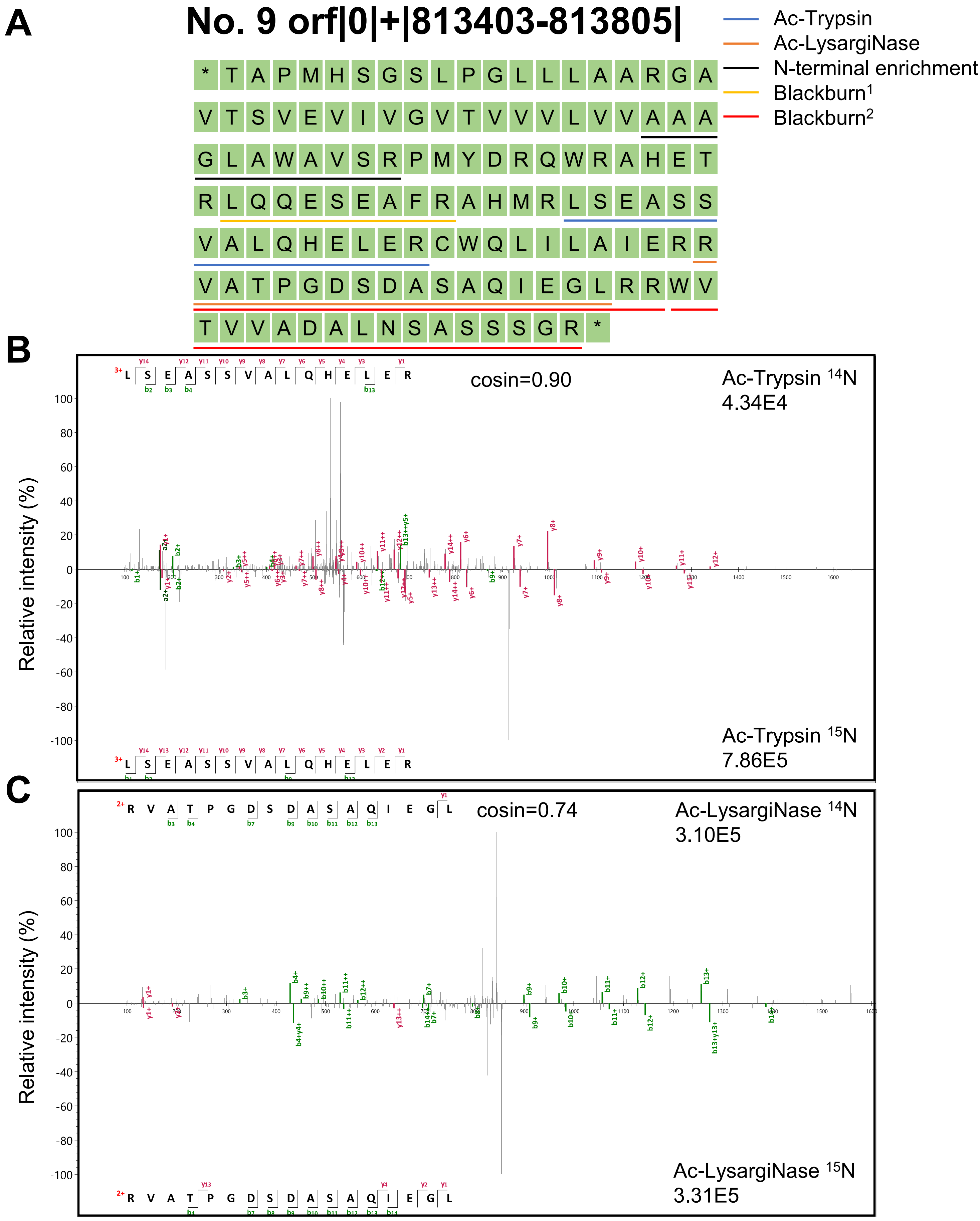

D

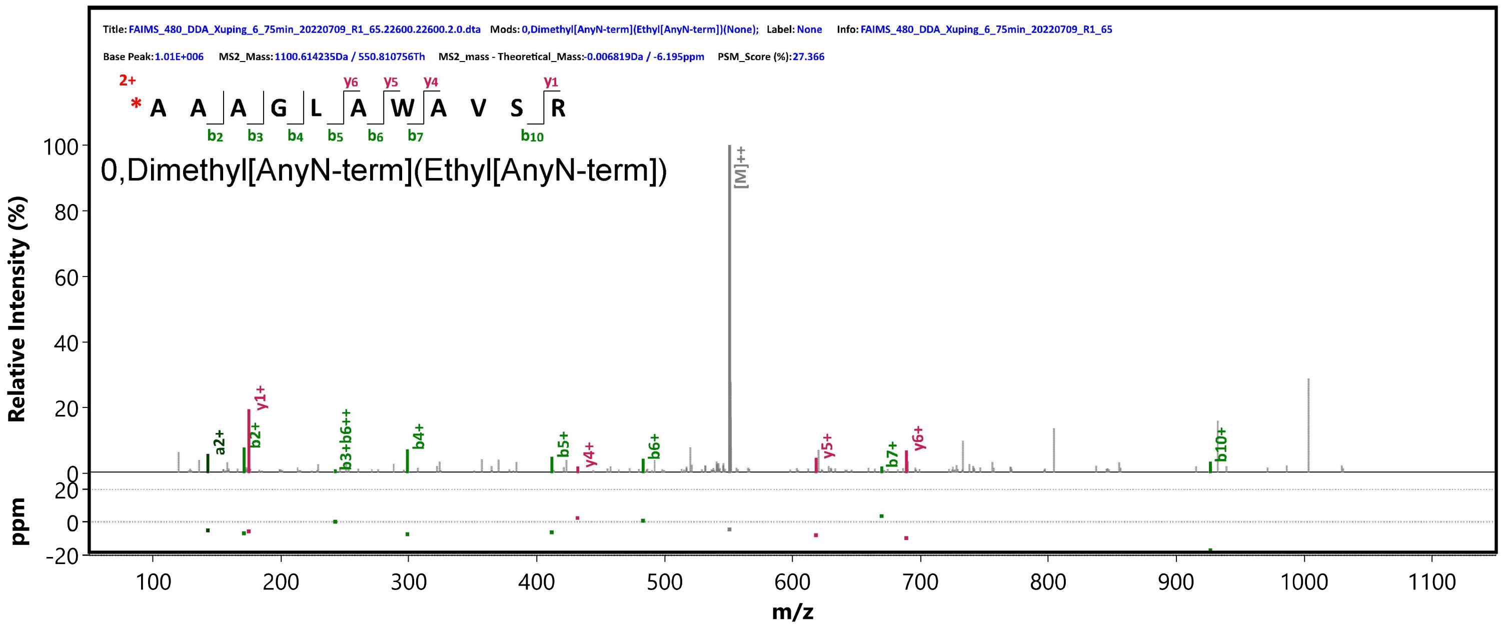
Title: FAIMS_480_DDA_Xuping_6_75min_20220709_R1_65.22600.22600.2.0.dta Mods: 0,Dimethyl[AnyN-term](Ethyl[AnyN-term])(None); Label: None Info: FAIMS_480_DDA_Xuping_6_75min_20220709_R1_65
Base Peak: 1.01E+006 MS2_Mass: 1100.614235Da / 550.810756Th MS2_mass - Theoretical_Mass: -0.006819Da / -6.195ppm PSM_Score (%): 27.366
2+
* A A A G L A W A V S R
0,Dimethyl[AnyN-term](Ethyl[AnyN-term])
[M]++
Relative Intensity (%)
ppm
m/z

Supplementary Figure 12

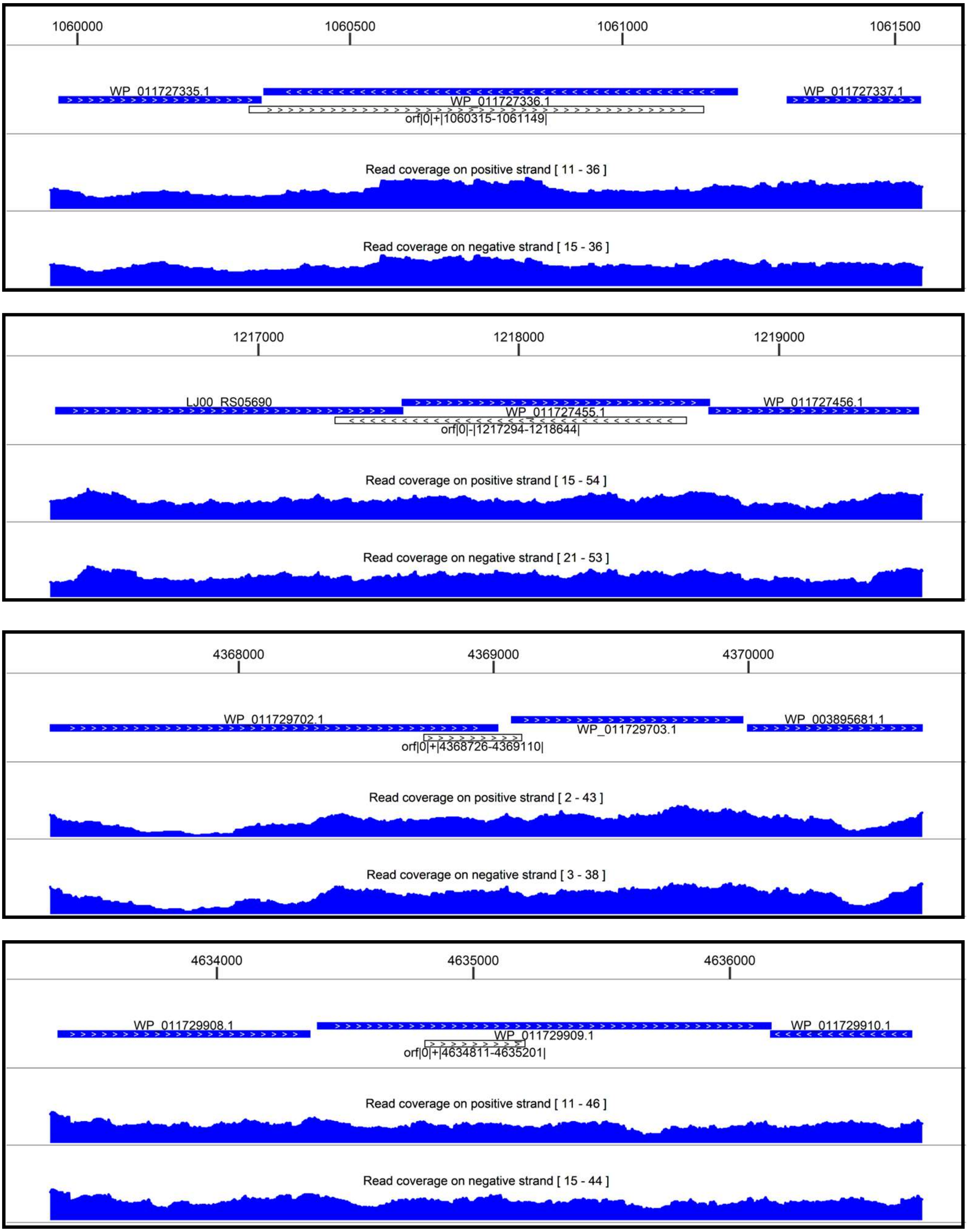

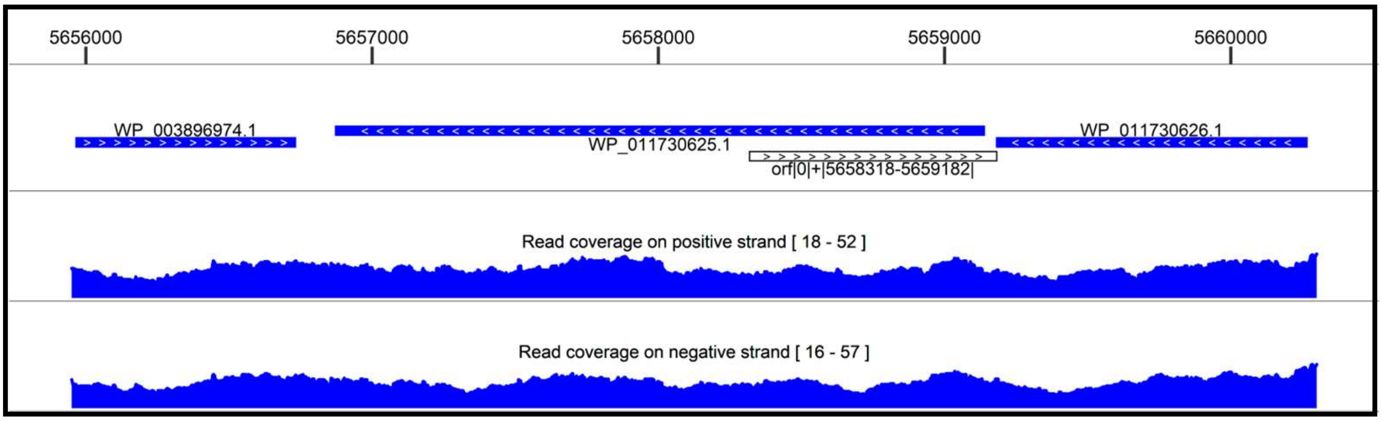

5656000
5657000
5658000
5659000
5660000
WP_003896974.1
WP_011730625.1
WP_011730626.1
orf|0|+|5658318-5659182|
Read coverage on positive strand [18 - 52]
Read coverage on negative strand [16 - 57]

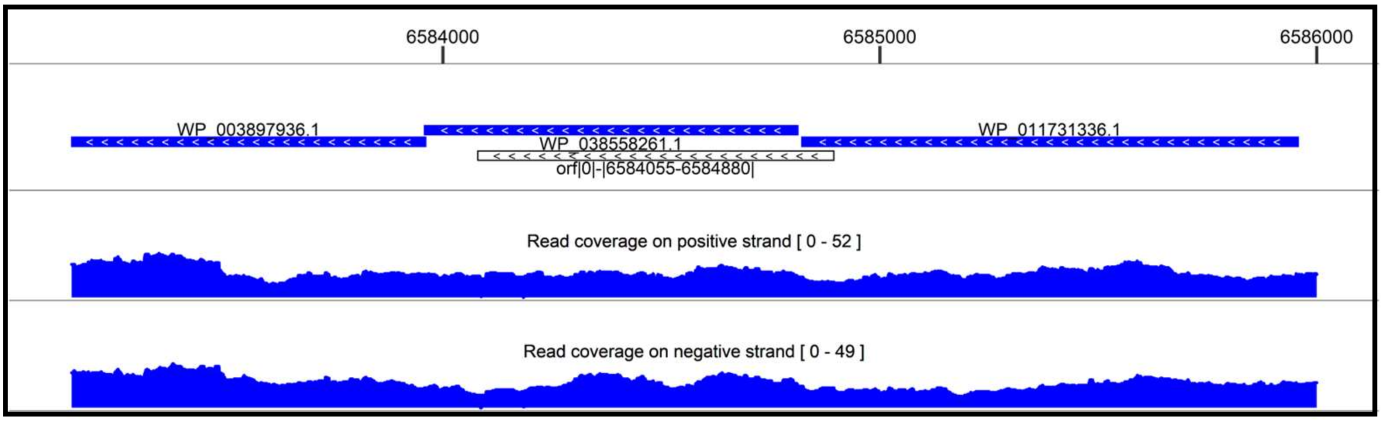

6584000
6585000
6586000
WP_003897936.1
WP_038558261.1
WP_011731336.1
orf|0|-|6584055-6584880|
Read coverage on positive strand [0 - 52]
Read coverage on negative strand [0 - 49]

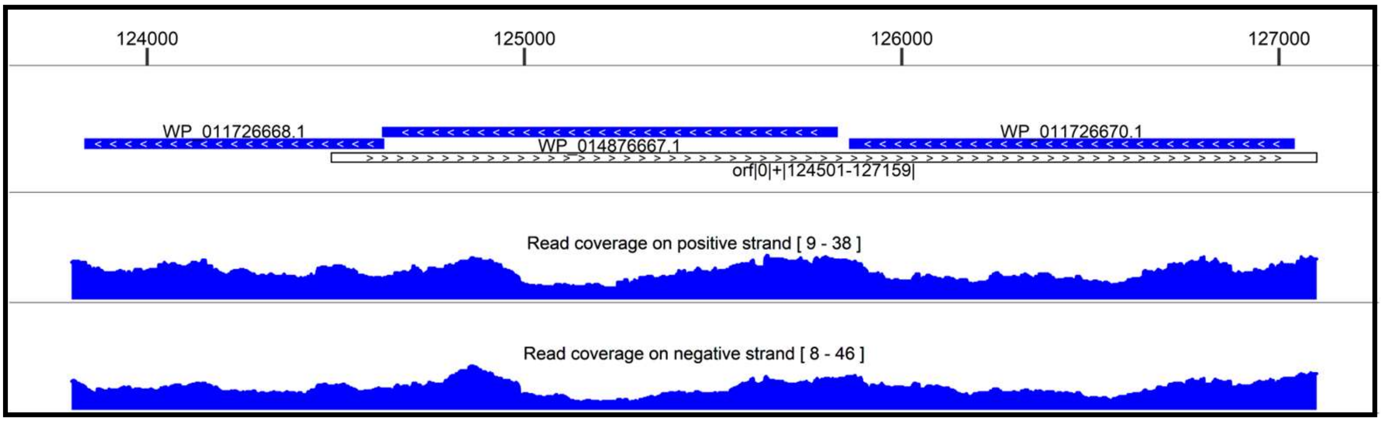

124000
125000
126000
127000
WP_011726668.1
WP_014876667.1
WP_011726670.1
orf|0|+|124501-127159|
Read coverage on positive strand [9 - 38]
Read coverage on negative strand [8 - 46]

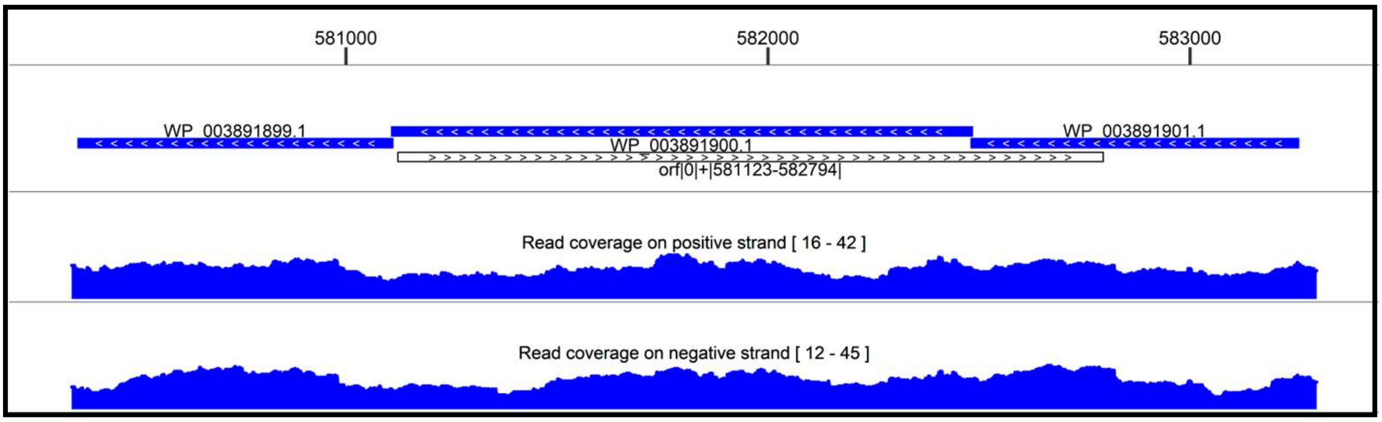

581000
582000
583000
WP_003891899.1
WP_003891900.1
WP_003891901.1
orf|0|+|581123-582794|
Read coverage on positive strand [16 - 42]
Read coverage on negative strand [12 - 45]

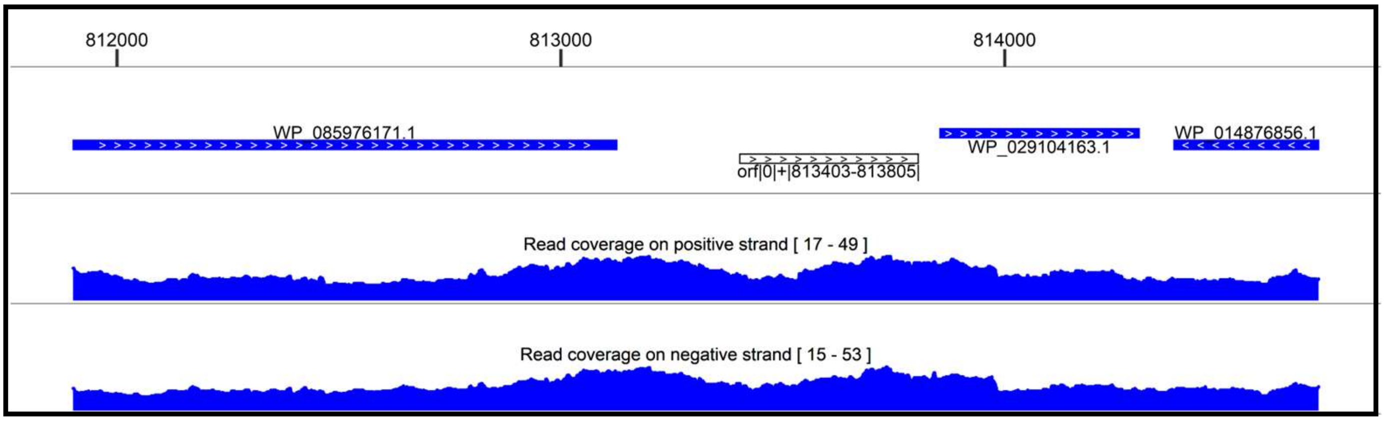
812000
813000
814000
WP_085976171.1
orf|0|+|813403-813805|
WP_029104163.1
WP_014876856.1
Read coverage on positive strand [17 - 49]
Read coverage on negative strand [15 - 53]

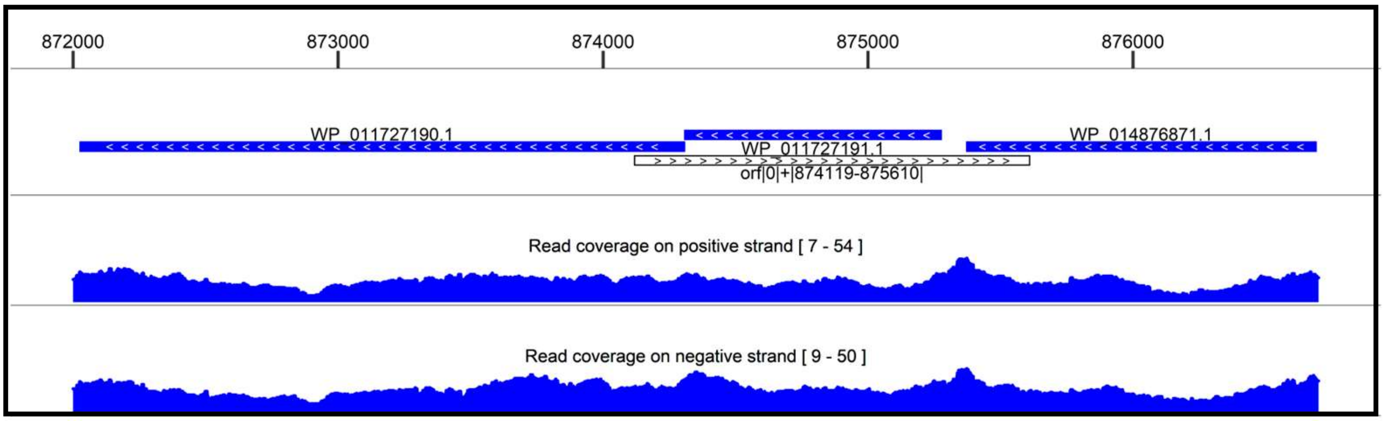
872000
873000
874000
875000
876000
WP_011727190.1
WP_011727191.1
orf|0|+|874119-875610|
WP_014876871.1
Read coverage on positive strand [7 - 54]
Read coverage on negative strand [9 - 50]

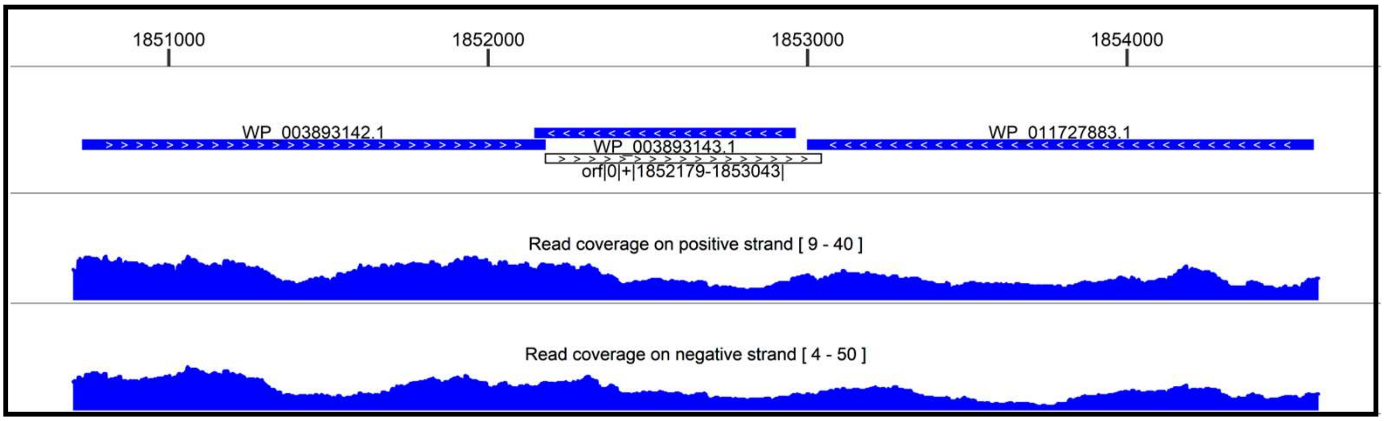
1851000
1852000
1853000
1854000
WP_003893142.1
WP_003893143.1
orf|0|+|1852179-1853043|
WP_011727883.1
Read coverage on positive strand [9 - 40]
Read coverage on negative strand [4 - 50]

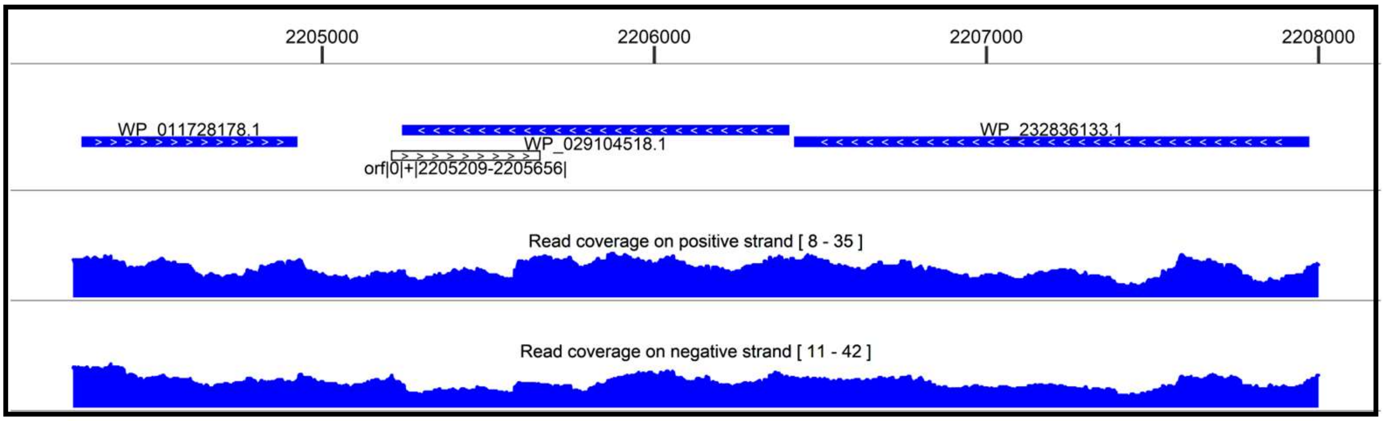
2205000
2206000
2207000
2208000
WP_011728178.1
WP_029104518.1
orf|0|+|2205209-2205656|
WP_232836133.1
Read coverage on positive strand [8 - 35]
Read coverage on negative strand [11 - 42]

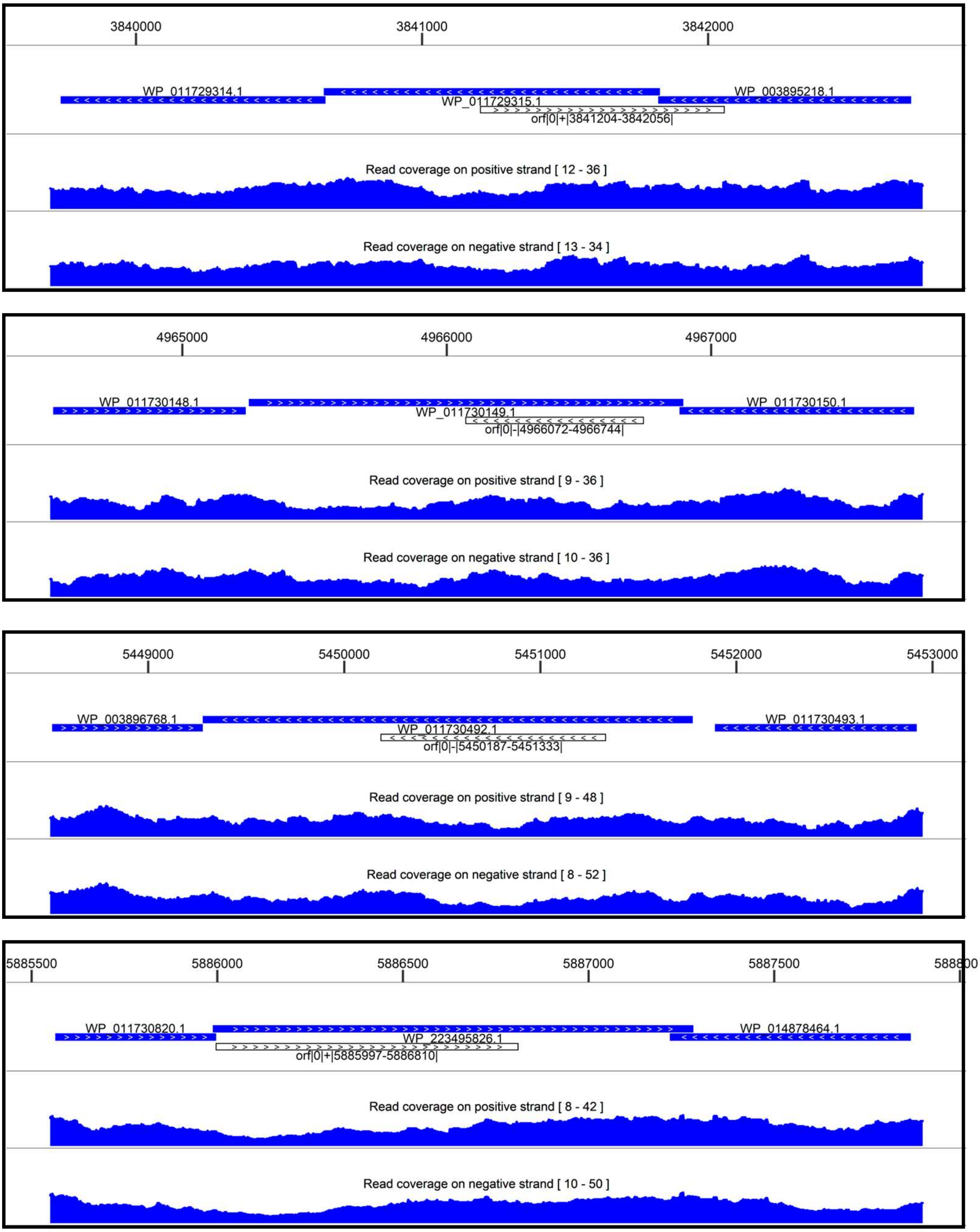
3840000
3841000
3842000
WP_011729314.1
WP_011729315.1
WP_003895218.1
orf|0|+|3841204-3842056|
Read coverage on positive strand [12 - 36]
Read coverage on negative strand [13 - 34]
4965000
4966000
4967000
WP_011730148.1
WP_011730149.1
WP_011730150.1
orf|0|-|4966072-4966744|
Read coverage on positive strand [9 - 36]
Read coverage on negative strand [10 - 36]
5449000
5450000
5451000
5452000
5453000
WP_003896768.1
WP_011730492.1
WP_011730493.1
orf|0|-|5450187-5451333|
Read coverage on positive strand [9 - 48]
Read coverage on negative strand [8 - 52]
5885500
5886000
5886500
5887000
5887500
WP_011730820.1
WP_223495826.1
WP_014878464.1
orf|0|+|5885997-5886810|
Read coverage on positive strand [8 - 42]
Read coverage on negative strand [10 - 50]

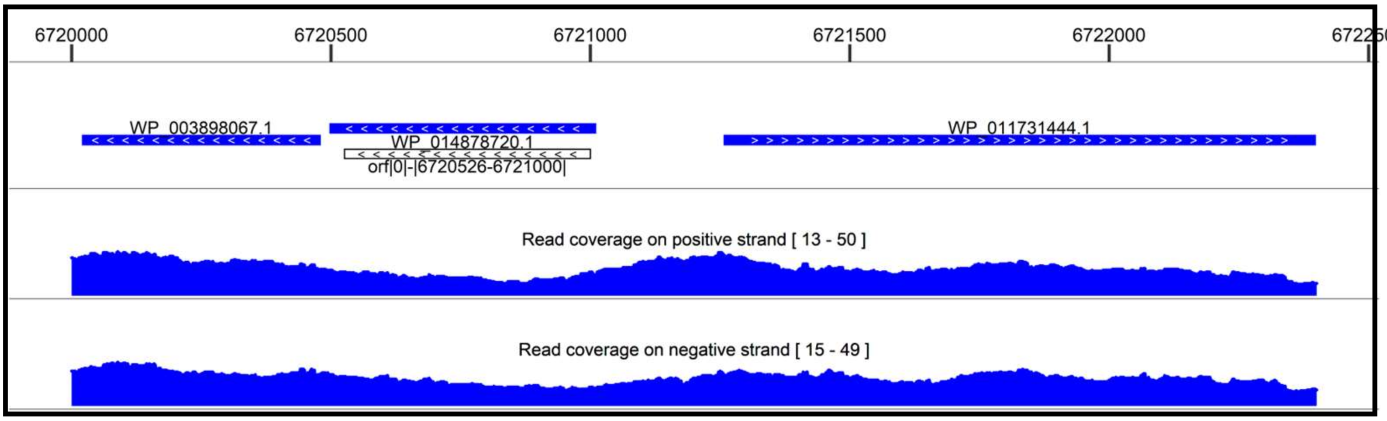
6720000
6720500
6721000
6721500
6722000
WP_003898067.1
WP_014878720.1
orf|0|-|6720526-6721000|
WP_011731444.1
Read coverage on positive strand [13 - 50]
Read coverage on negative strand [15 - 49]

Supplementary Figure 13

A

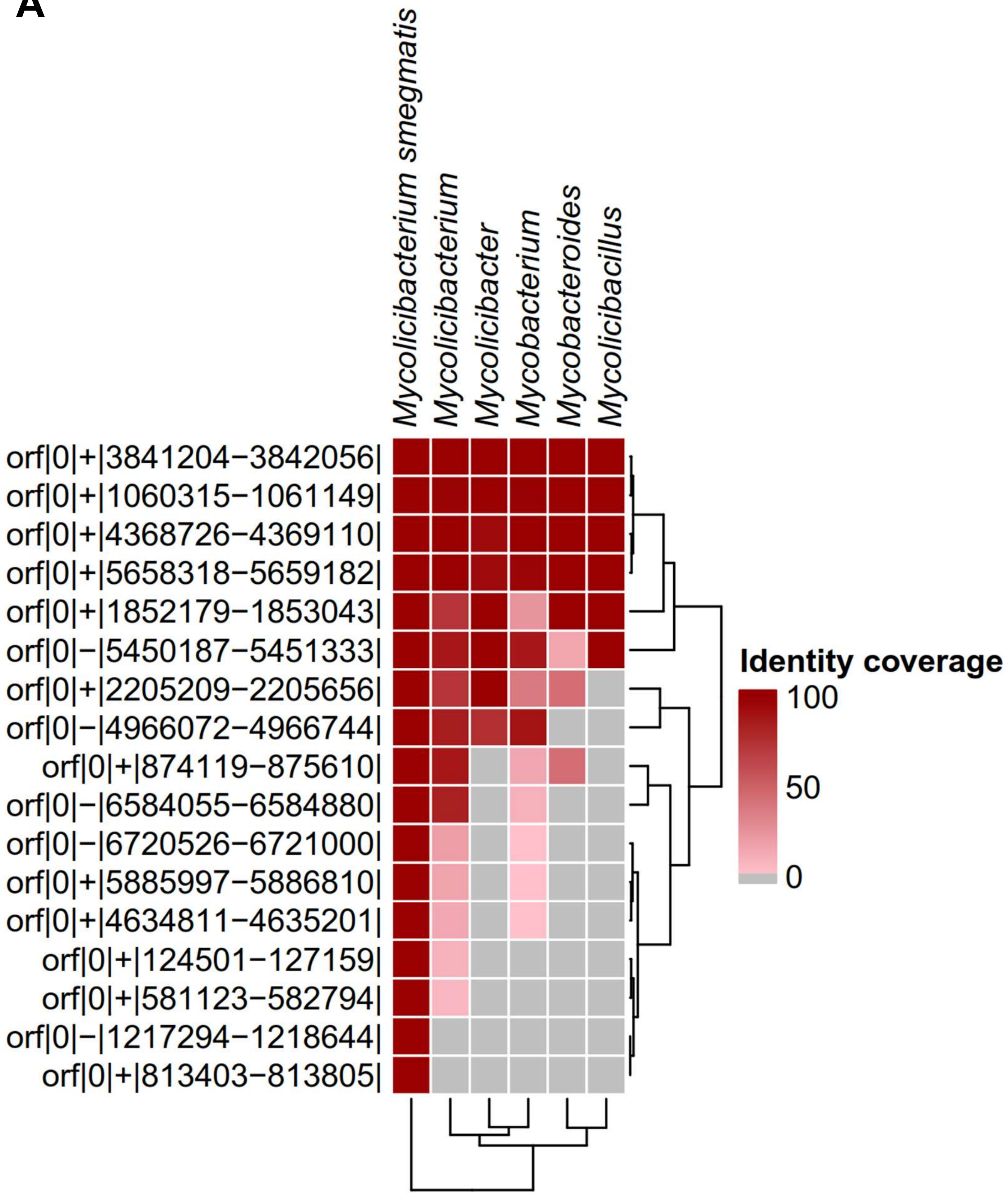

B

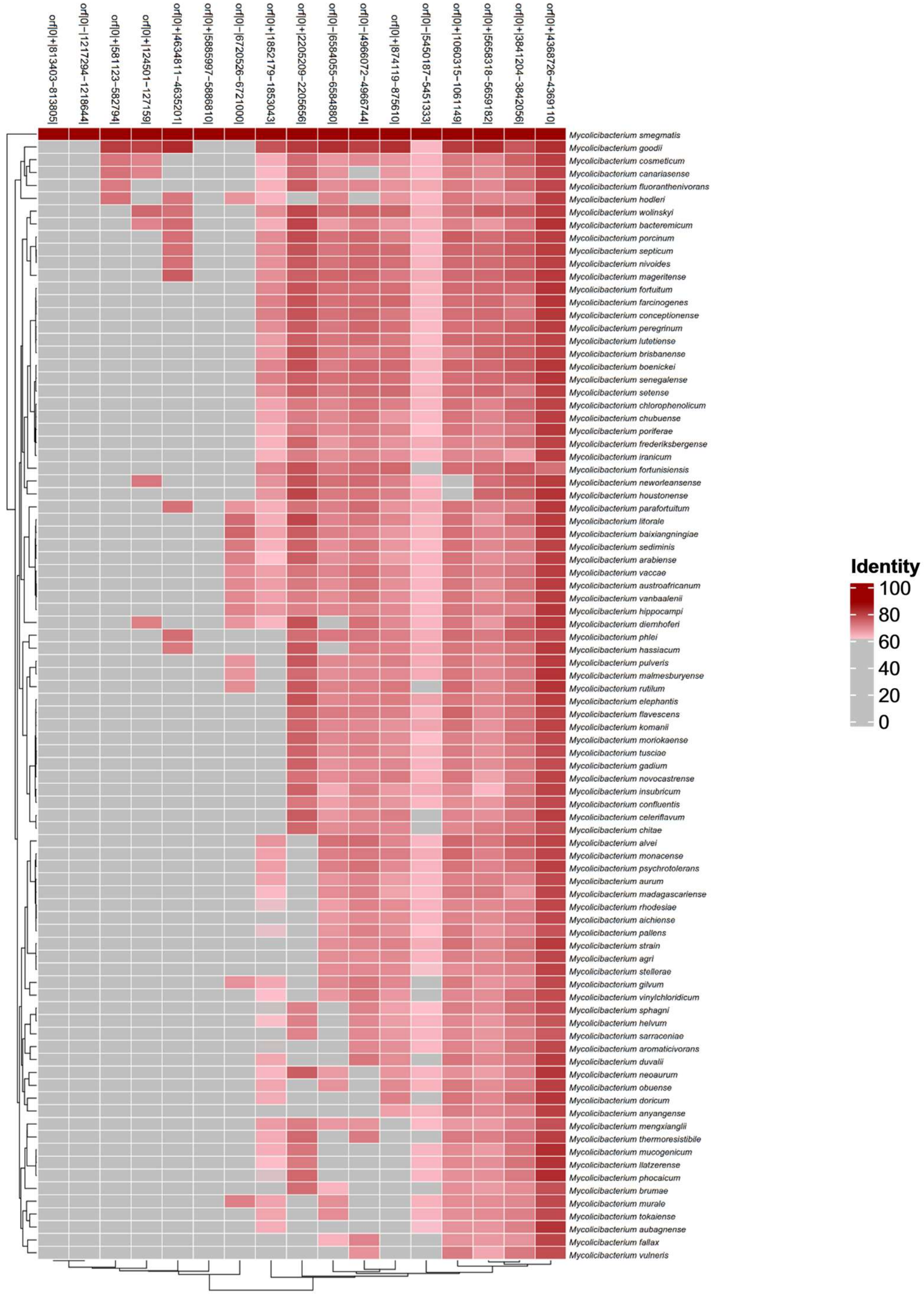
orf|0|+|813403–813805|
orf|0|−|1217294–1218644|
orf|0|+|581123–582794|
orf|0|+|124501–127159|
orf|0|+|4634811–4635201|
orf|0|+|5885997–5886810|
orf|0|−|6720526–6721000|
orf|0|+|1852179–1853043|
orf|0|+|2205209–2205656|
orf|0|−|6584055–6584880|
orf|0|−|4966072–4966744|
orf|0|+|874119–875610|
orf|0|−|5450187–5451333|
orf|0|+|1060315–1061149|
orf|0|+|5658318–5659182|
orf|0|+|3841204–3842056|
orf|0|+|4368726–4369110|
Mycolicibacterium smegmatis
Mycolicibacterium goodii
Mycolicibacterium cosmeticum
Mycolicibacterium canariasense
Mycolicibacterium fluoranthenivorans
Mycolicibacterium hodleri
Mycolicibacterium wolinskyi
Mycolicibacterium bacteremicum
Mycolicibacterium porcinum
Mycolicibacterium septicum
Mycolicibacterium nivoides
Mycolicibacterium mageritense
Mycolicibacterium fortuitum
Mycolicibacterium farcinogenes
Mycolicibacterium conceptionense
Mycolicibacterium peregrinum
Mycolicibacterium lutetiense
Mycolicibacterium brisbanense
Mycolicibacterium boenickei
Mycolicibacterium senegalense
Mycolicibacterium setense
Mycolicibacterium chlorophenolicum
Mycolicibacterium chubuense
Mycolicibacterium poriferae
Mycolicibacterium frederiksbergense
Mycolicibacterium iranicum
Mycolicibacterium fortunisiensis
Mycolicibacterium neworleansense
Mycolicibacterium houstonense
Mycolicibacterium parafortuitum
Mycolicibacterium litorale
Mycolicibacterium baixiangningiae
Mycolicibacterium sediminis
Mycolicibacterium arabiense
Mycolicibacterium vaccae
Mycolicibacterium austroafricanum
Mycolicibacterium vanbaalenii
Mycolicibacterium hippocampi
Mycolicibacterium diernhoferi
Mycolicibacterium phlei
Mycolicibacterium hassiacum
Mycolicibacterium pulveris
Mycolicibacterium malmesburyense
Mycolicibacterium rutilum
Mycolicibacterium elephantis
Mycolicibacterium flavescens
Mycolicibacterium komanii
Mycolicibacterium moriokaense
Mycolicibacterium tusciae
Mycolicibacterium gadium
Mycolicibacterium novocastrense
Mycolicibacterium insubricum
Mycolicibacterium confluentis
Mycolicibacterium celeriflavum
Mycolicibacterium chitae
Mycolicibacterium alvei
Mycolicibacterium monacense
Mycolicibacterium psychrotolerans
Mycolicibacterium aurum
Mycolicibacterium madagascariense
Mycolicibacterium rhodesiae
Mycolicibacterium aichiense
Mycolicibacterium pallens
Mycolicibacterium strain
Mycolicibacterium agri
Mycolicibacterium stellerae
Mycolicibacterium gilvum
Mycolicibacterium vinylchloridicum
Mycolicibacterium sphagni
Mycolicibacterium helvum
Mycolicibacterium sarraceniae
Mycolicibacterium aromaticivorans
Mycolicibacterium duvalii
Mycolicibacterium neoaurum
Mycolicibacterium obuense
Mycolicibacterium doricum
Mycolicibacterium anyangense
Mycolicibacterium mengxianglii
Mycolicibacterium thermoresistibile
Mycolicibacterium mucogenicum
Mycolicibacterium llatzerense
Mycolicibacterium phocaicum
Mycolicibacterium brumae
Mycolicibacterium murale
Mycolicibacterium tokaiense
Mycolicibacterium aubagnense
Mycolicibacterium fallax
Mycolicibacterium vulneris
Identity
100
80
60
40
20
0

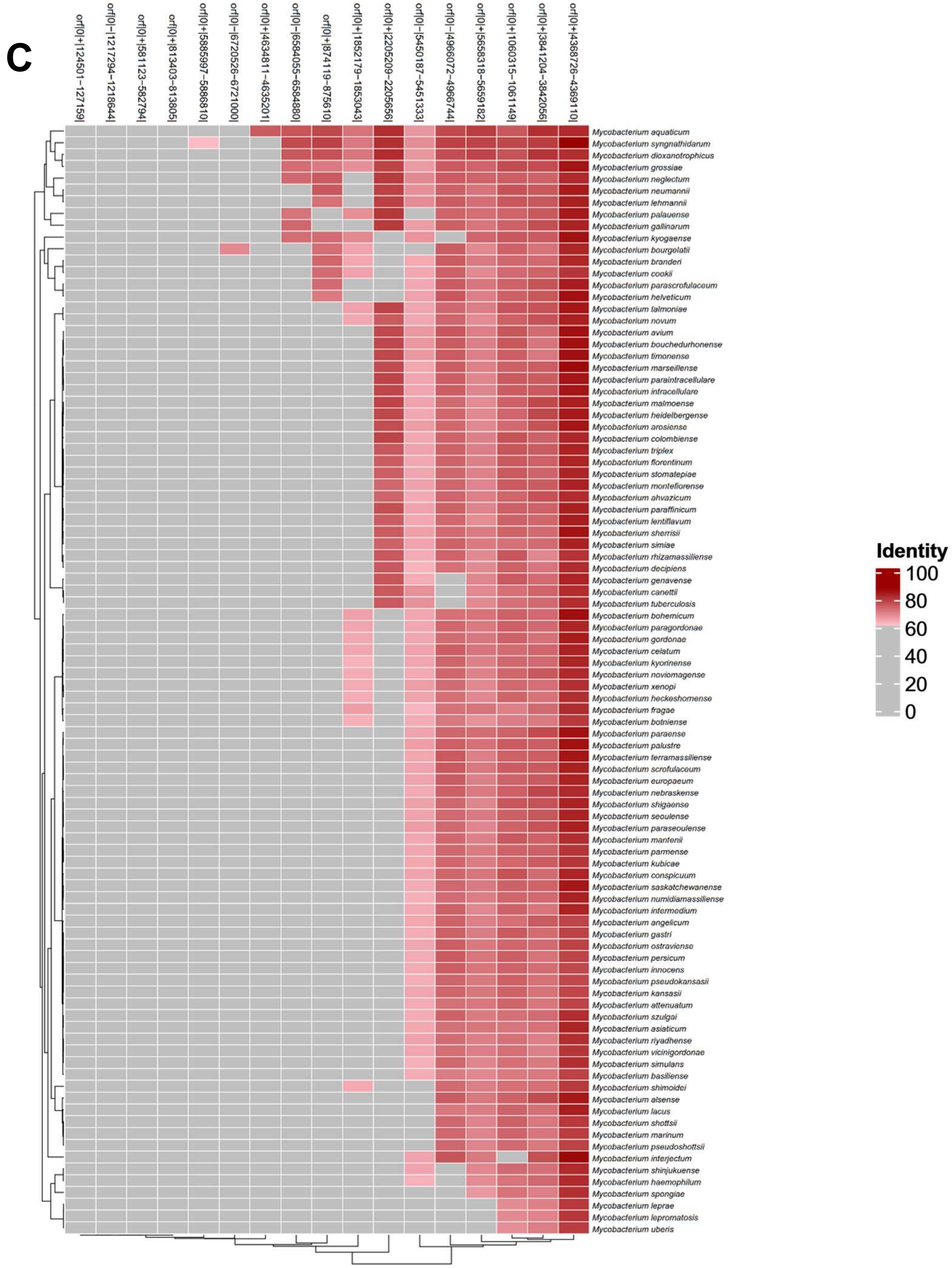
C
orf|0|+|124501–127159|
orf|0|–|1217294–1218644|
orf|0|+|581123–582794|
orf|0|+|813403–813805|
orf|0|+|5885997–5886810|
orf|0|–|6720526–6721000|
orf|0|+|4634811–4635201|
orf|0|–|6584055–6584880|
orf|0|+|874119–875610|
orf|0|+|1852179–1853043|
orf|0|+|2205209–2205656|
orf|0|–|5450187–5451333|
orf|0|–|4966072–4966744|
orf|0|+|5658318–5659182|
orf|0|+|1060315–1061149|
orf|0|+|3841204–3842056|
orf|0|+|4368726–4369110|
Mycobacterium aquaticum
Mycobacterium syngnathidarum
Mycobacterium dioxanotrophicus
Mycobacterium grossiae
Mycobacterium neglectum
Mycobacterium neumannii
Mycobacterium lehmannii
Mycobacterium palauense
Mycobacterium gallinarum
Mycobacterium kyogaense
Mycobacterium bourgelatii
Mycobacterium branderi
Mycobacterium cookii
Mycobacterium parascrofulaceum
Mycobacterium helveticum
Mycobacterium talmoniae
Mycobacterium novum
Mycobacterium avium
Mycobacterium bouchedurhonense
Mycobacterium timonense
Mycobacterium marseillense
Mycobacterium paraintracellulare
Mycobacterium intracellulare
Mycobacterium malmoense
Mycobacterium heidelbergense
Mycobacterium arosiense
Mycobacterium colombiense
Mycobacterium triplex
Mycobacterium florentinum
Mycobacterium stomatepiae
Mycobacterium montefiorense
Mycobacterium ahvazicum
Mycobacterium paraffinicum
Mycobacterium lentiflavum
Mycobacterium sherrisii
Mycobacterium simiae
Mycobacterium rhizamassiliense
Mycobacterium decipiens
Mycobacterium genavense
Mycobacterium canettii
Mycobacterium tuberculosis
Mycobacterium bohemicum
Mycobacterium paragordonae
Mycobacterium gordonae
Mycobacterium celatum
Mycobacterium kyorinense
Mycobacterium noviomagense
Mycobacterium xenopi
Mycobacterium heckeshornense
Mycobacterium fragae
Mycobacterium botniense
Mycobacterium paraense
Mycobacterium palustre
Mycobacterium terramassiliense
Mycobacterium scrofulaceum
Mycobacterium europaeum
Mycobacterium nebraskense
Mycobacterium shigaense
Mycobacterium seoulense
Mycobacterium paraseoulense
Mycobacterium mantenii
Mycobacterium parmense
Mycobacterium kubicae
Mycobacterium conspicuum
Mycobacterium saskatchewanense
Mycobacterium numidiamassiliense
Mycobacterium intermedium
Mycobacterium angelicum
Mycobacterium gastri
Mycobacterium ostraviense
Mycobacterium persicum
Mycobacterium innocens
Mycobacterium pseudokansasii
Mycobacterium kansasii
Mycobacterium attenuatum
Mycobacterium szulgai
Mycobacterium asiaticum
Mycobacterium riyadhense
Mycobacterium vicinigordonae
Mycobacterium simulans
Mycobacterium basiliense
Mycobacterium shimoidei
Mycobacterium alsense
Mycobacterium lacus
Mycobacterium shottsii
Mycobacterium marinum
Mycobacterium pseudoshottsii
Mycobacterium interjectum
Mycobacterium shinjukuense
Mycobacterium haemophilum
Mycobacterium spongiae
Mycobacterium leprae
Mycobacterium lepromatosis
Mycobacterium uberis
Identity
100
80
60
40
20
0

D

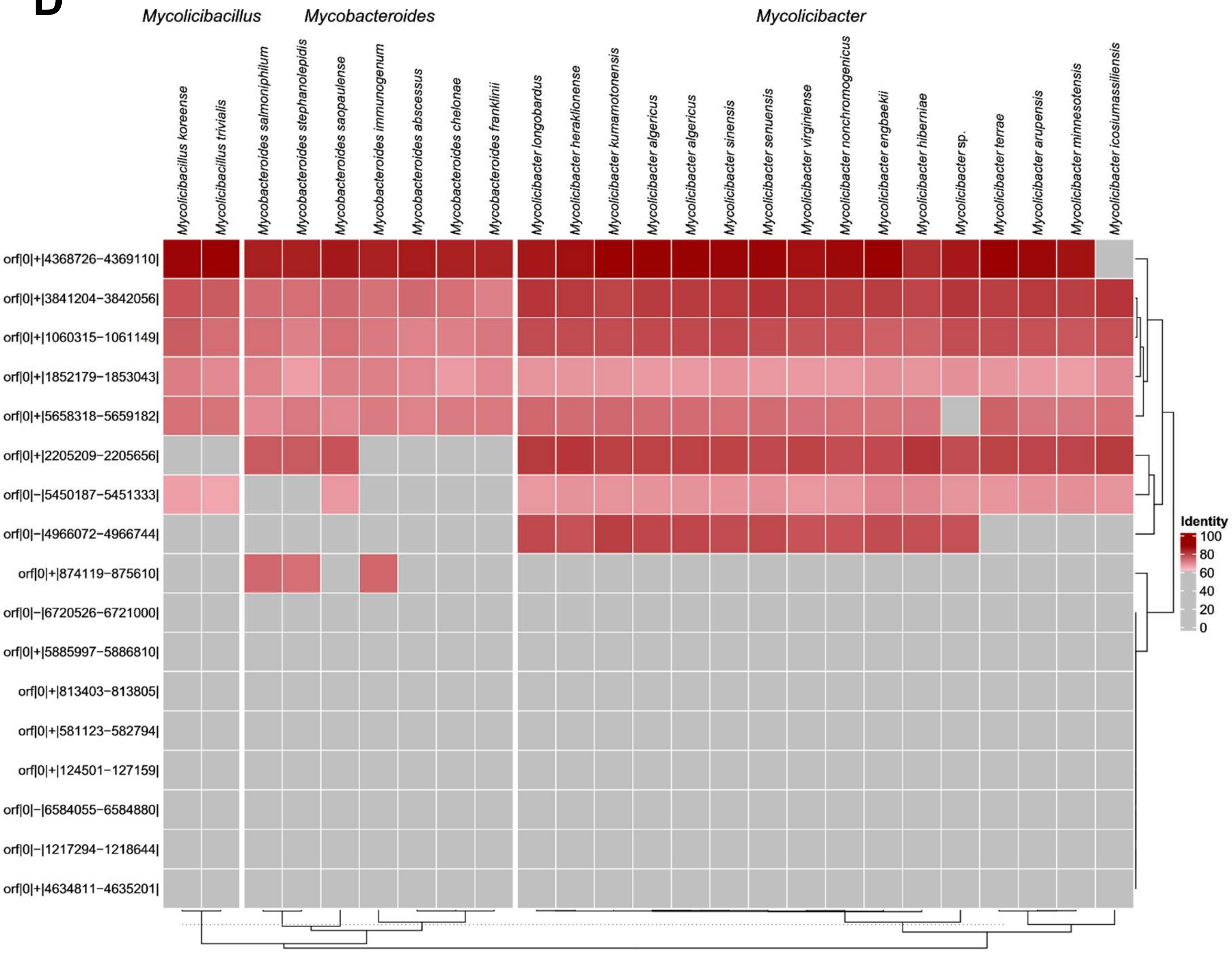
Mycolicibacillus
Mycobacteroides
Mycolicibacter
Mycolicibacillus koreense
Mycolicibacillus trivialis
Mycobacteroides salmoniphilum
Mycobacteroides stephanolepidis
Mycobacteroides saopaulense
Mycobacteroides immunogenum
Mycobacteroides abscessus
Mycobacteroides chelonae
Mycobacteroides franklinii
Mycolicibacter longobardus
Mycolicibacter heraklionense
Mycolicibacter kumamotonensis
Mycolicibacter algericus
Mycolicibacter algericus
Mycolicibacter sinensis
Mycolicibacter senuensis
Mycolicibacter virginiense
Mycolicibacter nonchromogenicus
Mycolicibacter engbaekii
Mycolicibacter hiberniae
Mycolicibacter sp.
Mycolicibacter terrae
Mycolicibacter arupensis
Mycolicibacter minnesotensis
Mycolicibacter icosiumassiliensis
orf|0|+|4368726−4369110|
orf|0|+|3841204−3842056|
orf|0|+|1060315−1061149|
orf|0|+|1852179−1853043|
orf|0|+|5658318−5659182|
orf|0|+|2205209−2205656|
orf|0|−|5450187−5451333|
orf|0|−|4966072−4966744|
orf|0|+|874119−875610|
orf|0|−|6720526−6721000|
orf|0|+|5885997−5886810|
orf|0|+|813403−813805|
orf|0|+|581123−582794|
orf|0|+|124501−127159|
orf|0|−|6584055−6584880|
orf|0|−|1217294−1218644|
orf|0|+|4634811−4635201|
Identity
100
80
60
40
20
0
